# Synthesis of Molecular Phenylcalcium Derivatives: Application to the Formation of Biaryls

**DOI:** 10.1002/anie.202200305

**Published:** 2022-03-07

**Authors:** Kyle G. Pearce, Chiara Dinoi, Michael S. Hill, Mary F. Mahon, Laurent Maron, Ryan S. Schwamm, Andrew S. S. Wilson

**Affiliations:** ^1^ Department of Chemistry University of Bath Claverton Down, Bath UK; ^2^ Université de Toulouse et CNRS INSA UPS UMR 5215 LPCNO 135 Avenue de Rangueil 31077 Toulouse France

**Keywords:** Aryl Bromides, Biaryls, Calcium, Density Functional Theory, Main Group Chemistry

## Abstract

Hydrocarbon‐soluble β‐diketiminato phenylcalcium derivatives, which display various modes of Ca−μ_2_‐Ph−Ca bridging, are accessible from reactions of Ph_2_Hg and [(BDI)CaH]_2_. Although the resultant compounds are inert toward the C−H bonds of benzene, they yield selective and uncatalyzed biaryl formation when reacted with readily available aryl bromides.

## Graphical abstract

Isolable and hydrocarbon‐soluble β‐diketiminato calcium phenyls are accessible from the reaction of Ph_2_Hg and [(BDI)CaH]_2_. The compounds were shown to react with bromoarenes through a direct S_N_Ar displacement of halide, leading to the uncatalyzed formation of biaryl molecules.

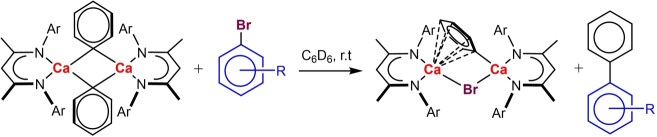

Organocalcium synthesis and the organometallic chemistry of calcium's heavier Group 2 congeners (Ae=Ca, Sr and Ba) has advanced rapidly during the past 20 years.^[1]^ Reports of σ‐C−Ca‐bonded calcium alkyl and aryls date from as early as 1905.^[2]^ For much of the 20^th^ Century, however, they remained incompletely characterized curiosities and the first crystallographically confirmed calcium σ‐alkyl derivative, [Ca{CH(SiMe_3_)_2_}_2_(Diox)_2_] (**1**; Diox=1,4‐dioxane), was only reported in 1991.^[3]^ Since that time, a realisation that such species can provide a distinctive addition to the synthetic canon, complementing or surpassing their lighter congener magnesium, has prompted the emergence of heavier Group 2 organometallic chemistry as a defined area of study in its own right.^[4]^ A case in point is provided by the heavier alkaline earth (Ae) σ‐alkyls derived from the β‐diketiminato hydride derivatives, [(BDI)AeH]_2_ (**2**: Ae=Ca, BDI=HC{(Me)CN‐2,6‐*i*‐Pr_2_C_6_H_3_}_2_=^Dipp^BDI;^[5]^ **3**: Ae=Sr, BDI=HC{(Me)CN‐2,6‐(Et_2_CH)_2_C_6_H_3_}_2_=^DIPeP^BDI).^[6]^ Compounds **2** and **3** react in a stepwise fashion with terminal alkenes to provide the corresponding dinuclear organometallics, which alkylate benzene solvent through the regeneration of [(BDI)AeH]_2_ (Scheme 1a). This latter reactivity has been reasoned to be a consequence of both the extreme nucleophilicity of the σ‐organyl species and the relative coordinative unsaturation of the highly electropositive Group 2 metal centers.[^5a^, ^6^]

**Scheme 1:**
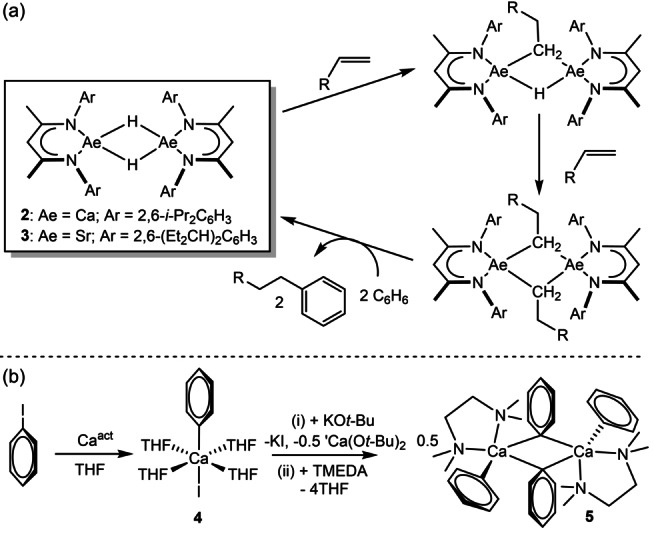
a) Reactivity of compounds **2** and **3** with alkenes and the nucleophilic alkylation of benzene by the resultant σ‐alkyl derivatives; b) direct synthesis of phenylcalcium iodide and diphenylcalcium.

Although sporadic reports of comparable arylcalcium compounds have appeared for over a century,[^2a^, ^2d^, ^7^] Westerhausen and co‐workers have only relatively recently devised a palette of synthetic methods that enable reliable access to Ca−C(*sp* ^*2*^) bonded compounds.[^7^, ^8^] The direct synthesis of a diverse assortment of arylcalcium halides, ArCaX(L)_*n*_ (X=Br or I; L=e.g. ether or amine donor),[^8e^, ^9^] has most commonly been achieved by the reaction of the aryl halides in THF with calcium metal that has been pre‐activated by dissolution in liquid ammonia.^[7a]^ Although species such as the parent phenylcalcium iodide (**4**, Scheme 1b) tend to crystallize as molecular compounds with terminal Ca−C interactions, conventional Ca−μ_2_‐C(*sp* ^2^)−Ca bridging is displayed by diorganocalcium derivatives such as [Ph_2_Ca(TMEDA)]_2_ (**5**; TMEDA=tetramethylethylenediamine), which is itself prepared by the induced Schlenk equilibration of compound **4**.^[8e]^ Similar Ca−μ_2_‐C(*sp* ^2^)−Ca bridging has also been observed in the bimetallic ion pair species, [(THF)_3_Ca(μ‐Ph)_3_Ca(THF)]^+^[(THF)_2_PhCa(μ‐Ph)_3_MnPh]^−^ (**6**) and [(THF)_3_Ca(μ‐Ph)_3_Ca(THF)]^+^[Ph_2_Cu]^−^ (**7**), albeit these compounds were prepared by redox transmetallation of ammonia‐activated calcium with diphenylmanganese and phenylcopper, respectively.[^8a^, ^8e^] These latter methods are also redolent of Niemeyer's earlier Ae reduction (Ae=Ca, Sr, Ba) of (C_6_F_5_)_2_Hg to synthesize terminal pentafluorophenyl derivatives supported by a sterically demanding triazenide ligand.^[10]^ Compounds such as **4** and **5** have been shown to display a limited range of reactivity with various electrophiles and C−H acids.[^8f^, ^8m^, ^11^] By analogy to the nucleophilic reactivity summarised in Scheme 1a, however, we speculated that access to more coordinatively unsaturated σ‐aryl derivatives may provide related C(*sp* ^2^)−C(*sp* ^*2*^) bond forming reactivity with the C−H bonds of benzene or the C−X bonds of related arene derivatives. Although the former supposition has proven incorrect, in this contribution, we report that β‐diketiminato calcium phenyl derivatives allow uncatalyzed access to biaryl molecules by direct S_N_Ar displacement of halide from bromoarenes.

With the production of compounds **6** and **7** in mind, we speculated that the reducing character of compound **2** would behave similarly towards sacrificial equivalents of aryl reagents of less electropositive metals. Circumventing the use of potentially explosive phenylcopper, we first assayed the reactivity of compound **2** with mesityl copper in benzene at room temperature, which induced the formation of a predominant product, compound **8**, over the course of several days.^[8h]^ Although **8** was identified by single crystal X‐ray diffraction analysis as an unusual μ_3_‐H‐bridged dimesitylcuprate derivative, [{(^Dipp^BDI)Ca}_2_(μ‐H){(2,4,6‐Me_3_C_6_H_2_)_2_Cu}] (see Figure S75), all attempts to isolate a pure bulk sample were frustrated by its solution instability. These processes were observed to induce apparent copper metal deposition. Analysis of the solution by ^1^H NMR spectroscopy, however, indicated that it was accompanied by the formation of a variety of products, including ^Dipp^BDI‐H and [(^Dipp^BDI)_2_Ca],^[12]^ and no definitive evidence for the formation of a desired mesitylcalcium species could be observed.

In contrast to these observations, reactions of **2** with Ph_2_Hg induced a rapid bubbling and the formation of a grey precipitate, assumed to be elemental mercury. The identities of the desired phenylcalcium products were found to be dependent on the reaction stoichiometry (Scheme 2). Monitoring of the reaction in a **2**:Ph_2_Hg stoichiometry of 1 : 0.5 by ^1^H NMR spectroscopy demonstrated the appearance of a characteristic series of well‐defined resonances between *δ*=6.5–6.9 ppm that were assigned to the *o‐*, *m*‐ and *p‐*CH environments of a new phenylcalcium species (**9**). These signals emerged simultaneously alongside a new (1H) Ca−*H* environment at *δ*=4.81 ppm while, in common with previously described phenylcalcium derivatives such as **4** and **5**,[^8e^, ^9^] a low field resonance at *δ*=180.7 ppm was observed in the ^13^C{^1^H} NMR experiment. This latter signal did not provide any correlation in the corresponding HSQC experiment and was, thus, assigned as the *ipso*‐carbon of a calcium‐bound phenyl residue. Although proceeding via an unidentified mechanism, further reactions performed in an equimolar ratio of the Group 2 and mercury reagents provided similar observations but gave rise to the formation of an alternative β‐diketiminato calcium derivative (**10**). Although an *ipso* Ca−*C*_6_H_5_ environment could again be readily identified at *δ*=178.0 ppm in its ^13^C{^1^H} NMR spectrum, the corresponding ^1^H NMR experiment evidenced the complete disappearance of any observable calcium hydride signals.

**Scheme 2:**
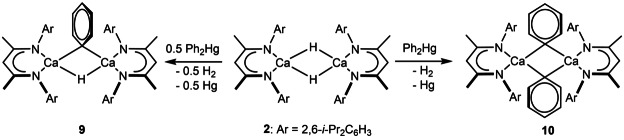
Synthetic routes to compounds **9** and **10**.

The origins of these observations were resolved by X‐ray diffraction analysis of compounds **9** and **10**, single crystals of which were isolated from toluene and benzene solutions, respectively. Although both compounds display dinuclear structures featuring Ca−μ_2_‐C−Ca interactions (Figure 1), **9** also comprises a Ca−μ_2_‐H−Ca unit while the dimeric structure of **10** is solely propagated by the bridging phenyl substituents. The Ca−C bond in **9** [Ca1−C30 2.5838(19) Å] is marginally elongated in comparison to those observed in compound **10** [Ca1−C30 2.5402(12), Ca1−C30′ 2.5667(12) Å]. Consistent with the lower 4‐coordinate geometry of their constituent calcium centers, however, both these latter distances are shorter than the analogous measurements across the only previously reported neutral, but 5‐coordinate, species to feature twofold, Ca−μ_2_‐C−Ca bridging [**5**, 2.618(2), 2.571(2) Å].^[8e]^ The more significant contrast between the two β‐diketiminate derivatives is provided by the orientation of the phenyl rings. Whereas the phenyl group of **9** is effectively orthogonal to that defined by the Ca1−C30−Ca1′−H least squares plane (88.4°), the corresponding angle subtended with the plane defined by the Ca1−C30−Ca1′−C30′ unit in compound **10** is only 24.88°. This feature, which does not appear to be precedented in any previous σ‐aryl organometallic derivative, is accompanied by a close approach (ca. 2.387, 2.439 Å) between the *ortho*‐CH units of the phenyl substituents and the calcium atoms.

**Figure 1:**
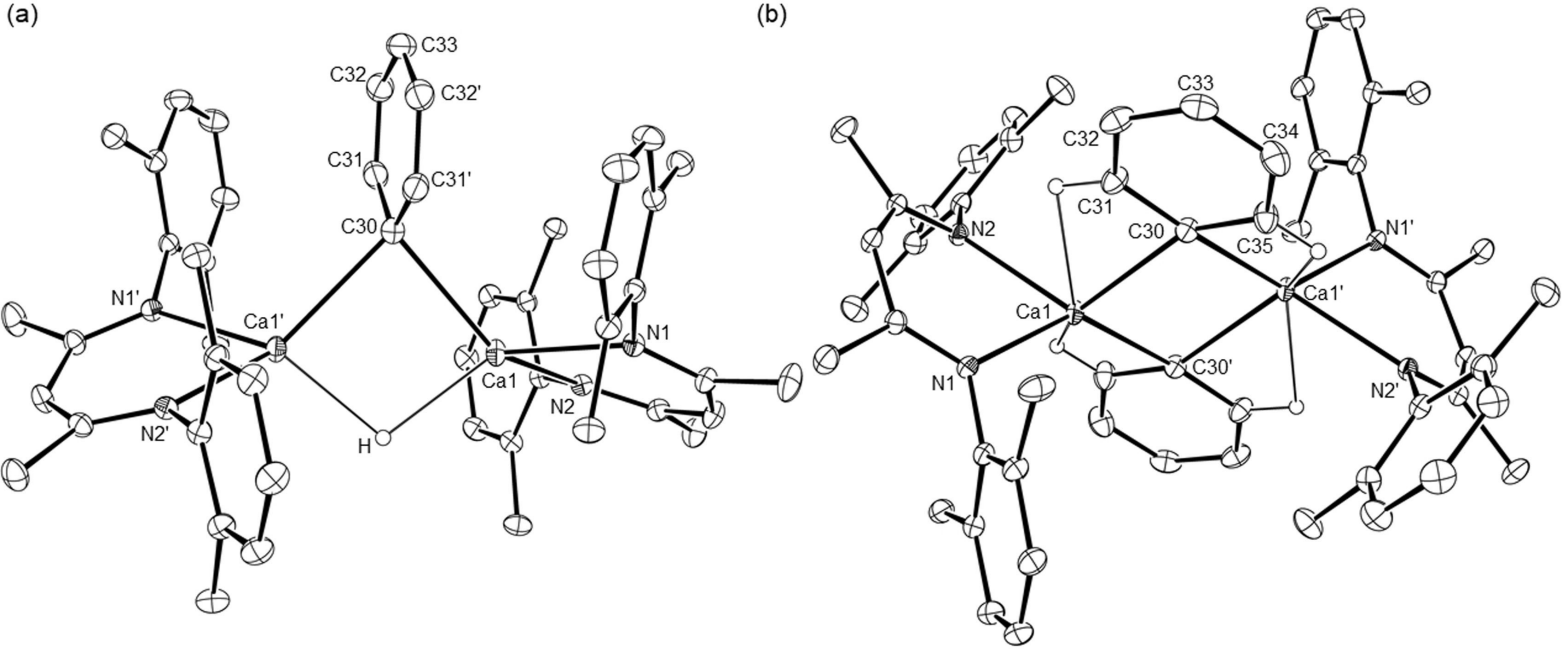
ORTEPs (30 % probability ellipsoids) of a) compound **9** and b) compound **10**. Hydrogen atoms, apart from the bridging hydride in **9** and the H31 and H35 atoms in **10**, Dipp methyl substituents, disordered atoms and occluded solvent are omitted for clarity. Selected bond lengths [Å] and angles [°]; **9**: Ca1−N1 2.3387(12), Ca1−N2 2.3602(13), Ca1−C30 2.5838(19), N1−Ca1−N2 78.17(4), N1−Ca1−C30 113.98(4), N2−Ca1−C30 138.37(4); **10**: Ca1−N1 2.3588(10), Ca1−N2 2.3610(10), Ca1−C30 2.5402(12), Ca1−C30′ 2.5667(12), N1−Ca1−N2 81.11(3), N1−Ca1−C30 106.35(4), N1−Ca1−C30′ 139.25(4), N2−Ca1−C30 116.11(4), N2−Ca1−C30′ 118.13(4), C30−Ca1−C30′ 96.78(4). Symmetry operations to generate primed atoms, **9**: 1−*x*, +*y*, 1/2−*z*; **10**: 1−*x*, 1−*y*, 1−*z*.

In order to shed light on the contrasting orientation of the phenyl rings we optimized the structures of compounds **9** and **10** by density functional theory (DFT) at the B3PW91‐D3 level of theory in the gas phase (**9_opt** and **10_opt**). As shown in Figures S76, S77 and Tables S2 and S3, the computed bond lengths and angles accurately reproduce the experimental values. In accordance with the experimental results, compound **10_opt** also displays short distances between the *ortho*‐CH units of the phenyl substituents and the calcium atoms (in the 2.427–2.469 Å range). To establish the relationship between the orientation of the phenyl rings and the energy of the complexes, we optimized complex **9** starting from a structure with the phenyl substituent almost coplanar to the plane defined by the Ca1−C30−Ca1′−H unit (**9′_opt**). Significantly, **9′_opt** displays a phenyl group oriented at an angle of ca. 30° with respect to the plane defined by the Ca1−C30−Ca1′−H unit and is 4.6 (3.2) kcal mol^−1^ less stable than the **9_opt** analogue containing an orthogonal phenyl group. The smaller angle between the phenyl plane and the Ca1−C30−Ca1′−H plane is likely to increase the steric hindrance between the phenyl moiety and the substituents of the ^Dipp^BDI ligands, causing an elongation of the Ca−N distances (in the 2.344–2.368 Å range for **9′_opt** compared to 2.334–2.355 Å for **9_opt**) and a consequent destabilization of the structure. Following the same approach, we optimized complex **10** starting from a structure with both the phenyl substituents orthogonal to the plane defined by the Ca1−C30−Ca1′−C30′ unit (**10′_opt**). The resulting **10′_opt** displays one phenyl group orthogonal to the Ca1−C30−Ca1′−C30′ plane, while the other is oriented at an angle of ca. 30° with respect to the same plane. Although **10′_opt** is more stable by 4.0 (5.8) kcal mol^−1^, in contrast to its **10_opt** analogue and the crystallized compound **10**, one of the two phenyl ligands remains in an orthogonal orientation. These adjustments again result in a reduction of the steric interactions between the phenyl groups and the BDI substituent and a contraction of the Ca−N bonds (in the 2.344–2.365 Å range for **10′_opt** compared to 2.373–2.387 Å for **10_opt**). The observed orientation of the phenyl ligands may, therefore, be judged to be dictated by a balance between the stabilizing interaction of the *ortho*‐CH units of the phenyl substituents with the coordinatively unsaturated calcium atoms and the steric hindrance between the phenyl groups and the ^Dipp^BDI substituents.

With compounds **9** and **10** in hand, we assessed their thermal stability in C_6_D_6_ solution by ^1^H NMR spectroscopy. In contrast to previous observations of alkylcalcium species derived from compound **2** (Scheme 1a),^[5a]^ neither compound provided any observable evidence for solvent‐derived C−H reactivity either under ambient temperature conditions or at 60 °C. Rather, any heating of the samples induced redistribution to the known homoleptic species, [(^Dipp^BDI)_2_Ca],^[12]^ along with decomposition to a variety of unidentifiable products. Although compound **2** is itself known to undergo slow C−D/Ca−H exchange with C_6_D_6_,^[13]^ these observations appear to discount biaryl formation by reaction of compounds **9** or **10** and the benzene solvent. Previous observations have shown that compound **2** can induce the hydrodehalogenation of C_6_H_5_X (X=I, Br, Cl) to provide benzene and the respective dimeric calcium hydride/halides and dihalides.^[14]^ In the case of the bromide‐based process, assessment by DFT calculations implied that the reactions take place in a stepwise fashion with the retention of the dimeric calcium structures and via S_N_Ar‐type displacement of the halide. While compound **4** has been reported as completely unreactive toward PhX (X=Cl, I), Westerhausen has previously demonstrated that biaryl formation can be achieved by reaction of such arylcalcium iodides with haloarenes under nickel‐catalyzed (Kumada‐type) conditions.^[8m]^ Although moderate (50–70 %) conversions of the haloarene reagents were achieved, this protocol was poorly specific, providing a mixture of the relevant homocoupled as well as the desired cross‐coupled biaryl products.

With these observations in mind, therefore, we undertook a study of the reactivity of compound **10** with a range of aryl bromides (Scheme 3). Initial reactions were performed at 25 °C in C_6_D_6_ to limit potential Schlenk‐type equilibration and were monitored by ^1^H and ^13^C{^1^H} NMR spectroscopy. In each case studied, this protocol provided slow (>100 hours) conversion to a single new β‐diketiminato calcium species (**11**) and the relevant cross‐coupled biaryl product, the identification of which was confirmed in situ by comparison of its NMR spectra to that of an authenticated sample and subsequent analysis by GC‐mass spectrometry (see the Supporting Information). Identical reactions performed at 60 °C provided similarly selective conversion to the biaryl products but were accompanied by redistribution of the heteroleptic calcium species to [(^Dipp^BDI)_2_Ca].^[12]^ At 25 °C, however, production of compound **11** was highly selective and characterized by the emergence of a single new ^Dipp^BDI γ‐methine signal at *δ*=4.77 ppm.

**Scheme 3:**
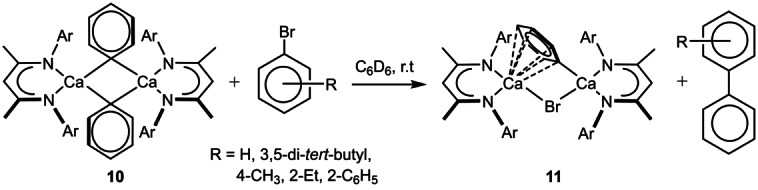
Biaryl formation by the direct reaction of compound **10** and bromoarenes and the synthesis of compound **11**.

Bulk samples of this new arylcalcium species (**11**) proved unstable, such that removal of volatiles also invariably led to the isolation of significant quantities of [(^Dipp^BDI)_2_Ca]. The structure of **11** was confirmed, however, by an X‐ray diffraction analysis performed on crystals isolated directly from the reaction mixture resulting from treatment of compound **10** with bromobenzene (Figure 2). Biaryl formation evidently occurs through the reaction of a single phenyl residue of compound **10**, as **11** is a further dinuclear calcium phenyl species in which dimer propagation is augmented by a Ca−μ‐Br−Ca bridging interaction via the bromide anion introduced by reaction with bromobenzene. In contrast to the Ca−μ_2_‐C−Ca interactions observed between the bridging organic anion and the two calcium centers in both compounds **9** and **10**, the remaining phenyl anion of **11** adopts a highly unsymmetrical disposition with respect to the Group 2 cations. Arene‐to‐calcium binding has been quite commonly observed in molecules in which the π‐donor substituent is a component of a more complex ligand.^[15]^ Compound **11**, however, appears to provide the first such example where the polyhapto‐bound unit is itself a σ‐bonded calcium organometallic. Whereas the bond length between the *ipso*‐phenyl carbon and Ca2 [Ca2−C33 2.5311(16) Å] is comparable to the analogous measurements observed in compounds **9** and **10**, the Ca1−C33 distance [2.8047(16) Å] is elongated by the adoption of an alternative η^6^‐interaction with the Ca1 center. While unsymmetrical, the range of Ca1−C_phenyl_ distances thus imposed [2.8047(16)–2.9093(17) Å] are all significantly shorter than those arising in the cationic components of [(^Dipp^BDI)Ca(C_6_H_6_)]^+^[A]^−^ [A=Al{OC(CF_3_)_3_}_4_ or B(C_6_F_5_)_4_] (ca. 2.93 Å) in which a neutral molecule of benzene solvent is bound to cationic calcium centers that are coordinated by an identical β‐diketiminate ligand.^[16]^

**Figure 2:**
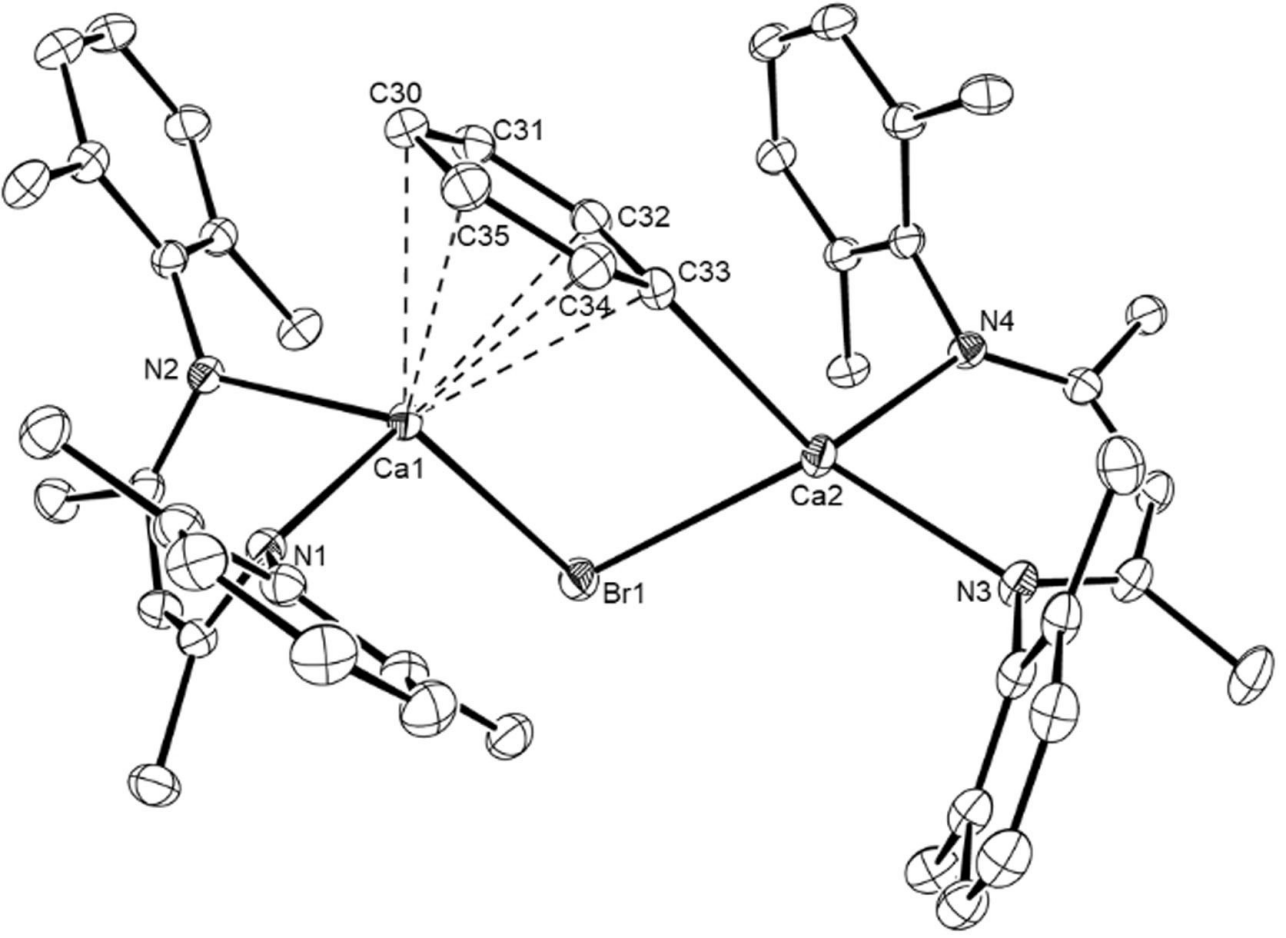
ORTEP (30 % probability ellipsoids) of compound **11**. Hydrogen atoms and Dipp methyl substituents are omitted for clarity. Selected bond lengths [Å] and angles [°]; Br1−Ca1 2.8603(3), Br1−Ca2 2.8809(4), Ca1−C30 2.9093(17), Ca1−C31 2.8860(17), Ca1−C32 2.8119(16), Ca1−C33 2.8047(16), Ca1−C34 2.8280(17), Ca1−C35 2.8969(18), Ca2−C33 2.5311(16), Ca1−Br1−Ca2 93.390(10), Ca2−C33−Ca1 102.95(5).

Calculations were carried out at the DFT level (B3PW91‐D3) to provide insight into the formation of complex **11** from the reaction of bromobenzene with **10**. The reaction of **10** with chlorobenzene has also been computed and is presented in the Supporting Information (Figures S79 and S80) for completeness. Starting from the dicalcium diphenyl complex, after the formation of a bromobenzene adduct (**B_Br_**, −10.0 kcal mol^−1^), a S_N_Ar‐type transition state (**TS‐BC_Br_**) has been located (Figure 3). Consistent with the relatively slow reaction observed at room temperature, the associated barrier is 33.8 kcal mol^−1^. Following the intrinsic reaction coordinate yields the formation of a very stable biphenyl solvated derivative of complex **11** (**C_B_**_r_), whose formation is exothermic by 76.2 kcal mol^−1^. The latter reaction energy indicates that the experimental timescale for the completion of the reaction is a direct consequence of the height of this barrier. The subsequent Ph/Br substitution via a second S_N_Ar‐type transition state has been also computed (see Figure S78). Although the reaction would be viable from both a kinetic and thermodynamic point of view, the experimentally observed decomposition of complex **11** in solution via Schlenk‐type equilibration presumably prevents the reaction from continuing toward the previously reported bromide derivative, [(BDI)CaBr]_2_.^[14]^

**Figure 3:**
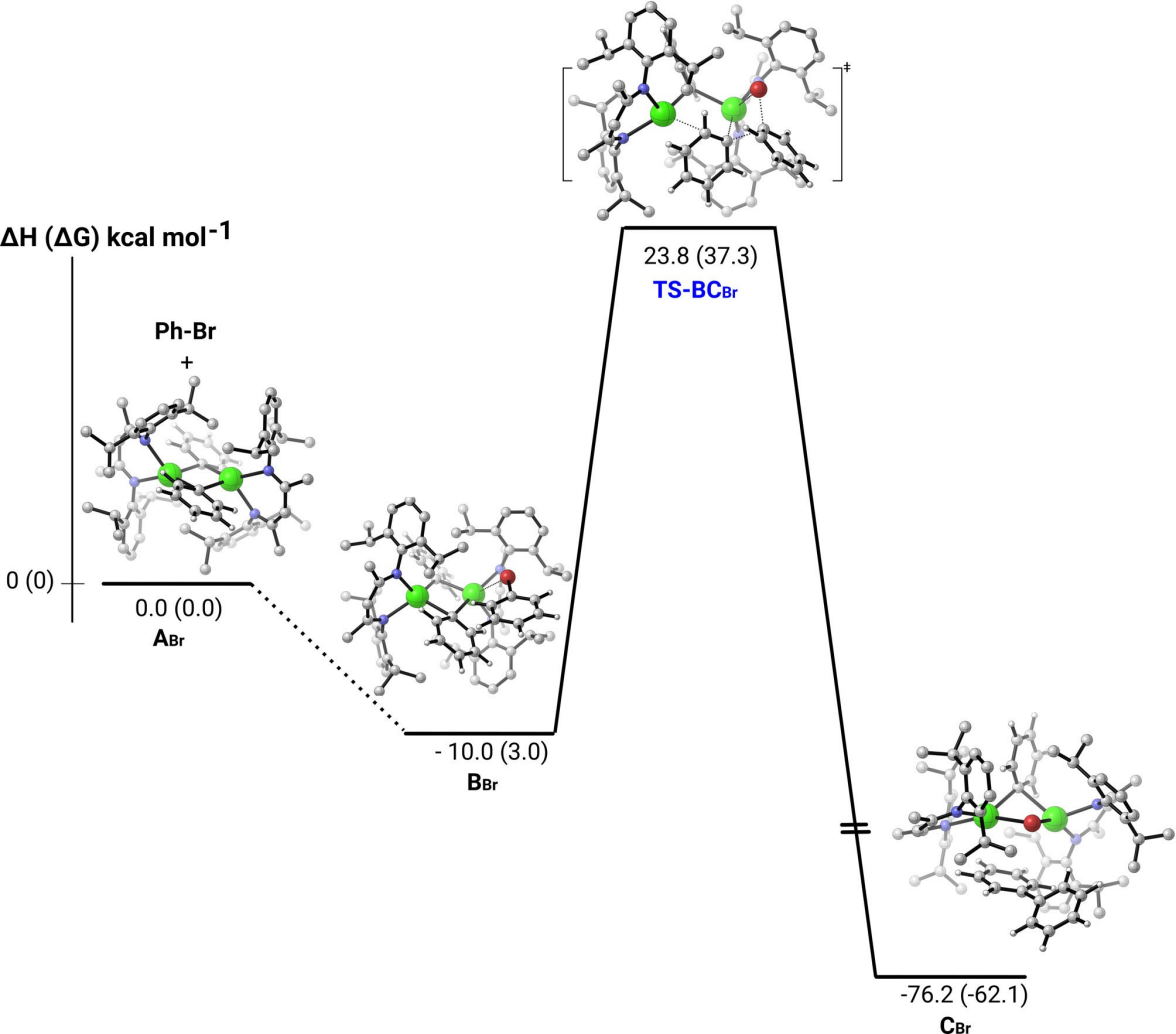
Computed enthalpy (Gibbs free energy) profile for the reaction of complex **A_Br_** (**10_opt**) with bromo‐benzene at room temperature.

In conclusion, we have shown that isolable and hydrocarbon‐soluble phenylcalcium derivatives are accessible from reactions of Ph_2_Hg and [(BDI)CaH]_2_. Although these compounds are inert toward the C−H bonds of benzene, they yield selective and uncatalyzed biaryl formation when reacted with readily available aryl bromides. We are continuing to extend this methodology to study the structures and reactivity of a wider range of heavier alkaline earth aryl derivatives.

**Supporting Information**: Experimental details, NMR spectra, X‐ray crystallography,^[17]^ and computational details and atomic coordinates for the optimized geometries of the compounds.

## Conflict of interest

The authors declare no conflict of interest.

## Supporting information

As a service to our authors and readers, this journal provides supporting information supplied by the authors. Such materials are peer reviewed and may be re‐organized for online delivery, but are not copy‐edited or typeset. Technical support issues arising from supporting information (other than missing files) should be addressed to the authors.

Supporting Information

Supporting Information

Supporting Information

## Data Availability

The data that support the findings of this study are available in the supplementary material of this article.
