# Supplementary material for: Synthesis of Molecular Phenylcalcium Derivatives: Application to the Formation of Biaryls
Source: Angew Chem Int Ed Engl. 2022 Mar 7;61(18):e202200305. doi: 10.1002/anie.202200305 (PMC9315018; doi:10.1002/anie.202200305)

checkCIF/PLATON report

You have not supplied any structure factors. As a result the full set of tests cannot be run.

THIS REPORT IS FOR GUIDANCE ONLY. IF USED AS PART OF A REVIEW PROCEDURE FOR PUBLICATION, IT SHOULD NOT REPLACE THE EXPERTISE OF AN EXPERIENCED CRYSTALLOGRAPHIC REFEREE.

No syntax errors found. CIF dictionary Interpreting this report

Datablock: s19msh74-cpd8

Bond precision: C-C = 0.0031 A Wavelength=1.54184

Cell:	a=16.3709(1)	b=38.7383(2)	c=13.7343(1)
	alpha=90	beta=112.363(1)	gamma=90
Temperature:	150 K		

	Calculated	Reported
Volume	8054.97(10)	8054.96(10)
Space group	P 21/c	P 1 21/c 1
Hall group	-P 2ybc	-P 2ybc
Moiety formula	C76 H105 Ca2 Cu N4, 2(C7 H8)	C76 H105 Ca2 Cu N4, 2(C7 H8)
Sum formula	C90 H121 Ca2 Cu N4	C90 H121 Ca2 Cu N4
Mr	1402.62	1402.60
Dx,g cm-3	1.157	1.157
Z	4	4
Mu (mm-1)	1.834	1.834
F000	3032.0	3032.0
F000'	3033.66	
h,k,lmax	20,48,17	20,47,17
Nref	16129	16057
Tmin,Tmax	0.729,0.822	0.807,1.000
Tmin'	0.549	

Correction method= # Reported T Limits: Tmin=0.807 Tmax=1.000
AbsCorr = MULTI-SCAN

Data completeness= 0.996 Theta(max)= 73.152

R(reflections)= 0.0407(14896) wR2(reflections)= 0.1064(16057)

S = 1.013 Npar= 990

The following ALERTS were generated. Each ALERT has the format
test-name_ALERT_alert-type_alert-level.
Click on the hyperlinks for more details of the test.

Alert level B

PLAT230_ALERT_2_B Hirshfeld Test Diff for C44 --C45 . 11.5 s.u.

Alert level C

PLAT220_ALERT_2_C NonSolvent Resd 1 C Ueq(max)/Ueq(min) Range 3.8 Ratio
PLAT222_ALERT_3_C NonSolvent Resd 1 H Uiso(max)/Uiso(min) Range 4.5 Ratio
PLAT230_ALERT_2_C Hirshfeld Test Diff for C44 --C46 . 6.0 s.u.
PLAT242_ALERT_2_C Low 'MainMol' Ueq as Compared to Neighbors of C44 Check
PLAT242_ALERT_2_C Low 'MainMol' Ueq as Compared to Neighbors of C49 Check
PLAT244_ALERT_4_C Low 'Solvent' Ueq as Compared to Neighbors of C78 Check
PLAT250_ALERT_2_C Large U3/U1 Ratio for Average U(i,j) Tensor .... 3.1 Note

Alert level G

PLAT002_ALERT_2_G Number of Distance or Angle Restraints on AtSite 18 Note
PLAT003_ALERT_2_G Number of Uiso or Uij Restrained non-H Atoms ... 10 Report
PLAT012_ALERT_1_G No _shelx_res_checksum Found in CIF ...... Please Check
PLAT083_ALERT_2_G SHELXL Second Parameter in WGHT Unusually Large 5.48 Why ?
PLAT142_ALERT_4_G s.u. on b - Axis Small or Missing .... 0.00020 Ang.
PLAT143_ALERT_4_G s.u. on c - Axis Small or Missing .... 0.00010 Ang.
PLAT176_ALERT_4_G The CIF-Embedded .res File Contains SADI Records 18 Report
PLAT178_ALERT_4_G The CIF-Embedded .res File Contains SIMU Records 2 Report
PLAT230_ALERT_2_G Hirshfeld Test Diff for C20 --C21 . 5.6 s.u.
PLAT231_ALERT_4_G Hirshfeld Test (Solvent) C81 --C82 . 10.7 s.u.
PLAT231_ALERT_4_G Hirshfeld Test (Solvent) C82 --C83 . 8.5 s.u.
PLAT300_ALERT_4_G Atom Site Occupancy of C21 Constrained at 0.4 Check
PLAT300_ALERT_4_G Atom Site Occupancy of C21A Constrained at 0.4 Check
PLAT300_ALERT_4_G Atom Site Occupancy of C21B Constrained at 0.2 Check
PLAT300_ALERT_4_G Atom Site Occupancy of H20 Constrained at 0.4 Check
PLAT300_ALERT_4_G Atom Site Occupancy of H20A Constrained at 0.4 Check
PLAT300_ALERT_4_G Atom Site Occupancy of H20B Constrained at 0.2 Check
PLAT300_ALERT_4_G Atom Site Occupancy of H21A Constrained at 0.4 Check
PLAT300_ALERT_4_G Atom Site Occupancy of H21B Constrained at 0.4 Check
PLAT300_ALERT_4_G Atom Site Occupancy of H21C Constrained at 0.4 Check
PLAT300_ALERT_4_G Atom Site Occupancy of H21D Constrained at 0.4 Check
PLAT300_ALERT_4_G Atom Site Occupancy of H21E Constrained at 0.4 Check
PLAT300_ALERT_4_G Atom Site Occupancy of H21F Constrained at 0.4 Check
PLAT300_ALERT_4_G Atom Site Occupancy of H21G Constrained at 0.2 Check
PLAT300_ALERT_4_G Atom Site Occupancy of H21H Constrained at 0.2 Check
PLAT300_ALERT_4_G Atom Site Occupancy of H21I Constrained at 0.2 Check
PLAT300_ALERT_4_G Atom Site Occupancy of C84 Constrained at 0.87 Check
PLAT300_ALERT_4_G Atom Site Occupancy of C85 Constrained at 0.87 Check
PLAT300_ALERT_4_G Atom Site Occupancy of C86 Constrained at 0.87 Check
PLAT300_ALERT_4_G Atom Site Occupancy of C87 Constrained at 0.87 Check
PLAT300_ALERT_4_G Atom Site Occupancy of C88 Constrained at 0.87 Check
PLAT300_ALERT_4_G Atom Site Occupancy of C89 Constrained at 0.87 Check
PLAT300_ALERT_4_G Atom Site Occupancy of C90 Constrained at 0.87 Check
PLAT300_ALERT_4_G Atom Site Occupancy of H84A Constrained at 0.87 Check
PLAT300_ALERT_4_G Atom Site Occupancy of H84B Constrained at 0.87 Check

```
PLAT300_ALERT_4_G Atom Site Occupancy of H84C       Constrained at       0.87 Check
PLAT300_ALERT_4_G Atom Site Occupancy of H86        Constrained at       0.87 Check
PLAT300_ALERT_4_G Atom Site Occupancy of H87        Constrained at       0.87 Check
PLAT300_ALERT_4_G Atom Site Occupancy of H88        Constrained at       0.87 Check
PLAT300_ALERT_4_G Atom Site Occupancy of H89        Constrained at       0.87 Check
PLAT300_ALERT_4_G Atom Site Occupancy of H90        Constrained at       0.87 Check
PLAT300_ALERT_4_G Atom Site Occupancy of C84A       Constrained at       0.13 Check
PLAT300_ALERT_4_G Atom Site Occupancy of C85A       Constrained at       0.13 Check
PLAT300_ALERT_4_G Atom Site Occupancy of C86A       Constrained at       0.13 Check
PLAT300_ALERT_4_G Atom Site Occupancy of C87A       Constrained at       0.13 Check
PLAT300_ALERT_4_G Atom Site Occupancy of C88A       Constrained at       0.13 Check
PLAT300_ALERT_4_G Atom Site Occupancy of C89A       Constrained at       0.13 Check
PLAT300_ALERT_4_G Atom Site Occupancy of C90A       Constrained at       0.13 Check
PLAT300_ALERT_4_G Atom Site Occupancy of H84D       Constrained at       0.13 Check
PLAT300_ALERT_4_G Atom Site Occupancy of H84E       Constrained at       0.13 Check
PLAT300_ALERT_4_G Atom Site Occupancy of H84F       Constrained at       0.13 Check
PLAT300_ALERT_4_G Atom Site Occupancy of H86A       Constrained at       0.13 Check
PLAT300_ALERT_4_G Atom Site Occupancy of H87A       Constrained at       0.13 Check
PLAT300_ALERT_4_G Atom Site Occupancy of H88A       Constrained at       0.13 Check
PLAT300_ALERT_4_G Atom Site Occupancy of H89A       Constrained at       0.13 Check
PLAT300_ALERT_4_G Atom Site Occupancy of H90A       Constrained at       0.13 Check
PLAT301_ALERT_3_G Main Residue  Disorder .............(Resd  1  )          1% Note
PLAT302_ALERT_4_G Anion/Solvent/Minor-Residue Disorder (Resd  3  )       100% Note
PLAT302_ALERT_4_G Anion/Solvent/Minor-Residue Disorder (Resd  4  )       100% Note
PLAT303_ALERT_2_G Full Occupancy Atom H1        with # Connections       3.00 Check
PLAT304_ALERT_4_G Non-Integer Number of Atoms in ..... (Resd  3  )      13.05 Check
PLAT304_ALERT_4_G Non-Integer Number of Atoms in ..... (Resd  4  )       1.95 Check
PLAT343_ALERT_2_G Unusual sp?      Angle Range in Main Residue for        C59 Check
PLAT343_ALERT_2_G Unusual sp?      Angle Range in Main Residue for        C68 Check
PLAT412_ALERT_2_G Short Intra XH3 .. XHn     H5C      ..H21D     .       1.91 Ang.
                                                      x,y,z  =      1_555 Check
PLAT412_ALERT_2_G Short Intra XH3 .. XHn     H22A     ..H21C     .       2.00 Ang.
                                                      x,y,z  =      1_555 Check
PLAT412_ALERT_2_G Short Intra XH3 .. XHn     H22C     ..H20B     .       1.95 Ang.
                                                      x,y,z  =      1_555 Check
PLAT794_ALERT_5_G Tentative Bond Valency for Cu1       (I)       .       0.44 Info
PLAT860_ALERT_3_G Number of Least-Squares Restraints ............          79 Note
```

---

0 **ALERT level A** = Most likely a serious problem - resolve or explain
1 **ALERT level B** = A potentially serious problem, consider carefully
7 **ALERT level C** = Check. Ensure it is not caused by an omission or oversight
69 **ALERT level G** = General information/check it is not something unexpected

1 ALERT type 1 CIF construction/syntax error, inconsistent or missing data
16 ALERT type 2 Indicator that the structure model may be wrong or deficient
3 ALERT type 3 Indicator that the structure quality may be low
56 ALERT type 4 Improvement, methodology, query or suggestion
1 ALERT type 5 Informative message, check

Datablock: s21msh31-cpd9

Bond precision: C-C = 0.0026 A   Wavelength=1.54184

Cell:	a=23.2964(4)	b=11.45398(16)	c=27.5622(4)
	alpha=90	beta=107.5826(16)	gamma=90
Temperature:	150 K		

	Calculated	Reported
Volume	7011.0(2)	7011.03(19)
Space group	C 2/c	C 1 2/c 1
Hall group	-C 2yc	-C 2yc
Moiety formula	C64 H88 Ca2 N4, 2(C7 H8)	C64 H88 Ca2 N4, 2(C7 H8)
Sum formula	C78 H104 Ca2 N4	C78 H104 Ca2 N4
Mr	1177.81	1177.81
Dx,g cm-3	1.116	1.116
Z	4	4
Mu (mm-1)	1.733	1.733
F000	2560.0	2560.0
F000'	2568.74	
h,k,lmax	28,14,34	28,14,34
Nref	7066	6944
Tmin,Tmax	0.571,0.825	0.615,1.000
Tmin'	0.517	

Correction method= # Reported T Limits: Tmin=0.615 Tmax=1.000
AbsCorr = MULTI-SCAN

Data completeness= 0.983 Theta(max)= 73.541

R(reflections)= 0.0459(6260) wR2(reflections)= 0.1264(6944)

S = 1.052 Npar= 413

The following ALERTS were generated. Each ALERT has the format
test-name_ALERT_alert-type_alert-level.
Click on the hyperlinks for more details of the test.

Alert level G

```
PLAT002_ALERT_2_G Number of Distance or Angle Restraints on AtSite          5 Note
PLAT003_ALERT_2_G Number of Uiso or Uij Restrained non-H Atoms ...          1 Report
PLAT012_ALERT_1_G No       _shelx_res_checksum Found in CIF ......     Please Check
PLAT083_ALERT_2_G SHELXL Second Parameter in WGHT  Unusually Large       5.10 Why ?
PLAT176_ALERT_4_G The CIF-Embedded .res File Contains SADI Records          2 Report
PLAT178_ALERT_4_G The CIF-Embedded .res File Contains SIMU Records          1 Report
PLAT300_ALERT_4_G Atom Site Occupancy of C16         Constrained at       0.6 Check
PLAT300_ALERT_4_G Atom Site Occupancy of C17         Constrained at       0.6 Check
PLAT300_ALERT_4_G Atom Site Occupancy of C16A        Constrained at       0.4 Check
PLAT300_ALERT_4_G Atom Site Occupancy of C17A        Constrained at       0.4 Check
PLAT300_ALERT_4_G Atom Site Occupancy of H15         Constrained at       0.6 Check
PLAT300_ALERT_4_G Atom Site Occupancy of H16A        Constrained at       0.6 Check
```

```
PLAT300_ALERT_4_G Atom Site Occupancy of H16B       Constrained at        0.6 Check
PLAT300_ALERT_4_G Atom Site Occupancy of H16C       Constrained at        0.6 Check
PLAT300_ALERT_4_G Atom Site Occupancy of H17A       Constrained at        0.6 Check
PLAT300_ALERT_4_G Atom Site Occupancy of H17B       Constrained at        0.6 Check
PLAT300_ALERT_4_G Atom Site Occupancy of H17C       Constrained at        0.6 Check
PLAT300_ALERT_4_G Atom Site Occupancy of H15A       Constrained at        0.4 Check
PLAT300_ALERT_4_G Atom Site Occupancy of H16D       Constrained at        0.4 Check
PLAT300_ALERT_4_G Atom Site Occupancy of H16E       Constrained at        0.4 Check
PLAT300_ALERT_4_G Atom Site Occupancy of H16F       Constrained at        0.4 Check
PLAT300_ALERT_4_G Atom Site Occupancy of H17D       Constrained at        0.4 Check
PLAT300_ALERT_4_G Atom Site Occupancy of H17E       Constrained at        0.4 Check
PLAT300_ALERT_4_G Atom Site Occupancy of H17F       Constrained at        0.4 Check
PLAT301_ALERT_3_G Main Residue  Disorder ..............(Resd  1  )          6% Note
PLAT303_ALERT_2_G Full Occupancy Atom H         with # Connections       2.00 Check
PLAT412_ALERT_2_G Short Intra XH3 .. XHn     H1A      ..H17F     .       2.13 Ang.
                                                        x,y,z  =      1_555 Check
PLAT860_ALERT_3_G Number of Least-Squares Restraints .............          2 Note
PLAT941_ALERT_3_G Average HKL Measurement Multiplicity ...........        4.3 Low
```

```
   0 ALERT level A = Most likely a serious problem - resolve or explain
   0 ALERT level B = A potentially serious problem, consider carefully
   0 ALERT level C = Check. Ensure it is not caused by an omission or oversight
  29 ALERT level G = General information/check it is not something unexpected

   1 ALERT type 1 CIF construction/syntax error, inconsistent or missing data
   5 ALERT type 2 Indicator that the structure model may be wrong or deficient
   3 ALERT type 3 Indicator that the structure quality may be low
  20 ALERT type 4 Improvement, methodology, query or suggestion
   0 ALERT type 5 Informative message, check
```

Datablock: s19msh123-cpd10

```
Bond precision:   C-C = 0.0019 A           Wavelength=1.54184

Cell:             a=11.8304(1)     b=15.1721(1)       c=17.8162(1)
                  alpha=90         beta=98.794(1)     gamma=90
Temperature:      150 K
```

	Calculated	Reported
Volume	3160.27(4)	3160.27(4)
Space group	P 21/n	P 1 21/n 1
Hall group	-P 2yn	-P 2yn
Moiety formula	C70 H92 Ca2 N4	C35 H46 Ca N2
Sum formula	C70 H92 Ca2 N4	C35 H46 Ca N2
Mr	1069.64	534.82
Dx,g cm-3	1.124	1.124
Z	2	4
Mu (mm-1)	1.876	1.876
F000	1160.0	1160.0
F000’	1164.09	
h,k,lmax	14,18,22	14,18,22
Nref	6322	6316
Tmin,Tmax	0.481,0.574	0.799,1.000
Tmin’	0.431	

Correction method= # Reported T Limits: Tmin=0.799 Tmax=1.000
AbsCorr = MULTI-SCAN

Data completeness= 0.999 Theta(max)= 73.092

R(reflections)= 0.0346(6081) wR2(reflections)= 0.0931(6316)

S = 1.019 Npar= 365

The following ALERTS were generated. Each ALERT has the format
test-name_ALERT_alert-type_alert-level.
Click on the hyperlinks for more details of the test.

Alert level G

```
PLAT012_ALERT_1_G No       _shelx_res_checksum Found in CIF ......     Please Check
PLAT042_ALERT_1_G Calc. and Reported Moiety Formula Strings Differ     Please Check
PLAT045_ALERT_1_G Calculated and Reported Z Differ by a Factor ...     0.5000 Check
PLAT142_ALERT_4_G s.u. on b - Axis Small or Missing .............    0.00010 Ang.
PLAT143_ALERT_4_G s.u. on c - Axis Small or Missing .............    0.00010 Ang.
PLAT153_ALERT_1_G The s.u.'s on the Cell Axes   are Equal ..(Note)     0.0001 Ang.
PLAT164_ALERT_4_G Nr. of Refined C-H H-Atoms in Heavy-Atom Struct.          1 Note
PLAT303_ALERT_2_G Full Occupancy Atom H31       with # Connections       2.00 Check
PLAT303_ALERT_2_G Full Occupancy Atom H35       with # Connections       2.00 Check
PLAT343_ALERT_2_G Unusual          Angle Range in Main Residue for        C30 Check
PLAT933_ALERT_2_G Number of HKL-OMIT Records in Embedded .res File          1 Note
```

---

0 **ALERT level A** = Most likely a serious problem - resolve or explain
0 **ALERT level B** = A potentially serious problem, consider carefully
0 **ALERT level C** = Check. Ensure it is not caused by an omission or oversight
11 **ALERT level G** = General information/check it is not something unexpected

```
   4 ALERT type 1 CIF construction/syntax error, inconsistent or missing data
   4 ALERT type 2 Indicator that the structure model may be wrong or deficient
   0 ALERT type 3 Indicator that the structure quality may be low
   3 ALERT type 4 Improvement, methodology, query or suggestion
   0 ALERT type 5 Informative message, check
```

Datablock: s21msh77-cpd11

Bond precision: C-C = 0.0025 A Wavelength=1.54184

Cell:	a=9.1351(2)	b=13.9397(3)	c=23.6217(5)
	alpha=93.187(2)	beta=98.481(2)	gamma=93.288(2)
Temperature:	150 K		

	Calculated	Reported
Volume	2963.96(11)	2963.95(11)
Space group	P -1	P -1
Hall group	-P 1	-P 1
Moiety formula	C64 H87 Br Ca2 N4	C64 H87 Br Ca2 N4
Sum formula	C64 H87 Br Ca2 N4	C64 H87 Br Ca2 N4
Mr	1072.44	1072.44
Dx,g cm-3	1.202	1.202
Z	2	2
Mu (mm-1)	2.760	2.760
F000	1148.0	1148.0
F000'	1150.54	
h,k,lmax	11,17,29	11,17,29
Nref	11965	11817
Tmin,Tmax	0.729,0.869	0.901,1.000
Tmin'	0.599	

Correction method= # Reported T Limits: Tmin=0.901 Tmax=1.000
AbsCorr = MULTI-SCAN

Data completeness= 0.988 Theta(max)= 73.511

R(reflections)= 0.0322(10441) wR2(reflections)= 0.0850(11817)

S = 1.020 Npar= 664

```
The following ALERTS were generated. Each ALERT has the format
       test-name_ALERT_alert-type_alert-level.
Click on the hyperlinks for more details of the test.
```

Alert level G

```
PLAT012_ALERT_1_G No       _shelx_res_checksum Found in CIF ......     Please Check
PLAT154_ALERT_1_G The s.u.'s on the Cell Angles are Equal ..(Note)      0.002 Degree
PLAT164_ALERT_4_G Nr. of Refined C-H H-Atoms in Heavy-Atom Struct.          1 Note
PLAT764_ALERT_4_G Overcomplete CIF Bond List Detected (Rep/Expd) .       1.14 Ratio
PLAT779_ALERT_4_G Suspect or Irrelevant (Bond) Angle(s) in CIF ...      41.88 Deg.
             N3   -C41  -CA2     1_555   1_555   1_555 ......     #  180 Check
PLAT779_ALERT_4_G Suspect or Irrelevant (Bond) Angle(s) in CIF ...      44.93 Deg.
             N4   -C53  -CA2     1_555   1_555   1_555 ......     #  201 Check
PLAT941_ALERT_3_G Average HKL Measurement Multiplicity ..........         2.2 Low
```

```
   0 ALERT level A = Most likely a serious problem - resolve or explain
   0 ALERT level B = A potentially serious problem, consider carefully
   0 ALERT level C = Check. Ensure it is not caused by an omission or oversight
   7 ALERT level G = General information/check it is not something unexpected

   2 ALERT type 1 CIF construction/syntax error, inconsistent or missing data
   0 ALERT type 2 Indicator that the structure model may be wrong or deficient
   1 ALERT type 3 Indicator that the structure quality may be low
   4 ALERT type 4 Improvement, methodology, query or suggestion
   0 ALERT type 5 Informative message, check
```

Validation response form

Please find below a validation response form (VRF) that can be filled in and pasted into your CIF.

```
# start Validation Reply Form
_vrf_PLAT230_s19msh74-cpd8
;
PROBLEM: Hirshfeld Test Diff for    C44      --C45      .       11.5 s.u.
RESPONSE: ...
;
# end Validation Reply Form
```

It is advisable to attempt to resolve as many as possible of the alerts in all categories. Often the minor alerts point to easily fixed oversights, errors and omissions in your CIF or refinement strategy, so attention to these fine details can be worthwhile. In order to resolve some of the more serious problems it may be necessary to carry out additional measurements or structure refinements. However, the purpose of your study may justify the reported deviations and the more serious of these should normally be commented upon in the discussion or experimental section of a paper or in the "special_details" fields of the CIF. checkCIF was carefully designed to identify outliers and unusual parameters, but every test has its limitations and alerts that are not important in a particular case may appear. Conversely, the absence of alerts does not guarantee there are no aspects of the results needing attention. It is up to the individual to critically assess their own results and, if necessary, seek expert advice.

Publication of your CIF in IUCr journals

A basic structural check has been run on your CIF. These basic checks will be run on all CIFs submitted for publication in IUCr journals (*Acta Crystallographica*, *Journal of Applied Crystallography*, *Journal of Synchrotron Radiation*); however, if you intend to submit to *Acta Crystallographica Section C* or *E* or *IUCrData*, you should make sure that full publication checks are run on the final version of your CIF prior to submission.

Publication of your CIF in other journals

Please refer to the *Notes for Authors* of the relevant journal for any special instructions relating to CIF submission.

PLATON version of 18/12/2021; check.def file version of 18/12/2021

Datablock s19msh74-cpd8 - ellipsoid plot

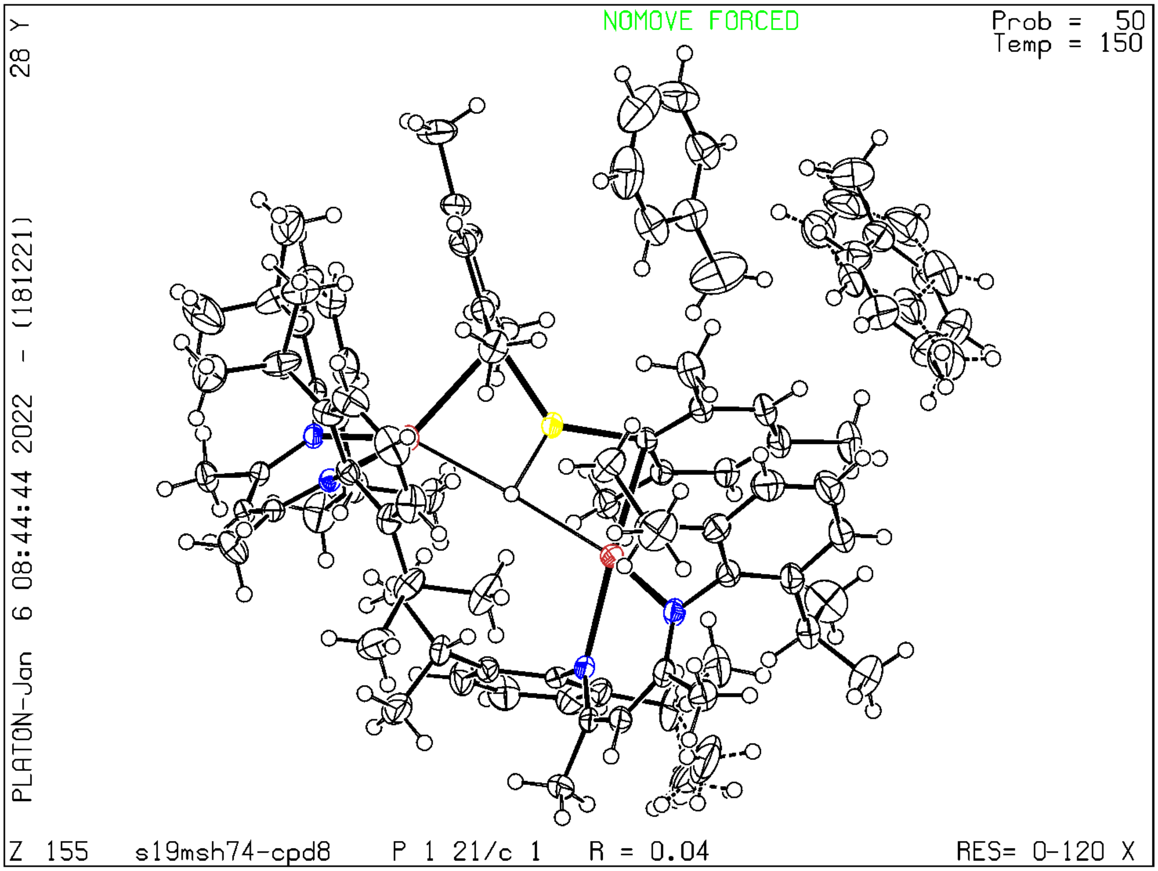

Datablock s21msh31-cpd9 - ellipsoid plot

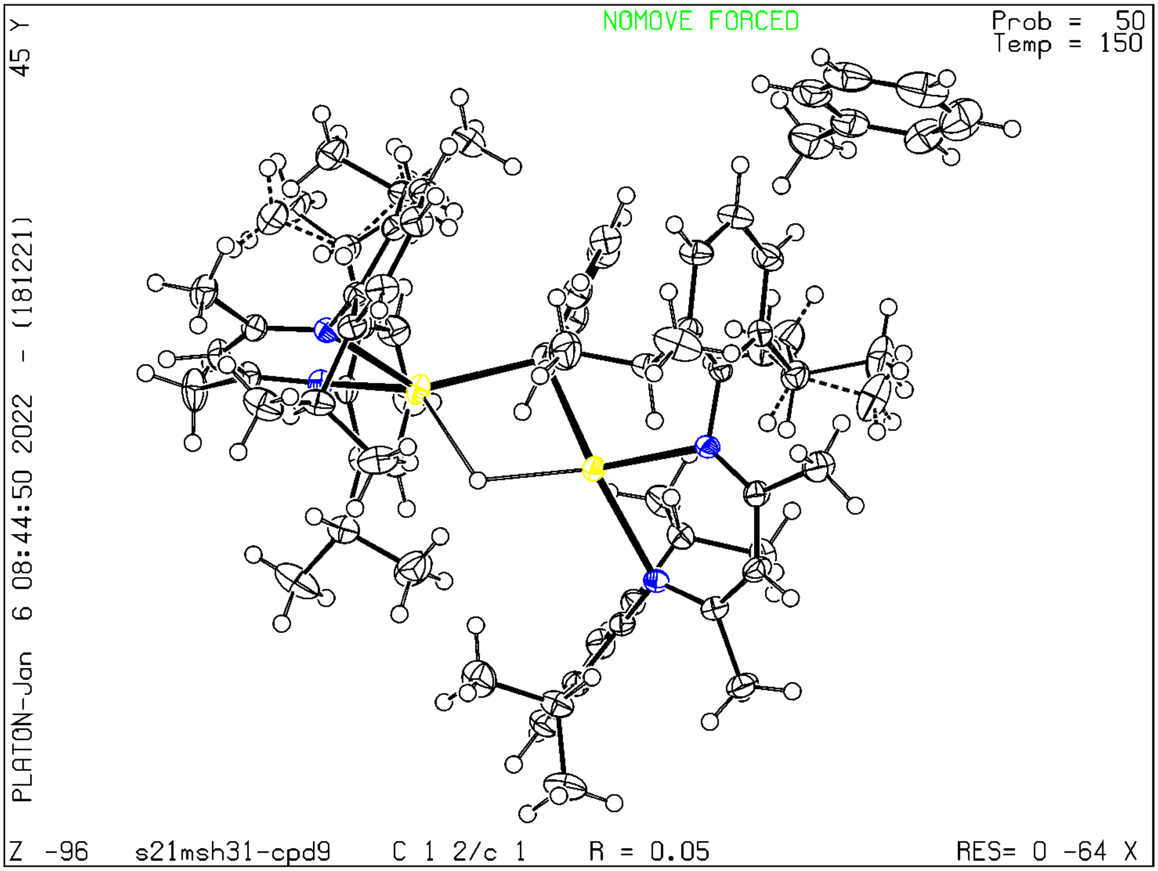

Datablock s19msh123-cpd10 - ellipsoid plot

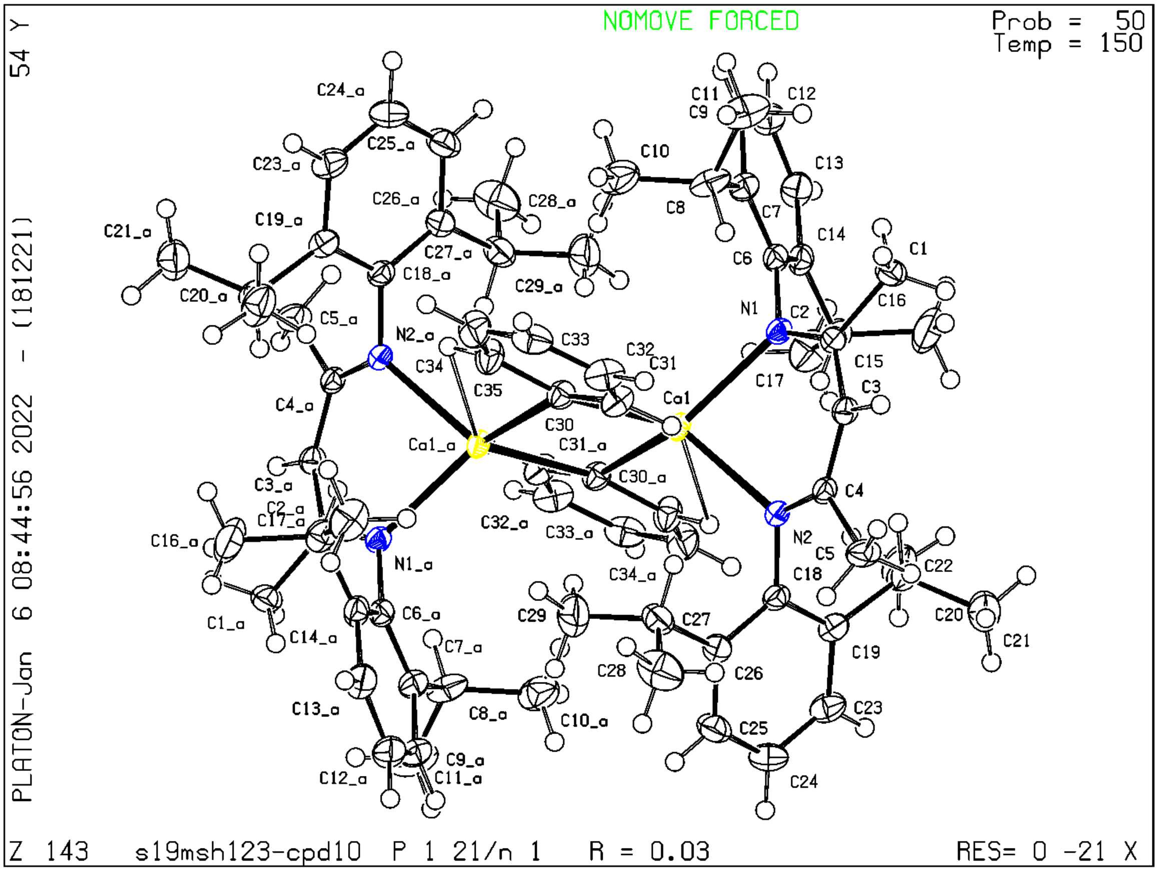

Datablock s21msh77-cpd11 - ellipsoid plot

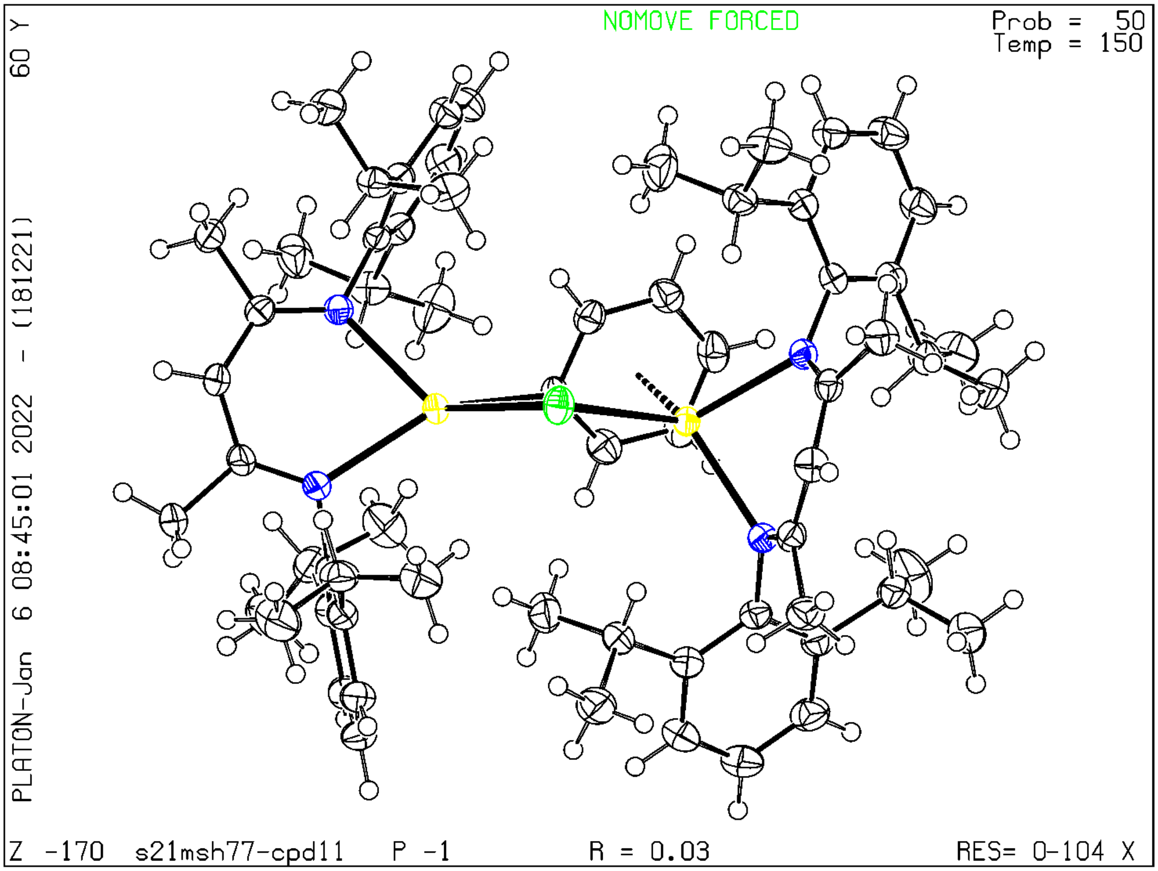

Supplement: Supplementary file 2 — Supporting Information [file ANIE-61-0-s001.pdf]
