# Supplementary material for: Synthesis of Molecular Phenylcalcium Derivatives: Application to the Formation of Biaryls
Source: Angew Chem Int Ed Engl. 2022 Mar 7;61(18):e202200305. doi: 10.1002/anie.202200305 (PMC9315018; doi:10.1002/anie.202200305)
Supplement: Supplementary file 3 — Supporting Information [file ANIE-61-0-s003.pdf]

Angewandte Chemie
Eine Zeitschrift der Gesellschaft Deutscher Chemiker

Supporting Information

Synthesis of Molecular Phenylcalcium Derivatives: Application to the Formation of Biaryls

K. G. Pearce, C. Dinoi, M. S. Hill, M. F. Mahon, L. Maron*, R. S. Schwamm, A. S. S. Wilson*

Synthetic Details

General Considerations

All manipulations were carried out using standard Schlenk line and glovebox techniques under an inert atmosphere of argon. NMR experiments were conducted in J-Young tap NMR tubes prepared in a glovebox. NMR spectra were recorded on a Bruker BioSpin GmbH spectrometer operating at 400.13 MHz (^{1}H) and 100.62 MHz (^{13}C). Elemental analyses were performed at Elemental Microanalysis Ltd., Okehampton, Devon, UK. GC-MS analysis were performed with a 8890 gas chromatography system coupled with 5977B MSD, used for the analysis. Split injections of 1 µL were performed, with a split ratio of 10:1 (split flow of 10 mL/min), using a split inlet liner, with tapered focus and deactivated glass wool (Agilent 210-4022-5). The inlet was heated to 280°C with 3 mL/min septum purge flow. An Agilent HP-5MS (30 m, 0.25mm, 0.25µm) column was used with He (BOC, N5.5) as the carrier gas, at a constant flow of 1.0 mL/min. The column oven gradient was started at 70°C, held for 4 min, then ramped at 15°C/min to 280°C, with a 5 min hold. The MSD transfer line was set at 280°C, MSD source at 230°C, and the MSD quad temperature was set to 150°C. After an initial solvent delay of 7 min the MSD detection was performed using full scan mode, over the range of 180 – 550 m/z, with a scan speed of 1562 µs, and a gain factor of 15. Data analysis was performed in the Agilent Qualitative Analysis v.10.0 and used the NIST 17 library to identify and confirm compounds through spectral matching. Solvents were dried by passage through a commercially available solvent purification system and stored under argon in ampoules over 4 Å molecular sieves. C_6D_6 was purchased from Sigma-Aldrich, dried over a potassium before distilling and storage over molecular sieves. Arylhalides were purchased from Merck, degassed, dried over calcium hydride then distilled under argon before use, 1-bromo-3,5-di-tert-butylbenzene was dried before use. [(BDI)CaH] (**2**) (BDI = HC{(Me)CN-2,6-i-$Pr_2C_6H_3$}$_2$) and Ph_2Hg were synthesised according to literature procedures.[1,2]

Synthesis of [(BDI)Ca(H)PhCa(BDI)] (9)

Toluene (2 cm^3) was added to a vial containing [HC{(Me)CN(2,6-^{i}Pr$_2$C$_6$H$_3$)}$_2$CaH]$_2$ (**2**) (30 mg, 0.03 mmol) and Ph_2Hg (5.8 mg, 0.016 mmol) resulting in effervescence. Once effervescence halted the grey suspension was filtered into a fresh vial and left to sit overnight (*ca.* 16 hours) in the freezer (-35 °C). After such time colourless crystals formed which were washed with cold (-35 °C) toluene (3x2 cm^3) and the residual solvent was allowed to evaporate. Yield: 25.4 mg, 77 % crystal yield. ^{1}H NMR (C_6D_6): δ = 7.13-6.96 (Ar-H and residual toluene), 6.89-6.86 (m, *p*-Ar-H, 1H), 6.69 (t, *m*-Ar-H, $^3J_{HH}$ = 7.2 Hz, 2H), 6.57-6.55 (m, *o*-Ar-H, 2H), 4.81 (s, Ca-H̲, 1H), 4.78 (s, NC(CH$_3$)CH̲, 2H), 2.97 (hept, CH̲(CH$_3$)$_2$, $^3J_{HH}$ = 6.9 Hz, 8H), 1.59 (s, NC(CH̲$_3$)CH, 12H), 1.11 (d, CH(CH̲$_3$)$_2$, $^3J_{HH}$ = 6.9 Hz, 24H), 0.93 (d, CH(CH̲$_3$)$_2$, $^3J_{HH}$ = 6.9 Hz, 24H). ^{13}C{^{1}H} NMR (C_6D_6) δ = 180.7 (C$_{ipso}$, C$_6$H$_5$), 165.8 (NC̲(CH$_3$)CH), 145.5 (Ar-C), 142.1 (Ar-C), 138.8 (*o*-Ar-C, C$_6$H$_5$), 129.0 (Ar-C), 124.9 (Ar-C), 124.4 (Ar-C), 94.9 (NC(CH$_3$)C̲H), 28.6 (CH(C̲H$_3$)$_2$), 24.8 (NC(CH̲$_3$)CH), 24.2 (CH(C̲H$_3$)$_2$), 24.1 (CH(C̲H$_3$)$_2$). Anal. Calc. for $Ca_2N_4C_{64}H_{88}$ -: C, 77.37; H, 8.93; N, 5.64. Found: C, 77.66; H, 8.48; N, 5.04.

Synthesis of $[(BDI)CaPh]_2$ (10)

Benzene (2 cm^3) was added to a vial containing $[HC\{(Me)CN(2,6\text{-}^{i}Pr_2C_6H_3)\}_2CaH]_2$ (**2**) (30 mg, 0.03 mmol) and Ph_2Hg (15 mg, 0.04 mmol) resulting in effervescence. Once effervescence halted the grey suspension was filtered into a fresh vial and left to sit overnight (*ca.* 16 hours). After such time colourless crystals formed which were washed with benzene (3x2 cm^3) and the residual solvent was allowed to evaporate. Yield: 12.1 mg, 35 % crystal yield. ^{1}H NMR (C_6D_6): δ = 7.43 (m, Ar-H, 4H), 7.14-6.98 (m, Ar-H, 18H), 4.86 (s, NC(CH$_3$)CH, 2H), 2.95 (hept, CH(CH$_3$)$_2$, $^3J_{HH}$ = 6.8 Hz, 8H), 1.65 (s, NC(CH$_3$)CH, 12H), 1.10 (d, CH(CH$_3$)$_2$, $^3J_{HH}$ = 6.8 Hz, 24H), 0.72 (d, CH(CH$_3$)$_2$, $^3J_{HH}$ = 6.8 Hz, 24H). ^{13}C{^{1}H} NMR (C_6D_6) δ = 178.0 (C_{ipso}, C_6H_5), 166.4 (NC(CH$_3$)CH), 146.3 (Ar-C), 141.6 (Ar-C), 137.0 (Ar-C), 128.9 (Ar-C), 124.4 (Ar-C), 123.6 (Ar-C), 93.9 (NC(CH$_3$)CH), 28.2 (CH(CH$_3$)$_2$), 24.6 (NC(CH$_3$)CH), 24.3 (CH(CH$_3$)$_2$), 24.0 (CH(CH$_3$)$_2$). Anal. Calc. for $Ca_2N_4C_{70}H_{92}$ -: 78.6; H, 8.67; N, 5.24. Found: C, 77.07; H, 8.51; N, 5.18.

NMR Spectra for (9) and (10).

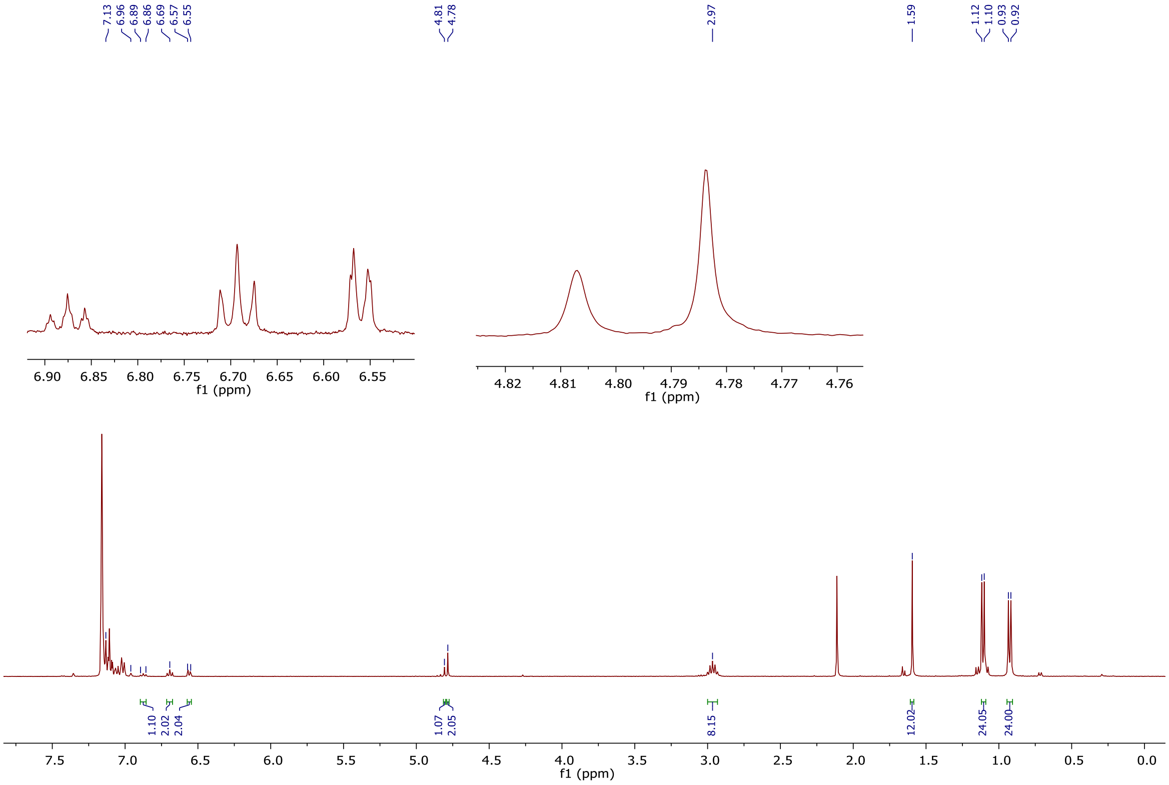

Figure S1. ^{1}H NMR Spectrum (C_6D_6, 298 K, 400.13 MHz) for **9**.

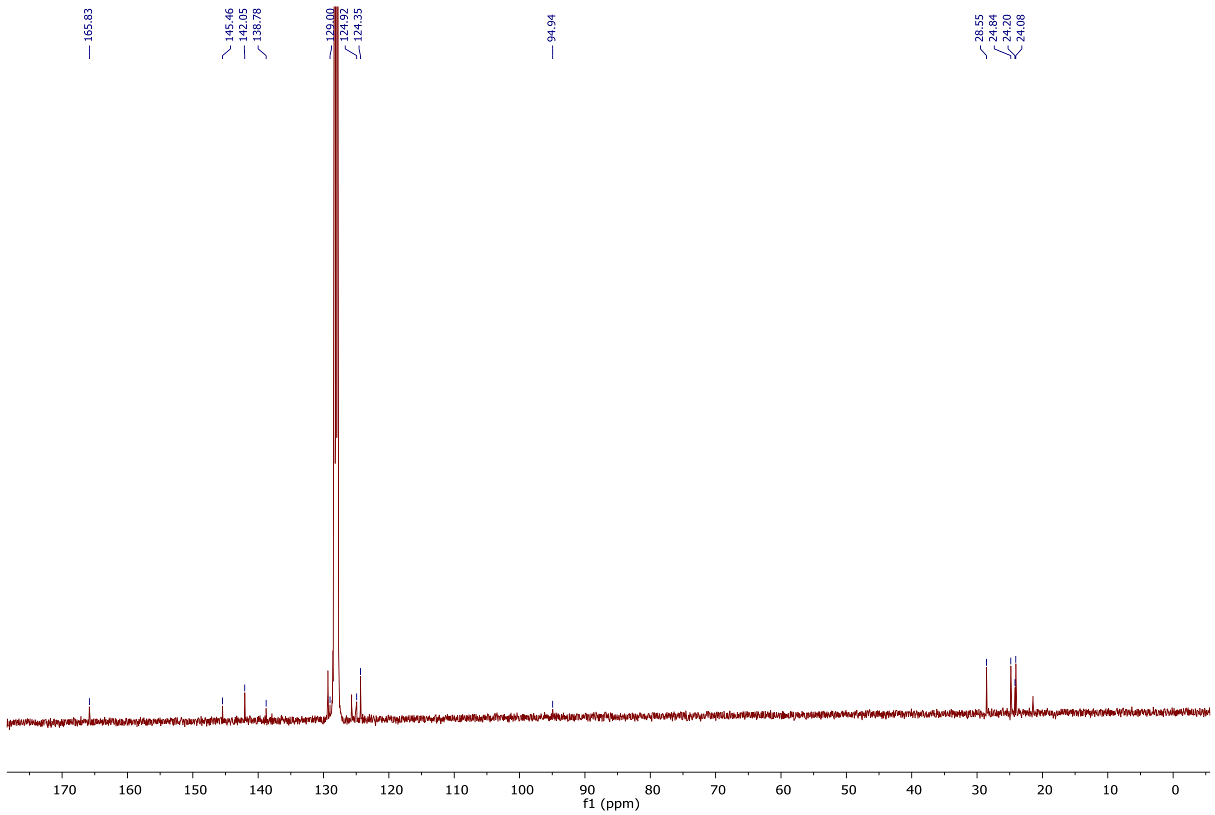

Figure S2. $^{13}C\{^{1}H\}$ NMR Spectrum (C_6D_6, 298 K, 100.62 MHz) for **9**, residual protio-toluene present.

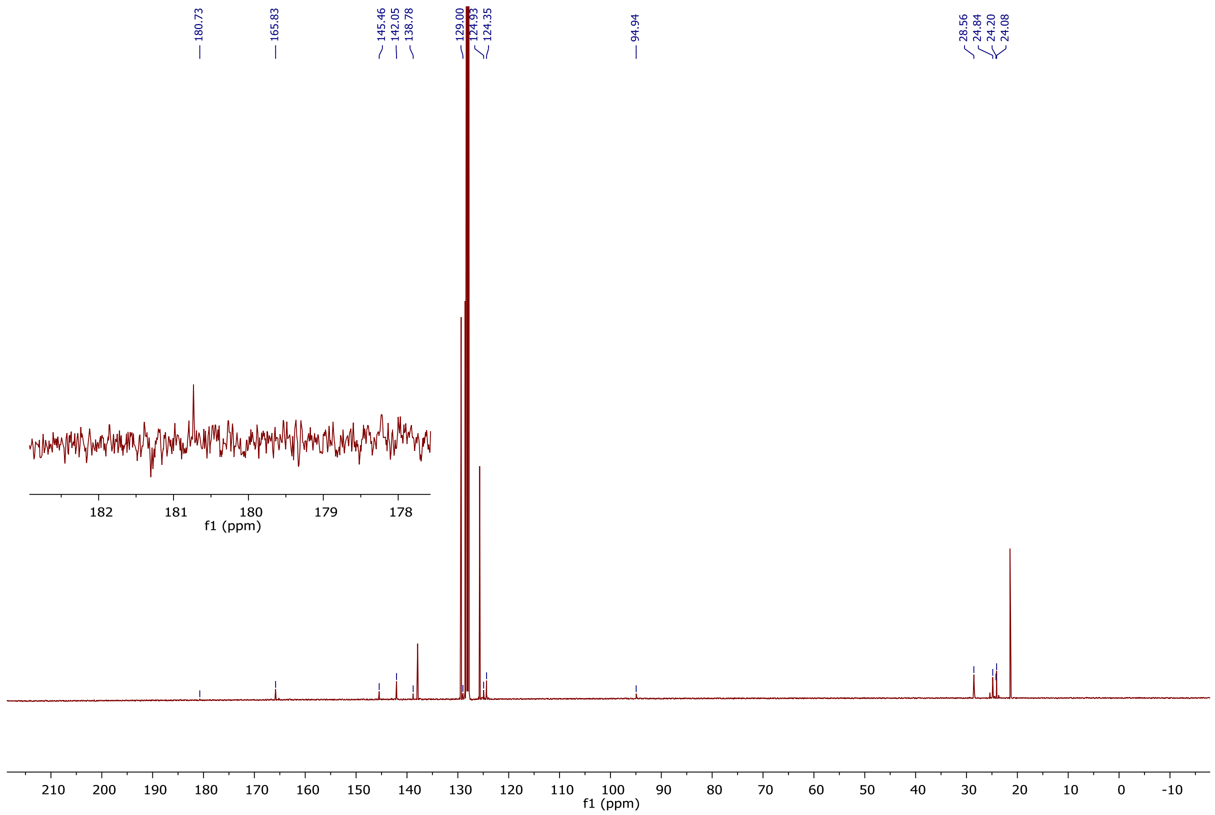

Figure S3. $^{13}C\{^{1}H\}$ NMR Spectrum (C_6D_6, 298 K, 100.62 MHz) for **9** with more protio-toluene present.

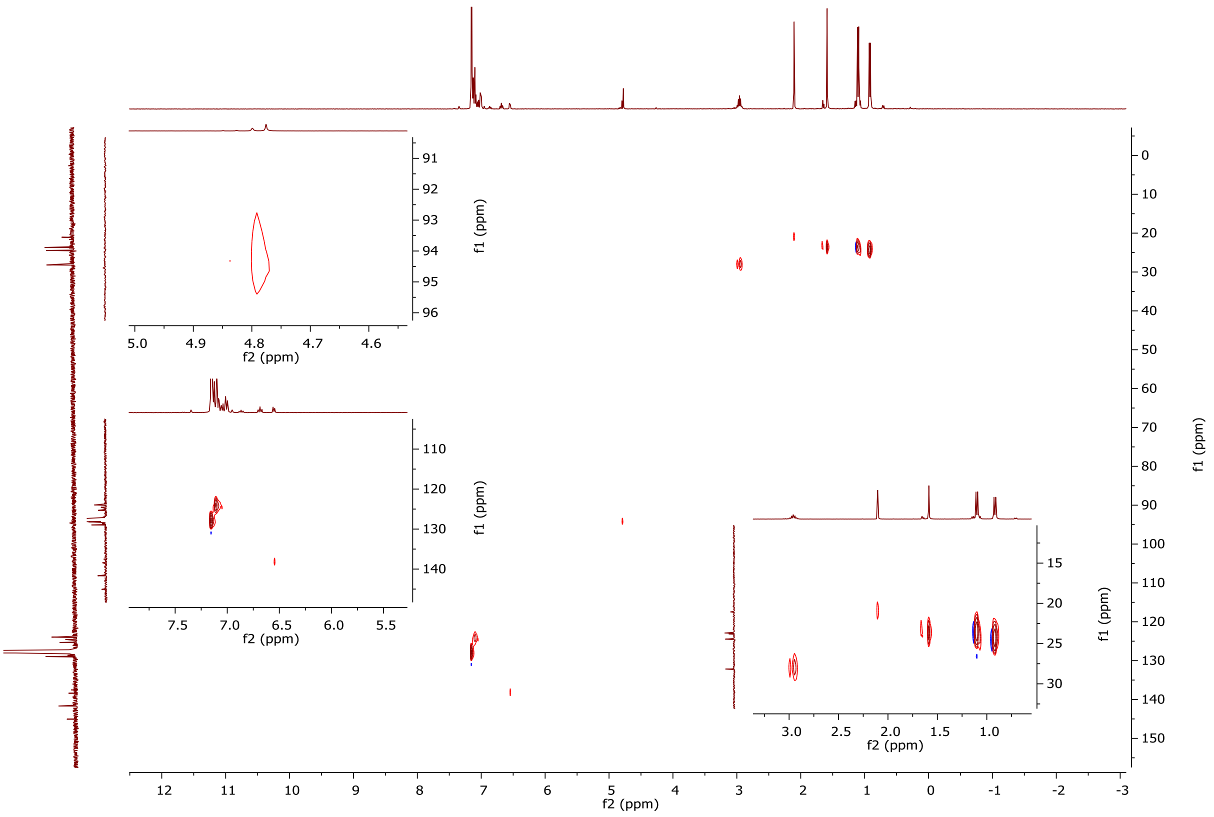

Figure S4. ^{1}H-^{13}C HSQC trace (C_6D_6, 298 K, 400.13, 100.62 MHz) for **9**.

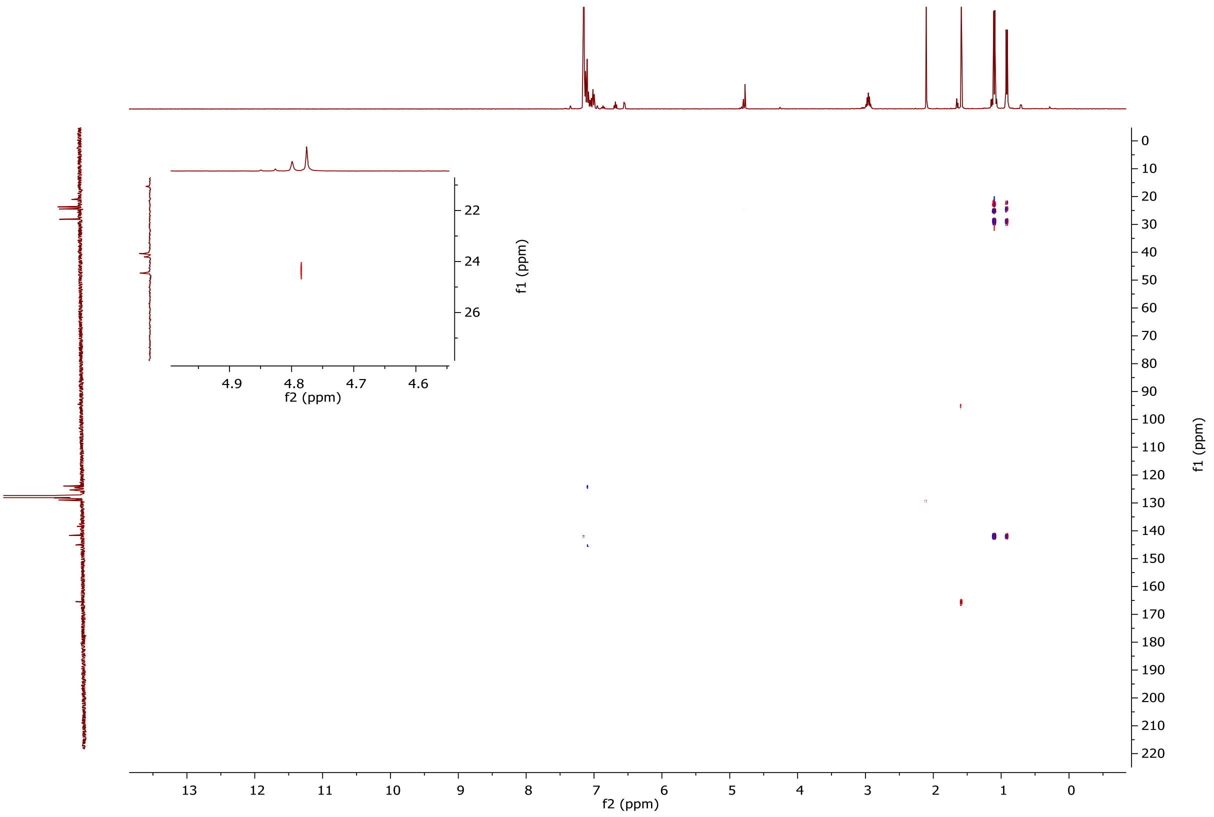

Figure S5. ^{1}H-^{13}C HMBC trace (C_6D_6, 298 K, 400.13, 100.62 MHz) for **9**.

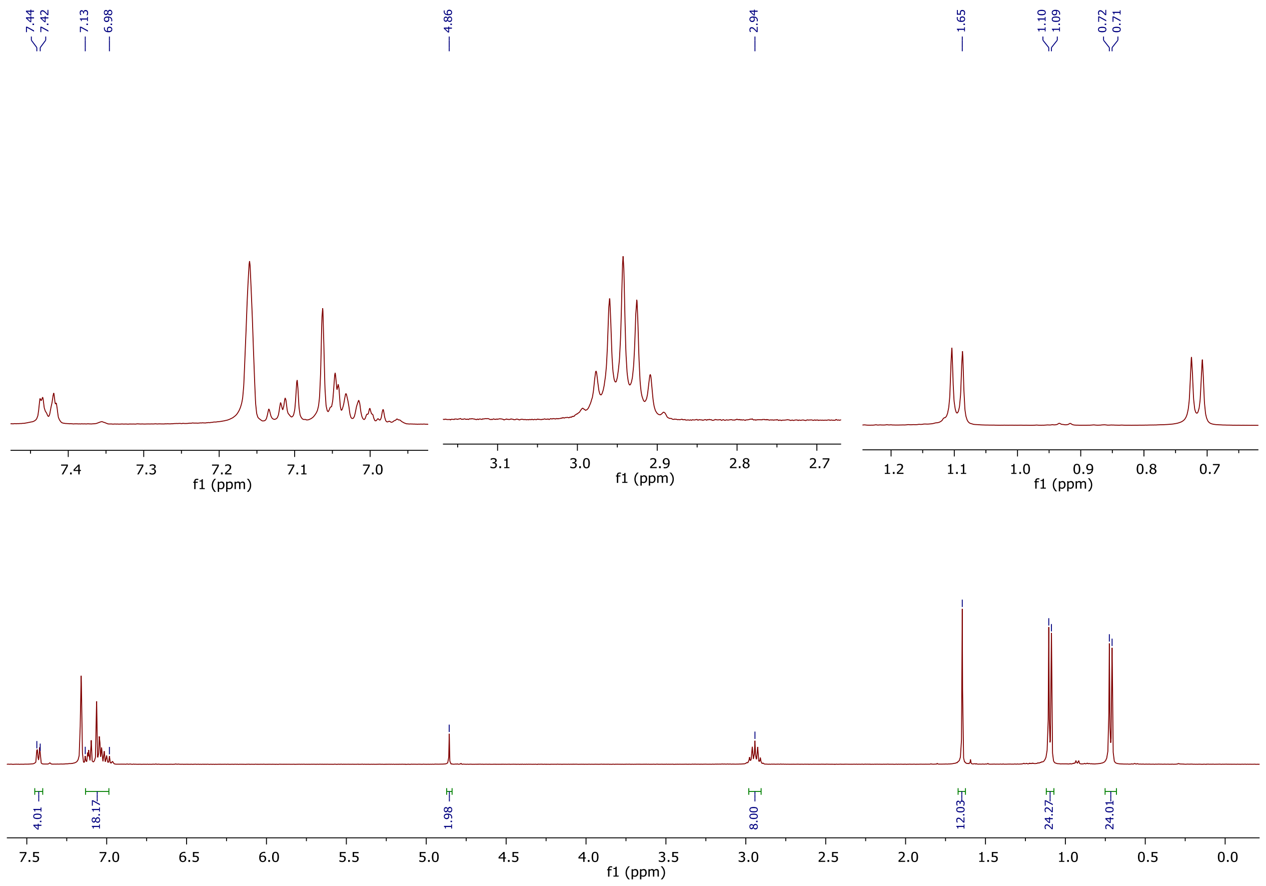

Figure S6. ^{1}H NMR Spectrum (C_6D_6, 298 K, 400.13 MHz) for **10**.

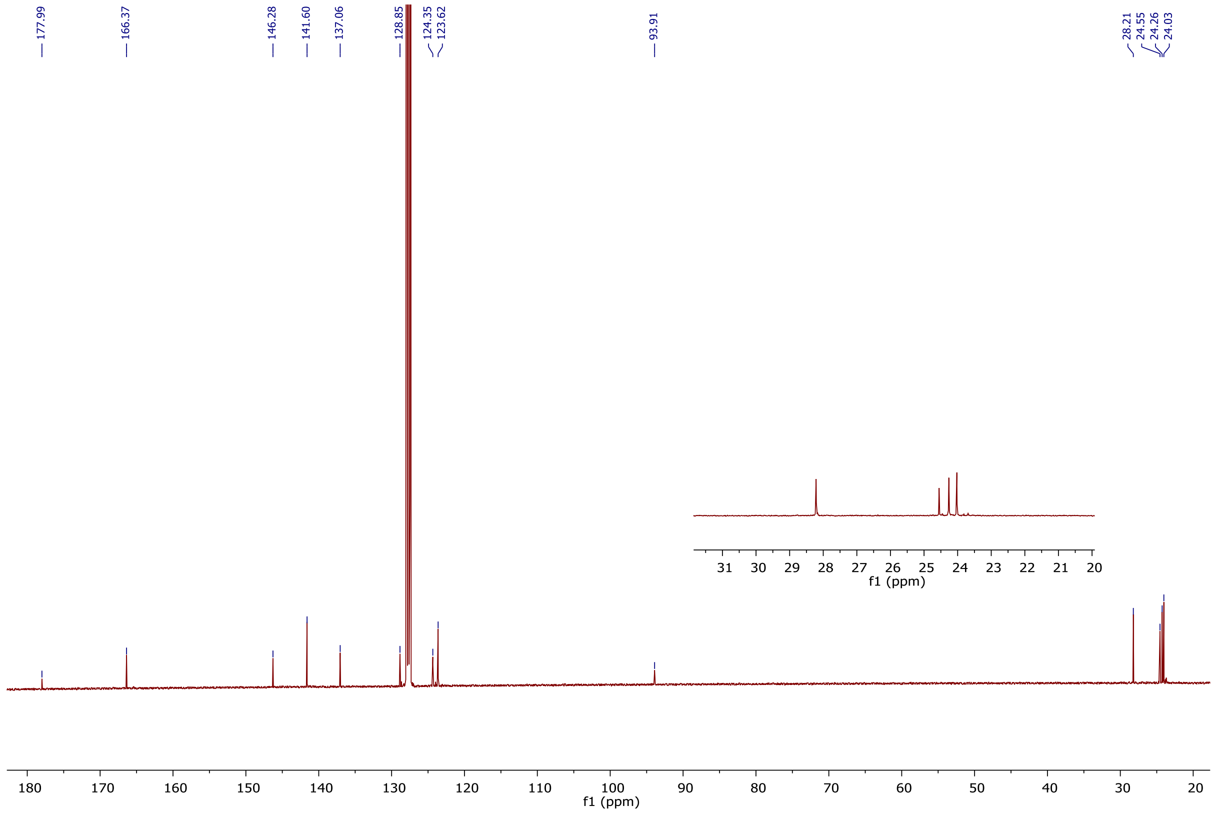

Figure S7. $^{13}C\{^{1}H\}$ NMR Spectrum (C_6D_6, 298 K, 100.62 MHz) for **10**.

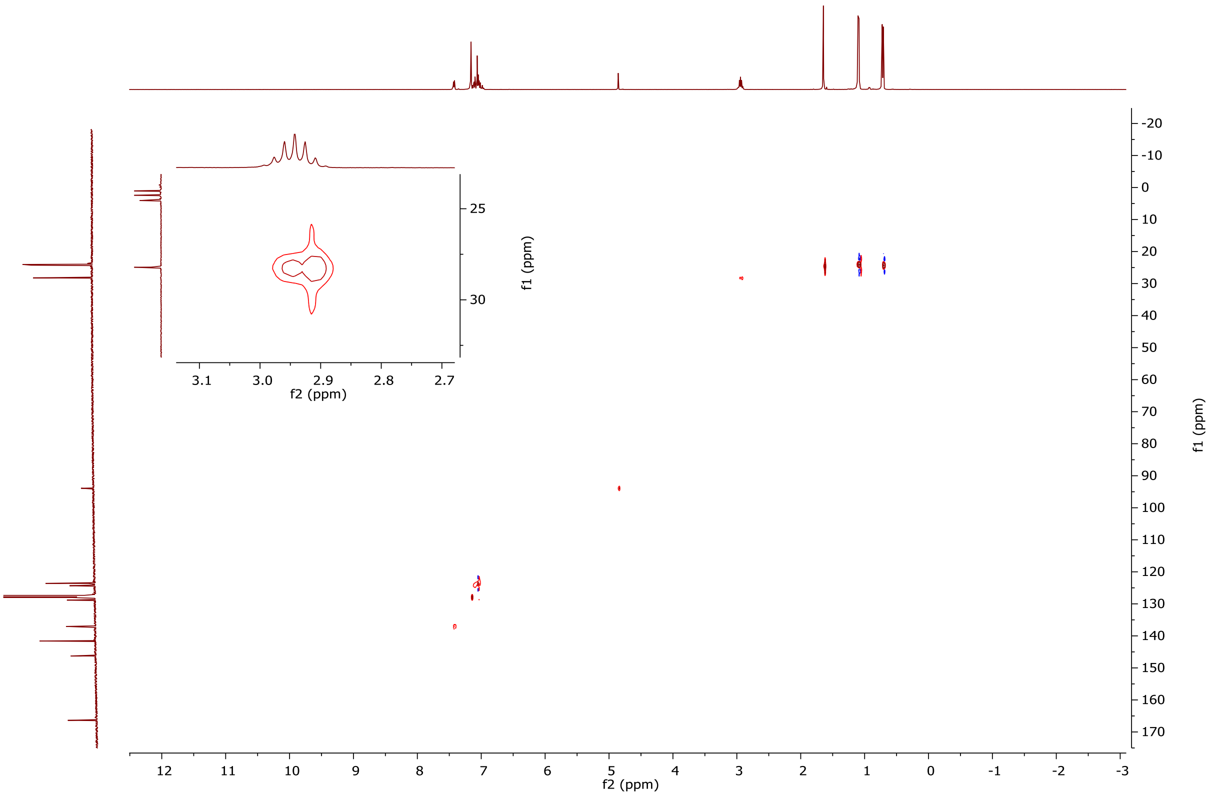

Figure S8. ^{1}H-^{13}C HSQC trace (C_6D_6, 298 K, 400.13, 100.62 MHz) for **10**.

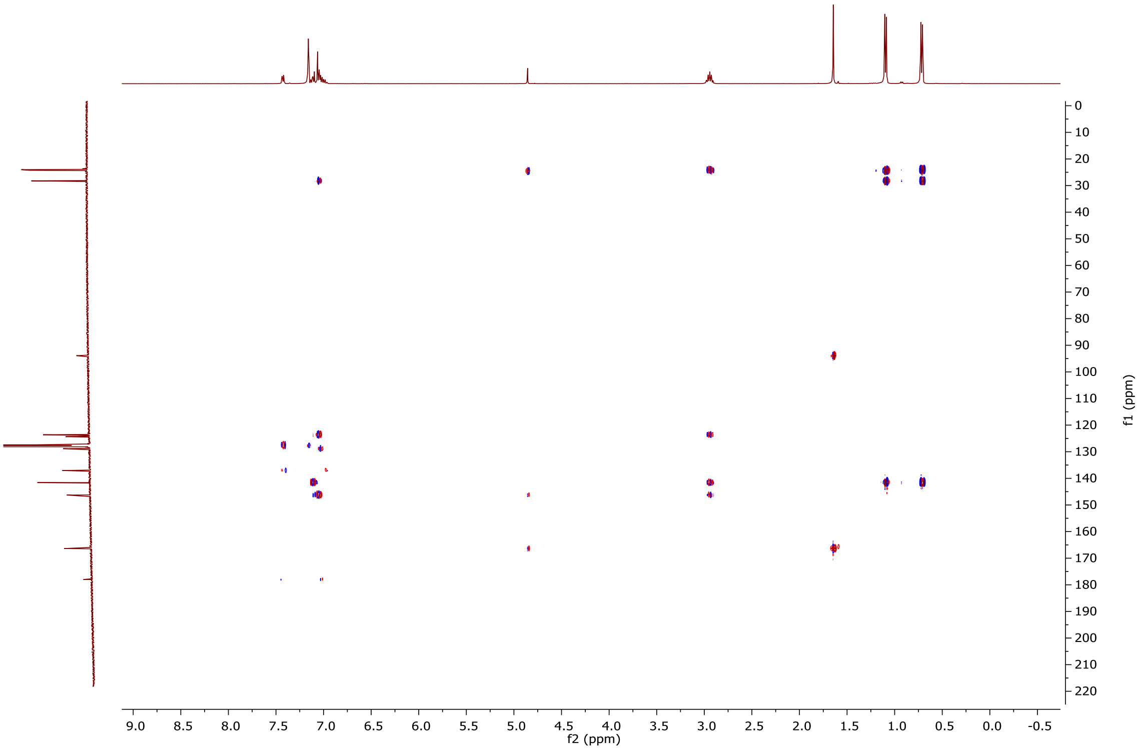

Figure S9. ^{1}H-^{13}C HMBC trace (C_6D_6, 298 K, 400.13, 100.62 MHz) for **10**.

Reactions of [(BDI)CaPh]$_2$ (10) with Arylbromides

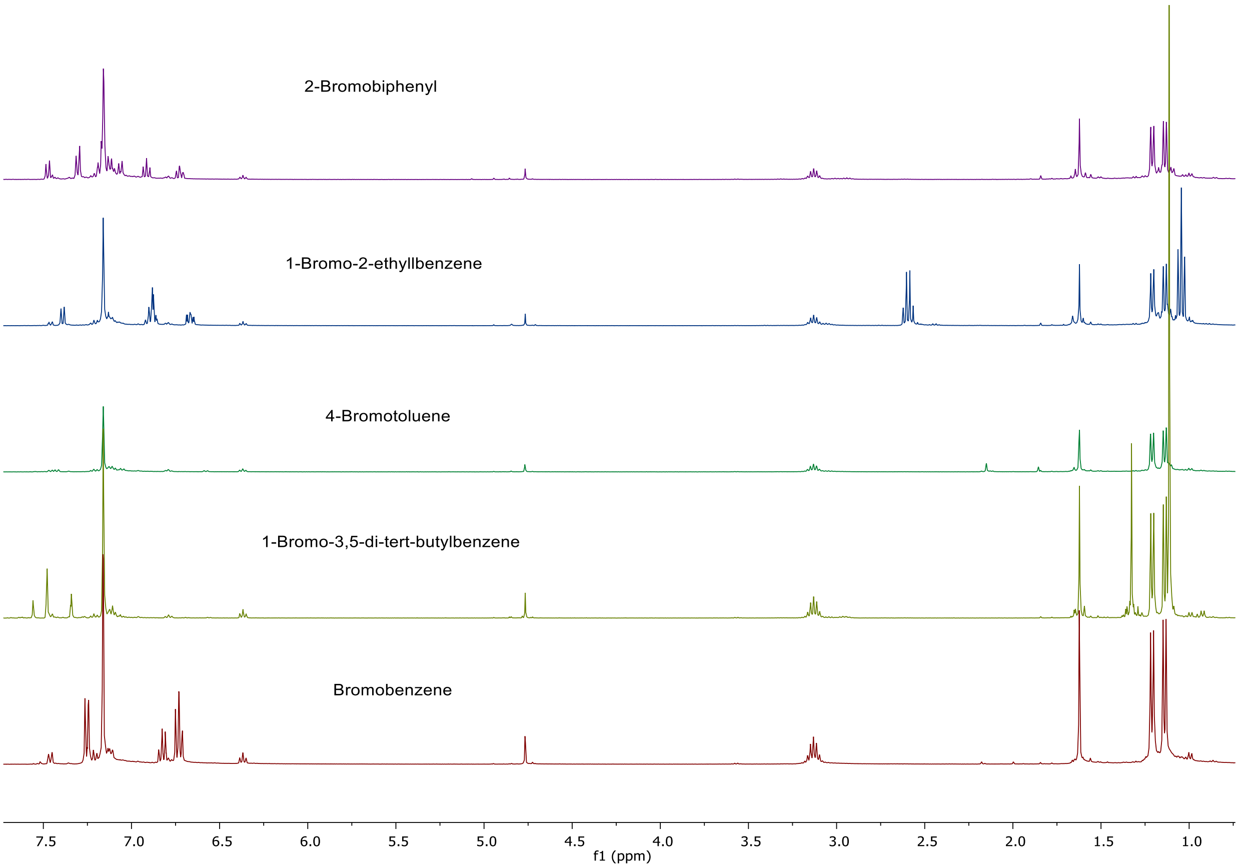

Figure S10. Overlayed ^{1}H NMR spectra (C_6D_6, 298 K, 400.13 MHz) from reacting **10** with different arylbromides (as per label), affording [(BDI)Ca(Br)PhCa(BDI)] (**11**) and the respective biaryl in each case.

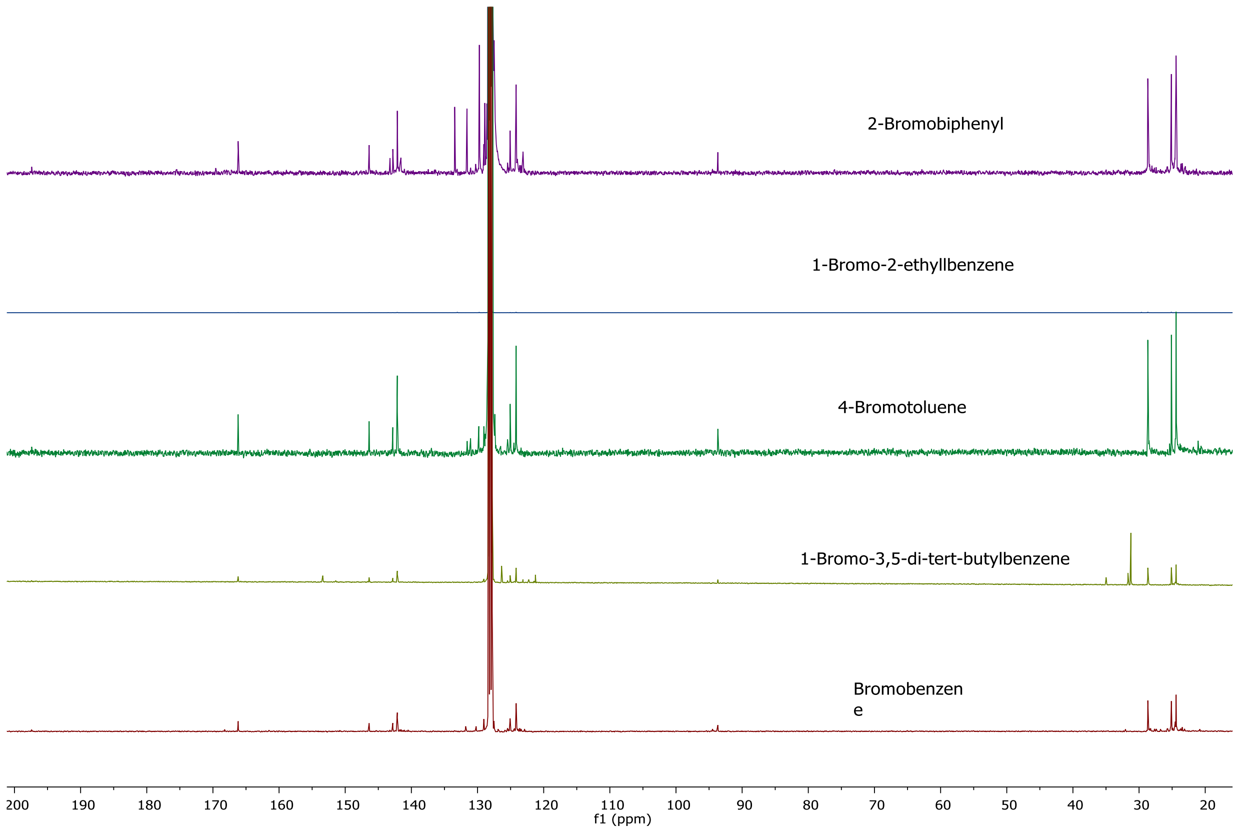

Figure S11. Overlayed $^{13}C\{^{1}H\}$ NMR spectra (C_6D_6, 298 K, 100.62 MHz) from reacting **10** with different arylbromides (as per label), affording [(BDI)Ca(Br)PhCa(BDI)] (**11**) and the respective biaryl in each case.

Reaction of 10 with bromobenzene

Bromobenzene (3.2 µl, 0.03 mmol) was added to a C_6D_6 solution of **10** (33 mg, 0.03 mmol) and mixed at ambient temperature for 144 hours, resulting in an orange solution. Alternatively, this reaction can be performed in 16 hours at 60 °C. Conversions were estimated by ^{1}H NMR through comparison of the product peaks *versus* the methyl resonances of hexamethylbenzene, which was added as an internal standard, to be 45% (**11**) and 45% (biphenyl). Exposure to vacuum leads to the generation of homoleptic calcium complex [[(BDI)$_2$Ca]].

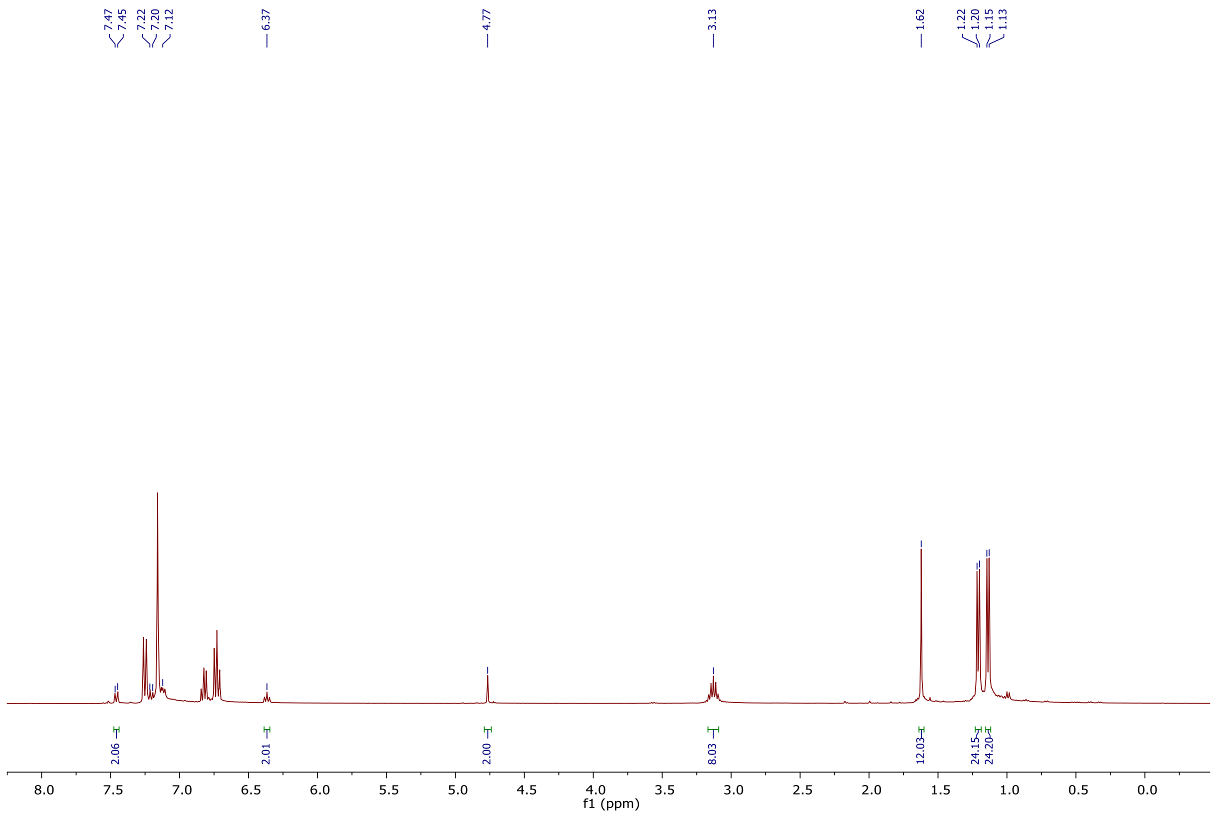

Figure S12.^{1}H NMR spectrum (C_6D_6, 298 K, 400.13 MHz) resulting from the reaction of **10** and bromobenzene for 144 hours at ambient temperature. The sample contains bromobenzene, [(BDI)Ca(Br)PhCa(BDI)] (**11**) and biphenyl.

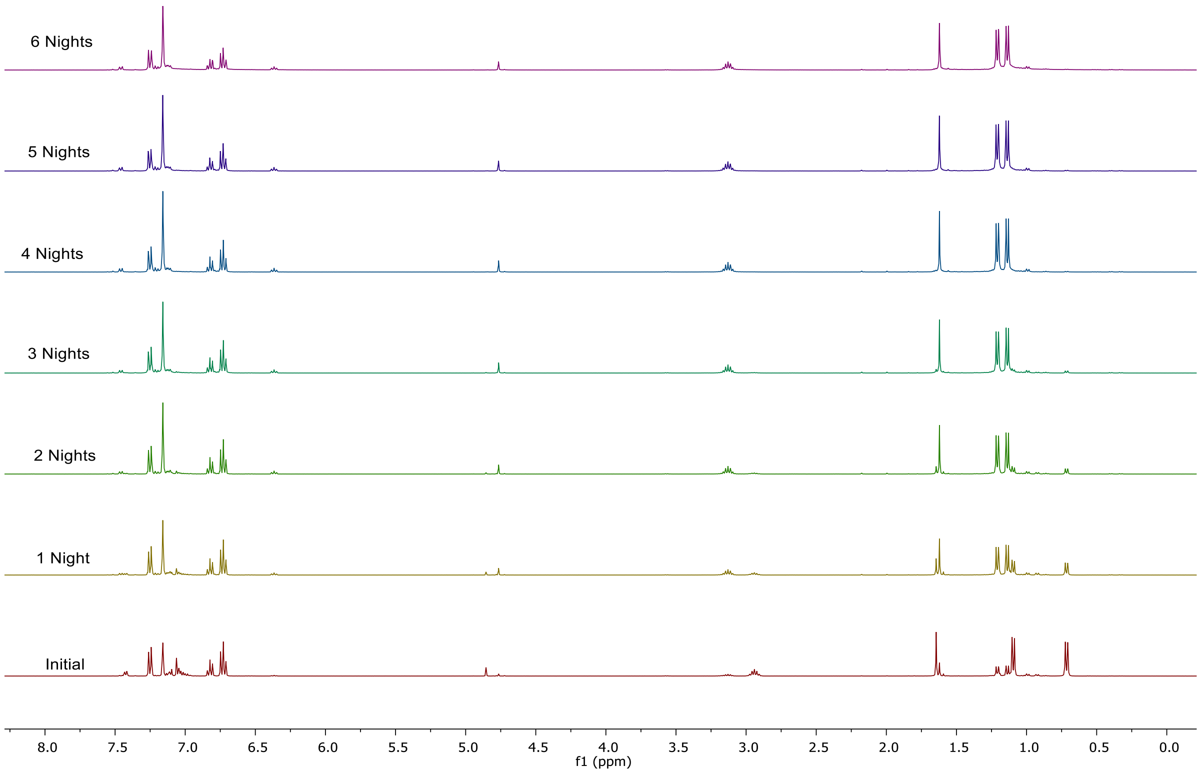

Figure S13. Stacked ^{1}H NMR spectra (C_6D_6, 298 K, 400.13 MHz) of the reaction between **10** and bromobenzene at ambient temperature.

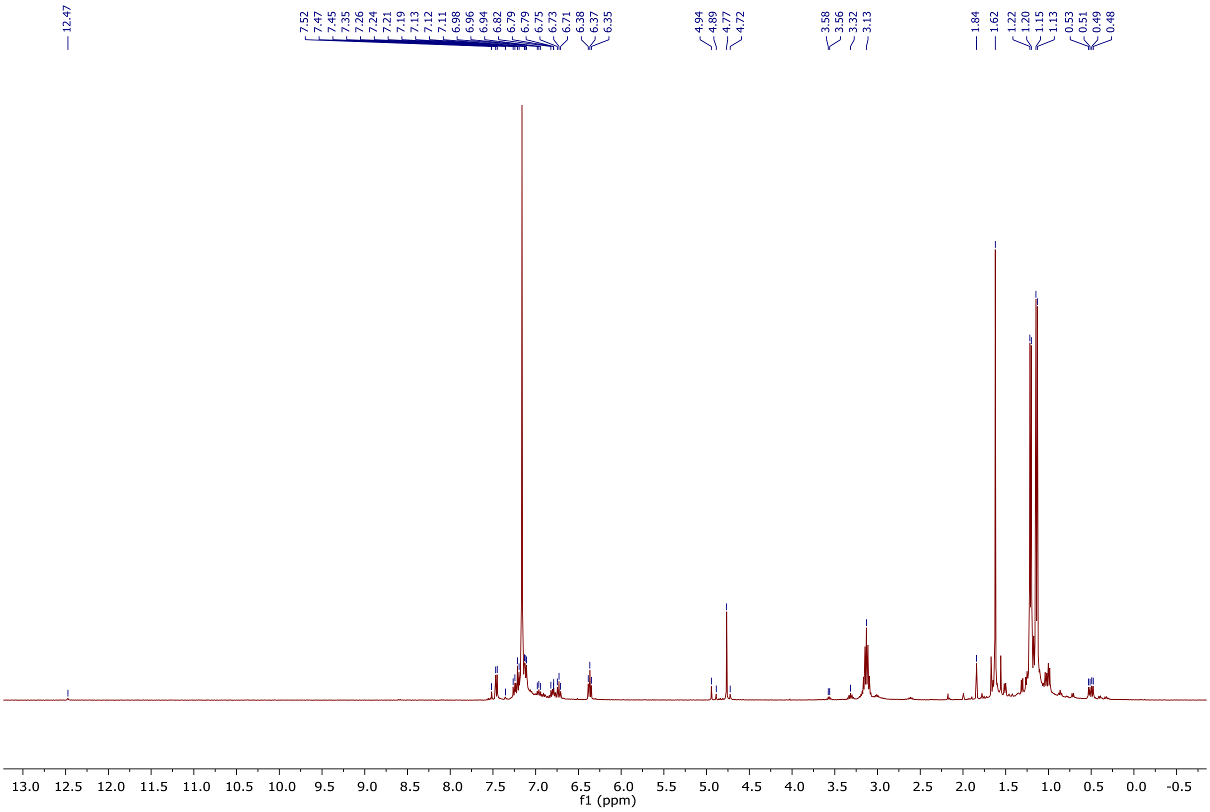

Figure S14. ^{1}H NMR spectrum (C_6D_6, 298 K, 400.13 MHz) after exposing the products from the reaction of **10** and bromobenzene for 144 hours at ambient temperature to vacuum.

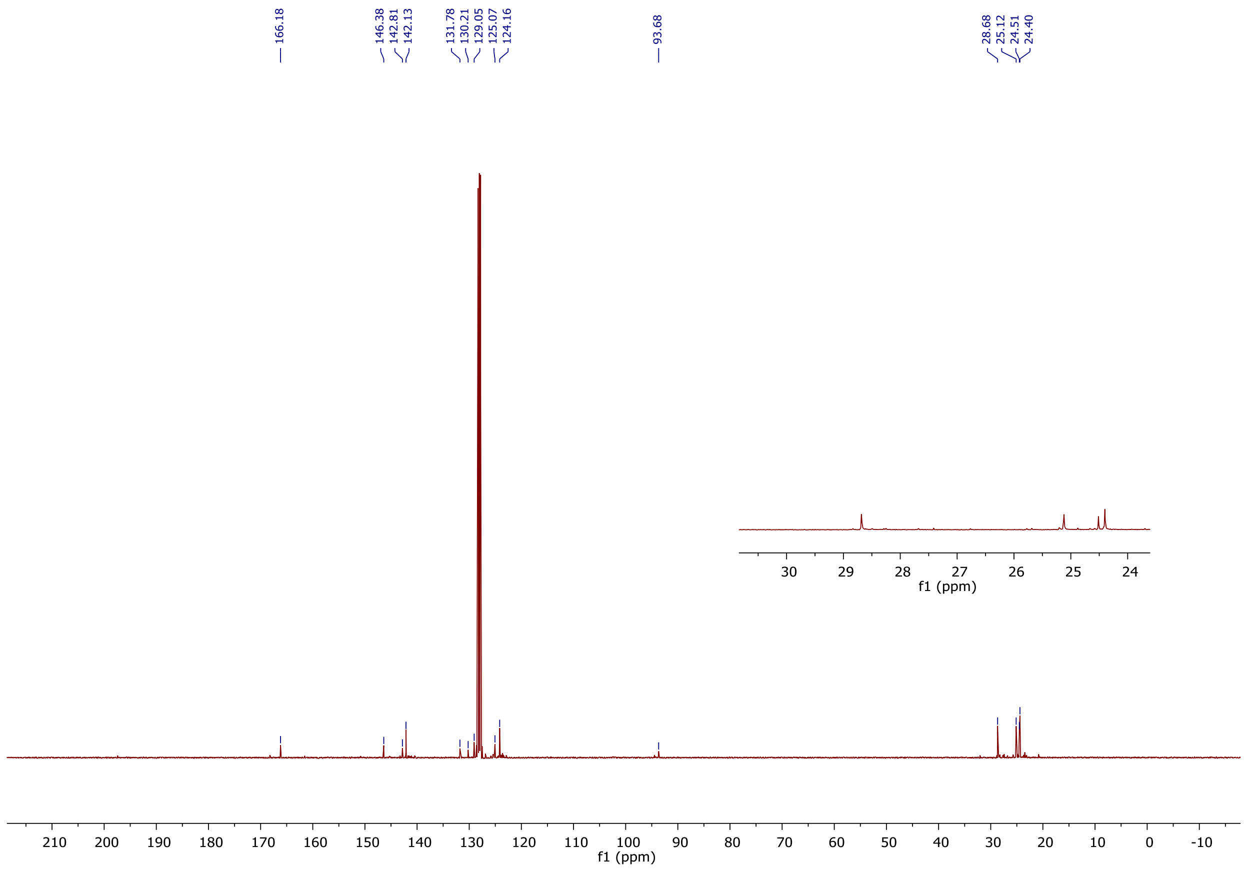

Figure S15. $^{13}C\{^{1}H\}$ NMR spectrum (C_6D_6, 298 K, 100.62 MHz) after exposing the products from the reaction of **10** and bromobenzene for 144 hours at ambient temperature to vacuum.

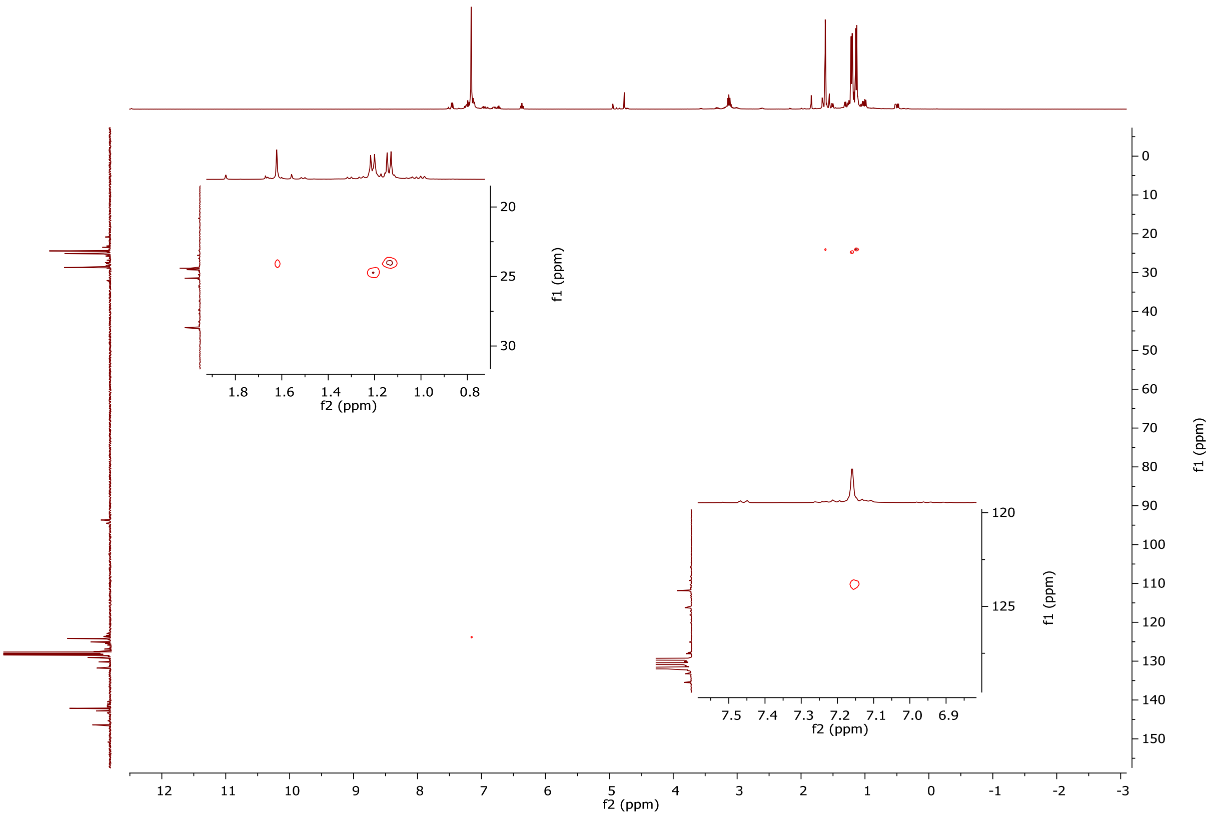

Figure S16. ^{1}H-^{13}C HSQC trace (C_6D_6, 298 K, 400.13, 100.62 MHz) after exposing the products from the reaction of **10** and bromobenzene for 144 hours at ambient temperature to vacuum.

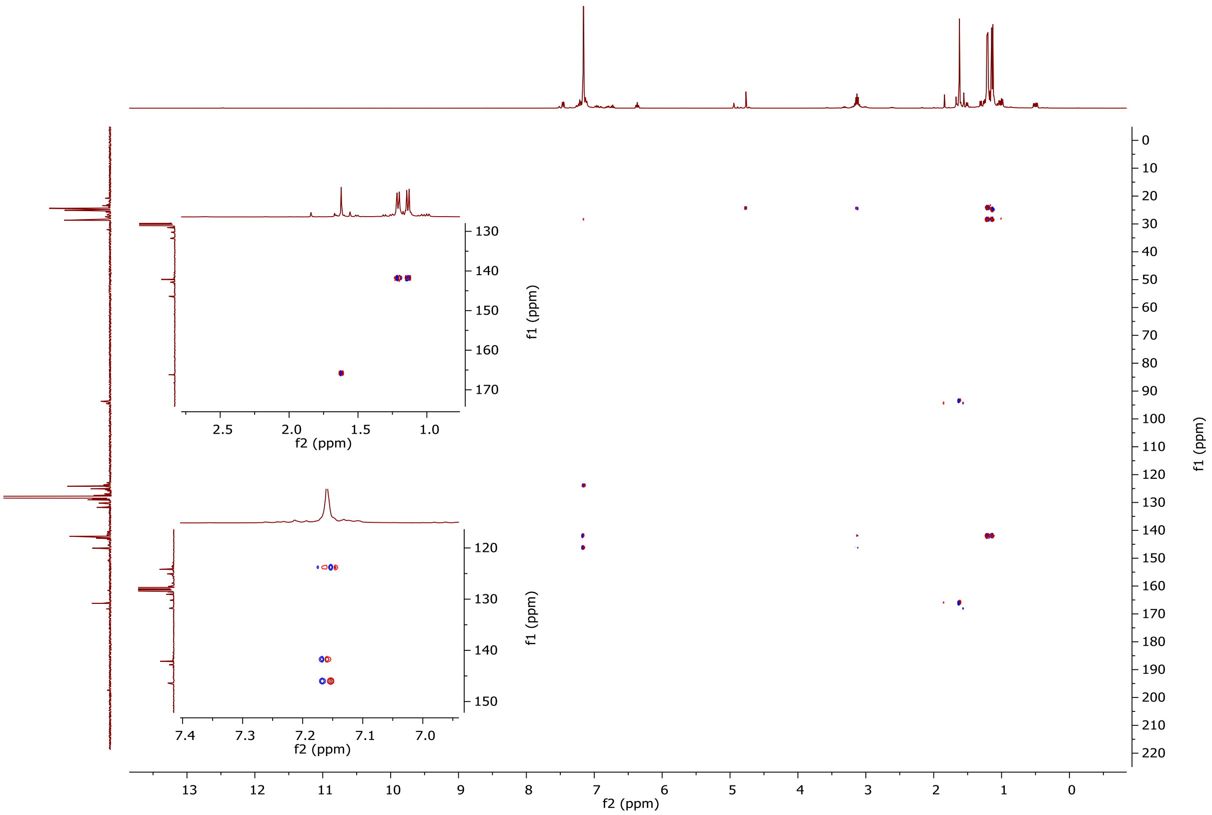

Figure S17. ^{1}H-^{13}C HMBC trace (C_6D_6, 298 K, 400.13, 100.62 MHz) after exposing the products from the reaction of **10** and bromobenzene for 144 hours at ambient temperature to vacuum.

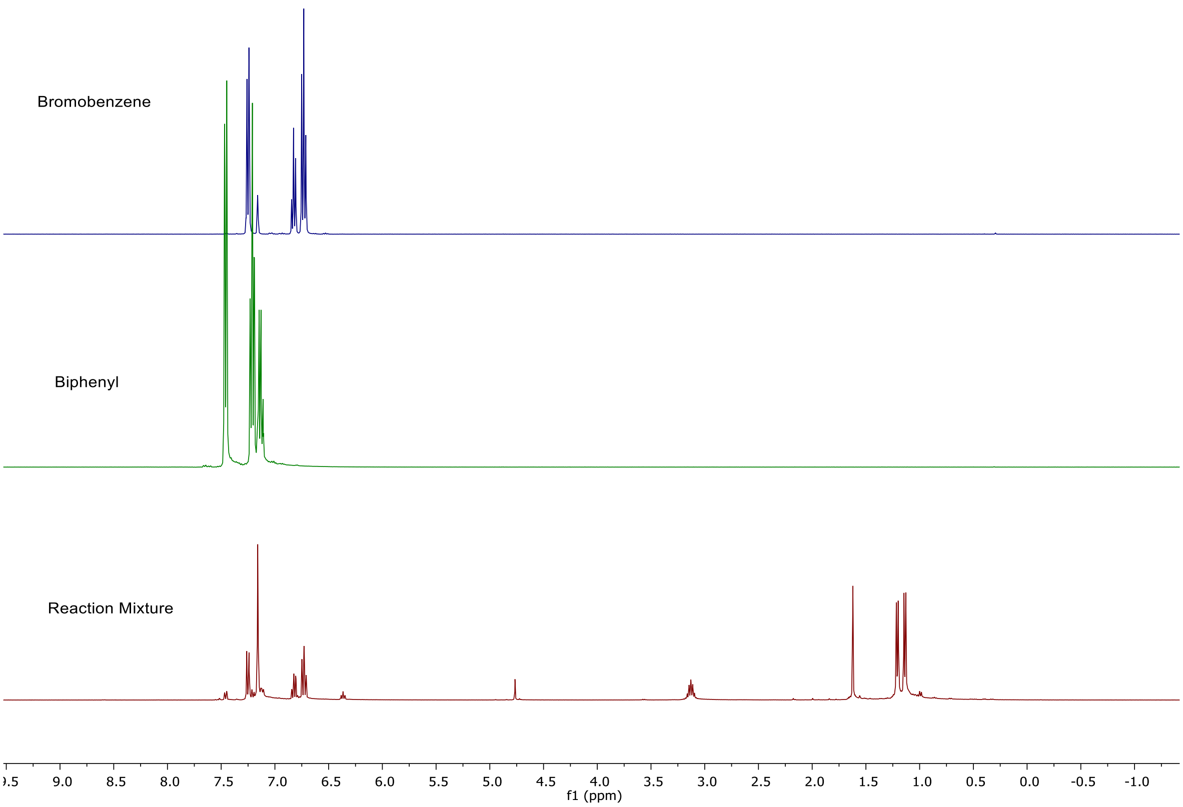

Figure S18. Stacked ^1H NMR spectra (C_6D_6, 298 K, 400.13 MHz) of the resulting spectrum from the reaction between **10** and bromobenzene at ambient temperature, with bromobenzene and biphenyl.

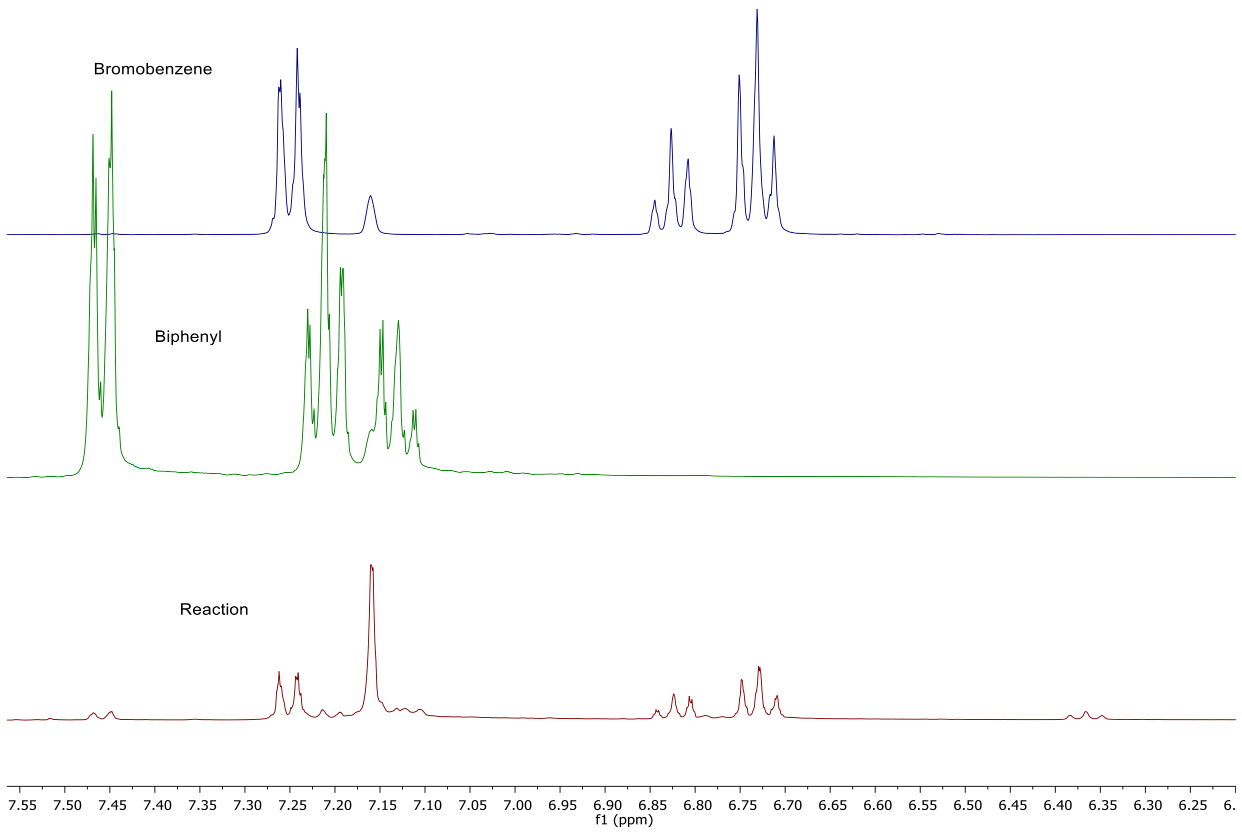

Figure S19. Expansion of the aromatic region shown in **Figure S18**, highlighting the presence of biphenyl.

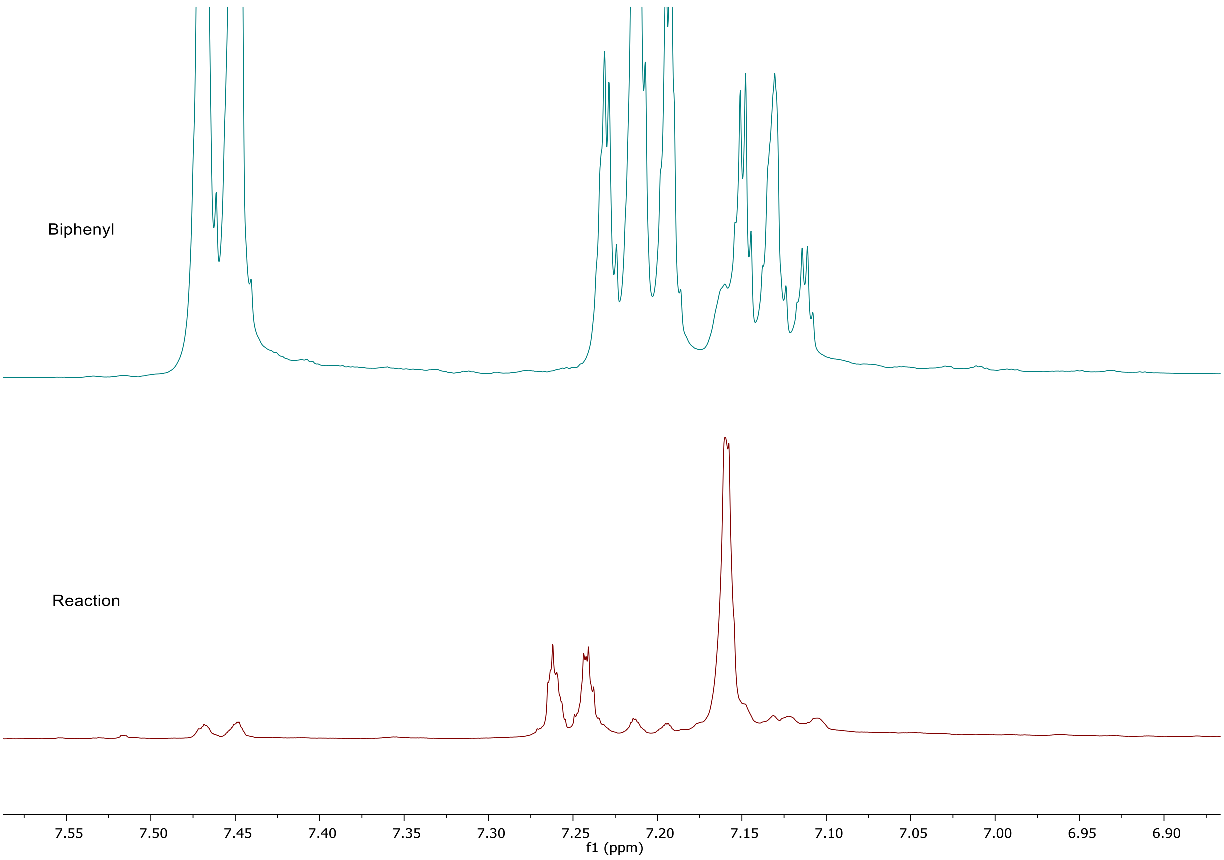

Figure S20. Expansion of the aromatic region of **Figure S12** with biphenyl, highlighting the presence of biphenyl in the reaction mixture.

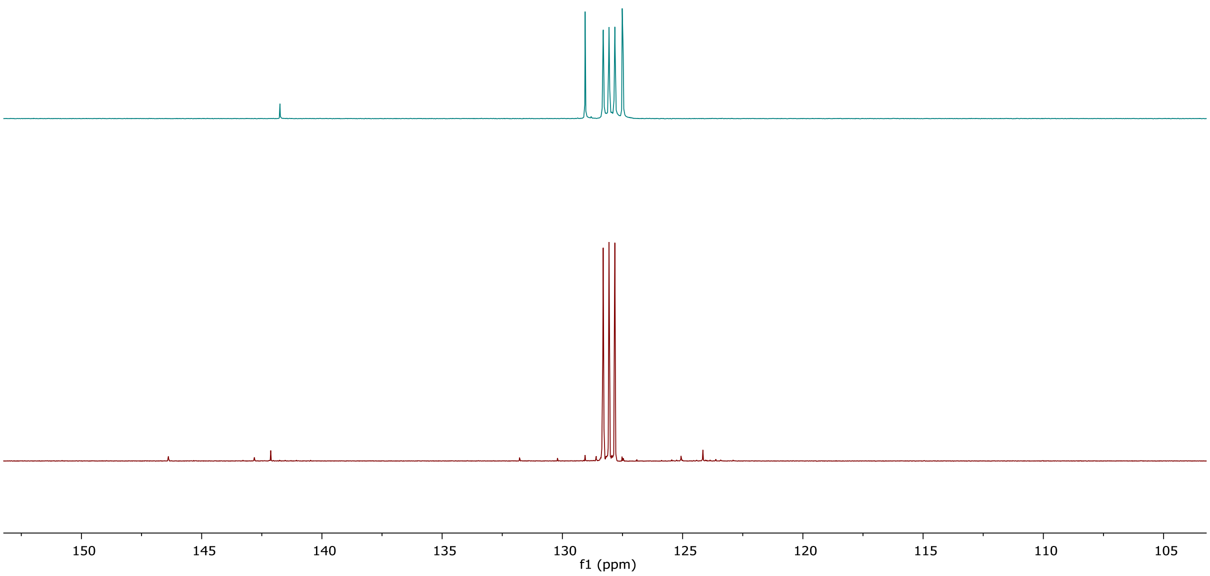

Figure S21. Stacked $^{13}C\{^{1}H\}$ NMR spectra (C_6D_6, 298 K, 100.62 MHz) illustrating the aromatic region of the resulting spectrum from the reaction between **10** and bromobenzene at ambient temperature, with biphenyl.

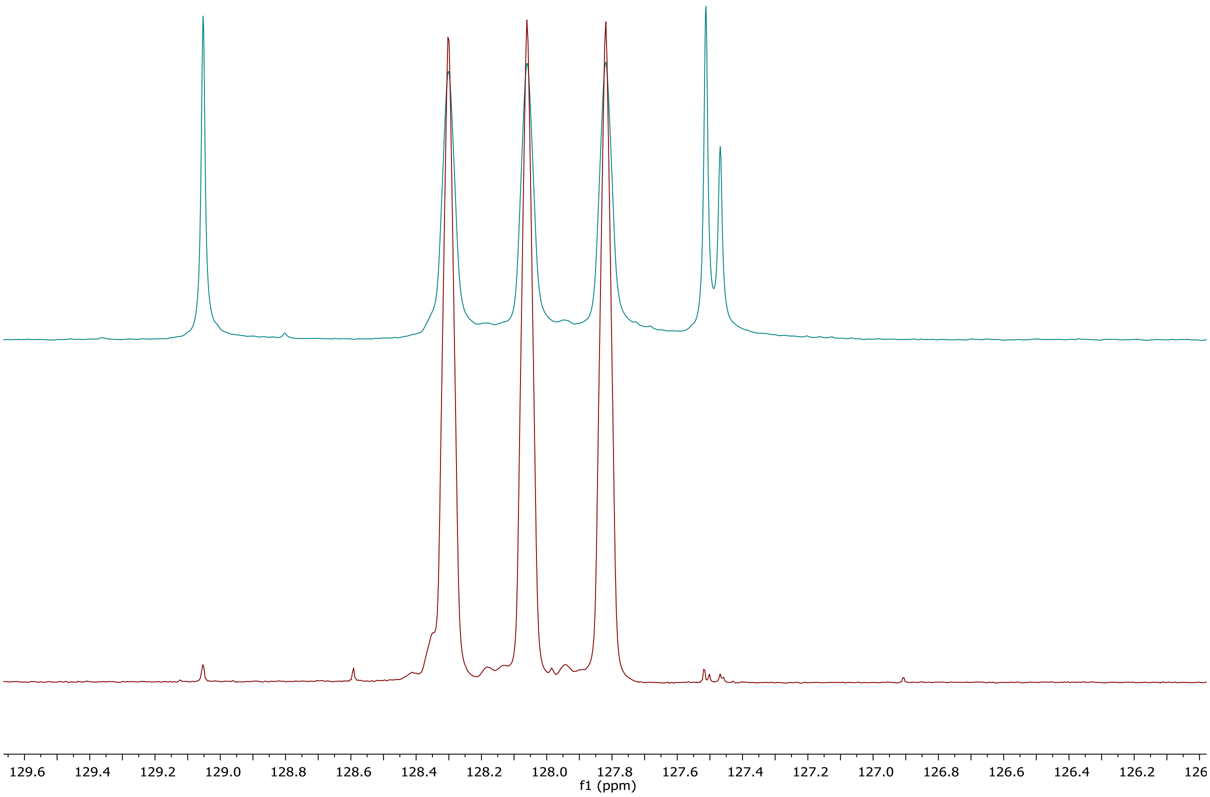

Figure S22. Expansion of part of the aromatic region shown in **Figure S21** with biphenyl, highlighting the presence of biphenyl.

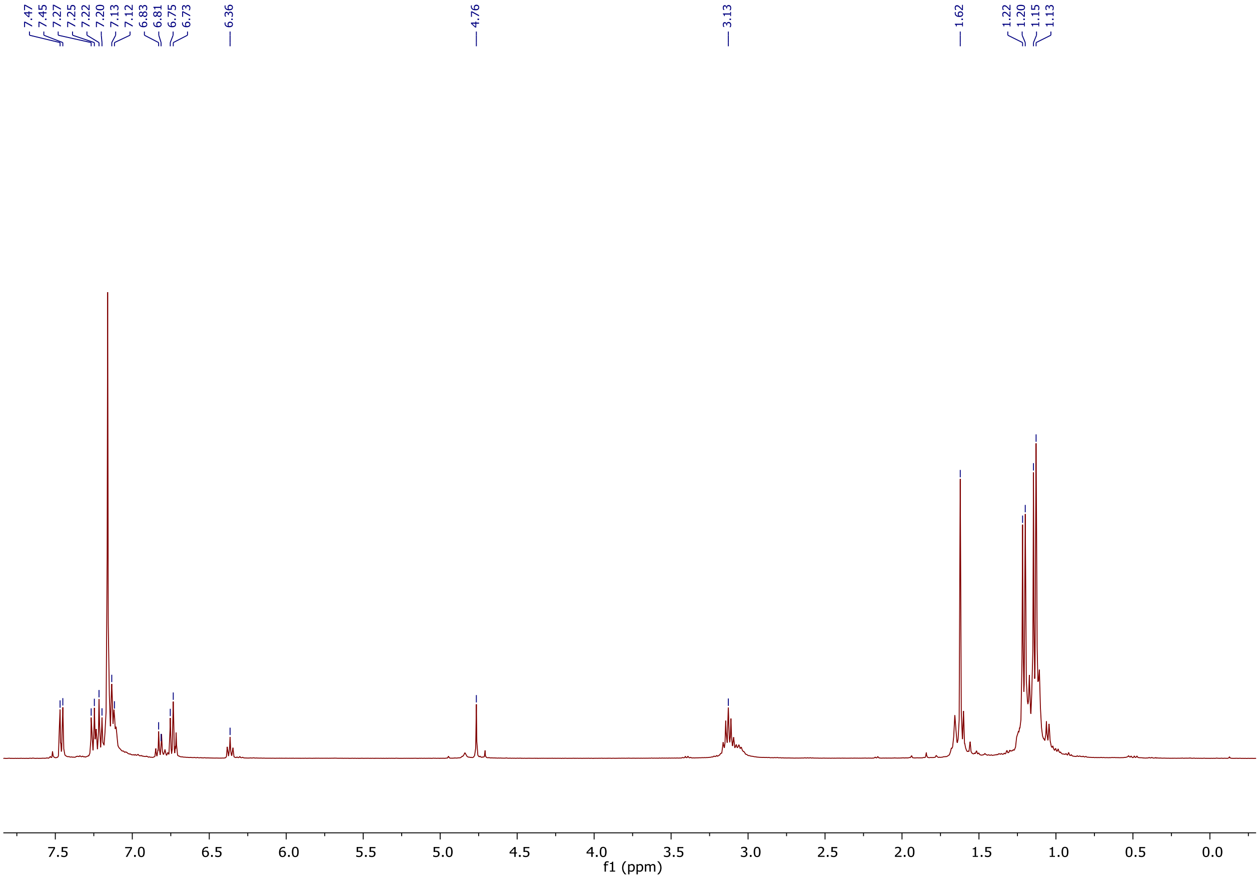

Figure S23. ^{1}H NMR spectrum (C_6D_6, 298 K, 400.13 MHz) resulting from the reaction of **10** and bromobenzene for 16 hours at 60 °C. The sample contains bromobenzene, [(BDI)Ca(Br)PhCa(BDI)] (**11**) and biphenyl.

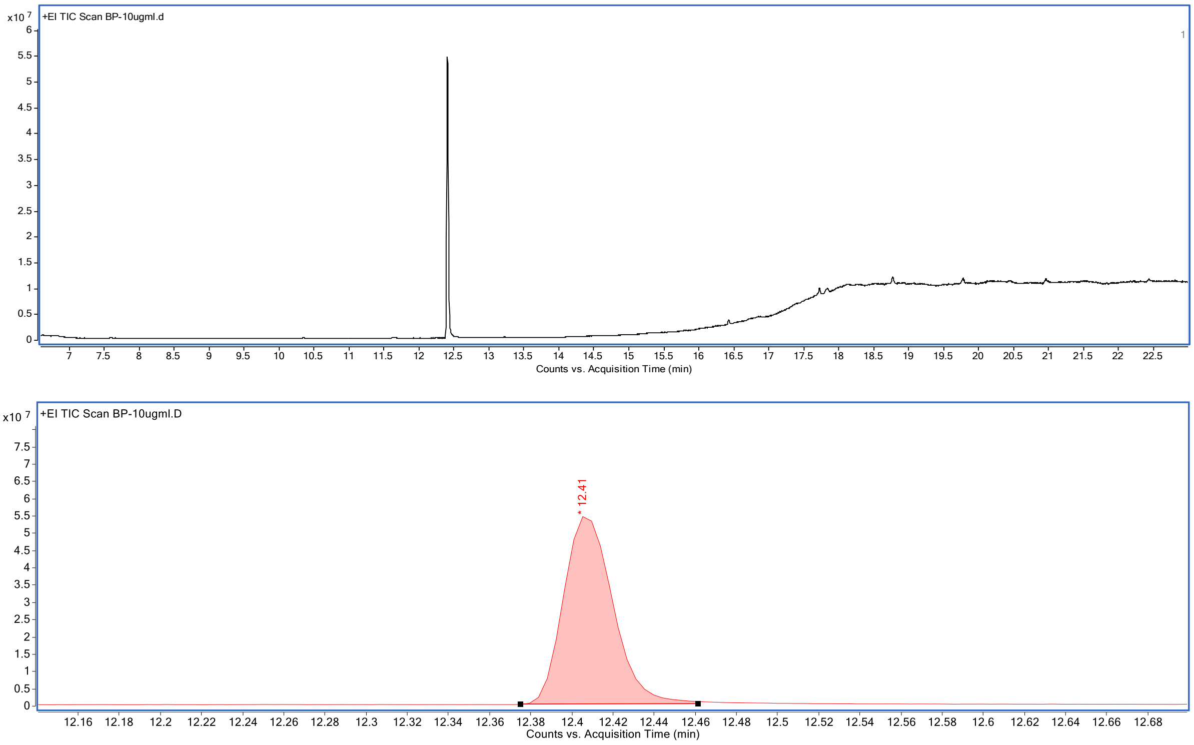

Figure S24. Reference GC-MS chromatogram of biphenyl. Top. Full chromatogram. Bottom. Expansion of the peak for biphenyl (12.4 min)

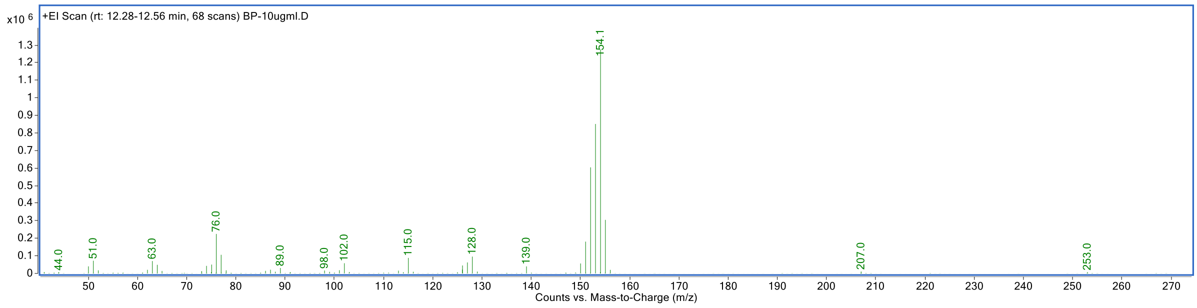

Figure S25. Reference GC-MS Trace showing the molecular ion of biphenyl.

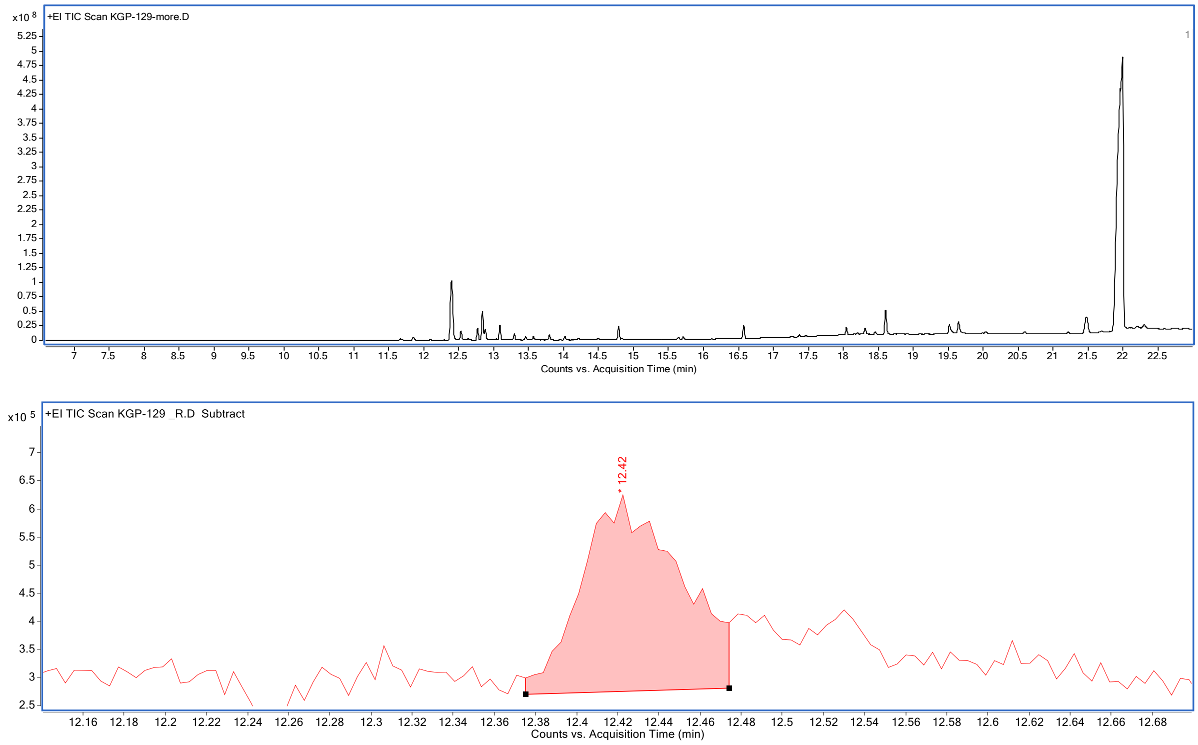

Figure S26. GC-MS chromatogram of the resulting spectra from the reaction between **10** and bromobenzene at ambient temperature. Top. Full chromatogram. Bottom. Expansion of the peak for biphenyl (12.4 min).

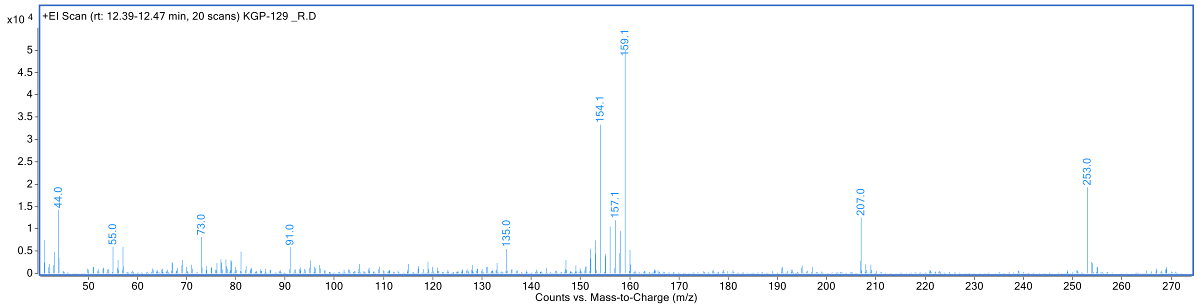

Figure S27. GC-MS trace **10** and bromobenzene at ambient temperature, showing the molecular ion for biphenyl.

Reaction of 10 with 1-bromo-3,5-tert-butylbenzene

1-Bromo-3,5-di-tert-butylbenzene (1.4 mg, 0.005 mmol) was added to a C_6D_6 solution of **10** (5.4 mg, 0.005 mmol) and mixed at ambient temperature for 120 hours, resulting in a pale-yellow solution. Conversions were estimated by ^{1}H NMR through comparison of the product peaks *versus* the methyl resonances of hexamethylbenzene, which was added as an internal standard, to be 72% (**11**) and 53% (3,5-tButyl-Biphenyl). Performing this reaction at 60 °C generates the homoleptic calcium complex $[(BDI)_2Ca]$.

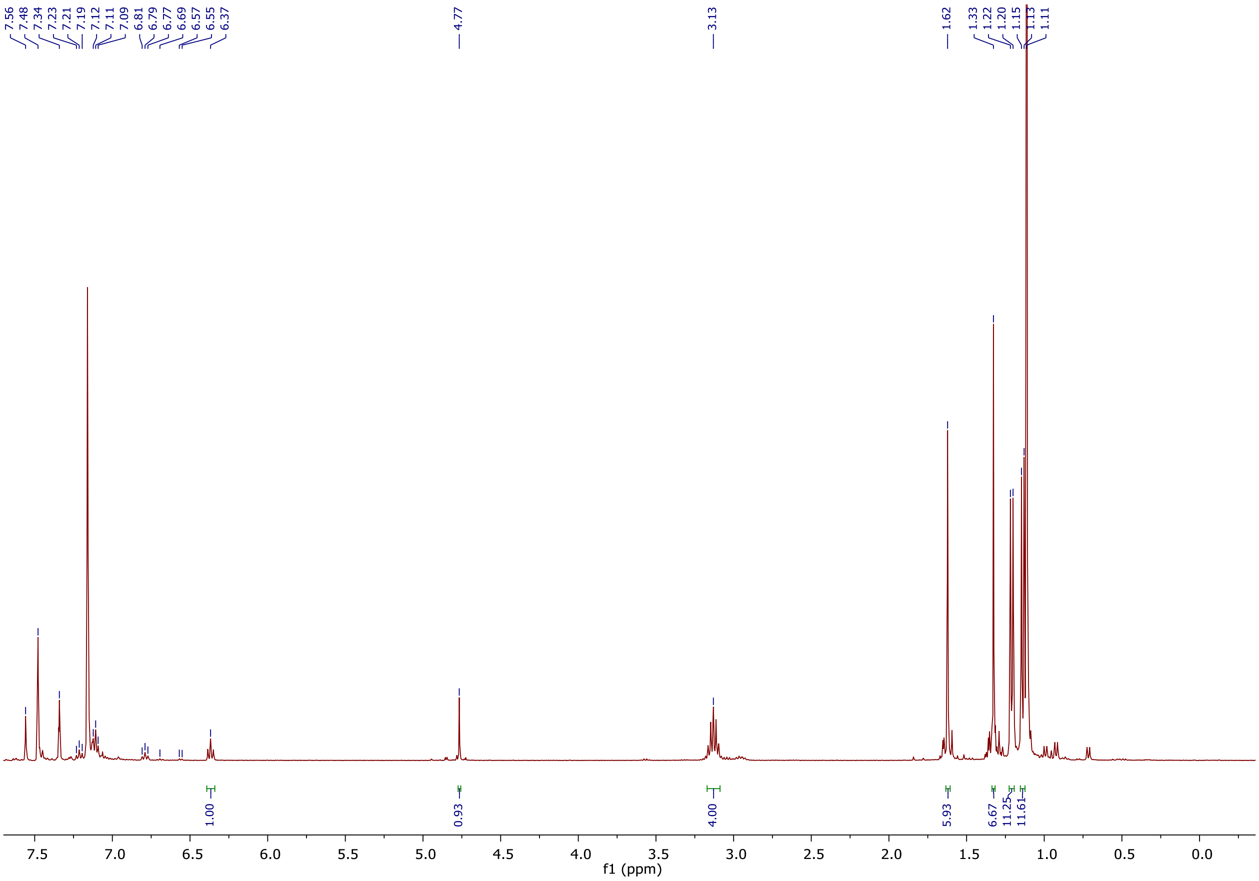

Figure S28. ^{1}H NMR spectrum (C_6D_6, 298 K, 400.13 MHz) resulting from the reaction of **10** and 1-bromo-3,5-di-tert-butylbenzene for 120 hours at ambient temperature. The sample contains 1-bromo-3,5-di-tert-butylbenzene, [(BDI)Ca(Br)PhCa(BDI)] (**11**) and 3,5-di-tertbutylbiphenyl.

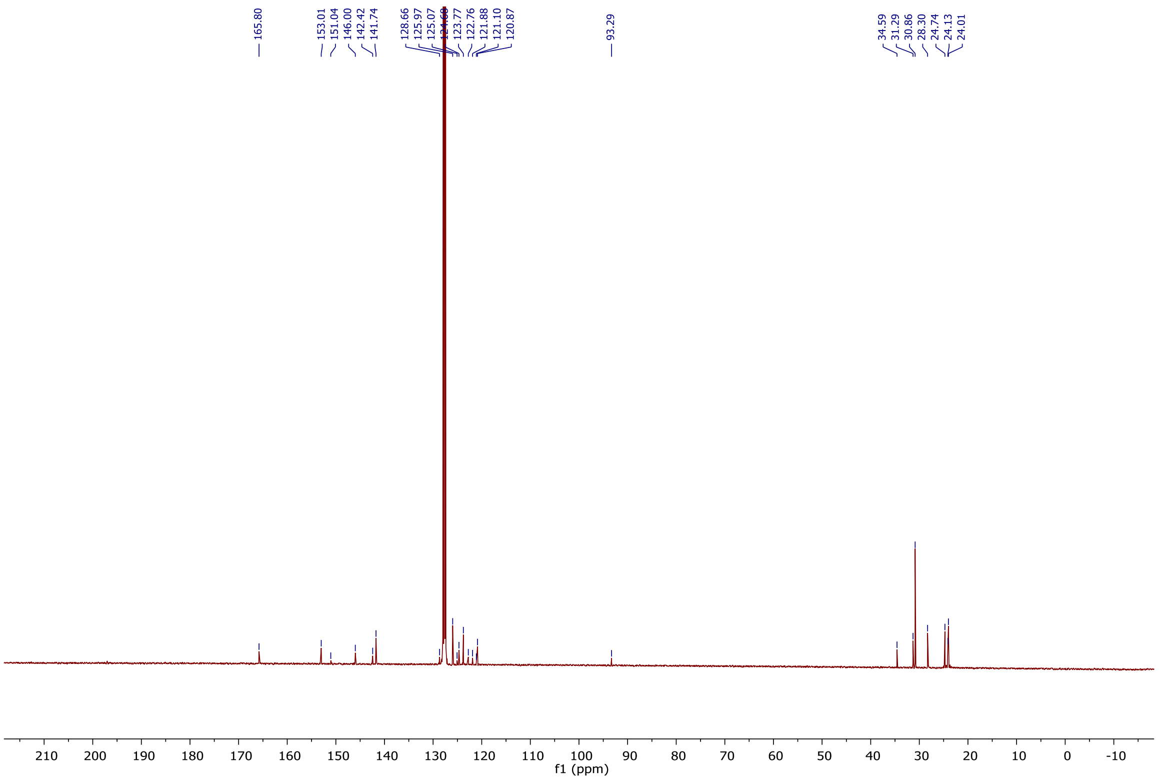

Figure S29. $^{13}C\{^{1}H\}$ NMR spectrum (C_6D_6, 298 K, 100.62 MHz) resulting from the reaction of **10** and 1-bromo-3,5-di-tert-butylbenzene for 120 hours at ambient temperature. The sample contains 1-bromo-3,5-di-tert-butylbenzene, [(BDI)Ca(Br)PhCa(BDI)] (**11**) and 3,5-di-tertbutylbiphenyl.

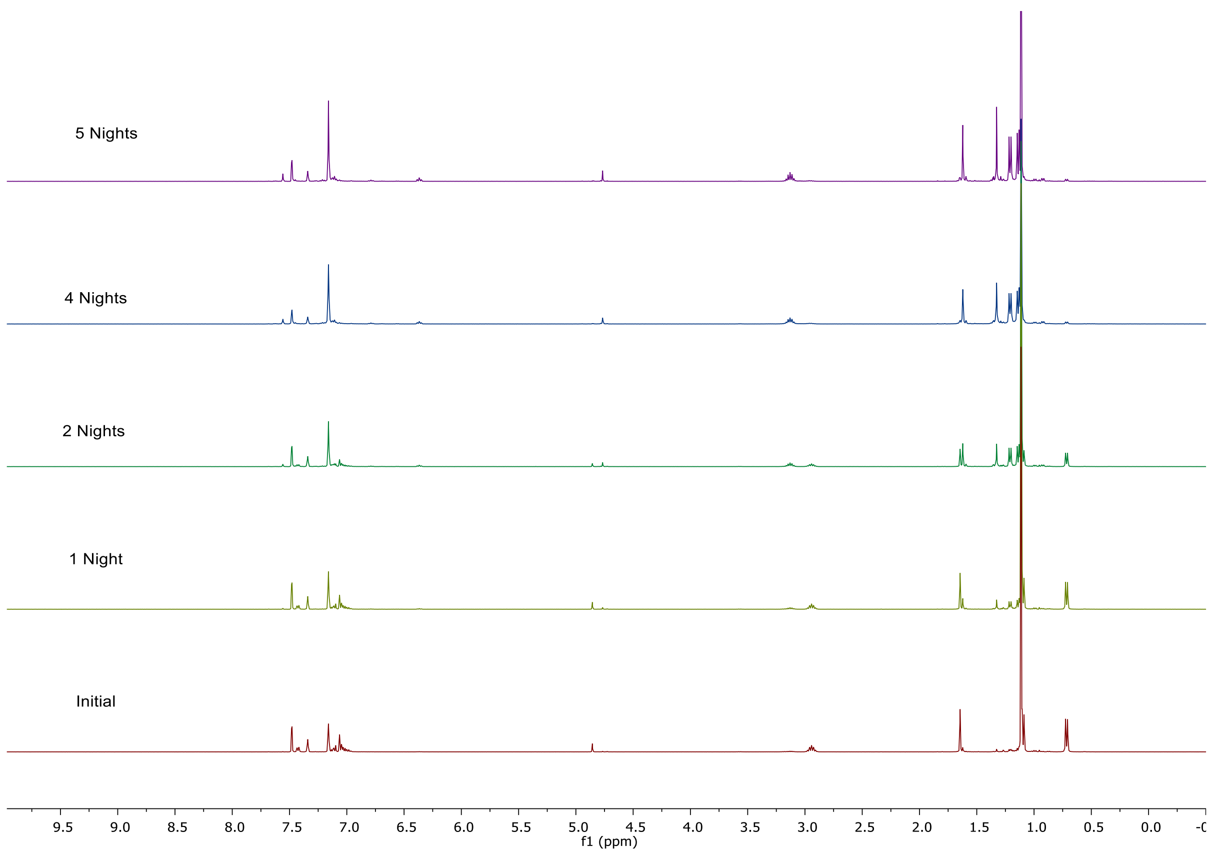

Figure S30. Stacked ^{1}H NMR spectra (C_6D_6, 298 K, 400.13 MHz) of the reaction between **10** and 1-bromo-3,5-di-tert-butylbenzene at ambient temperature.

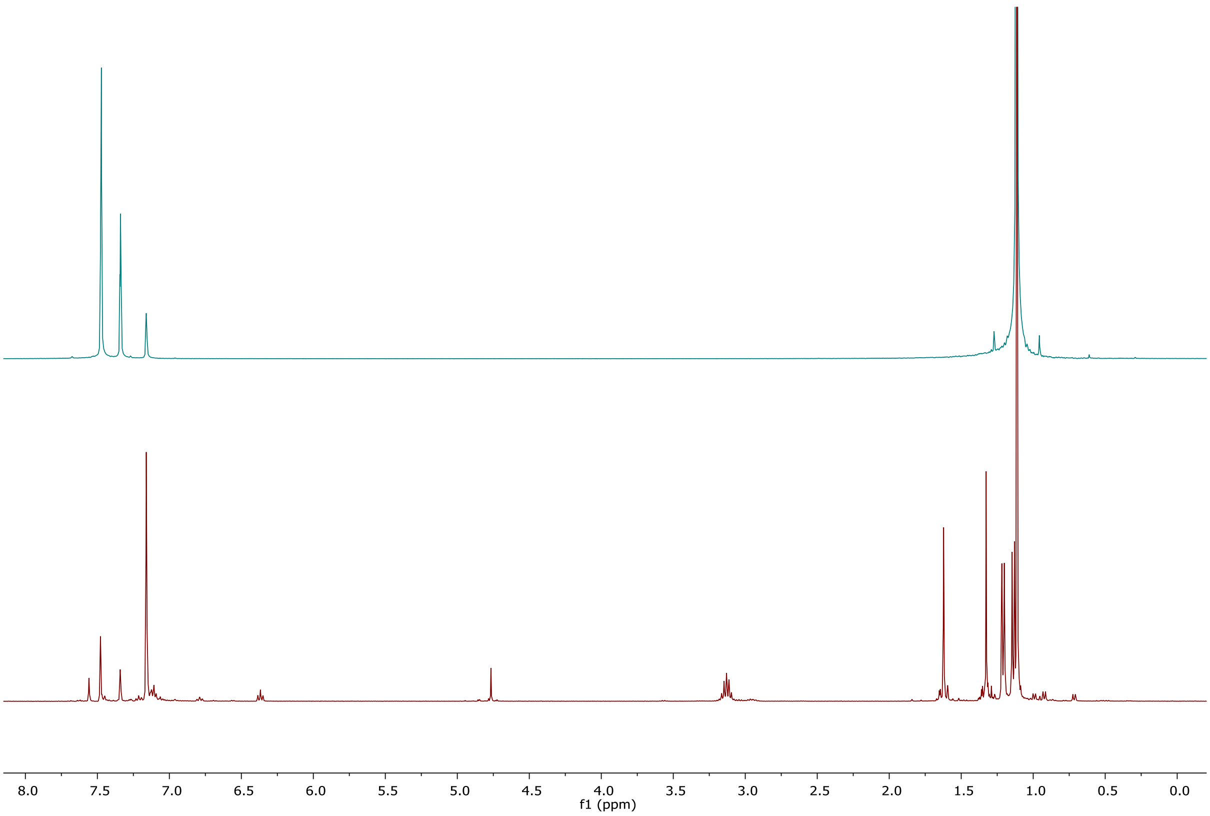

Figure S31. Stacked ^{1}H NMR spectra (C_6D_6, 298 K, 400.13 MHz) of the resulting spectrum from the reaction between **10** and 1-bromo-3,5-di-tert-butylbenzene at ambient temperature, with 1-bromo-3,5-di-tert-butylbenzene.

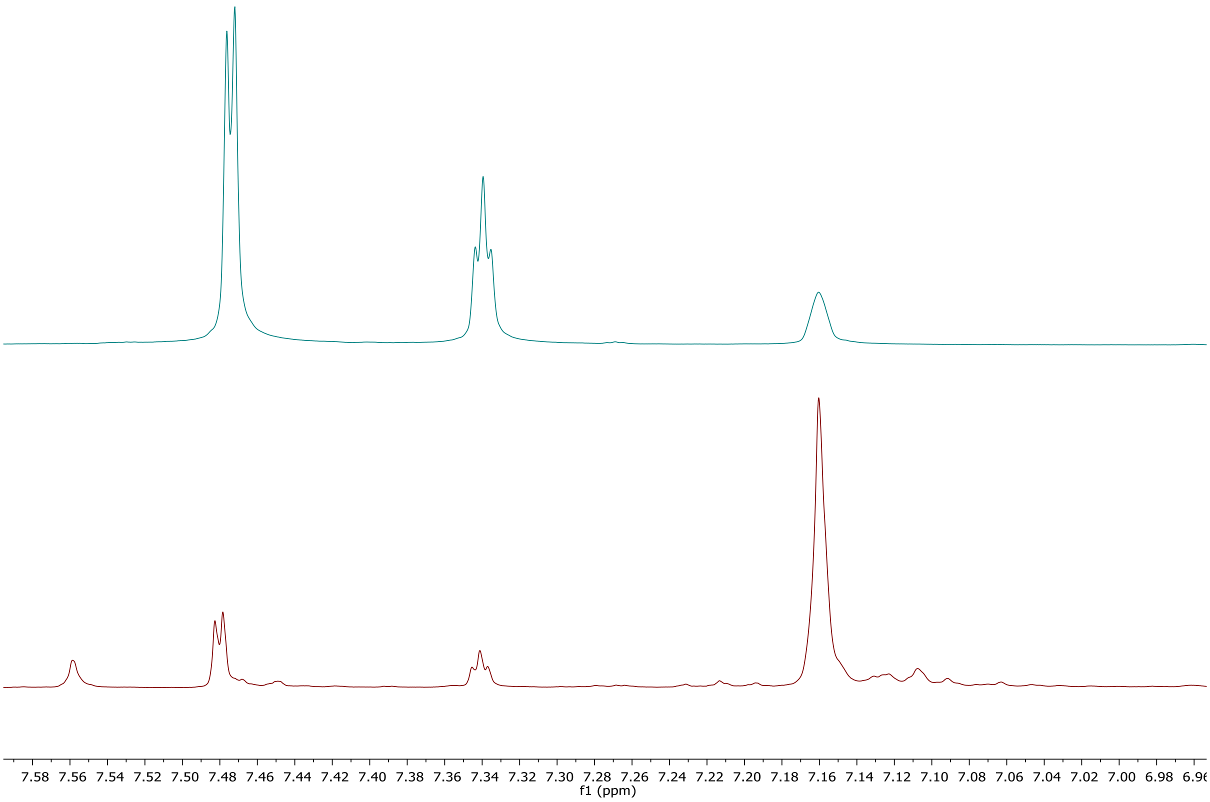

Figure S32. Expansion of part of the aromatic region shown in **Figure S31**, highlighting the presence of residual 1-bromo-3,5-di-tert-butylbenzene in the reaction mixture.

Reaction of 10 with 4-bromotoluene

4-Bromotoluene (0.5 mg, 0.003 mmol) was added to a C_6D_6 solution of **10** (3 mg, 0.003 mmol) and mixed at ambient temperature for 240 hours, resulting in a yellow solution. Conversions were estimated by ^{1}H NMR through comparison of the product peaks *versus* the methyl resonances of hexamethylbenzene, which was added as an internal standard, to be 33% (**11**) and 53% (4-methyl-biphenyl). Performing this reaction at 60 °C generates the homoleptic calcium complex [(BDI)$_2$Ca].

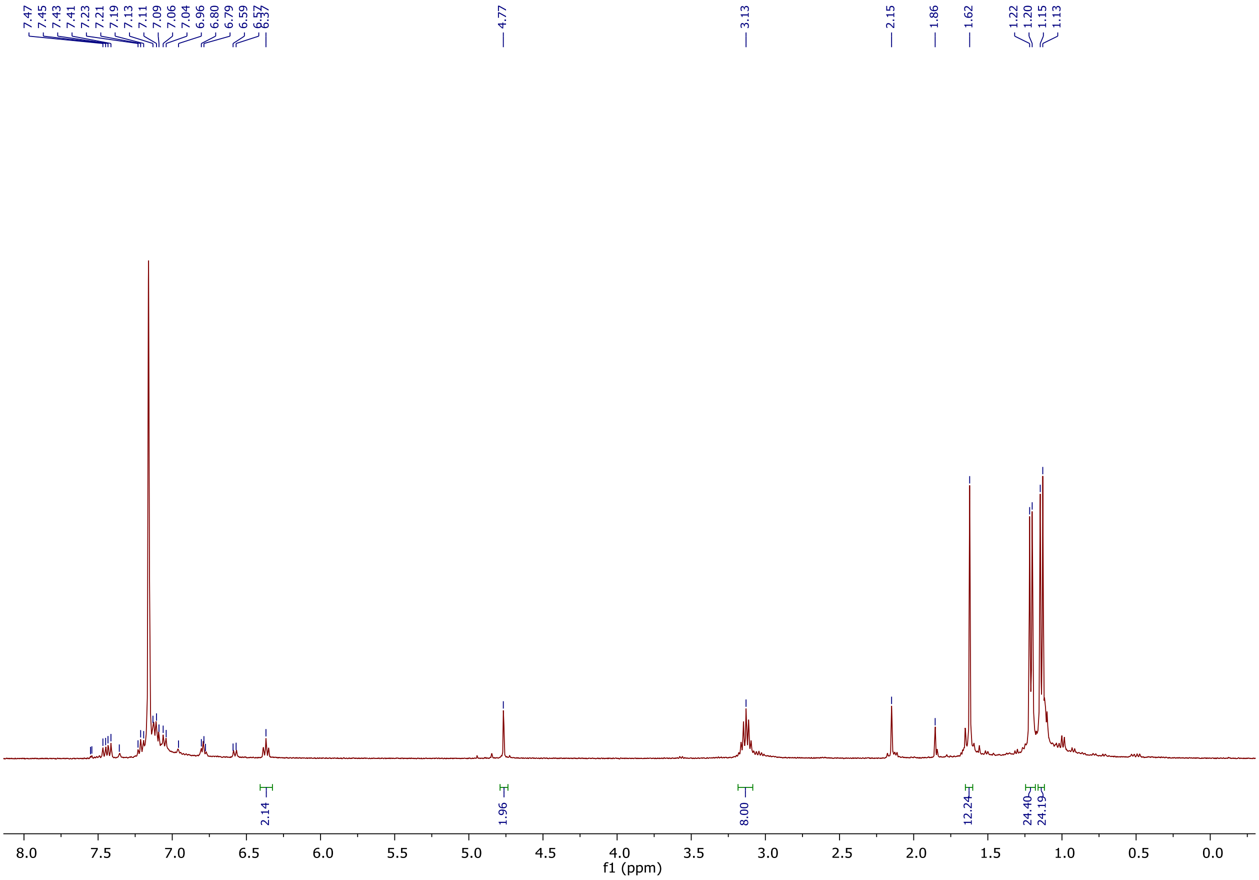

Figure S33. ^{1}H NMR spectrum (C_6D_6, 298 K, 400.13 MHz) resulting from the reaction of **10** and 4-bromotoluene for 240 hours at ambient temperature. The sample contains 4-bromotoluene, [(BDI)Ca(Br)PhCa(BDI)] (11) and 4-methylbiphenyl.

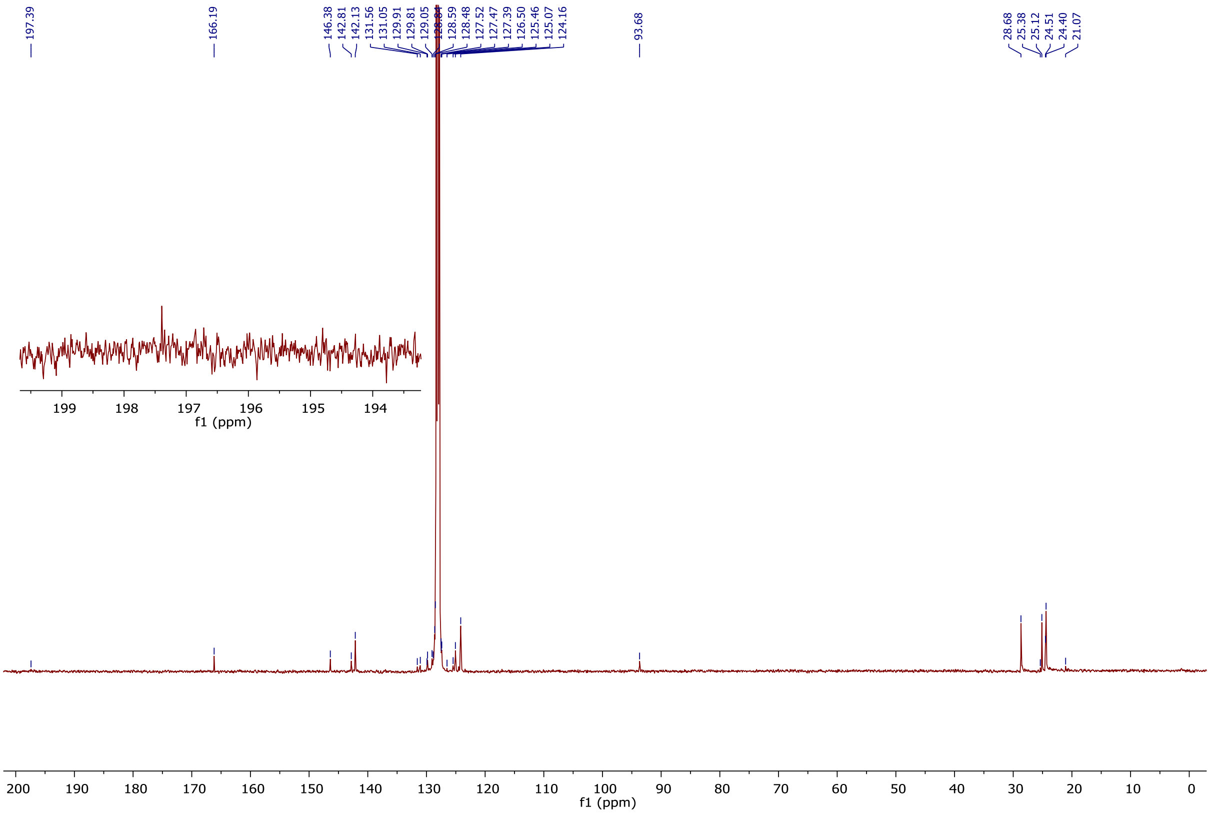

Figure S34. $^{13}C\{^{1}H\}$ NMR spectrum (C_6D_6, 298 K, 100.62 MHz) resulting from the reaction of **10** and 4-bromotoluene for 240 hours at ambient temperature. The sample contains 4-bromotoluene, [(BDI)Ca(Br)PhCa(BDI)] (**11**) and 4-methylbiphenyl.

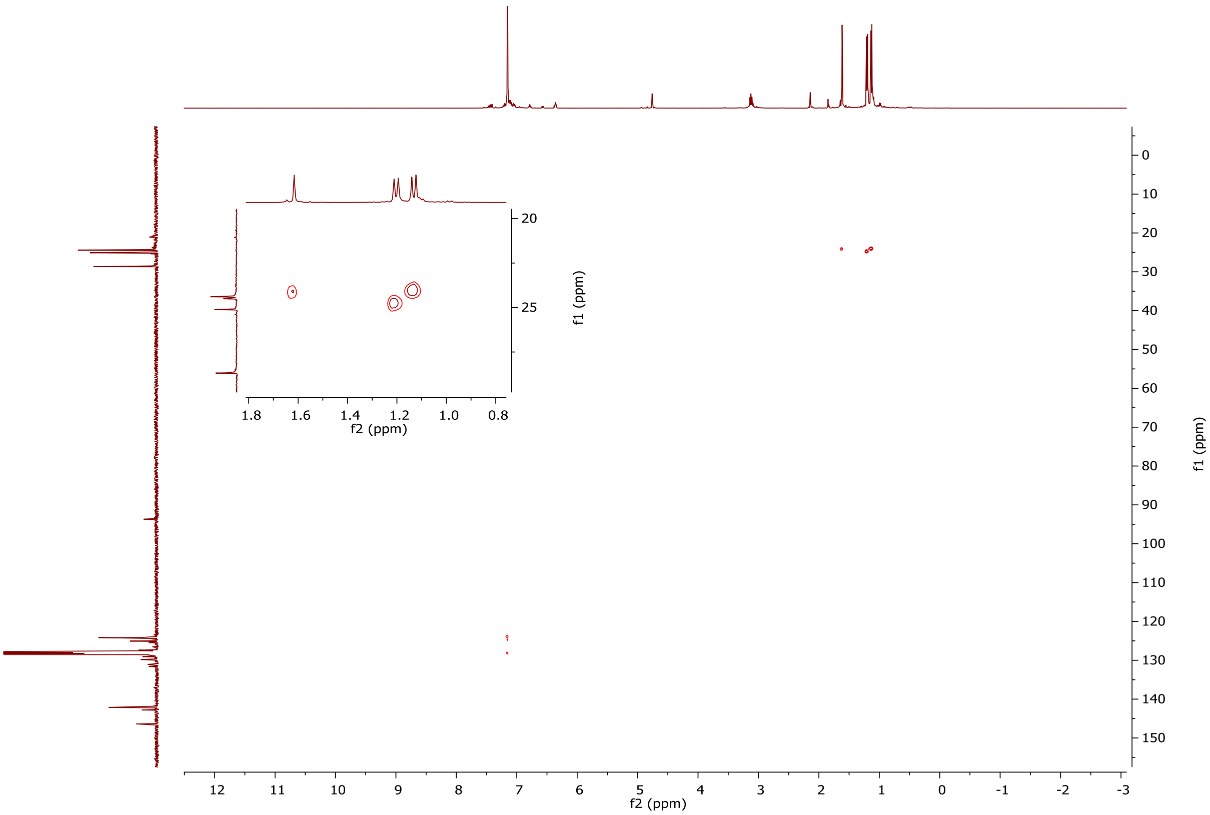

Figure S35. ^{1}H-^{13}C HSQC trace (C_6D_6, 298 K, 400.13, 100.62 MHz) from the reaction of **10** and 4-bromotoluene at ambient temperature.

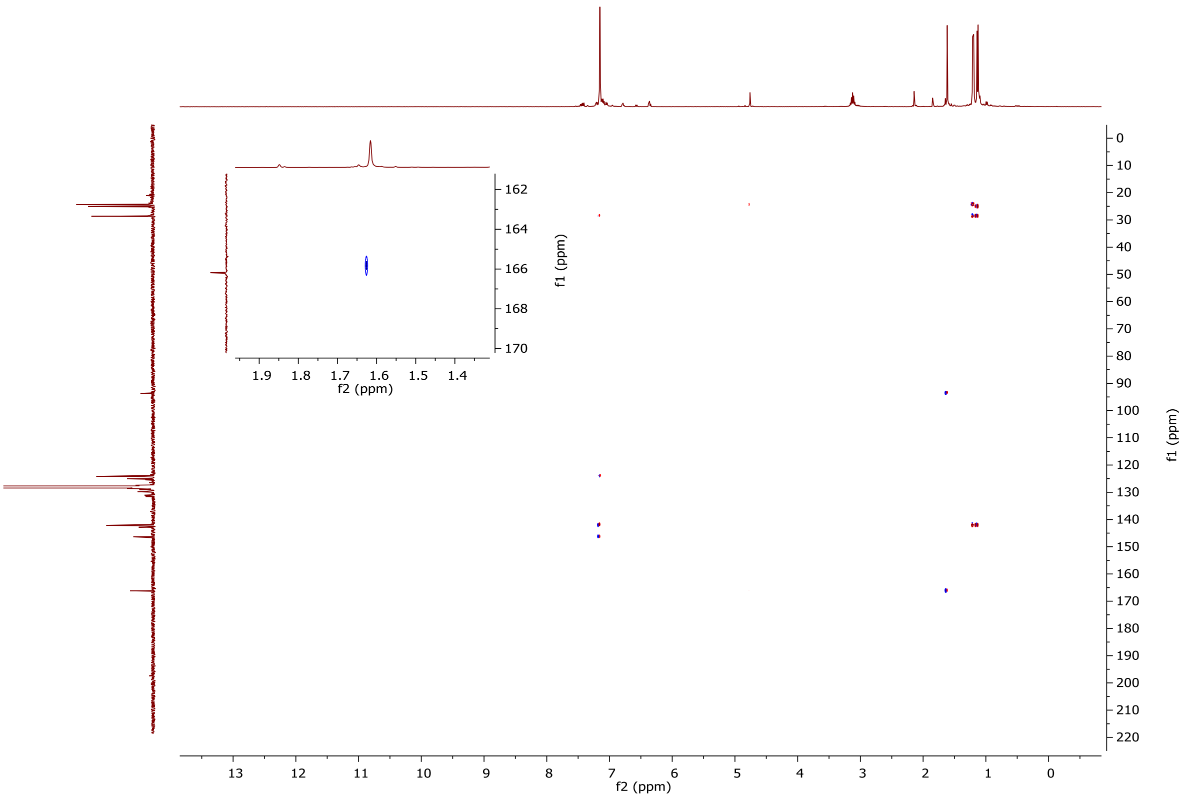

Figure S36. ^1H-^{13}C HMBC trace (C_6D_6, 298 K, 400.13, 100.62 MHz) from the reaction of **10** and 4-bromotoluene at ambient temperature.

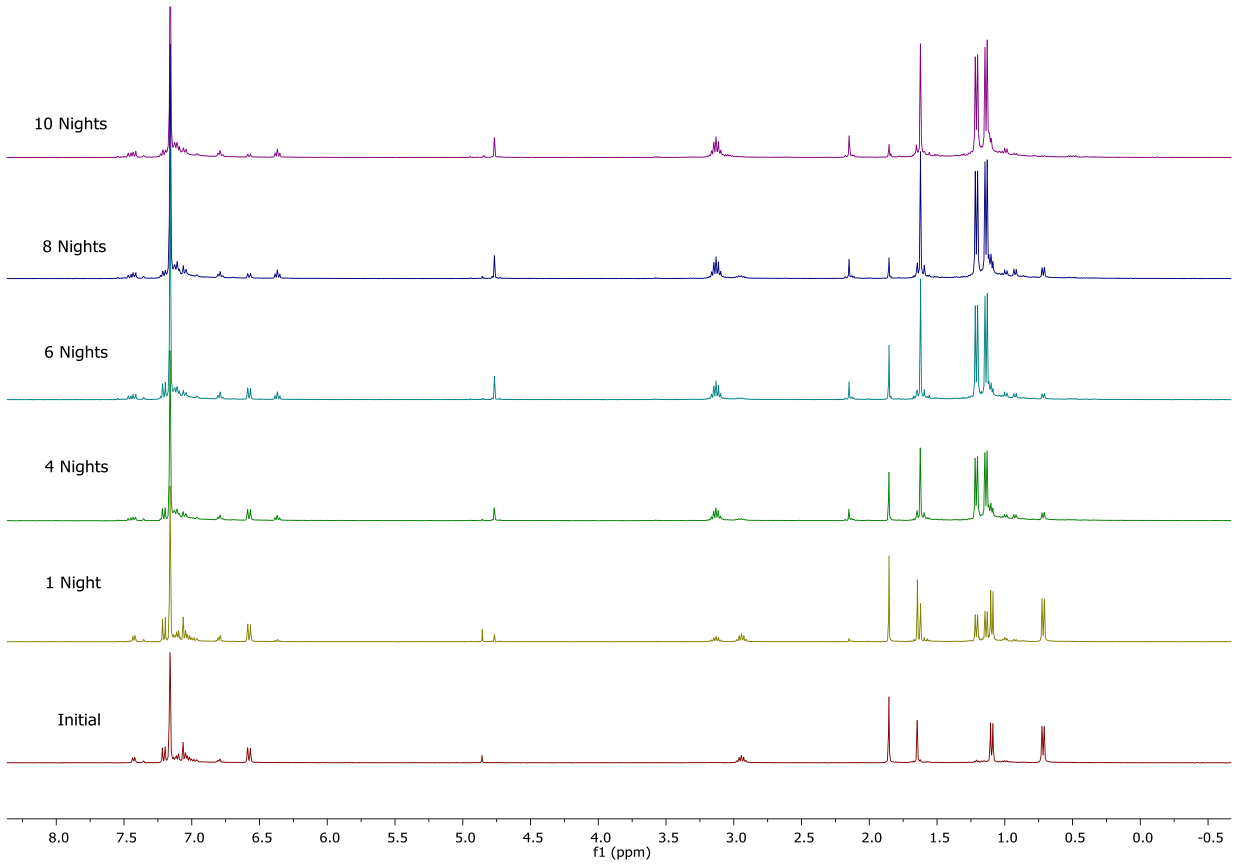

Figure S37. Stacked ^{1}H NMR spectra (C_6D_6, 298 K, 400.13 MHz) of the reaction between **10** and 4-bromotoluene at ambient temperature.

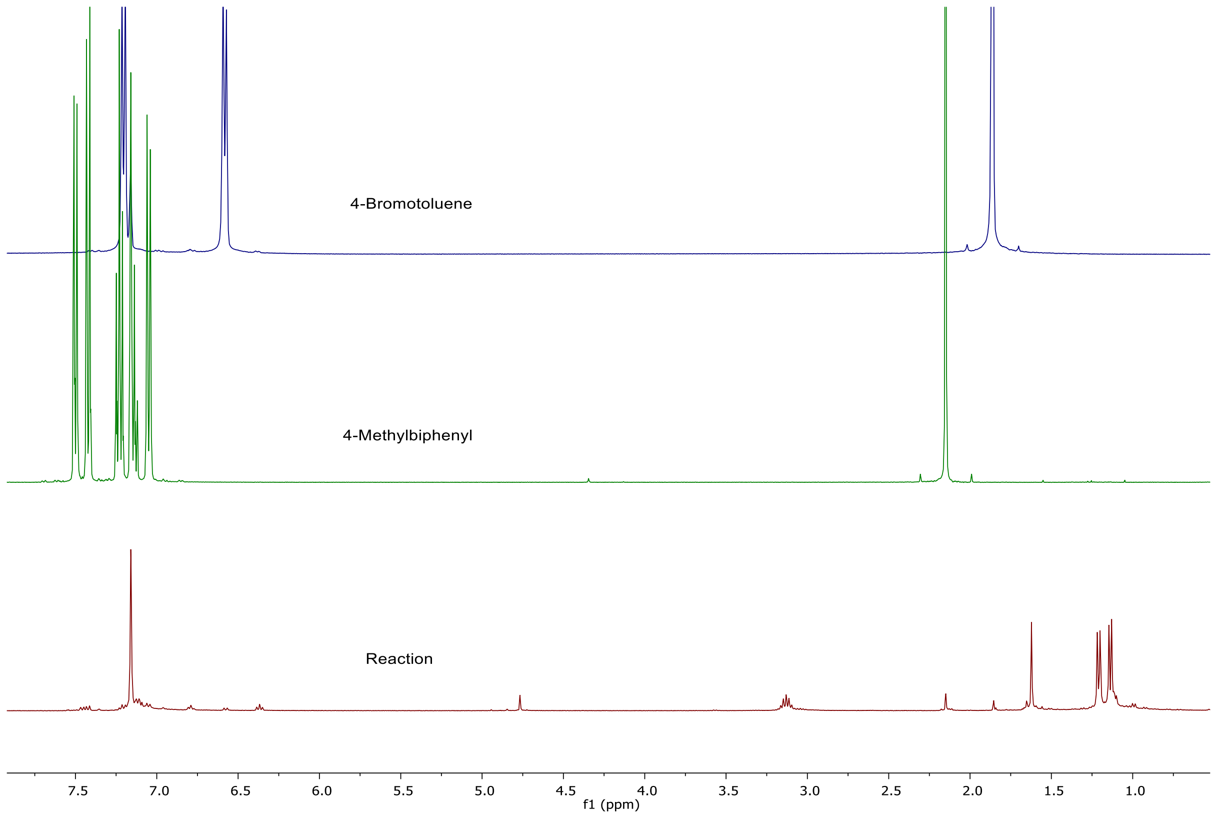

Figure S38. Stacked ^{1}H NMR spectra (C_6D_6, 298 K, 400.13 MHz) of the resulting spectrum from the reaction between **10** and 4-bromotoluene at ambient temperature, with 4-bromotoluene and 4-methylbiphenyl

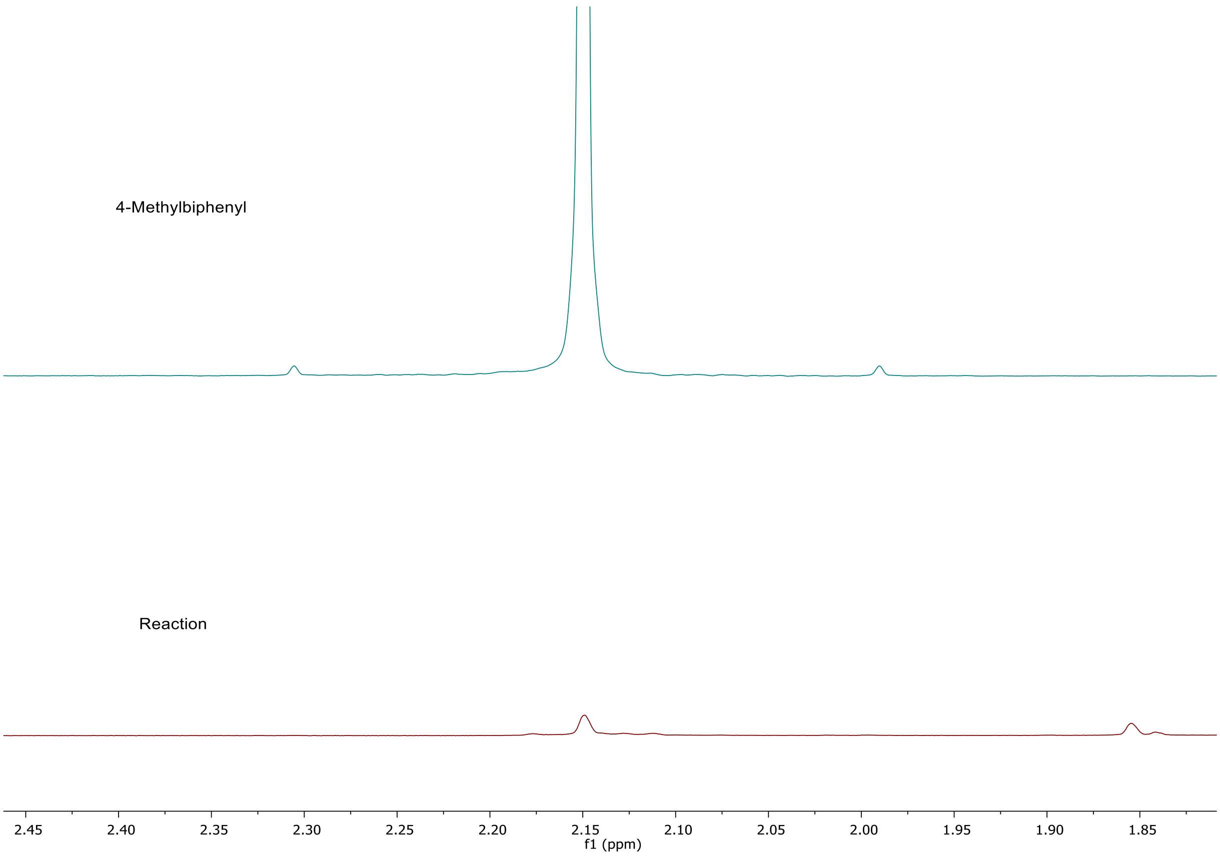

Figure S39. Expansion of methyl resonance for 4-methylbiphenyl, highlighting the presence of 4-methylbiphenyl in **Figure S33**.

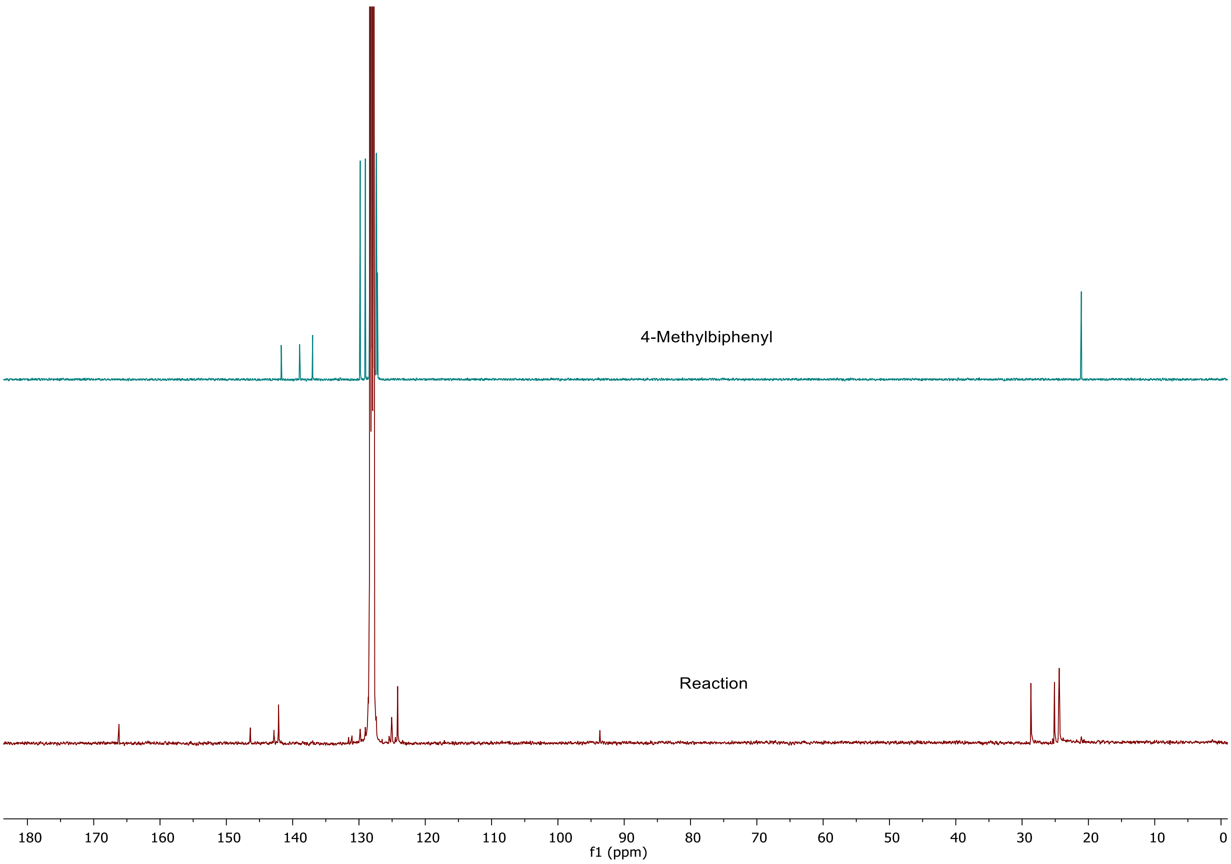

Figure S40. Stacked $^{13}C\{^{1}H\}$ NMR spectra (C_6D_6, 298 K, 400.13 MHz) of the resulting spectrum from the reaction between **10** and 4-bromotoluene at ambient temperature, with 4-methylbiphenyl.

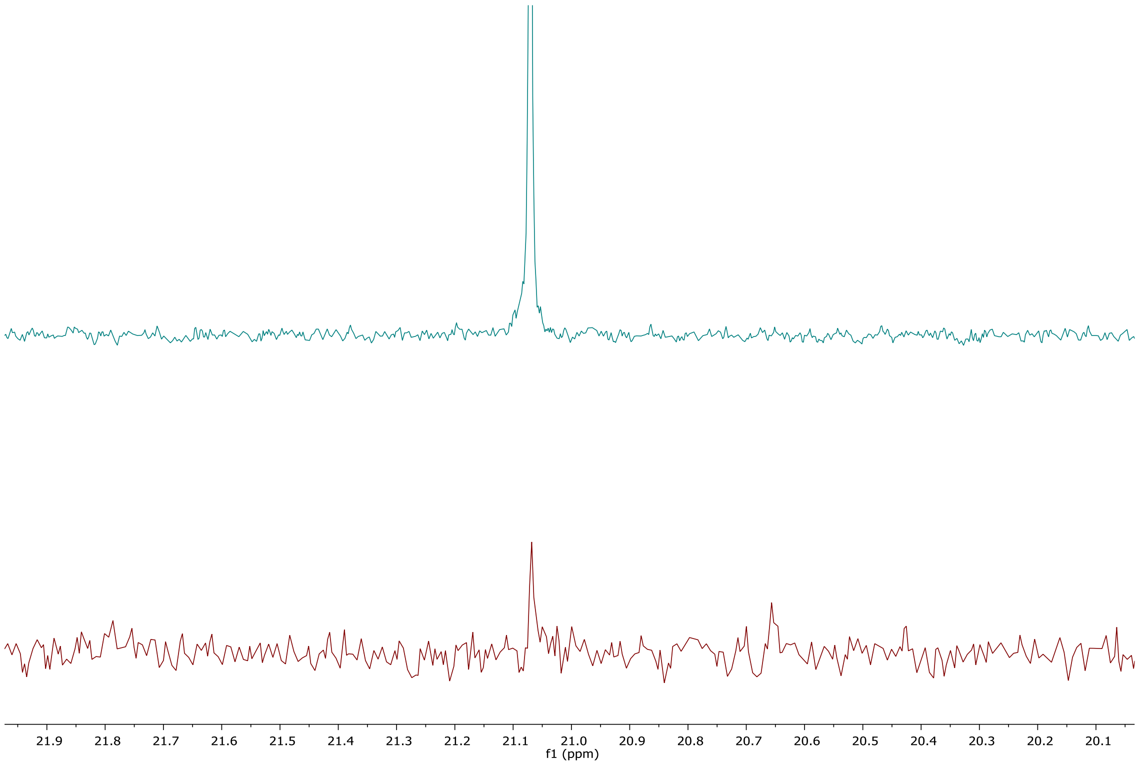

Figure S41. Expansion of methyl resonance in **Figure S40**, highlighting the presence of 4-methylbiphenyl in **Figure S34**.

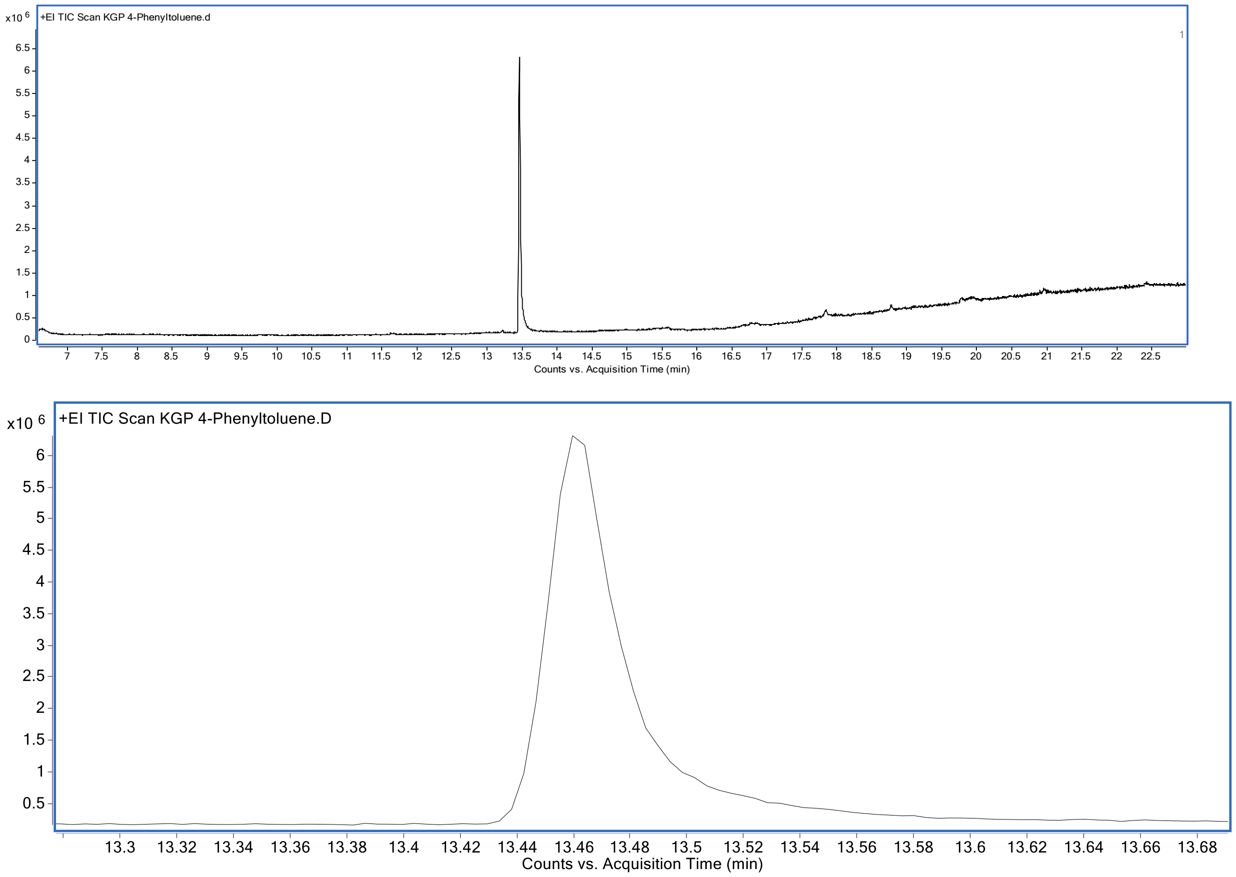

Figure S42. Reference GC-MS chromatogram of 4-methylbiphenyl. Top. Full chromatogram. Bottom. Expansion of the peak for 4-methylbiphenyl (13.46 min).

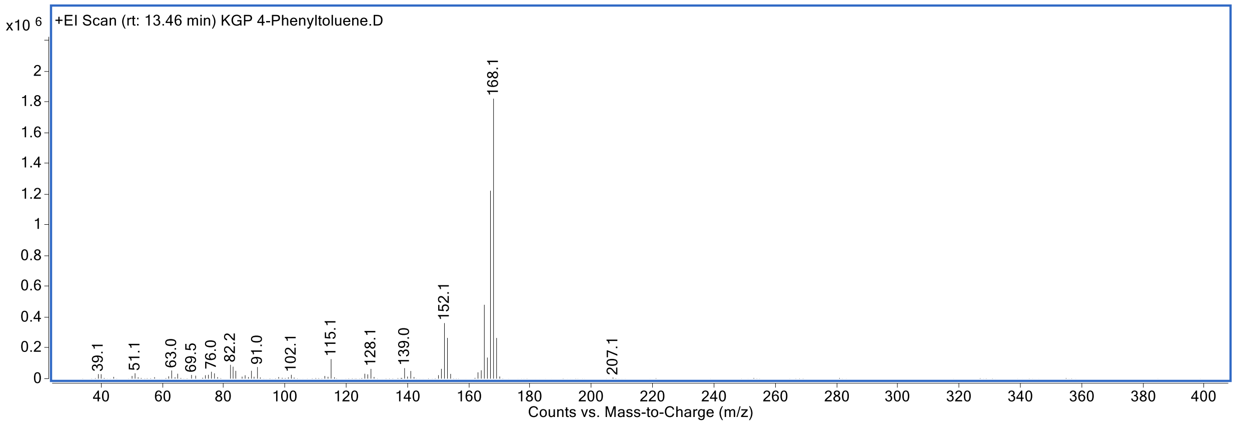

Figure S43. Reference GC-MS Trace showing the molecular ion of 4-methylbiphenyl.

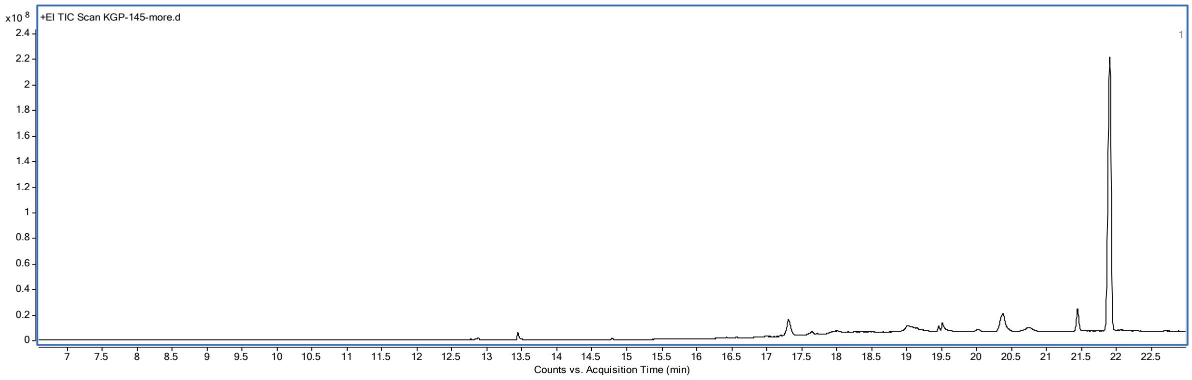

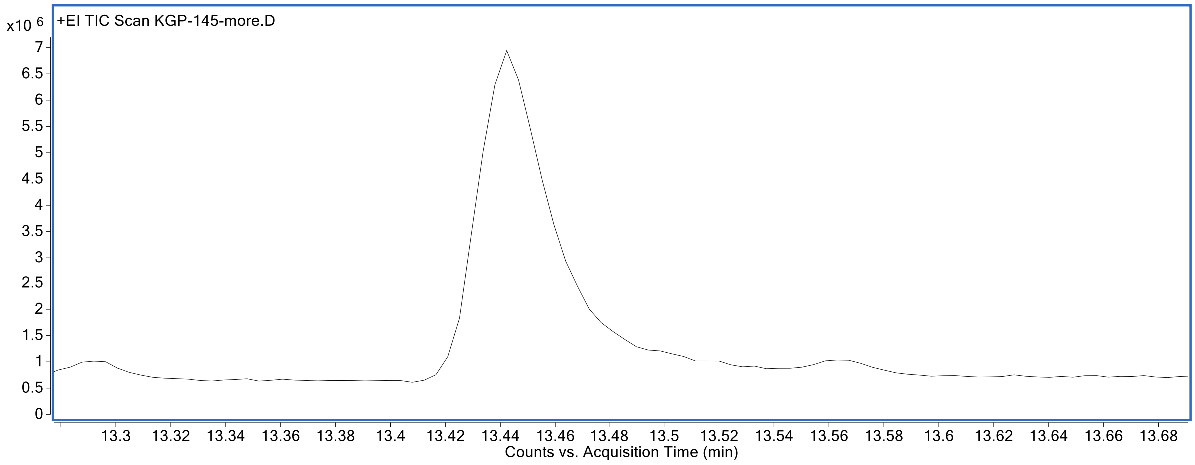

Figure S44. GC-MS chromatogram of the resulting spectra from the reaction between **10** and 4-bromotoluene at ambient temperature. Top. Full chromatogram. Bottom. Expansion of the peak for 4-methylbiphenyl (13.46 min).

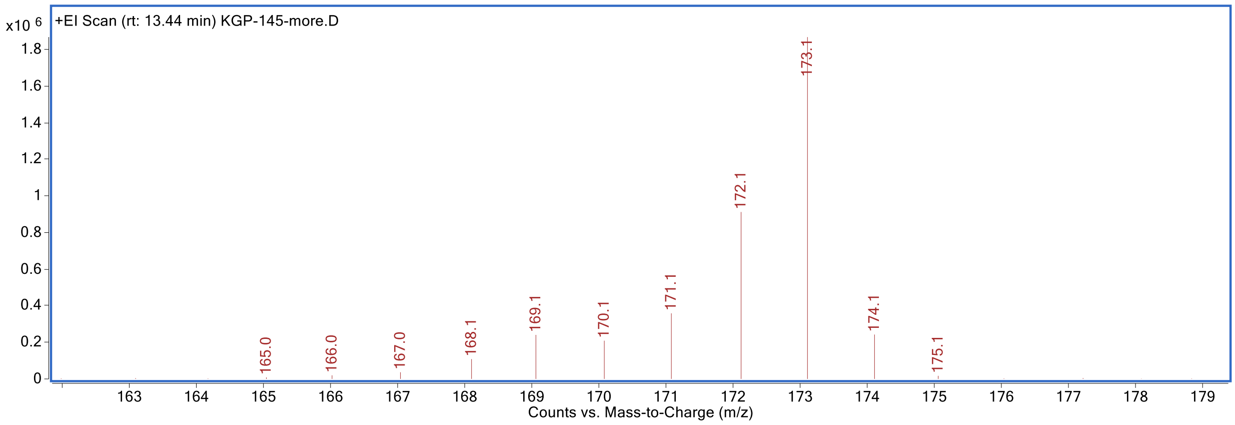

Figure S45. GC-MS trace **10** and 4-bromotoluene at ambient temperature, showing the molecular ion for 4-methylbiphenyl.

Reaction of 10 with 1-bromo-2-ethylbenzene

1-Bromo-2-ethylbenzene (0.67 μl, 0.005 mmol) was added to a C_6D_6 solution of **10** (5.2 mg, 0.005 mmol) and heated at 60 °C for 16 hours. Conversions were estimated by ^{1}H NMR through comparison

of the product peaks *versus* the methyl resonances of hexamethylbenzene, which was added as an internal standard, to be 45% (**11**) and 45% (2-ethyl-biphenyl).

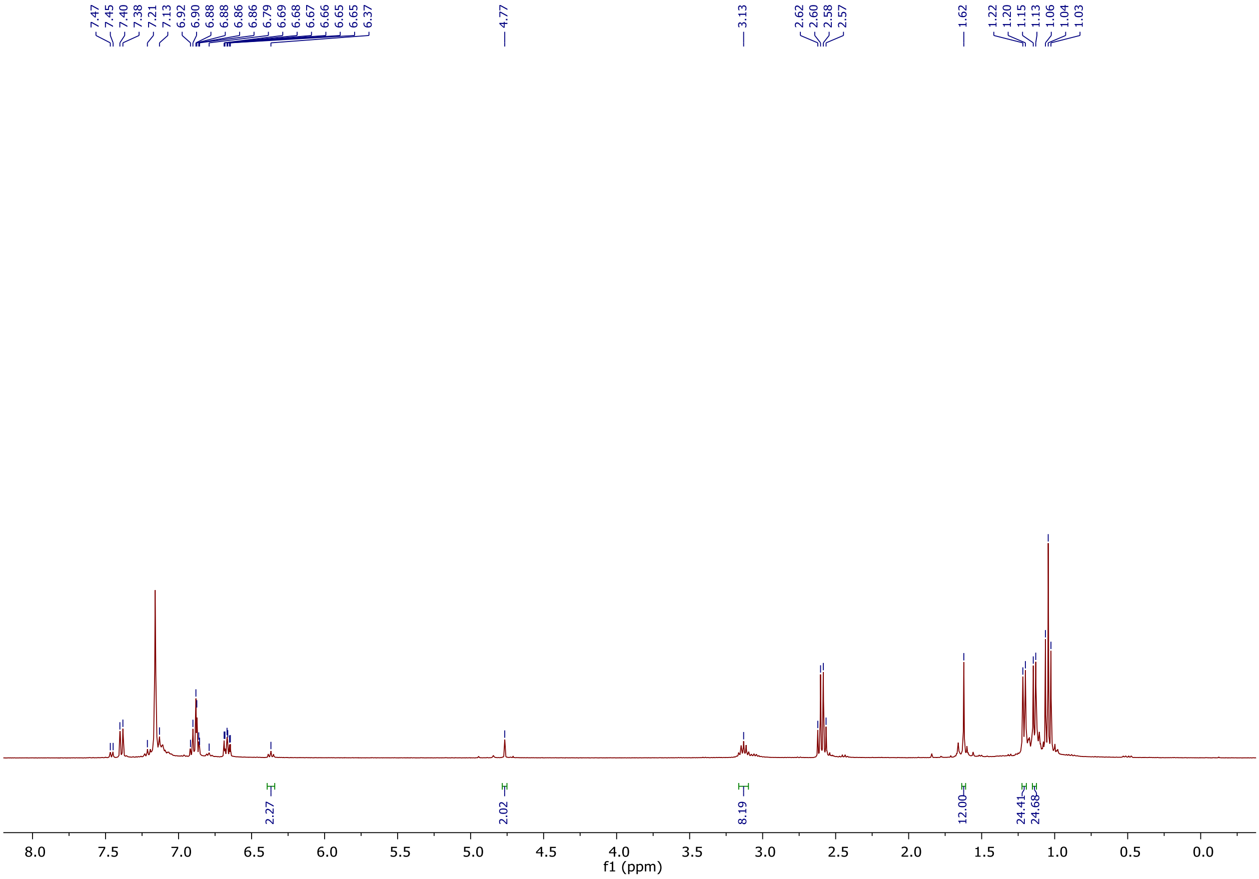

Figure S46. ^{1}H NMR spectrum (C_6D_6, 298 K, 400.13 MHz) resulting from the reaction of **10** and 1-bromo-2-ethylbenzene for 16 hours at 60 °C. The sample contains 1-bromo-2-ethylbenzene, [(BDI)Ca(Br)PhCa(BDI)] (**11**) and 2-ethylbiphenyl.

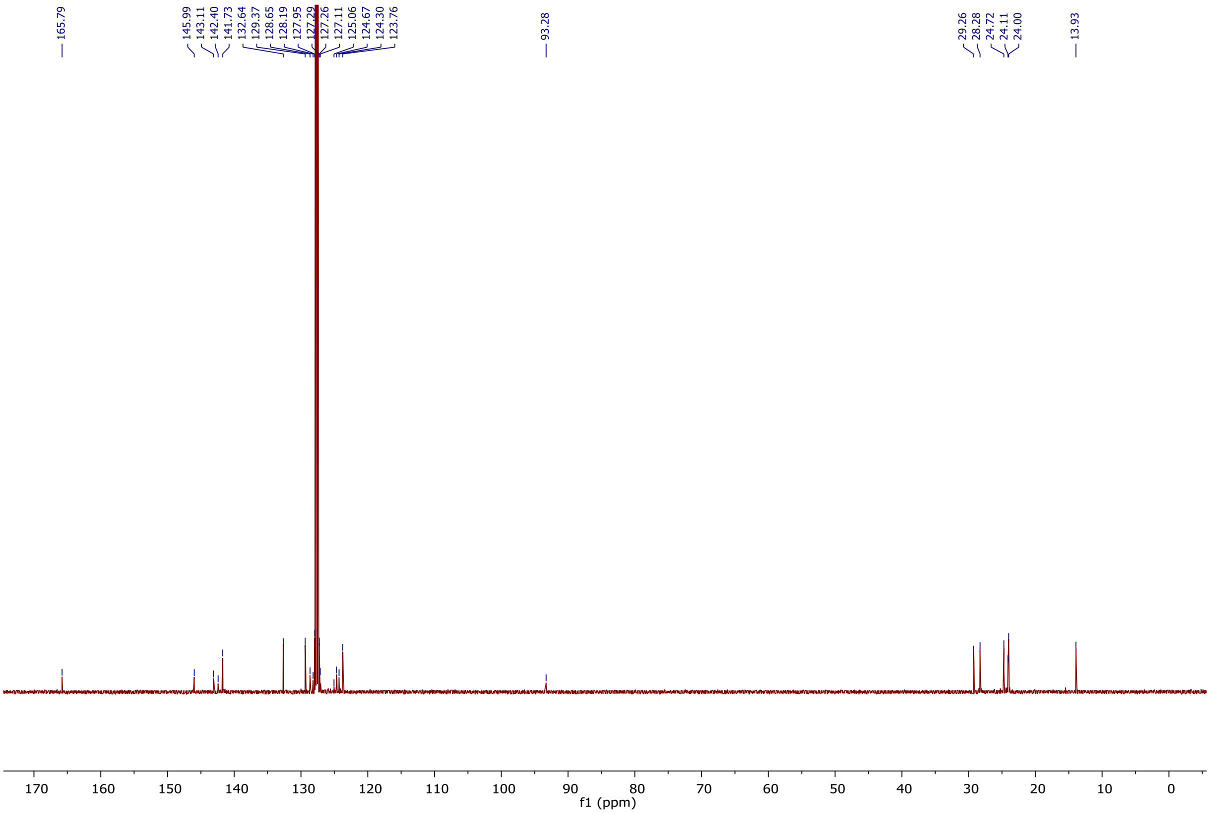

Figure S47. $^{13}C\{^{1}H\}$ NMR spectrum (C_6D_6, 298 K, 100.62 MHz) resulting from the reaction of **10** and 1-bromo-2-ethylbenzene for 16 hours at 60 °C. The sample contains 1-bromo-2-ethylbenzene, [(BDI)Ca(Br)PhCa(BDI)] (**11**) and 2-ethylbiphenyl.

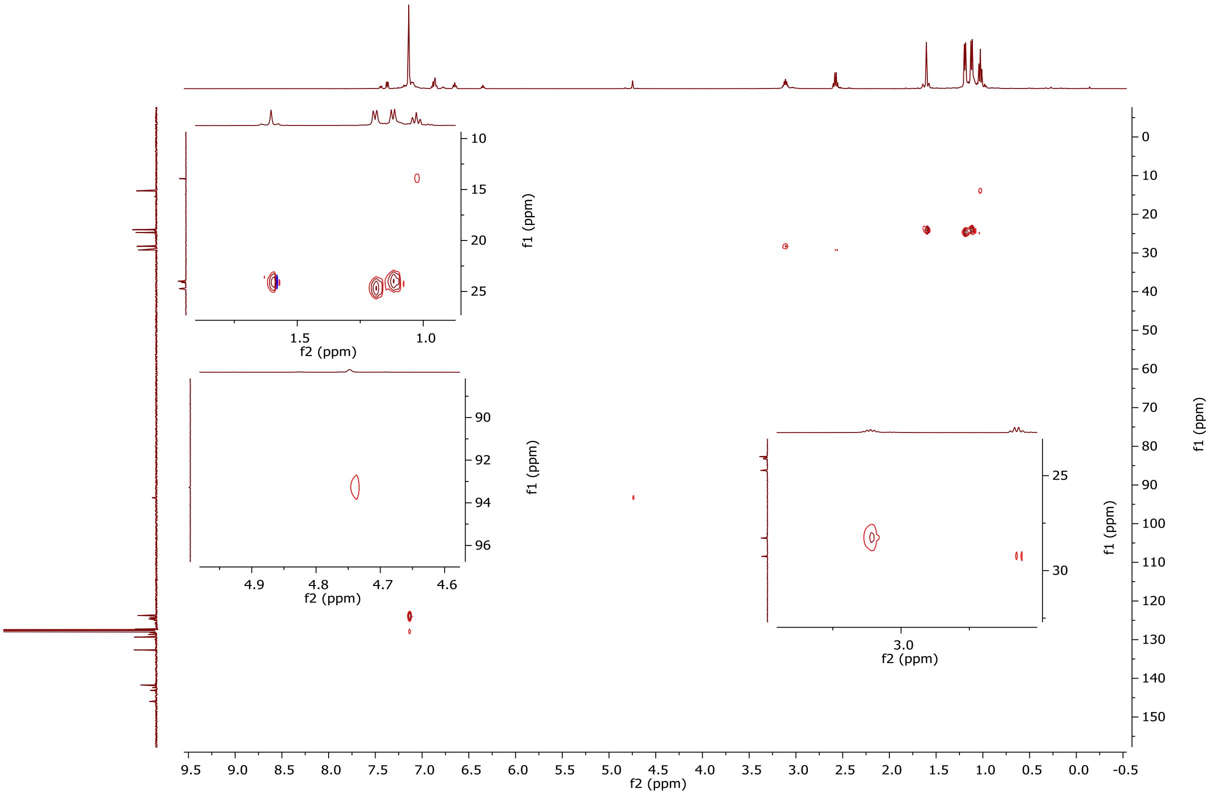

Figure S48. ^{1}H-^{13}C HSQC trace (C_6D_6, 298 K, 400.13, 100.62 MHz) from the reaction of **10** and 1-bromo-2-ethylbenzene for 16 hours at 60 °C.

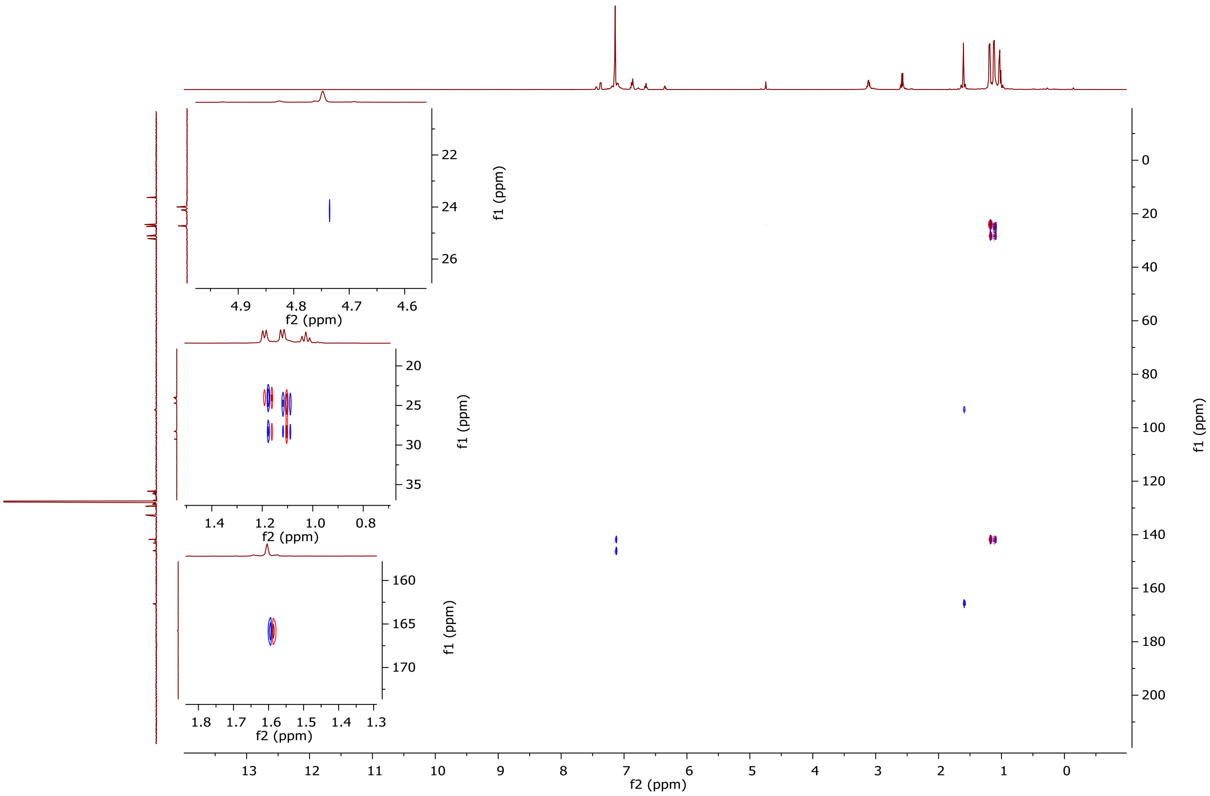

Figure S49. ^{1}H-^{13}C HMBC trace (C_6D_6, 298 K, 400.13, 100.62 MHz) from the reaction of **10** and 1-bromo-2-ethylbenzene for 16 hours at 60 °C.

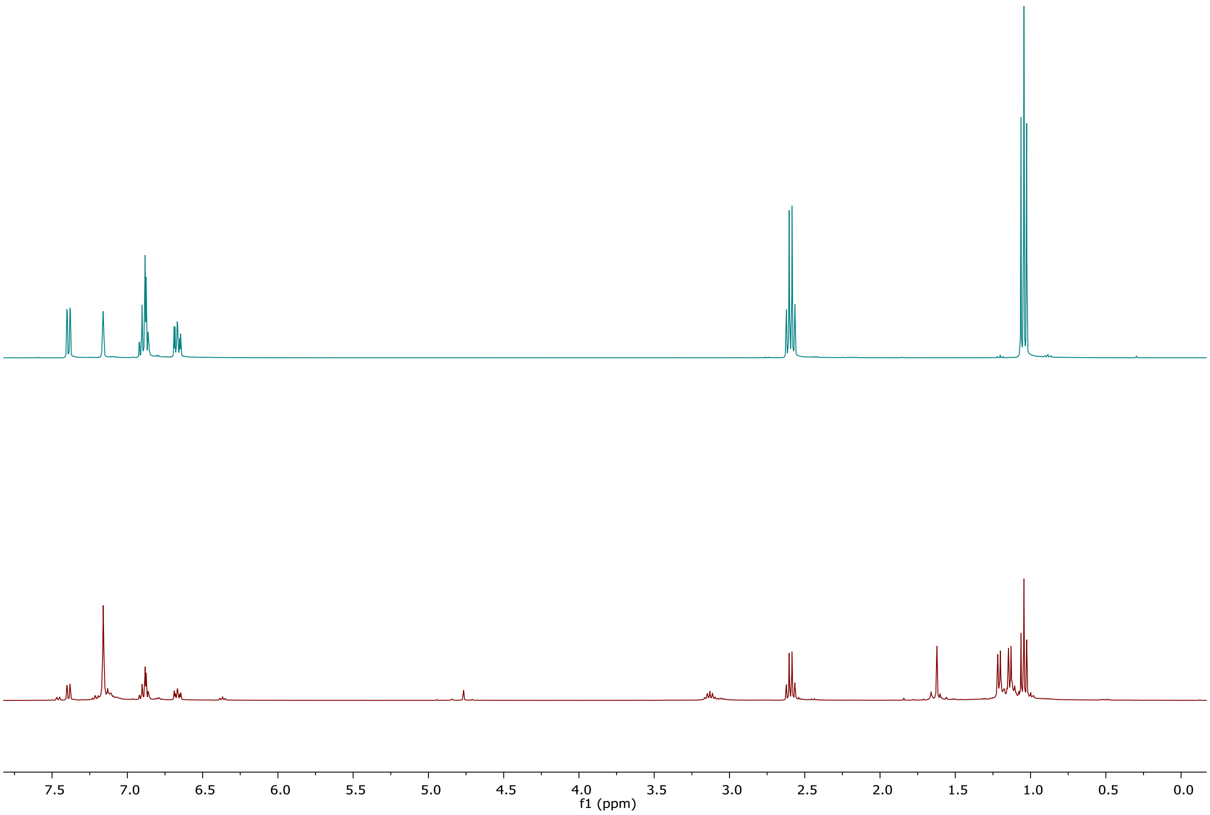

Figure S50. Stacked ^{1}H NMR spectra (C_6D_6, 298 K, 400.13 MHz) of the resulting spectrum from the reaction between **10** and 1-bromo-2-ethylbenzene, with 1-bromo-2-ethylbenzene, highlighting the residual 1-bromo-2-ethylbenzene present.

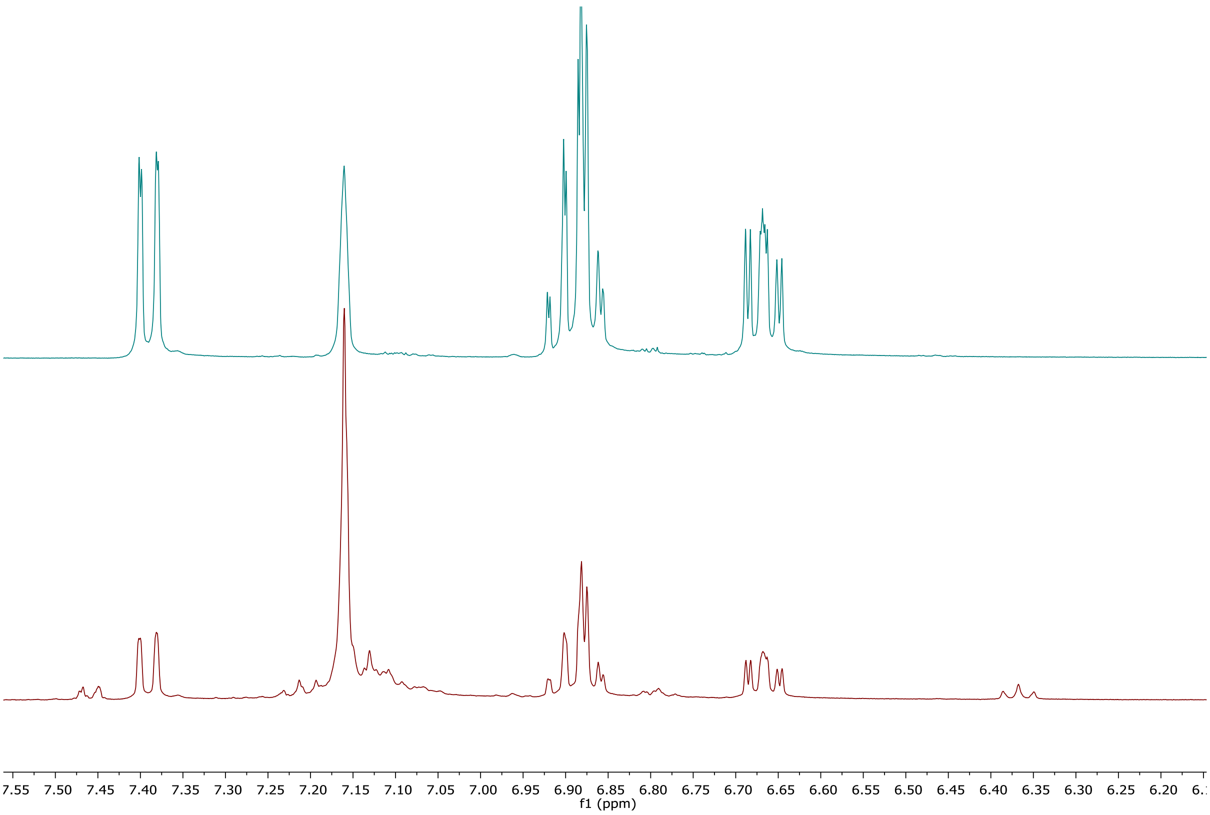

Figure S51. Expansion of part of the aromatic region shown in **Figure S50** with 1-bromo-2-ethylbenzene, highlighting the presence of residual 1-bromo-2-ethylbenzene in the reaction mixture.

Reaction of 10 with 2-bromobiphenyl

2-Bromobiphenyl (2.5 µl, 0.015 mmol) was added to a C_6D_6 solution of **10** (15.5 mg, 0.015 mmol) and mixed at ambient temperature for 360 hours, resulting in an orange solution. Alternatively, this reaction can be performed in 16 hours at 60 °C. Conversions were estimated by ^{1}H NMR through comparison of the product peaks *versus* the methyl resonances of hexamethylbenzene, which was added as an internal standard, to be 62% (**11**) and 31% (*o*-terphenyl).

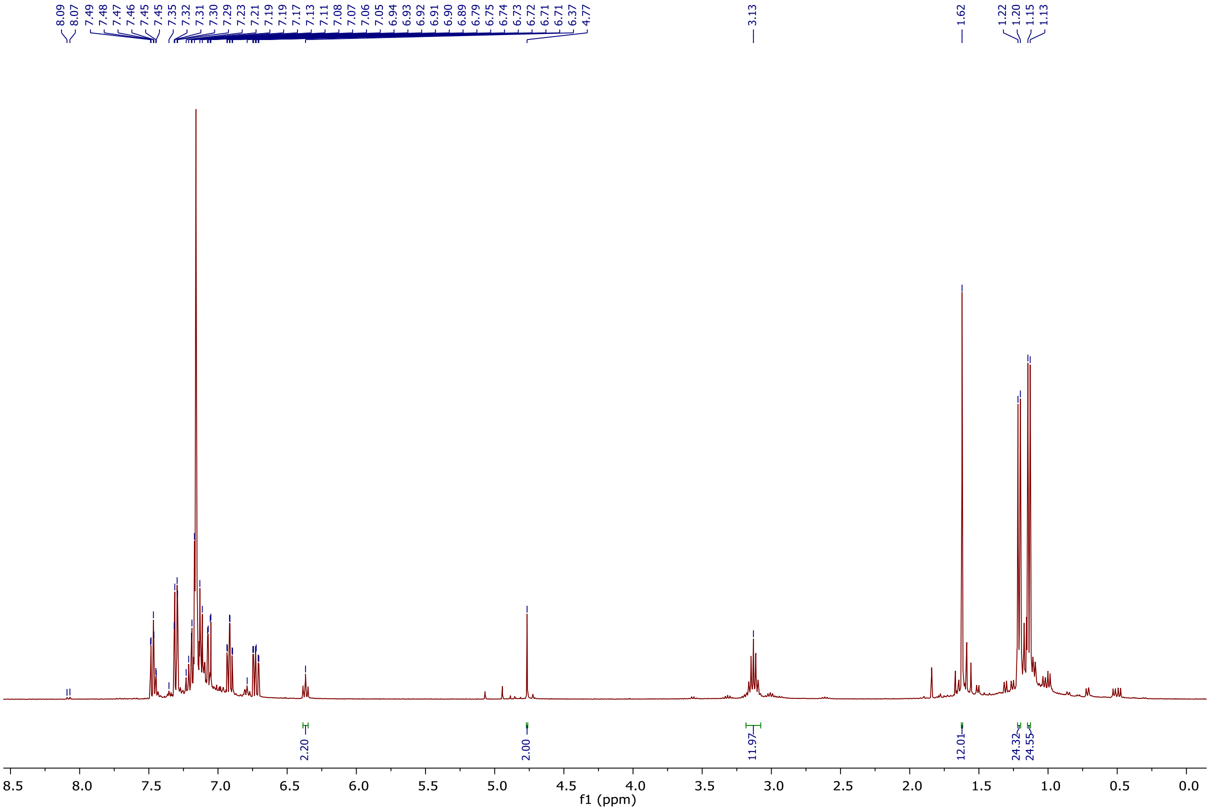

Figure S52. ^{1}H NMR spectrum (C_6D_6, 298 K, 400.13 MHz) resulting from the reaction of **10** and 2-bromobiphenyl for 360 hours at ambient temperature. The sample contains 2-bromobiphenyl, [(BDI)Ca(Br)PhCa(BDI)] (**11**) and o-terphenyl.

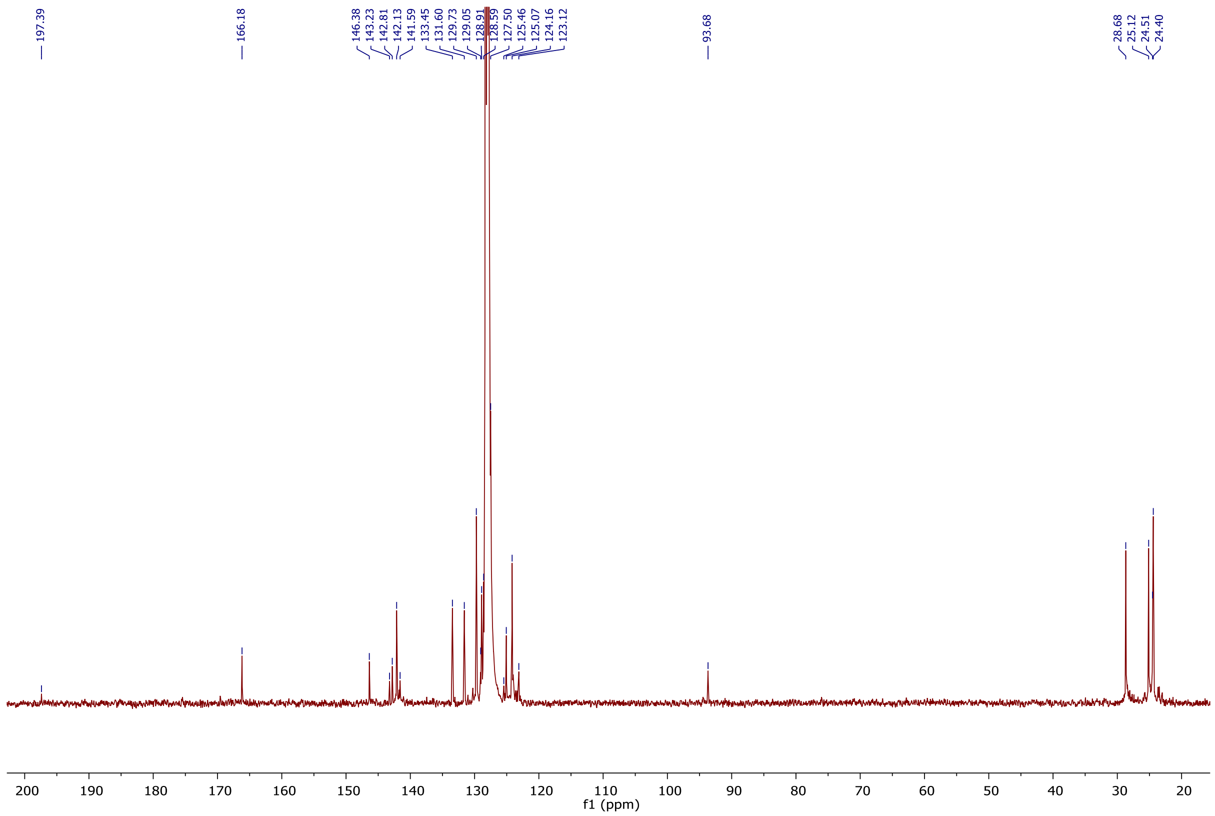

Figure S53. $^{13}C\{^{1}H\}$ NMR spectrum (C_6D_6, 298 K, 400.13 MHz) resulting from the reaction of **10** and 2-bromobiphenyl for 360 hours at ambient temperature. The sample contains 2-bromobiphenyl, [(BDI)Ca(Br)PhCa(BDI)] (**11**) and o-terphenyl.

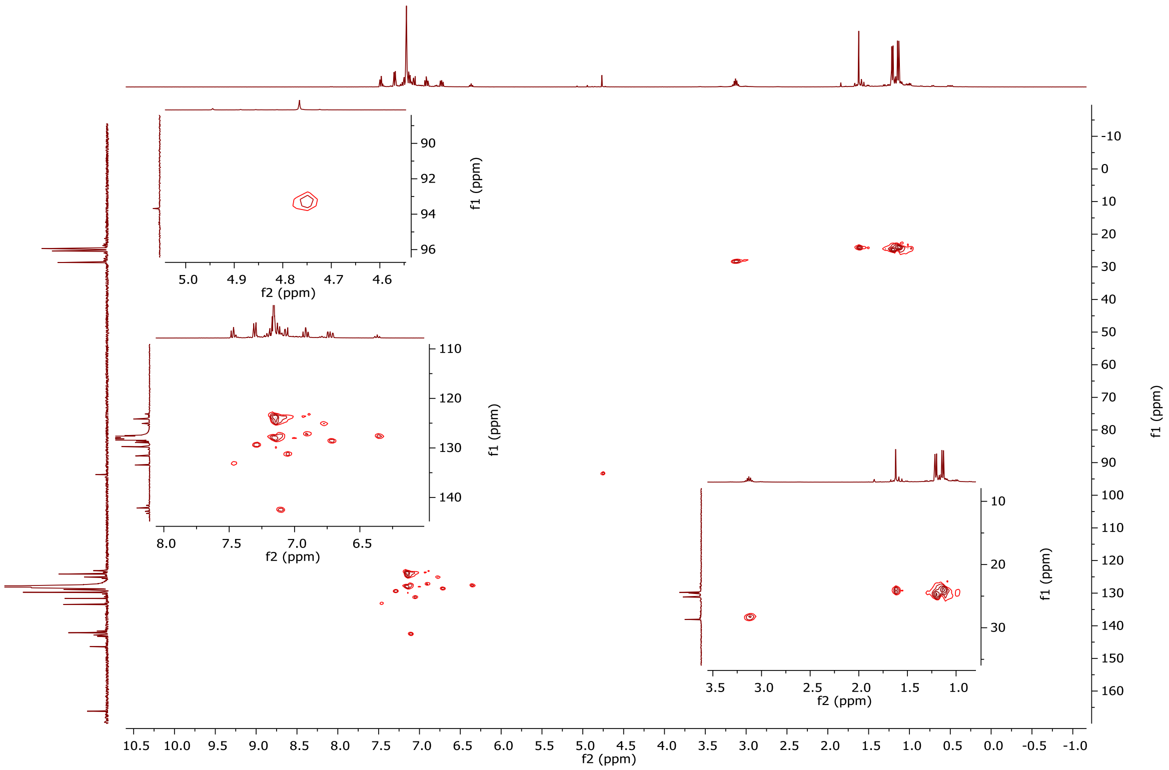

Figure S54. ^{1}H-^{13}C HSQC trace (C_6D_6, 298 K, 400.13, 100.62 MHz) resulting from the reaction of **10** and 2-bromobiphenyl for 360 hours at ambient temperature.

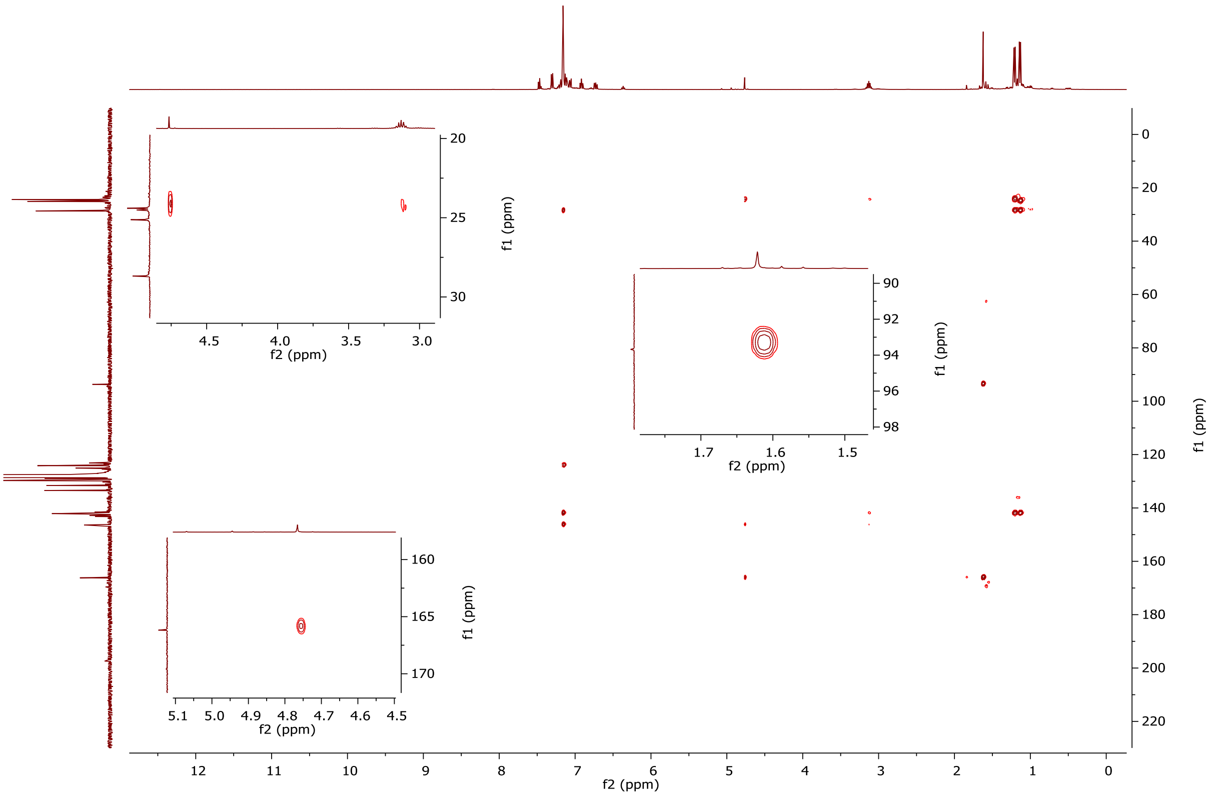

Figure S55. ^1H-^{13}C HSQC trace (C_6D_6, 298 K, 400.13, 100.62 MHz) resulting from the reaction of **10** and 2-bromobiphenyl for 360 hours at ambient temperature.

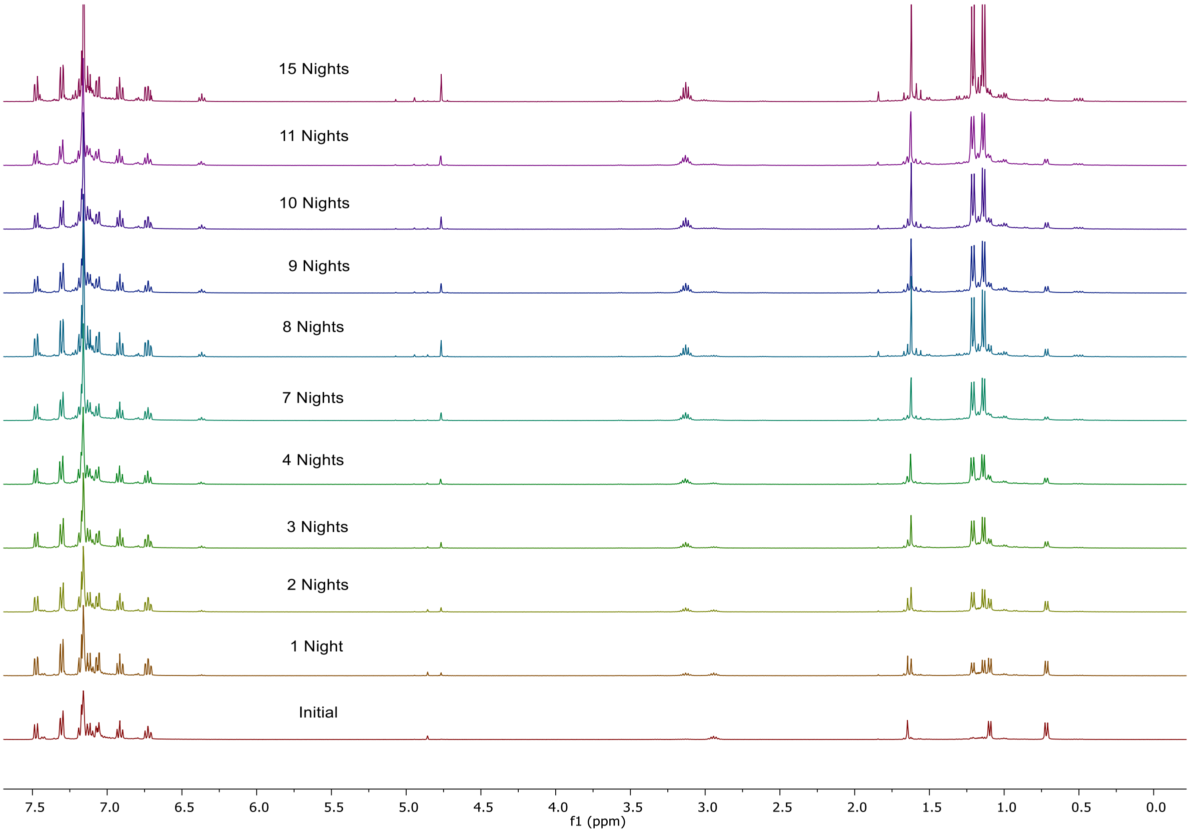

Figure S56. Stacked ^1H NMR spectra (C_6D_6, 298 K, 400.13 MHz) of the reaction between **10** and 2-bromobiphenyl at ambient temperature.

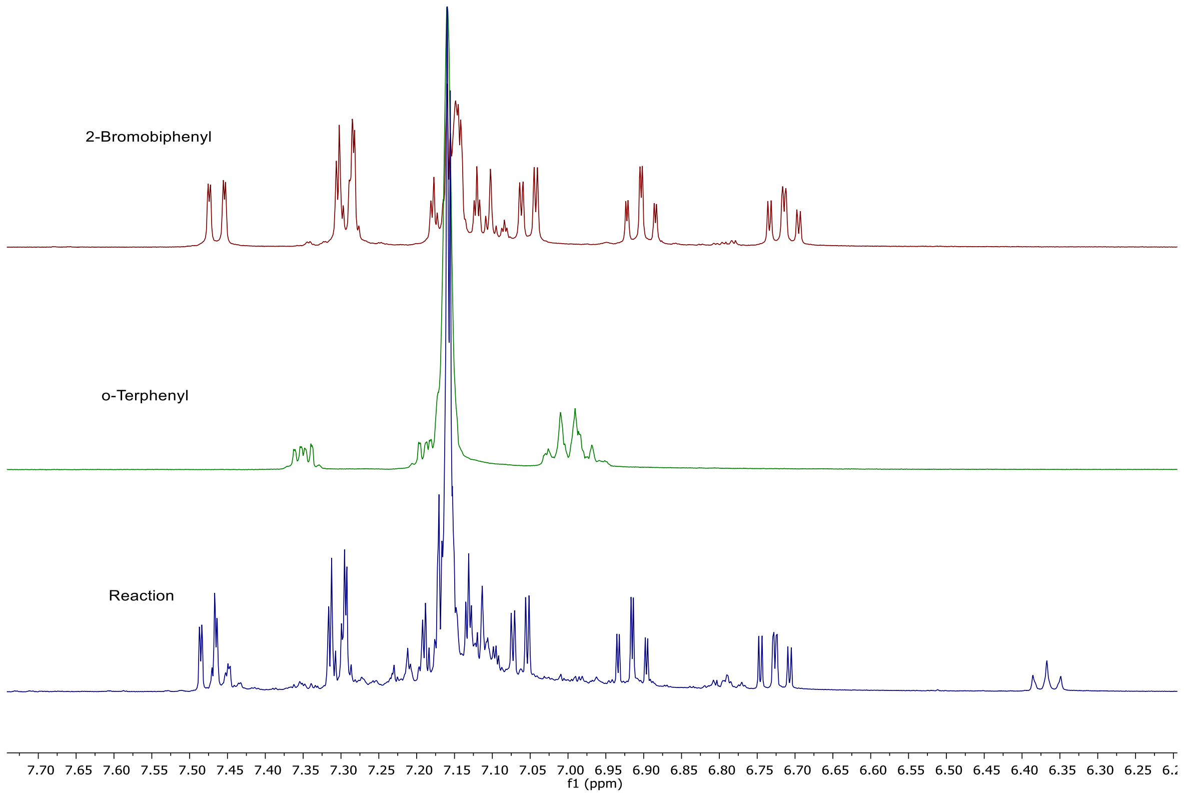

Figure S57. Stacked ^{1}H NMR spectra (C_6D_6, 298 K, 400.13 MHz) of the resulting spectrum from the reaction between **10** and 2-bromobiphenyl at ambient temperature, with 2-bromobiphenyl and o-terphenyl.

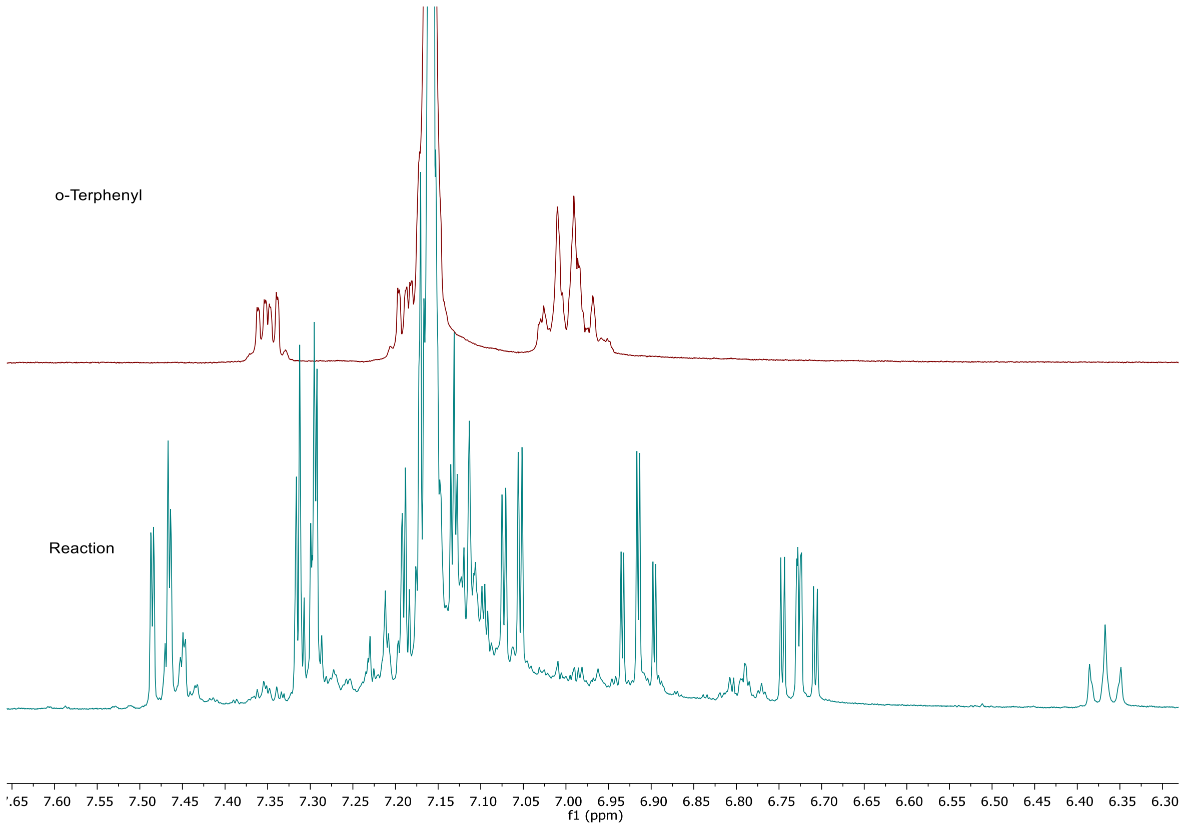

Figure S58. Expansion of the aromatic region shown in **Figure S52** with o-terphenyl, highlighting the presence of o-terphenyl.

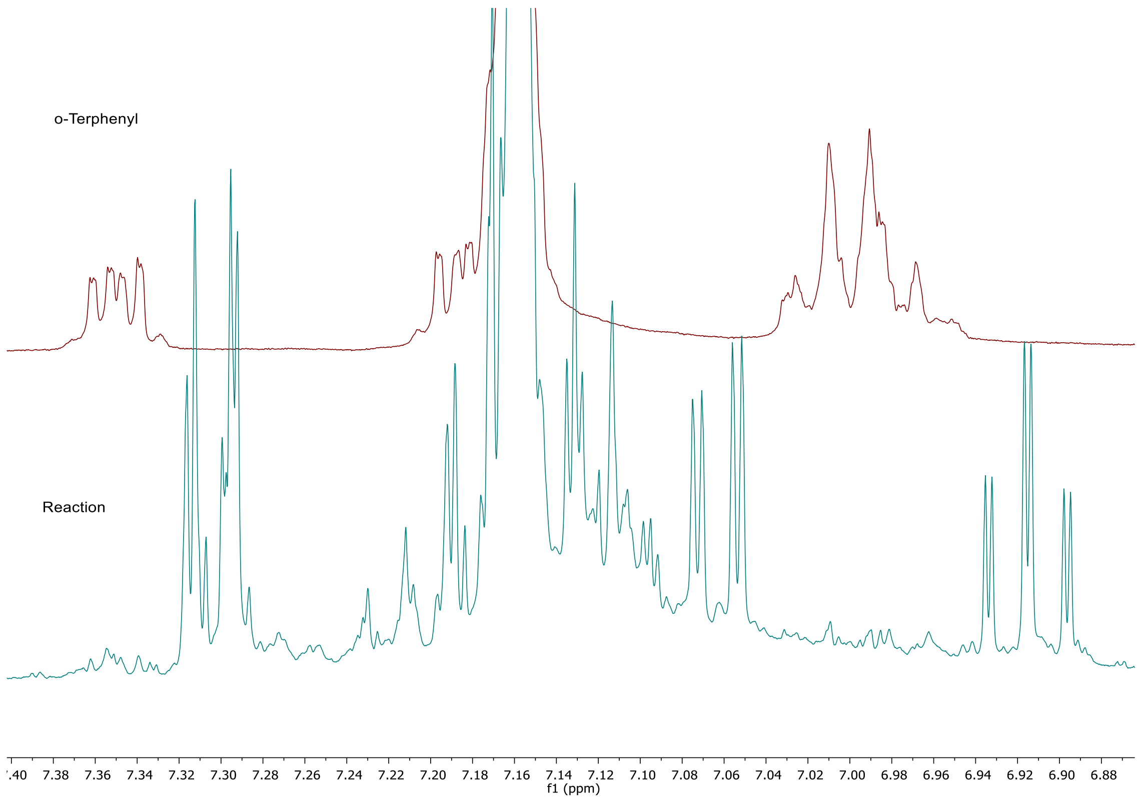

Figure S59. Expansion of the aromatic region shown in **Figure S58** with o-terphenyl, highlighting the presence of o-terphenyl.

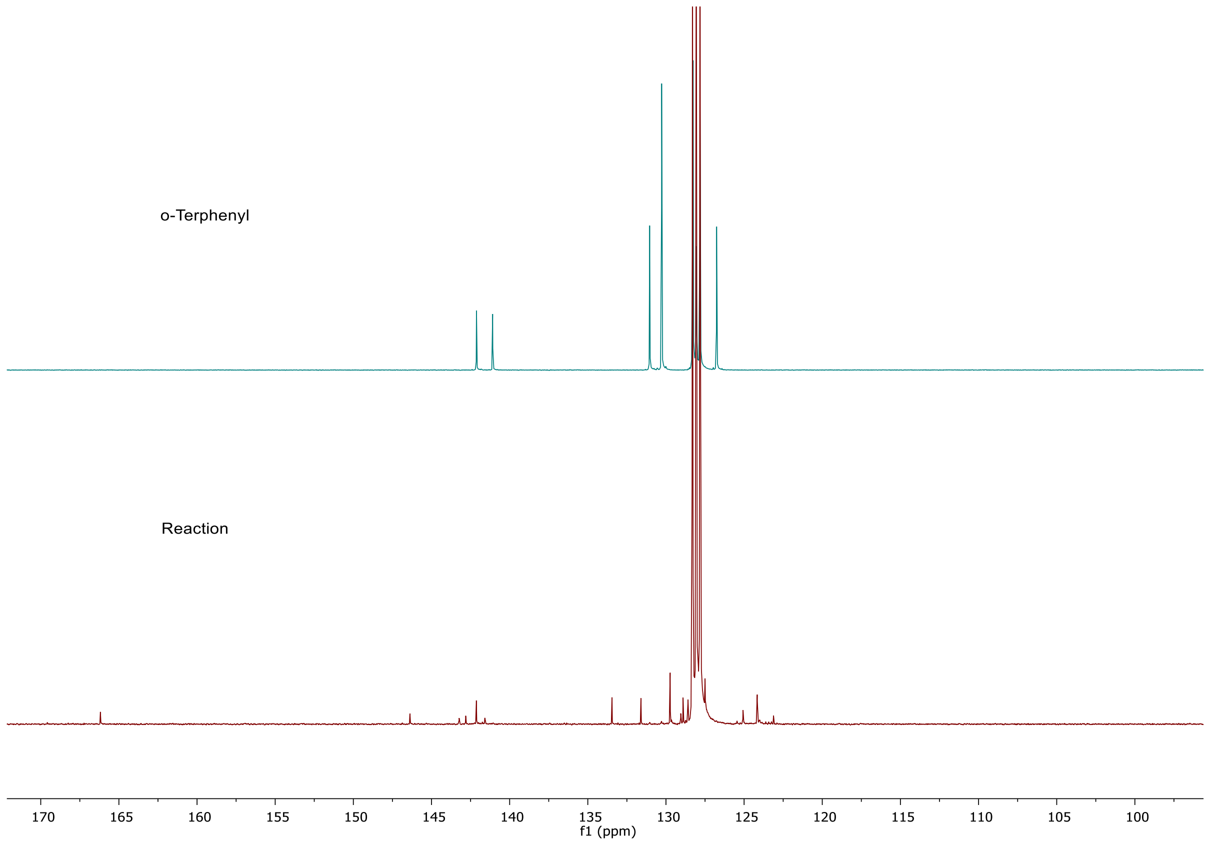

Figure S60. Stacked $^{13}C\{^{1}H\}$ NMR spectra (C_6D_6, 298 K, 100.62 MHz) illustrating the aromatic region of the resulting spectrum from the reaction between **10** and 2-bromobenzene at ambient temperature, with o-terphenyl.

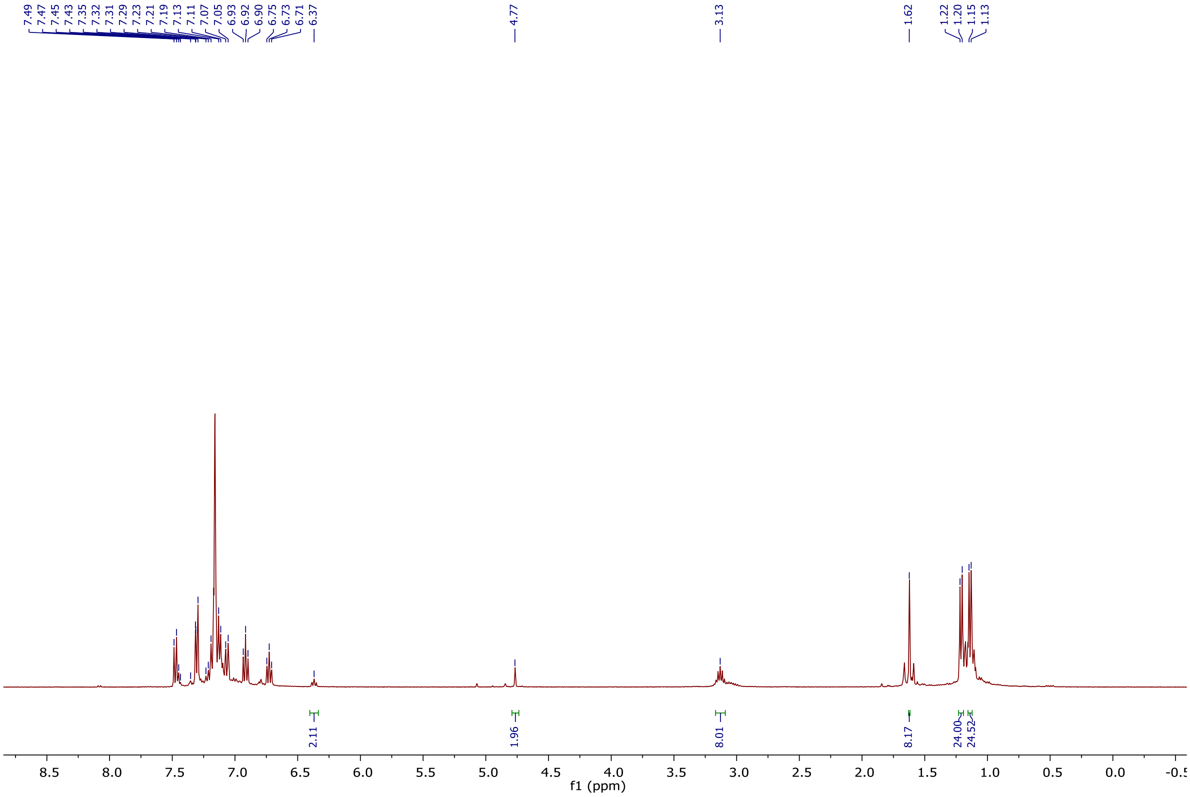

Figure S61. ^{1}H NMR spectrum (C_6D_6, 298 K, 400.13 MHz) resulting from the reaction of **10** and 2-bromobiphenyl for 16 hours at 60 °C. The sample contains 2-bromobiphenyl, [(BDI)Ca(Br)PhCa(BDI)] (**11**) and o-terphenyl.

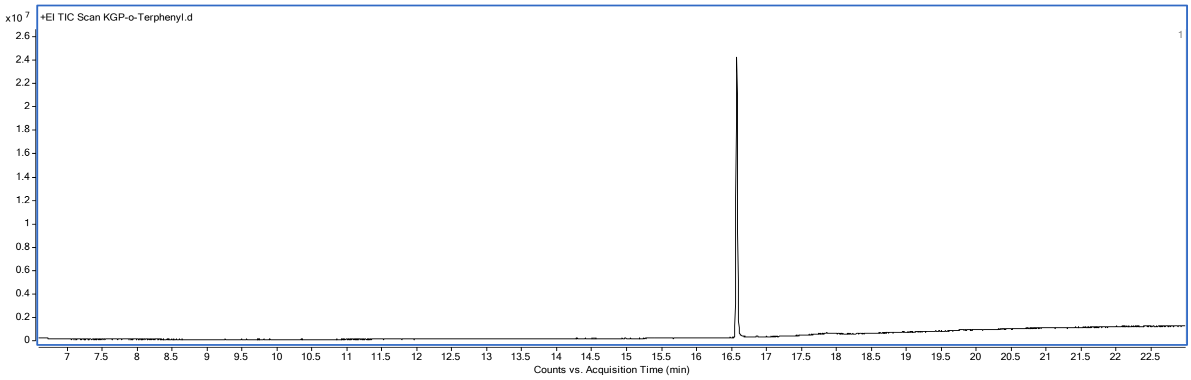

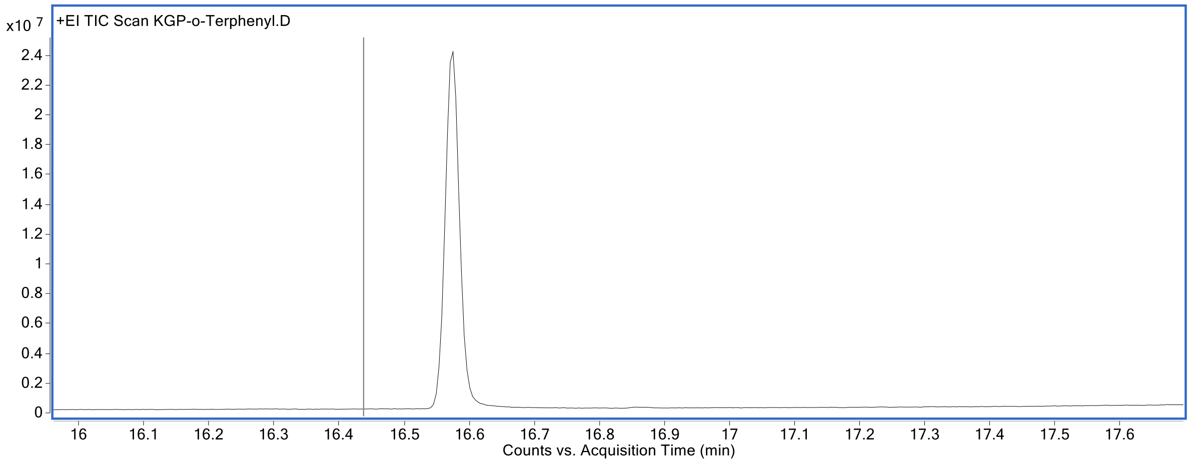

Figure S62. Reference GC-MS chromatogram of Biphenyl. Top. Full chromatogram. Bottom. Expansion of the peak for o-terphenyl (16.57 min).

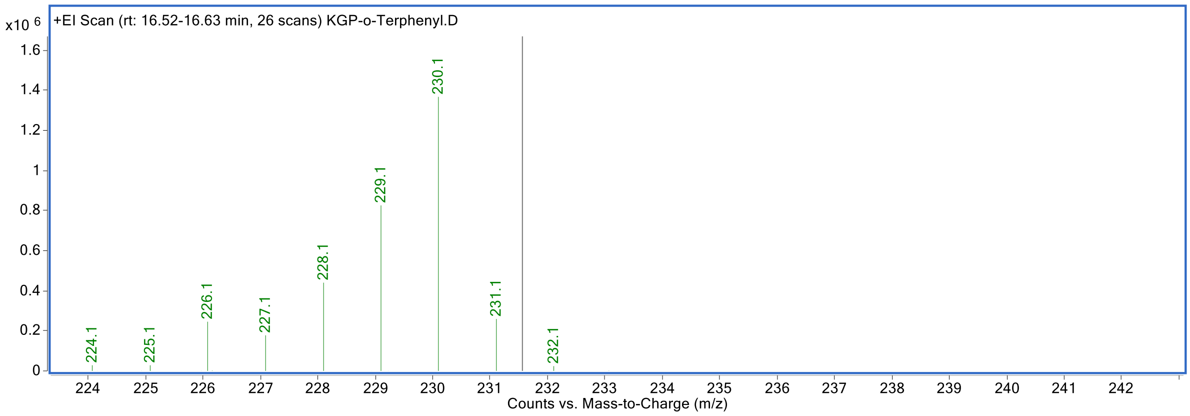

Figure S63. Reference GC-MS Trace showing the molecular ion of o-terphenyl.

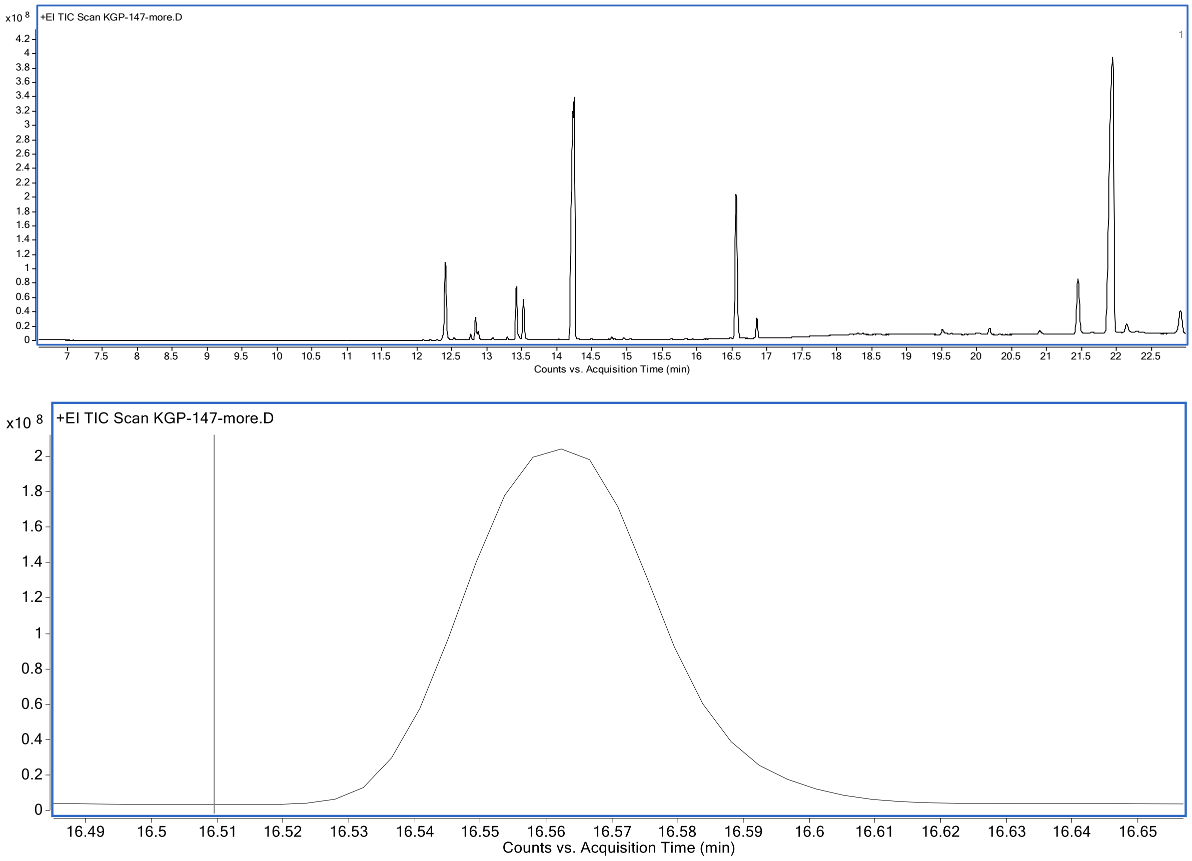

Figure S64. GC-MS chromatogram of the resulting spectra from the reaction between **10** and 2-bromobiphenyl at ambient temperature. Top. Full chromatogram. Bottom. Expansion of the peak for o-terphenyl (16.57 min).

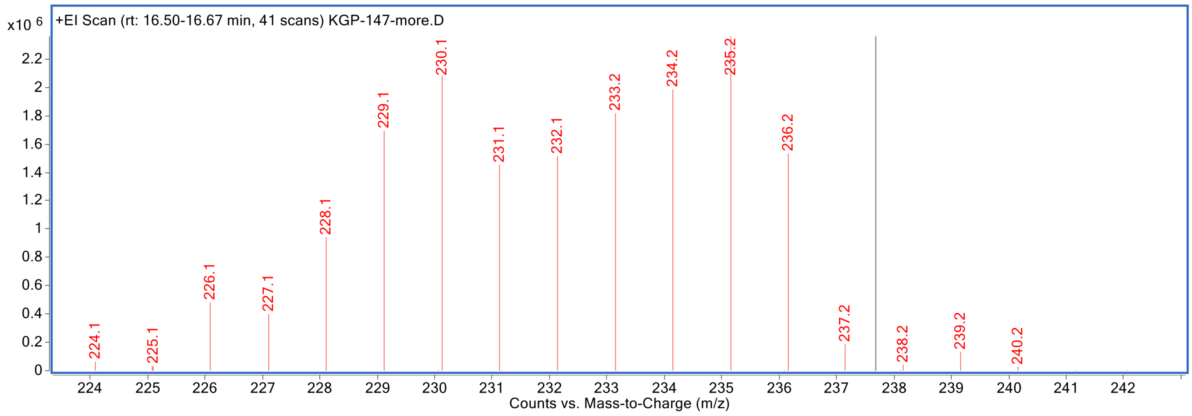

Figure S65. GC-MS trace **10** and 2-bromobiphenyl at ambient temperature, showing the molecular ion for o-terphenyl.

In each case [LCa(Br)PhCaL] was formed, for which only the following resonances can be assigned:

^{1}H NMR (C_6D_6): δ = 6.37 (t, Ar-H, $^{3}J_{HH}$ = 7.19 Hz, 2H), 4.77 (s, NC(CH_3)C$\underline{H}$, 2H), 3.13 (hept, C$\underline{H}$(CH_3)$_2$, $^{3}J_{HH}$ = 6.9 Hz, 8H), 1.62 (s, NC(C$\underline{H}_3$)CH, 12H), 1.21 (d, CH(C$\underline{H}_3$)$_2$, $^{3}J_{HH}$ = 6.9 Hz, 24H), 1.14 (d, CH(C$\underline{H}_3$)$_2$, $^{3}J_{HH}$ = 6.9 Hz, 24H). ^{13}C{^{1}H} NMR (C_6D_6) δ = 197.4 (C_{ipso}, C_6H_5), 166.2 (N$\underline{C}$(CH_3)CH), 146.3 (Ar-C), 142.8 (Ar-C), 129.1 (Ar-C), 128.6 (Ar-C), 125.4 (Ar-C), 125.1 (Ar-C),

124.1 (Ar-C), 93.7 (NC(CH3)C̲H), 28.7 (CH(C̲H3)2), 25.1 (NC(CH̲3)CH), 24.5 (CH(C̲H3)2), 24.4 (CH(C̲H3)2).

Reaction of [LCa(H)PhCaL] () with 2-bromobiphenyl

Bromobenzene (0.7 µl, 0.007 mmol) was added to a C_6D_6 solution of [LCa(H)PhCaL] (15.5 mg, 0.015 mmol) and mixed at ambient temperature for 48 hours, resulting in a pale-yellow solution. Generating a mixture of [(BDI)Ca(Br)PhCa(BDI)] and $[LCaH]_2$. Alternatively, the formation of [(BDI)Ca(Br)PhCa(BDI)] (**11**) and homoleptic calcium complex [(BDI)2Ca] and free BDIH has been observed.

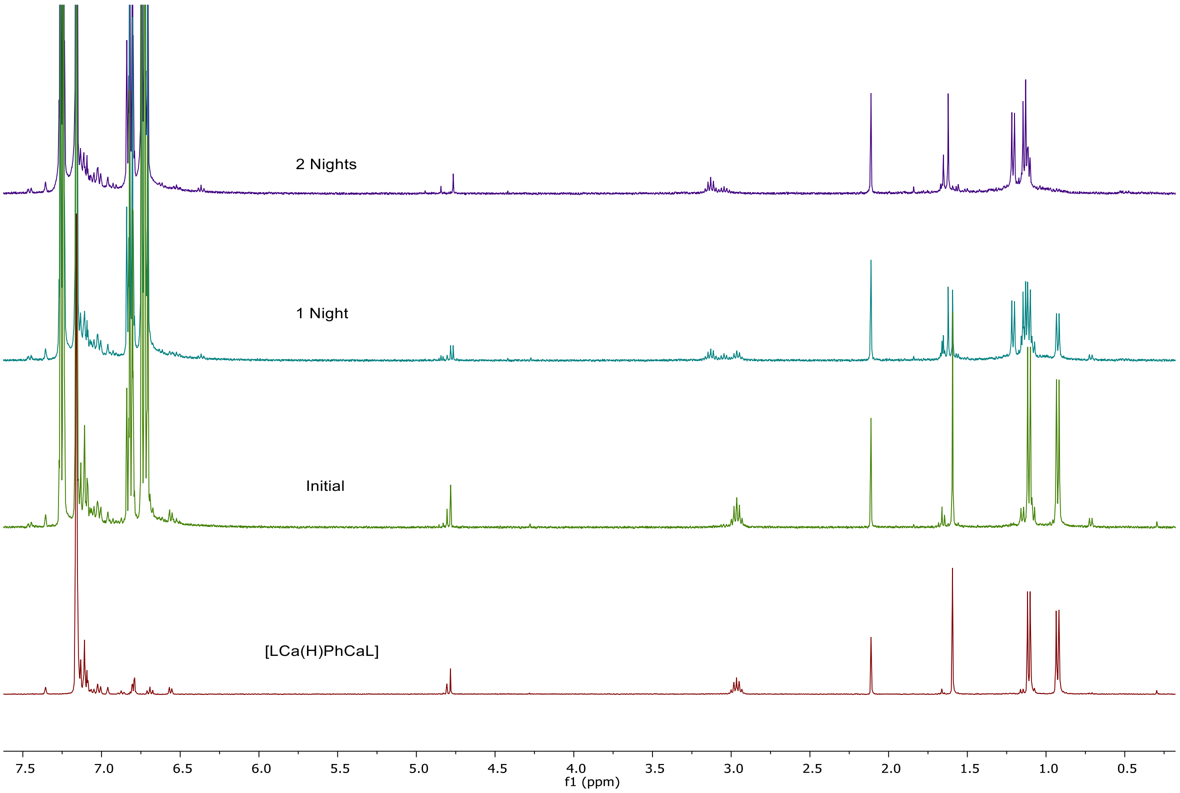

Figure S66. Stacked ^{1}H NMR spectra (C_6D_6, 298 K, 400.13 MHz) of the reaction between [(BDI)Ca(H)PhCa(BDI)] (**9**) and bromobenzene at ambient temperature.

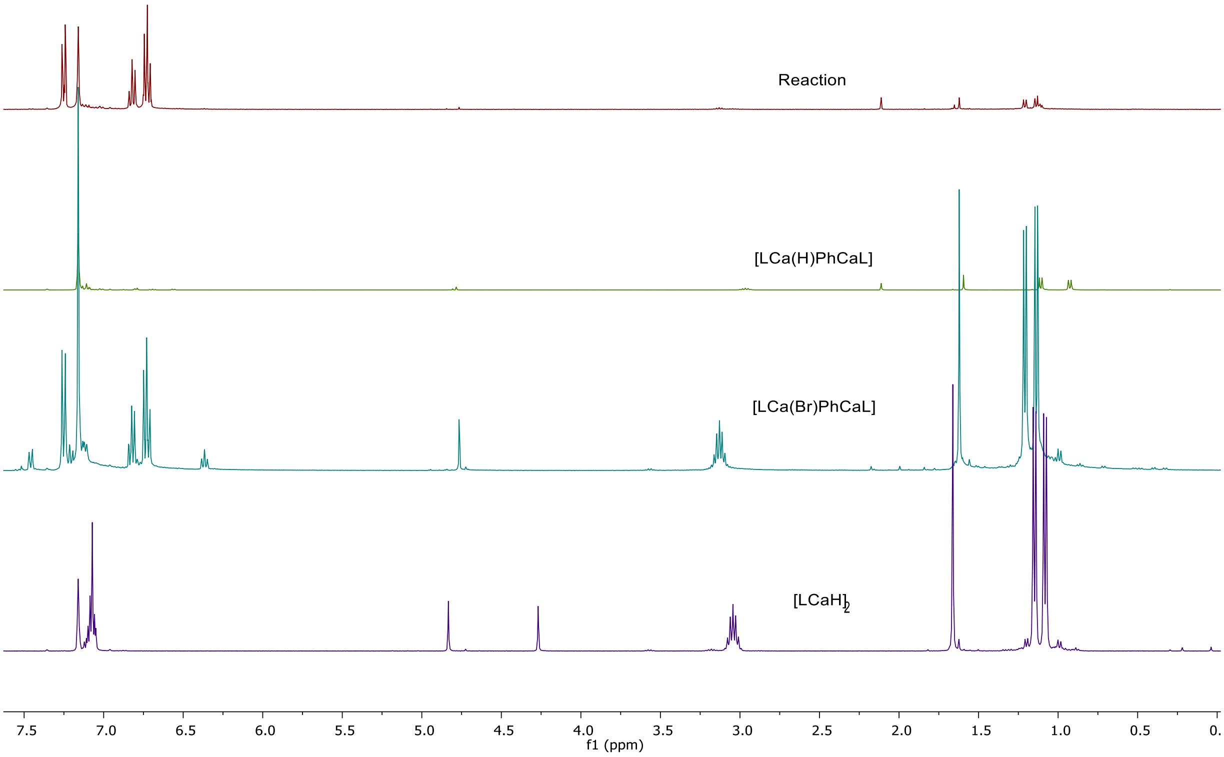

Figure S67. Stacked ^{1}H NMR spectra (C_6D_6, 298 K, 400.13 MHz) of the resulting spectrum from the reaction between [(BDI)Ca(H)PhCa(BDI)] (**9**) and bromobenzene at ambient temperature, with [(BDI)Ca(H)PhCa(BDI)] (**9**), [(BDI)Ca(Br)CaL] (**11**) and $[(BDI)CaH]_2$ (**2**) demonstrating to consumption of all of the former and the generation of the latter two compounds.

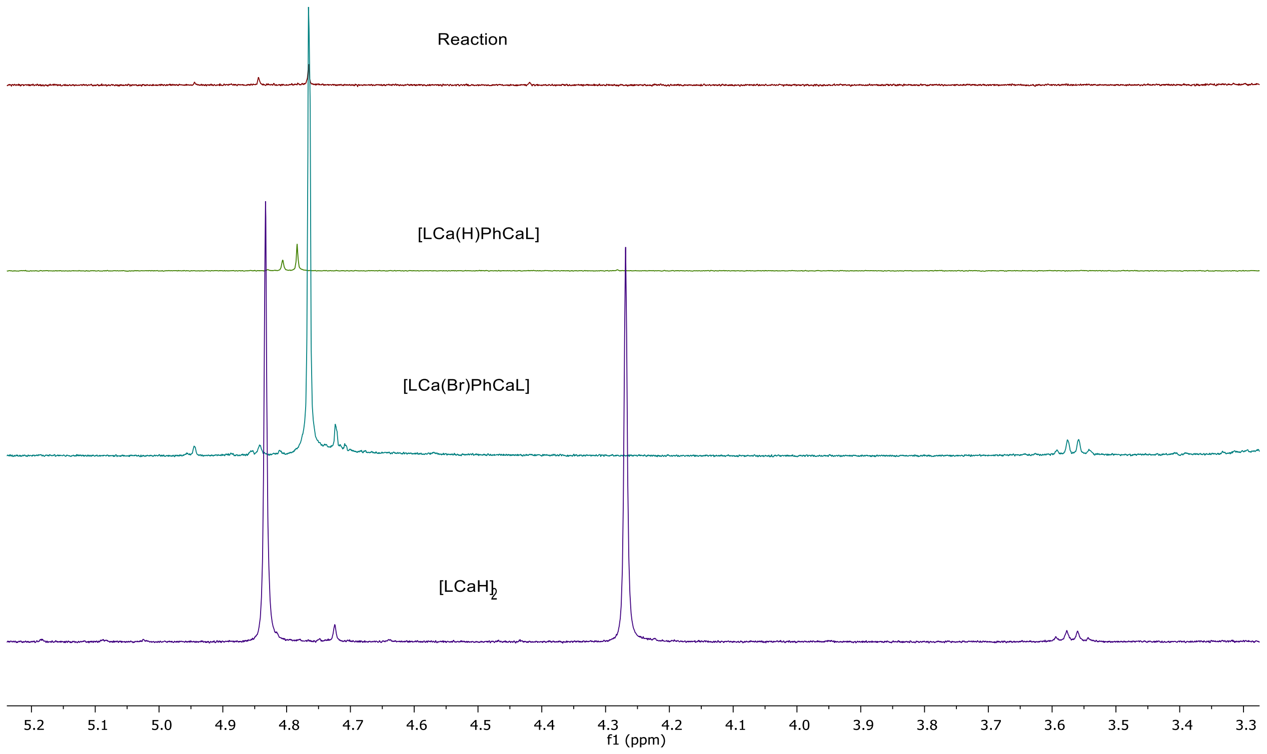

Figure S68. Expansion of methine region in **Figure S67**.

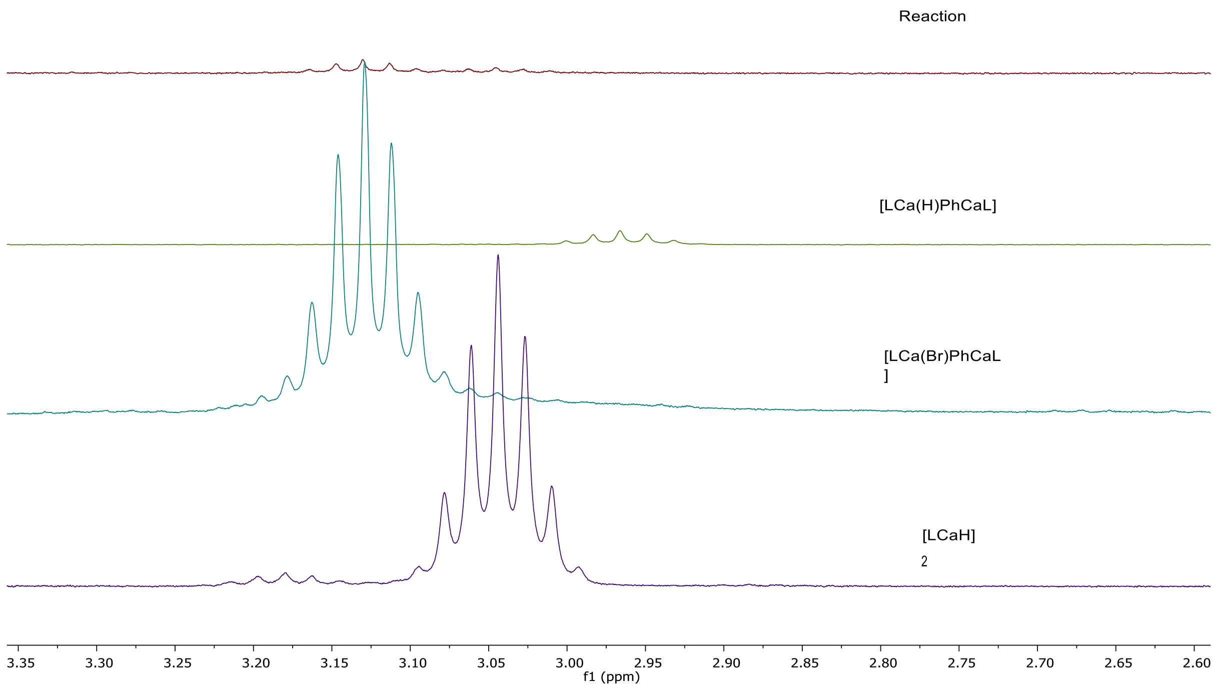

Figure S69. Expansion of the Dipp methine in **Figure S67**.

[(BDI)CaH(CuMes$_2$)Ca(BDI)] (8)

Compound **2** (30 mg, 0.032 mmol) and CuMes (12 mg, 0.065 mmol) were introduced to a J Young's NMR tube and dissolved in C_6D_6 (0.6 cm^3), resulting in a yellow solution. After 48 hours a brown precipitate had formed which was removed via filtration affording a dark brown solution which was placed in the freezer (-35 °C). This produced colourless crystals which proved to be a mixture of both of both BDI(H) and compound **8** after 30 days. The crystals were washed with hexane (3x0.5 cm^3) and dried under reduced pressure.

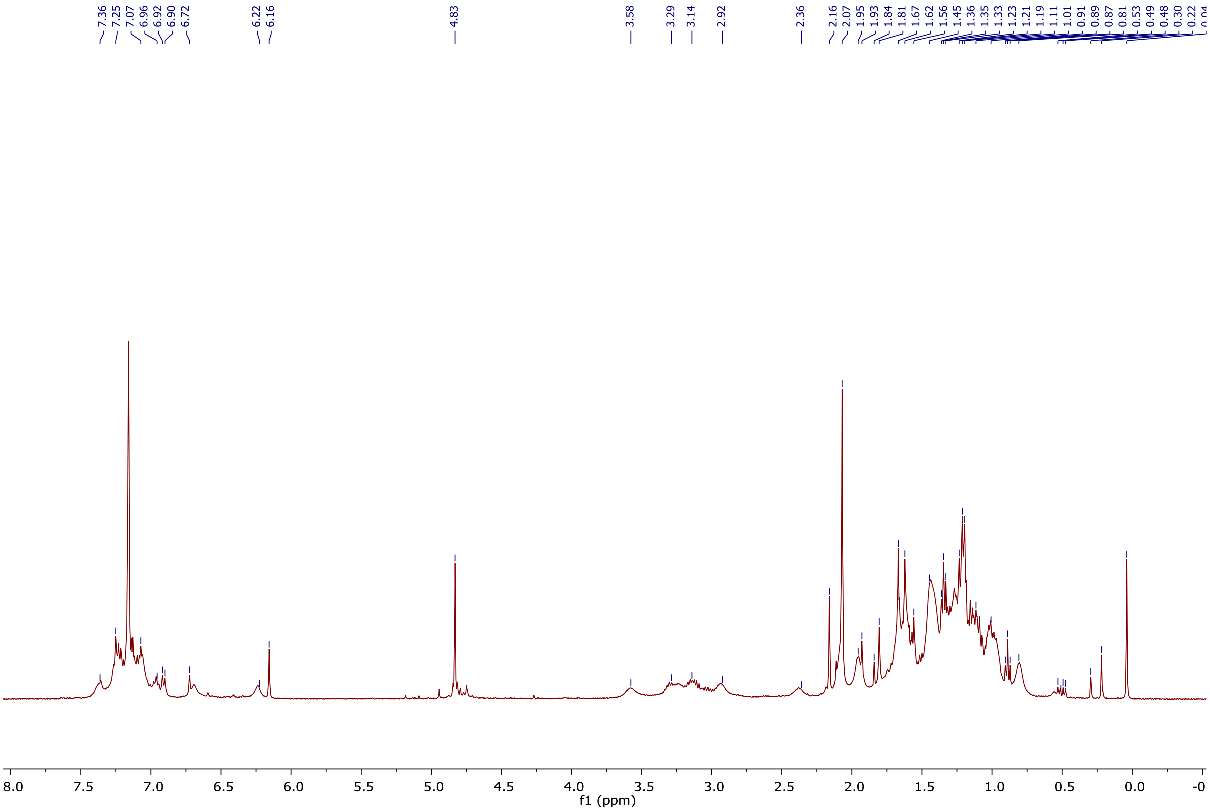

Figure S70. ^{1}H NMR Spectrum (C_6D_6, 298 K, 400.13 MHz) for (**2**) + CuMes$_2$ at the start of the reaction.

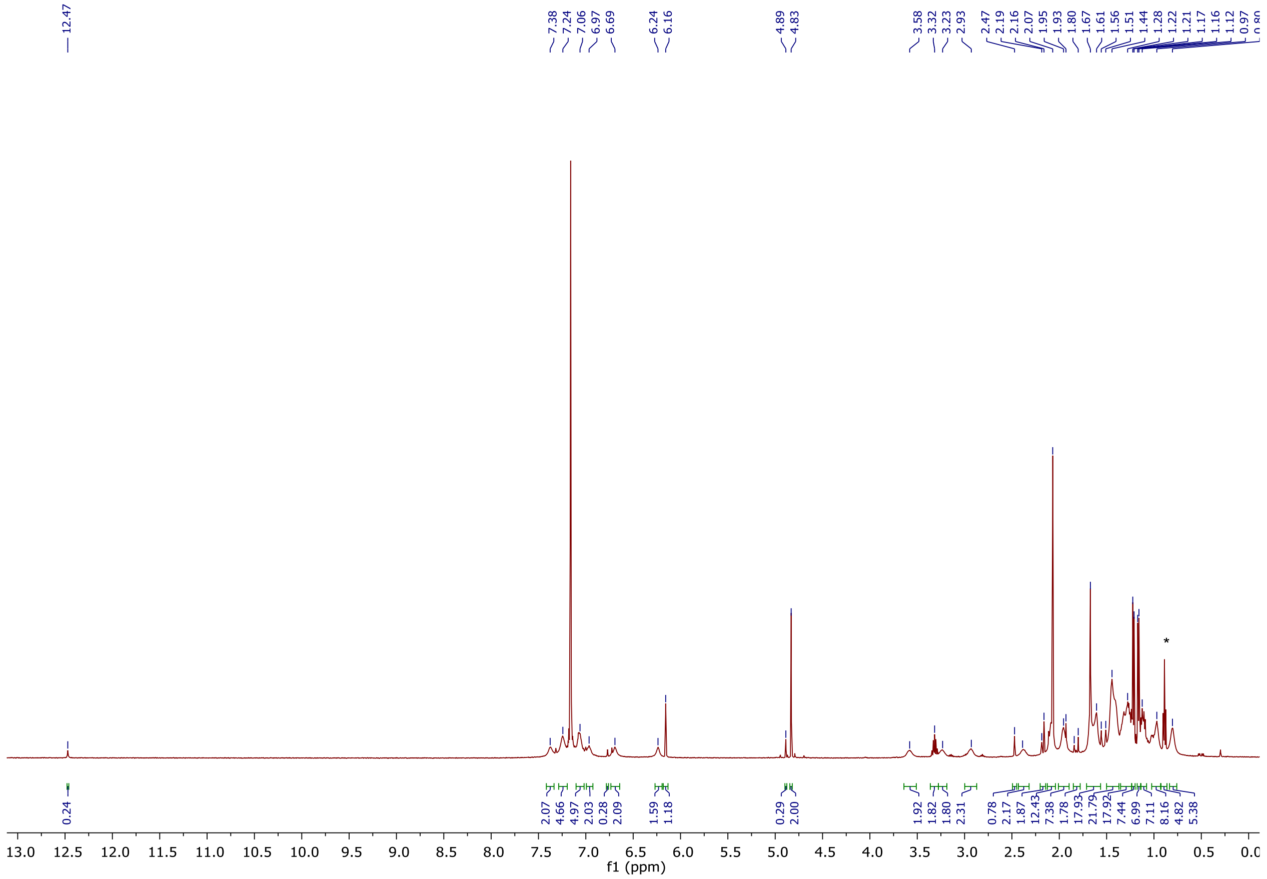

Figure S71. ^{1}H NMR Spectrum (C_6D_6, 298 K, 400.13 MHz) of crystals from (**2**) + $CuMes_2$, containing [(BDI)CaH($CuMes_2$)Ca(BDI)] (**8**), Free BDI(H) and Hexane (*).

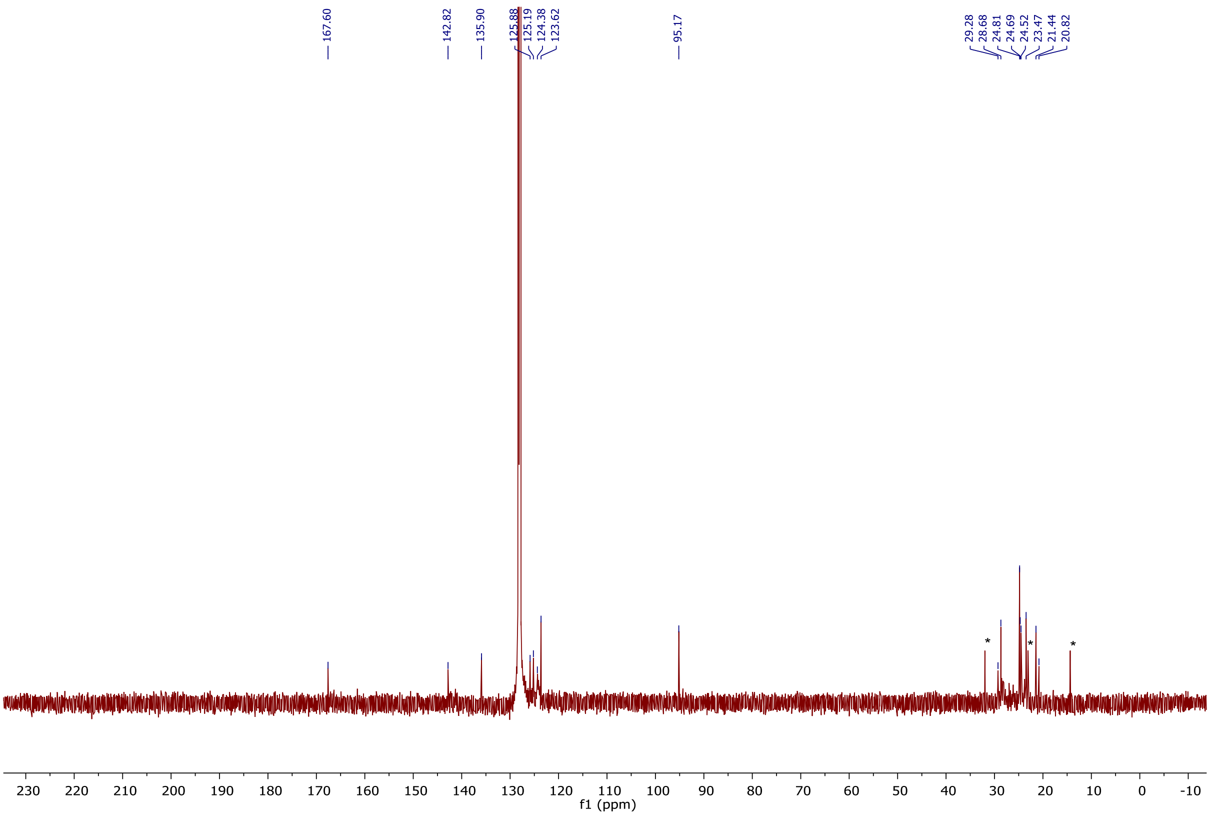

Figure S72. $^{13}C\{^1H\}$ NMR Spectrum (C_6D_6, 298 K, 100.62 MHz) of crystals from (**2**) + $CuMes_2$, containing [(BDI)CaH($CuMes_2$)Ca(BDI)] (**8**), Free BDI(H) and Hexane(*).

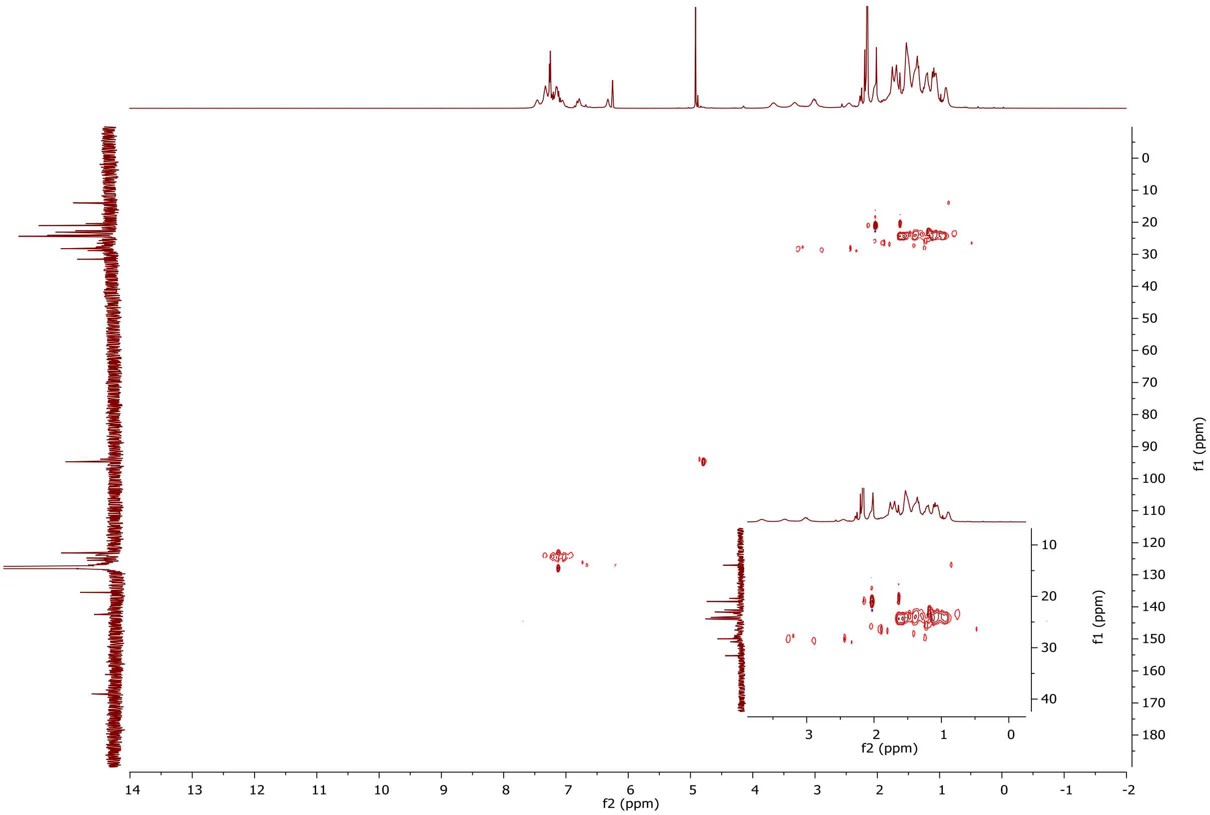

Figure S73. ^1H-^{13}C HSQC trace (C_6D_6, 298 K, 400.13, 100.62 MHz) of crystals from (**2**) + $CuMes_2$, containing [(BDI)CaH($CuMes_2$)Ca(BDI)] (**8**), Free BDI(H) and Hexane.

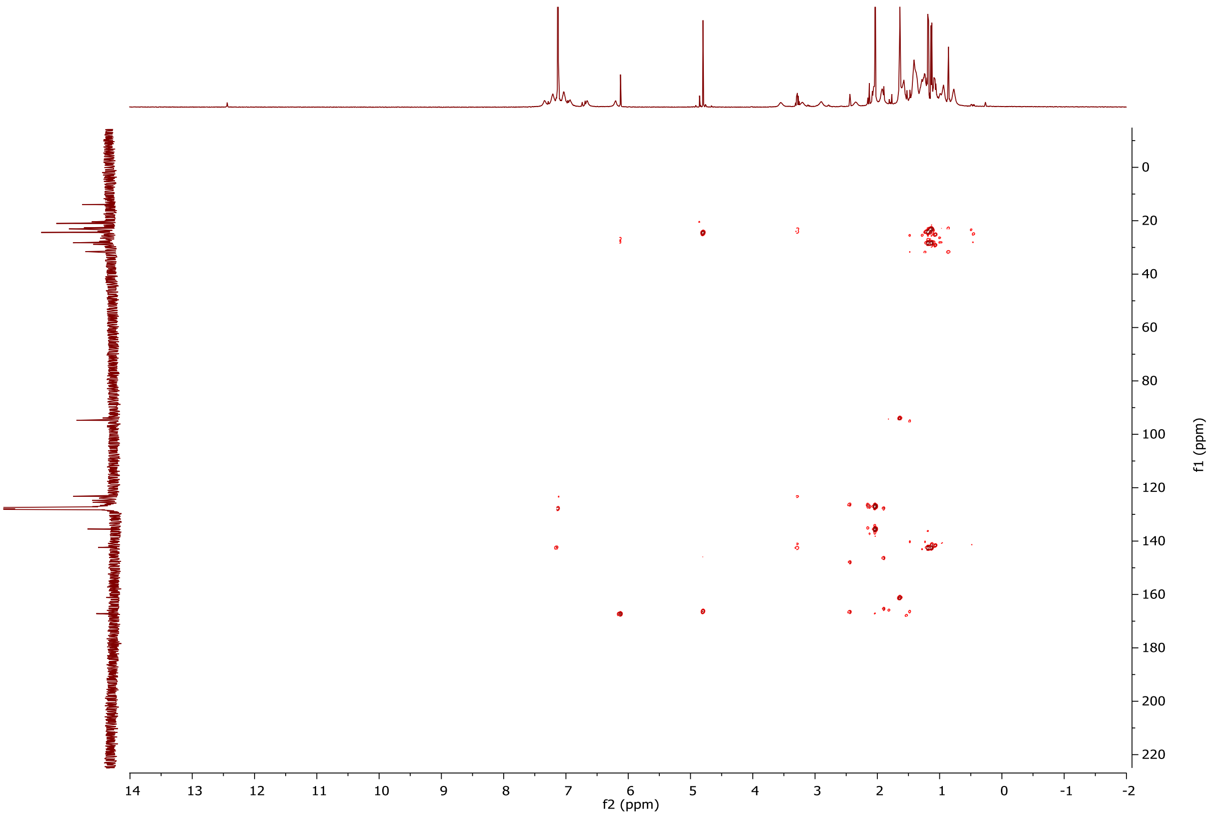

Figure S74. ^{1}H-^{13}C HMBC trace (C_6D_6, 298 K, 400.13, 100.62 MHz) of crystals from (**2**) + $CuMes_2$, containing [(BDI)CaH($CuMes_2$)Ca(BDI)] (**8**), Free BDI(H) and Hexane.

Crystallographic Details

Data were collected for compounds **8 - 11** on a SuperNova, Dual Cu at zero, EosS2 diffractometer (Cu*K*α; λ = 1.54184 Å). The crystals were all maintained at 150 K during data collection. Using Olex2,[3] the structures were solved with the ShelXT[4] program and refined with the ShelXL[5] package using Least Squares minimization.

The asymmetric unit in **8** comprises one molecule of the Ca/Cu complex, one ordered molecule of toluene and one disordered molecule of toluene. The latter was readily modelled as two components with occupancy ratios of 70:30. The aromatic ring in the minor fraction was treated as a regular hexagon in the final least squares, and ADP restraints were also employed. In the main feature, C21 (an isopropyl, methyl carbon) was attempting to dance the 'twist'. It was modelled over three sites in a 40:40:20 ratio, with accompanying distance and ADP restraints. The hydride ligand (H1) was located and refined without restraints.

The asymmetric unit in the structure of **9** comprises half of a dimer molecule and one molecule of toluene. The remainder of the main feature is generated by virtue of a crystallographic 2-fold rotation axis, coincident with Ca1, H1 and C30-33. H1 was located and refined without restraints. The methyl groups based on C16 and C17 were treated for 60:40 disorder. Distance restraints were employed in the disordered region, to assist with convergence.

The asymmetric unit in **10**, like **9**, also contains half of a dimer molecule, the remainder of which is generated *via* a crystallographic inversion centre. H3, attached to C3, was located and refined without restraints.

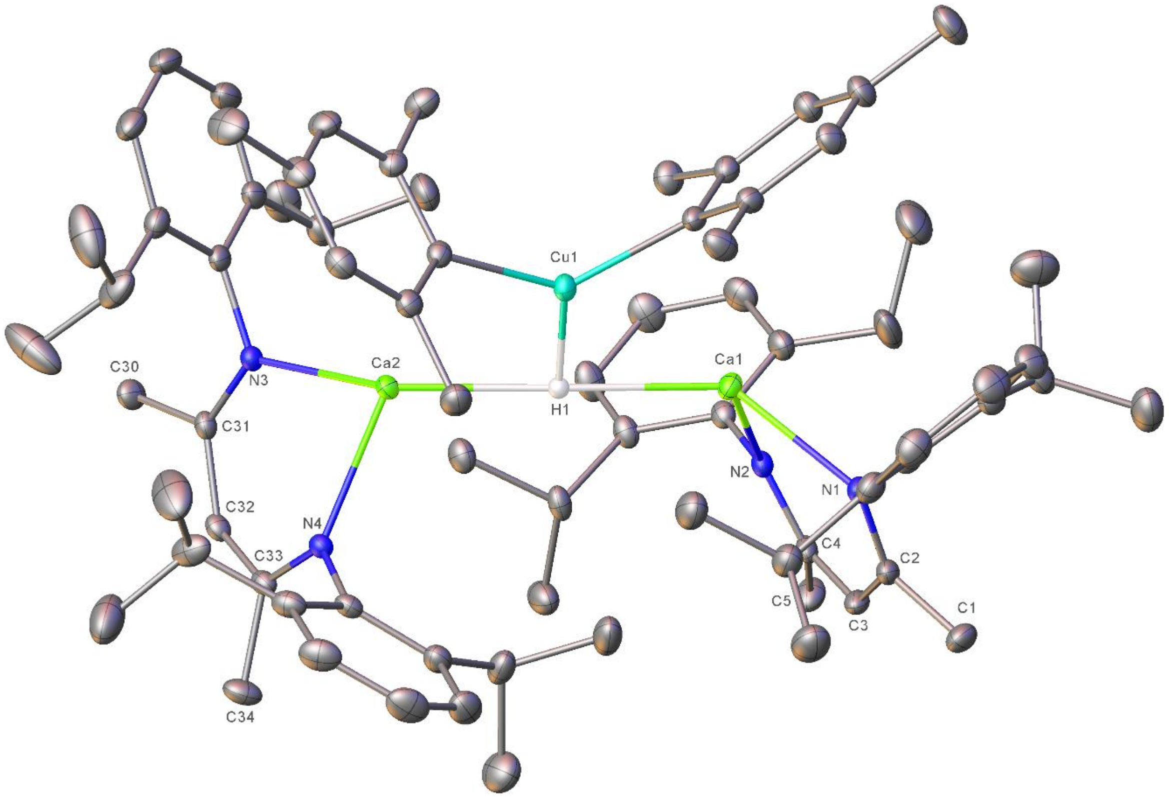

Figure S75. Displacement ellipsoid plot (30% probability) of compound **8**.

Table S1: Single crystal X-ray diffraction analysis of compounds **8 - 11**.

Compound	**8**	**9**	**10**	**11**
Empirical formula	$C_{90}H_{121}Ca_2CuN_4$	$C_{78}H_{104}Ca_2N_4$	$C_{35}H_{46}CaN_2$	$C_{64}H_{87}BrCa_2N_4$
Formula weight	1402.60	1177.81	534.82	1072.44
Crystal system	monoclinic	monoclinic	monoclinic	triclinic
Space group	$P2_1/c$	$C2/c$	$P2_1/n$	P-1
a/Å	16.3709(1)	23.2964(4)	11.83040(10)	9.1351(2)
b/Å	38.7383(2)	11.45398(16)	15.17210(10)	13.9397(3)
c/Å	13.7343(1)	27.5622(4)	17.81620(10)	23.6217(5)
α/°	90	90	90	93.187(2)
β/°	112.363(1)	107.5826(16)	98.7940(10)	98.481(2)
γ/°	90	90	90	93.288(2)
U/Å^3	8054.96(10)	7011.03(19)	3160.27(4)	2963.95(11)
Z	4	4	4	2
ρ_{calc}g cm^{-3}	1.157	1.116	1.124	1.202
μ/mm^{-1}	1.834	1.733	1.876	2.760
F(000)	3032.0	2560.0	1160.0	1148.0
Crystal size/mm^3	0.3 × 0.166 × 0.107	0.353 × 0.293 × 0.111	0.423 × 0.347 × 0.296	0.168 × 0.11 × 0.051
2□ range for data collection/°	5.838 to 146.304	7.962 to 147.082	7.692 to 146.184	7.194 to 147.022
Index ranges	$-19 \le h \le 20, -47 \le k \le 47, -17 \le l \le 11$	$-28 \le h \le 28, -14 \le k \le 13, -30 \le l \le 34$	$-14 \le h \le 14, -18 \le k \le 18, -22 \le l \le 21$	$-11 \le h \le 9, -17 \le k \le 17, -29 \le l \le 28$
Reflections collected	116753	29767	39281	26038
Independent reflections, R_{int}	16057 [R_{int} = 0.0358, R_{sigma} = 0.0209]	6944 [R_{int} = 0.0335, R_{sigma} = 0.0277]	6316 [R_{int} = 0.0336, R_{sigma} = 0.0199]	11817 [R_{int} = 0.0238, R_{sigma} = 0.0341]
Data/restraints/parameters	16057/79/990	6944/2/413	6316/0/365	11817/0/664
Goodness−of−fit on F^2	1.013	1.052	1.019	1.020
Final R1, wR_2[I>=2σ (I)]	R_1 = 0.0407, wR_2 = 0.1035	R_1 = 0.0459, wR_2 = 0.1228	R_1 = 0.0346, wR_2 = 0.0921	R_1 = 0.0322, wR_2 = 0.0812
Final R1, wR_2[all data]	R_1 = 0.0437, wR_2 = 0.1064	R_1 = 0.0503, wR_2 = 0.1264	R_1 = 0.0356, wR_2 = 0.0931	R_1 = 0.0380, wR_2 = 0.0850
Largest diff. peak/hole / e Å^{-3}	0.91/-0.62	0.44/-0.45	0.32/-0.28	0.31/-0.33

Computational Details

Calculations were carried out using the Gaussian09 package[6] at the DFT level by means of the hybrid density functional B3PW91.[7] A triple-zeta 6-311G basis set augmented by a polarization and diffuse function was used for the Ca atom. Polarized all electron triple-zeta 6-311G(d,p) basis set were used for N, whereas a polarized all electron double-zeta 6-31G(d) basis set were used for the C and H atoms. For the Cl and Br atoms, the Stuttgart-Dresden pseudopotentials were used in combination with their associated basis sets[8] augmented by a set of polarization functions (d-orbital polarization exponents of 0.643 and 0.43 for the Cl and Br atoms, respectively).[9] The nature of the optimized stationary point, minima or transition state, has been verified by means of analytical frequency calculation at 298.15 K and 1 atm. The geometry optimizations have been achieved without any geometrical constraints. IRC calculations were carried out in order to confirm the connectivity between reactant(s), transition state and product(s). Energy data are reported in the gas phase. They correspond to the enthalpy (Gibbs free energy) of the computed compounds. The calculated profiles were simulated with a dispersion corrected functional by using the D3 version of Grimme's dispersion with Becke-Johnson damping.[10] The electron density and partial charge distribution were examined in terms of localized electron-pair bonding units using the NBO program.[11,12]

Figure S76. DFT-optimised structures of **9 (9_opt** and **9'_opt)**.

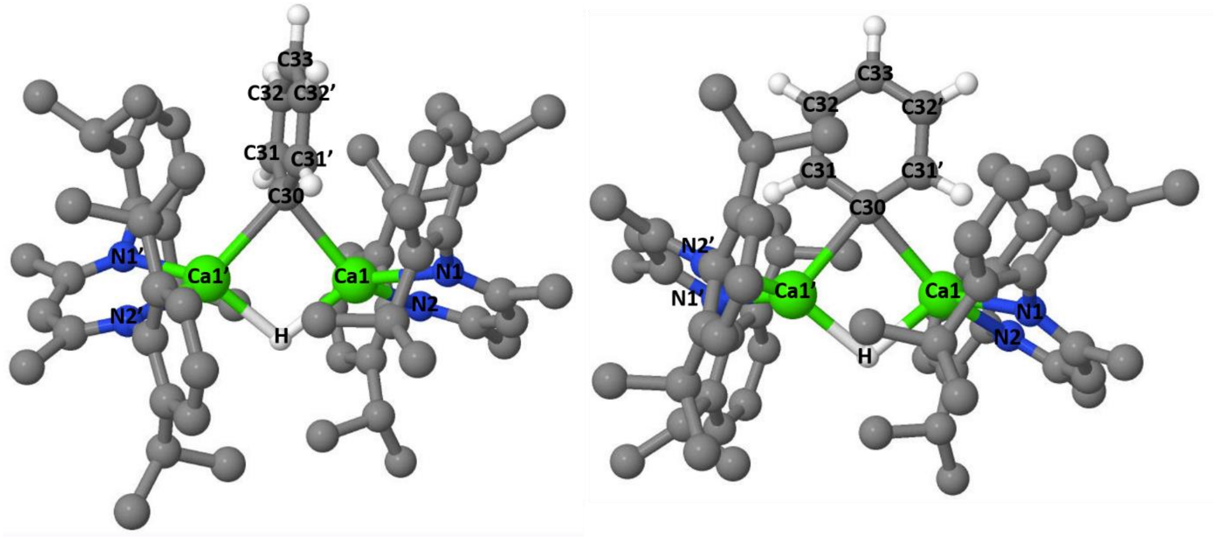

Table S2. Relevant bond length distances and angles for **9**, **9_opt** and **9'_opt**.

	**Ca1-Ca1’**	**Ca1-C30**	**Ca1-H**	**Ca1-N1**	**Ca1-N2**
			**Bond lengths (Å)**		
**9**	3.518	2.5838(19)	2.237	2.3387(12)	2.3602(13)
**9_opt**	3.418	2.567/2.584	2.164/2.172	2.334/2.342	2.350/2.355
**9'_opt**	3.362	2.514/2.576	2.151/2.152	2.349/2.349	2.368/2.344

	**Ca1-C30-Ca1’**	**Ca1-H-Ca1’**
	**Bond angles (°)**	
**9**	85.79	103.69
**9_opt**	83.15	104.05
**9'_opt**	82.66	102.73

Figure S77. DFT-optimised structures of **10 (10_opt** and **10'_opt)**.

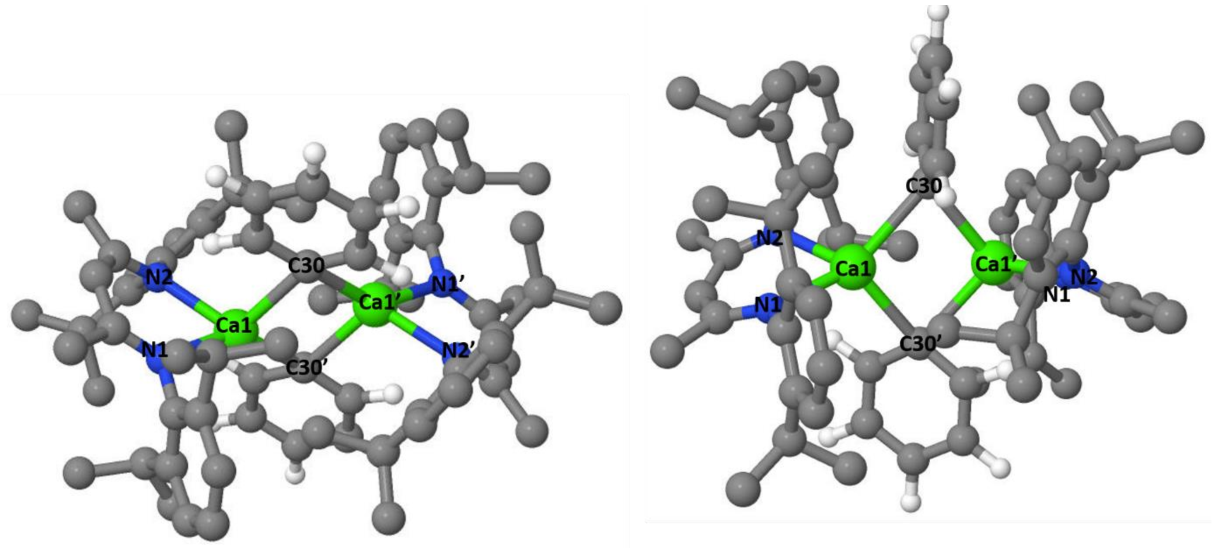

Table S3. Relevant bond length distances and angles for **10**, **10_opt** and **10'_opt**

	**Ca1-Ca1'**	**Ca1-C30**	**Ca1-C30'**	**Ca1-N1**	**Ca1-N2**
		**Bond lengths (Å)**			
**10**	3.391	2.5402(12)	2.5667(12)	2.3588(10)	2.3610(10)
**10_opt**	3.331	2.524/2.518	2.566/2.545	2.375/2.387	2.373/2.374
**10'_opt**	3.344	2.574/2.575	2.537/2.538	2.365	2.344

	**Ca1-C30-Ca1'**
**Bond angles (°)**	
**10**	83.22
**10_opt**	81.19/81.87
**10'_opt**	81.00/82.43

Figure S78. Computed enthalpy (Gibbs free energy) profiles for the second reaction of complex **D-Br** with bromo-benzene at room temperature.

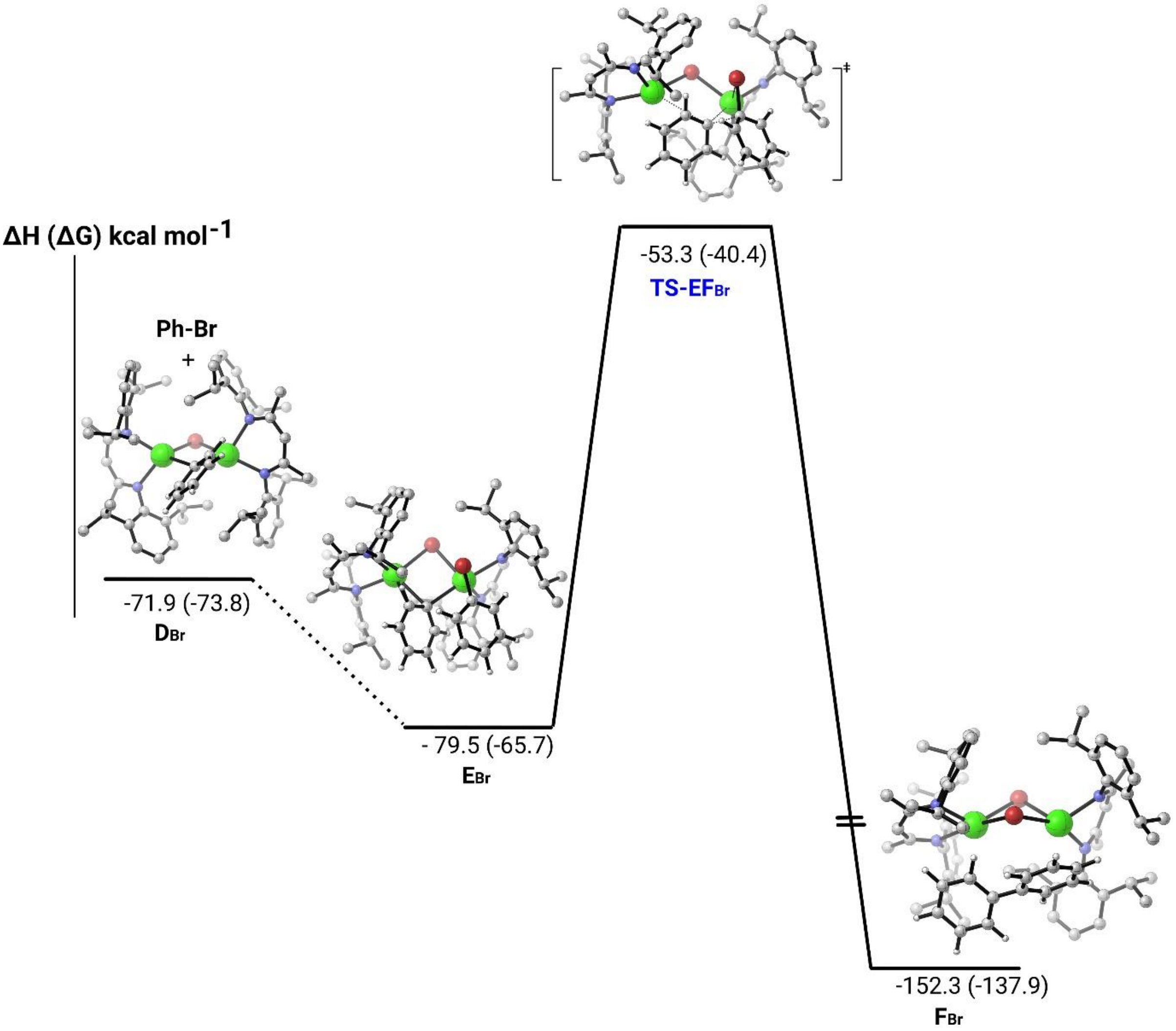

Figure S79. Computed enthalpy (Gibbs free energy) profile for the reaction of complex **A-Cl (10_opt)** with chloro-benzene at room temperature.

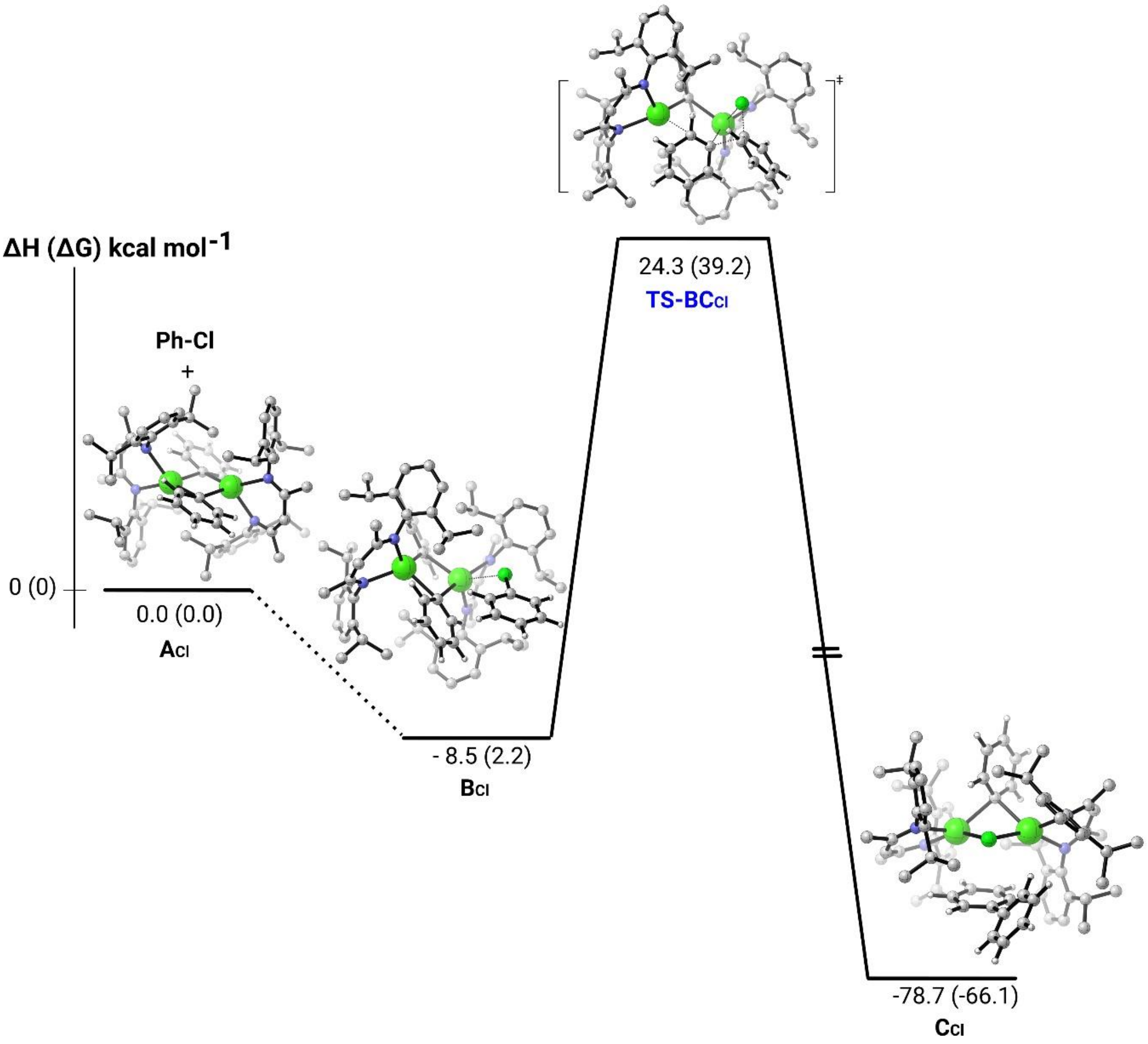

Figure S80. Computed enthalpy (Gibbs free energy) profiles for the second reaction of complex D-Cl with chloro-benzene at room temperature.

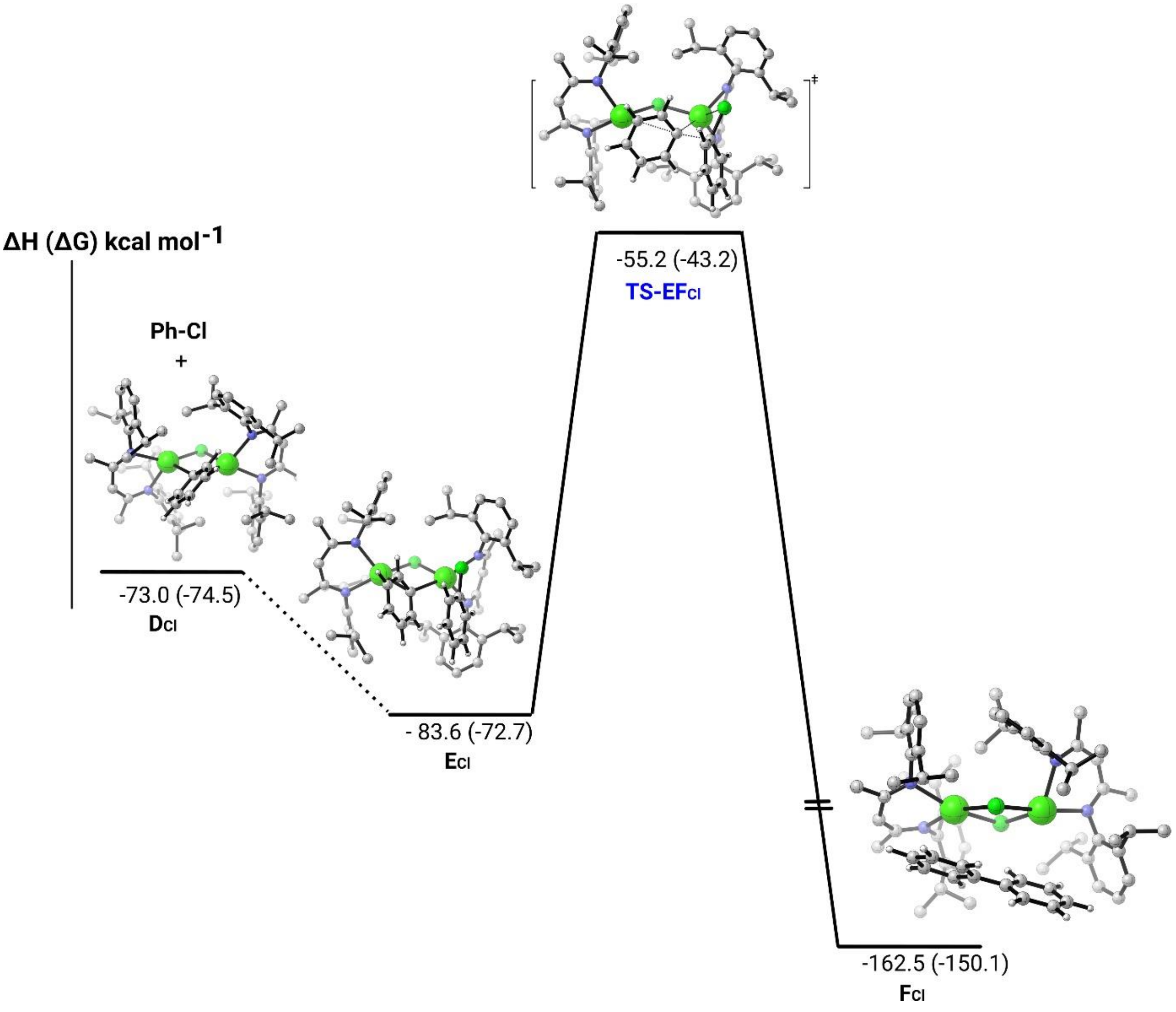

Geometries of all computed complexes

12

[Chlorobenzene] scf done: -246.603491 / Energies= -246.506458 / Enthalpies= -246.505514 / Free Energies= -246.541753

C	-1.249798	-2.467900	6.353311
C	-2.292156	-1.628254	5.972990
C	-2.167662	-0.258706	6.198453
C	-1.018385	0.257889	6.794484
C	0.014191	-0.600805	7.167873
C	-0.094125	-1.972894	6.949600
Cl	-1.396513	-4.194158	6.074176
H	0.702368	-2.651180	7.235840
H	0.912922	-0.205427	7.632997
H	-0.927672	1.326280	6.967372
H	-2.976457	0.404551	5.904627
H	-3.181442	-2.041985	5.509969

168

[A-Cl] scf done: -4296.584459 / Sum of electronic and thermal Energies= -4295.035912 / Sum of electronic and thermal Enthalpies= -4295.034968 / Sum of electronic and thermal Free Energies= -4295.235413

Ca	3.815227	8.766180	9.739653
N	2.860869	9.373666	11.828084
N	4.381181	11.069822	9.834140
C	2.659223	10.528727	14.000136
H	2.431825	11.579627	14.201481
H	3.391396	10.203320	14.748046
H	1.752858	9.935204	14.136040
C	3.233266	10.371148	12.606878
C	4.165626	11.368254	12.232865
H	4.441742	12.046452	13.034193
C	4.625662	11.739596	10.959959
C	5.426243	13.021655	10.884995
H	6.340044	12.869247	10.301488
H	5.694808	13.384341	11.879734
H	4.857559	13.805652	10.374219
C	1.883597	8.434426	12.237188
C	2.180643	7.408935	13.163513
C	3.546907	7.270076	13.807559
H	4.164389	8.111668	13.479944
C	3.465137	7.296842	15.338394
H	2.933387	8.180302	15.705256
H	4.471927	7.295191	15.772320
H	2.941119	6.413098	15.721363
C	4.243451	5.989012	13.337374
H	3.679663	5.098517	13.638418
H	5.246847	5.918975	13.769369
H	4.353589	5.963160	12.251390
C	1.191450	6.463193	13.450583
H	1.410331	5.672113	14.163603
C	-0.052122	6.500768	12.830178
H	-0.804171	5.752074	13.064785
C	-0.323044	7.495746	11.895603
H	-1.290962	7.516311	11.403577
C	0.629296	8.465577	11.582059
C	0.316014	9.576499	10.597445
H	1.271225	9.945775	10.200673
C	-0.322414	10.759939	11.334830
H	-1.270346	10.457594	11.795883
H	-0.522900	11.587097	10.644927
H	0.337562	11.127322	12.127193
C	-0.539976	9.126311	9.413967
H	-0.090026	8.274591	8.892917
H	-0.640469	9.942644	8.691228
H	-1.551890	8.842129	9.724841

C	4.648058	11.701755	8.589918
C	3.700109	12.600020	8.045982
C	2.476918	13.039192	8.832689
H	2.564535	12.636302	9.846357
C	2.385703	14.566080	8.937061
H	3.305815	14.999142	9.343568
H	1.555150	14.854638	9.591920
H	2.210251	15.025479	7.957202
C	1.190349	12.470560	8.226757
H	1.072764	12.784064	7.182448
H	0.312200	12.818571	8.783008
H	1.193906	11.377889	8.252706
C	3.895270	13.080718	6.749652
H	3.162165	13.759304	6.319546
C	5.004912	12.708115	5.997373
H	5.132161	13.084535	4.985933
C	5.949695	11.853209	6.551539
H	6.819047	11.558149	5.968869
C	5.792238	11.346839	7.843759
C	6.831897	10.405157	8.412564
H	6.520293	10.141172	9.426390
C	8.218339	11.046224	8.512426
H	8.605503	11.309741	7.521348
H	8.926230	10.347988	8.974376
H	8.193711	11.958557	9.117894
C	6.900216	9.121636	7.586023
H	5.879411	8.770517	7.378465
H	7.472917	8.352640	8.117527
H	7.372694	9.287538	6.615215
C	5.802094	7.312699	10.266930
C	6.127411	8.280958	11.242725
H	5.663584	9.273647	11.245364
C	7.040082	8.065287	12.275893

H	7.236776	8.855497	12.997282
C	7.688997	6.837550	12.382233
H	8.398455	6.655554	13.185642
C	7.400764	5.839674	11.455925
H	7.868849	4.863035	11.540318
C	6.480952	6.088129	10.435104
H	6.270984	5.225221	9.791732
Ca	5.317467	6.423192	7.909025
N	6.158162	5.831328	5.754747
N	4.786763	4.111698	7.829684
C	6.199016	4.696023	3.561507
H	6.395075	3.645315	3.329337
H	5.418222	5.042689	2.874922
H	7.101479	5.278593	3.366447
C	5.729157	4.840893	4.994937
C	4.828276	3.835847	5.421883
H	4.497812	3.166444	4.634041
C	4.464646	3.447880	6.719484
C	3.665209	2.169136	6.842430
H	2.713323	2.366194	7.348576
H	3.458620	1.726643	5.865544
H	4.194295	1.434016	7.456307
C	7.119945	6.756550	5.278287
C	6.774874	7.793655	4.383668
C	5.368747	7.959458	3.839831
H	4.759845	7.130994	4.213896
C	5.338825	7.931408	2.306910
H	5.824166	7.037885	1.902626
H	4.303258	7.955123	1.947856
H	5.852369	8.804515	1.886996
C	4.734354	9.255336	4.353456
H	5.279219	10.134407	3.990394
H	3.696073	9.332852	4.014907

H	4.726338	9.296056	5.443870
C	7.758746	8.721227	4.026834
H	7.502144	9.521519	3.337311
C	9.046868	8.648941	4.544191
H	9.795566	9.381736	4.254817
C	9.369058	7.638562	5.446274
H	10.371435	7.593117	5.860821
C	8.421240	6.690439	5.830987
C	8.775410	5.574288	6.796318
H	7.859635	5.303609	7.340975
C	9.193003	4.318352	6.021762
H	10.079023	4.524046	5.409350
H	9.431755	3.498348	6.707984
H	8.389881	3.983894	5.358536
C	9.832068	5.968024	7.827817
H	9.555907	6.887863	8.355120
H	9.945473	5.173044	8.571564
H	10.814611	6.122712	7.367012
C	4.612746	3.467038	9.084115
C	5.660576	2.670590	9.601204
C	6.888744	2.343714	8.768912
H	6.784070	2.857442	7.807993
C	6.988665	0.838658	8.492551
H	6.083362	0.457542	8.009654
H	7.839083	0.625026	7.834139
H	7.132454	0.273251	9.421131
C	8.177485	2.848916	9.423480
H	8.312412	2.426109	10.426147
H	9.050695	2.565078	8.824866
H	8.174081	3.938204	9.515209
C	5.546987	2.173860	10.901457
H	6.351064	1.566030	11.310242
C	4.427018	2.441408	11.682539
H	4.363327	2.057482	12.697172
C	3.388143	3.199287	11.154315
H	2.511009	3.408640	11.761473
C	3.458785	3.711823	9.857303
C	2.332405	4.565271	9.312986
H	2.534841	4.742024	8.252699
C	0.961671	3.894113	9.420412
H	0.669754	3.745694	10.466244
H	0.196009	4.522345	8.950650
H	0.955235	2.916444	8.926158
C	2.315161	5.918920	10.023514
H	3.340022	6.312823	10.081178
H	1.667646	6.628732	9.498220
H	1.960741	5.820926	11.051853
C	3.314572	7.874811	7.409514
C	2.898048	6.867369	6.512640
H	3.351743	5.870529	6.521168
C	1.903921	7.050269	5.550792
H	1.637210	6.232069	4.885198
C	1.258918	8.280980	5.445740
H	0.481465	8.434869	4.701551
C	1.636299	9.316332	6.295810
H	1.166998	10.292650	6.211168
C	2.641736	9.103082	7.241274
H	2.926279	9.990405	7.820013

180

[B-Cl] scf done: -4543.205419 / Sum of electronic and thermal Energies= -4541.555033 / Sum of electronic and thermal Enthalpies= -4541.554089 / Sum of electronic and thermal Free Energies= -4541.773566

C	1.165701	4.231247	-0.495422
C	-0.191453	4.305525	-0.102519
C	-1.127547	5.043059	-0.864637
C	-0.663307	5.803431	-1.941431
C	0.685486	5.821508	-2.273736

C	1.580771	5.023225	-1.569900
N	-0.659554	3.621890	1.051374
C	-0.738195	4.347806	2.170344
C	-0.104946	5.728551	2.215145
C	-2.616910	4.975492	-0.552813
C	-3.473986	5.089360	-1.817187
C	2.147725	3.287782	0.193210
C	3.391519	3.001834	-0.652354
C	-1.421119	3.972195	3.339675
C	-2.349555	2.929771	3.568944
C	-3.197710	3.107861	4.812394
N	-2.517238	1.892394	2.771368
C	-3.494811	0.902895	3.027231
C	-3.347981	-0.039308	4.070565
C	-4.323537	-1.030543	4.215252
C	-5.401868	-1.121518	3.344288
C	-5.519193	-0.210548	2.298674
C	-4.578190	0.803691	2.119590
C	-2.147779	-0.044381	5.000076
C	-2.564404	0.035116	6.473619
C	-4.731483	1.824722	1.007561
C	-5.428375	1.286366	-0.238660
C	-1.279079	-1.284559	4.761758
C	-5.420391	3.089013	1.533962
C	-3.090981	6.010574	0.476614
C	2.599046	3.753324	1.583436
Ca	-1.212160	1.314240	0.889117
C	-1.762463	0.245623	-1.295542
C	-2.306878	-0.905534	-1.899761
C	-3.201163	-0.869123	-2.970312
C	-3.628271	0.359588	-3.468125
C	-3.156486	1.531463	-2.881014
C	-2.244458	1.461036	-1.825910

Ca	0.370812	-1.134561	-0.845994
N	-0.193756	-3.472168	-0.809220
C	-0.107202	-4.128790	0.447654
C	-1.217043	-4.141780	1.320140
C	-1.057502	-4.684673	2.596094
C	0.162787	-5.213039	3.006544
C	1.250640	-5.193260	2.139891
C	1.140638	-4.644330	0.859893
C	-2.521718	-3.490101	0.903956
C	-2.522168	-2.020793	1.325114
C	2.334909	-4.584164	-0.072814
C	3.634419	-4.191332	0.631406
C	-0.710745	-4.131379	-1.839646
C	-1.298588	-5.511528	-1.627834
C	-0.767301	-3.625731	-3.153535
C	-0.018766	-2.592183	-3.747678
C	-0.224590	-2.398203	-5.234991
N	0.828196	-1.796532	-3.098568
C	1.831439	-1.128330	-3.854441
C	1.677189	0.207123	-4.273279
C	2.734239	0.831926	-4.943600
C	3.916733	0.157198	-5.213983
C	4.061915	-1.163308	-4.797903
C	3.042109	-1.820697	-4.110063
C	0.405034	0.985436	-4.008282
C	0.679785	2.122665	-3.024408
C	3.226271	-3.249345	-3.631274
C	4.655436	-3.540382	-3.169709
C	-0.218490	1.532256	-5.296006
C	2.804316	-4.268978	-4.696591
C	-3.768144	-4.190083	1.445629
C	2.496196	-5.902587	-0.837577
C	4.455685	-0.561475	0.270009

C	5.690339	-1.188108	0.135230
C	6.596982	-1.106659	1.189567
C	6.268062	-0.404325	2.349047
C	5.025940	0.216534	2.454774
C	4.103335	0.141815	1.412377
C	0.705225	-0.132910	1.565127
C	0.889123	0.834994	2.579770
C	1.551383	0.583788	3.784761
C	2.072978	-0.682171	4.035356
C	1.915527	-1.679951	3.076187
C	1.249274	-1.392508	1.885584
H	-3.570227	4.135284	4.868305
H	-2.596015	2.930739	5.711053
H	-4.046621	2.422069	4.831031
H	-1.369868	4.700594	4.143227
H	0.862283	5.747431	1.710576
H	0.024548	6.060880	3.248156
H	-0.734723	6.460784	1.700262
H	-1.531660	0.829446	4.767074
H	-3.233664	0.879645	6.664360
H	-1.680875	0.144433	7.113481
H	-3.085032	-0.877396	6.787578
H	-1.854178	-2.205193	4.919178
H	-0.429008	-1.295292	5.451835
H	-0.875855	-1.306734	3.746646
H	-4.222514	-1.758247	5.016848
H	-6.143792	-1.905208	3.471503
H	-6.356040	-0.294228	1.611957
H	-3.725267	2.140519	0.699918
H	-6.425600	2.852620	1.902754
H	-5.513425	3.837544	0.738495
H	-4.849448	3.534337	2.353989
H	-4.938676	0.381026	-0.611930
H	-5.387656	2.034094	-1.036474
H	-6.485305	1.063201	-0.051367
H	-2.800639	3.990185	-0.107869
H	-2.734073	5.774052	1.480963
H	-4.186578	6.025269	0.515103
H	-2.750400	7.016988	0.204217
H	-3.531074	6.124529	-2.174717
H	-4.498202	4.761871	-1.606861
H	-3.075757	4.478548	-2.632596
H	-1.365836	6.387001	-2.529374
H	1.035541	6.428167	-3.104816
H	2.620340	5.004330	-1.879057
H	1.624699	2.328586	0.334799
H	3.073227	4.740403	1.523324
H	3.337757	3.051715	1.987434
H	1.771628	3.808947	2.292398
H	3.142223	2.655946	-1.658932
H	3.995311	2.228211	-0.170930
H	4.025291	3.892587	-0.739612
H	0.526836	1.862020	2.459038
H	1.654226	1.378458	4.520525
H	2.588265	-0.892588	4.969093
H	2.293154	-2.683007	3.253059
H	1.155056	-2.244311	1.207595
H	-0.723348	-3.255625	-5.693181
H	-0.857962	-1.514113	-5.382253
H	0.716744	-2.210324	-5.756882
H	-1.368051	-4.208089	-3.843697
H	-2.197424	-5.469317	-1.002587
H	-1.559373	-5.980481	-2.578912
H	-0.587752	-6.155138	-1.100458
H	-0.324863	0.306011	-3.556577
H	-0.375096	0.735974	-6.031314

H	-1.188016	1.990446	-5.073099
H	0.417906	2.295454	-5.759736
H	1.494206	2.761940	-3.379105
H	-0.197333	2.757376	-2.880798
H	0.977444	1.740591	-2.039554
H	2.619759	1.863989	-5.266243
H	4.724256	0.655833	-5.744020
H	4.991666	-1.685963	-5.003882
H	2.557156	-3.379645	-2.774069
H	3.389975	-4.134349	-5.614411
H	2.967935	-5.290743	-4.332976
H	1.745933	-4.172226	-4.949345
H	4.998323	-2.789813	-2.451899
H	4.704260	-4.523273	-2.689002
H	5.362543	-3.557386	-4.007418
H	2.115360	-3.808705	-0.815208
H	1.615835	-6.112422	-1.452283
H	3.366266	-5.862953	-1.502406
H	2.635696	-6.740128	-0.143209
H	3.976313	-4.964534	1.329772
H	4.428879	-4.045017	-0.107829
H	3.523182	-3.255709	1.188006
H	2.203789	-5.601153	2.466159
H	0.266712	-5.632378	4.003888
H	-1.898438	-4.689870	3.283537
H	-2.578268	-3.516829	-0.188063
H	-3.844189	-4.096150	2.534678
H	-4.666966	-3.727550	1.023355
H	-3.773977	-5.256539	1.193089
H	-1.591767	-1.525101	1.025047
H	-3.362939	-1.478628	0.883948
H	-2.591315	-1.937126	2.410014
H	-2.020634	-1.898991	-1.549847
H	-3.556020	-1.797488	-3.411915
H	-4.325504	0.405723	-4.301087
H	-3.493780	2.494874	-3.252968
H	-1.877792	2.418907	-1.440402
H	3.128483	0.604213	1.499254
Cl	3.342983	-0.652593	-1.094720
H	5.935858	-1.725938	-0.773320
H	7.563749	-1.593875	1.098755
H	6.979794	-0.343136	3.167373
H	4.752576	0.757585	3.355192

180

[TS-BC-Cl] scf done: -4543.150914 / Sum of electronic and thermal Energies= -4541.502735 / Sum of electronic and thermal Enthalpies= -4541.501791 / Sum of electronic and thermal Free Energies= -4541.714738

C	5.607845	12.222150	8.441574
C	4.323186	11.988948	8.991814
C	3.175527	12.556453	8.389146
C	3.340185	13.457817	7.334219
C	4.603589	13.770714	6.851688
C	5.716672	13.132433	7.384923
N	4.130828	11.162221	10.144633
C	4.080524	11.818153	11.310277
C	4.518354	13.271289	11.373387
C	1.780380	12.145754	8.830414
C	0.758573	12.201719	7.692328
C	6.838147	11.442093	8.892362
C	8.037996	11.598935	7.955989
C	3.553676	11.323829	12.518857
C	2.643124	10.267963	12.777767
C	1.755158	10.529627	13.978154
N	2.492966	9.182162	12.038366
C	1.458606	8.244345	12.330862
C	1.566091	7.302575	13.383470
C	0.575514	6.323268	13.508780

C	-0.490871	6.241662	12.622650
C	-0.597337	7.179795	11.603886
C	0.350385	8.193878	11.455150
C	2.703168	7.301091	14.392744
C	2.200273	7.637553	15.804763
C	0.162408	9.232114	10.367205
C	0.327335	8.626455	8.970223
C	3.427630	5.949326	14.443319
C	-1.188060	9.942480	10.502962
C	1.252412	12.944070	10.029593
C	7.307927	11.712874	10.326424
Ca	3.906847	8.793117	10.126072
C	3.915543	7.814365	7.796304
C	3.024298	6.988002	7.068535
C	2.224295	7.460109	6.027458
C	2.252153	8.812322	5.679550
C	3.105891	9.672158	6.362992
C	3.921300	9.161803	7.377243
Ca	5.949009	6.275123	7.982544
N	5.114457	4.043837	8.202667
C	5.109868	3.453232	9.494639
C	4.064184	3.727062	10.401863
C	4.128136	3.215928	11.699398
C	5.204362	2.438137	12.106195
C	6.231774	2.165511	11.207383
C	6.209401	2.660845	9.901005
C	2.922090	4.636486	10.008564
C	3.124380	6.008966	10.647320
C	7.342188	2.368615	8.934318
C	8.629293	1.924621	9.627541
C	4.380482	3.487672	7.239225
C	3.479193	2.313248	7.568417
C	4.359863	3.904811	5.895485

C	5.231735	4.761705	5.187618
C	5.044006	4.769905	3.684246
N	6.154821	5.526954	5.749476
C	7.213109	6.057374	4.960920
C	7.150520	7.344763	4.389722
C	8.259646	7.811974	3.676692
C	9.410234	7.047430	3.539481
C	9.470790	5.788356	4.128580
C	8.389014	5.277024	4.845459
C	5.921928	8.229908	4.501933
C	6.266133	9.600333	5.095017
C	8.456005	3.891774	5.462840
C	9.849431	3.526012	5.979356
C	5.242224	8.418336	3.139566
C	7.965901	2.823166	4.476715
C	1.539332	4.092893	10.370116
C	6.942652	1.331860	7.876283
C	8.700267	7.006694	9.220351
C	9.120003	5.717238	8.810473
C	10.140957	5.074427	9.498185
C	10.831892	5.708094	10.531772
C	10.520117	7.041440	10.818445
C	9.514793	7.711889	10.142482
C	6.939586	6.966451	10.270676
C	6.503386	8.220843	10.748626
C	6.073443	8.417263	12.068631
C	6.022884	7.324019	12.935629
C	6.420849	6.062271	12.482532
C	6.893279	5.896446	11.181719
H	1.270789	11.504943	13.864763
H	2.353142	10.569424	14.895189
H	0.981477	9.770719	14.094071
H	3.608698	12.038495	13.334734

H	5.391865	13.466195	10.750995
H	4.737230	13.566027	12.402563
H	3.717211	13.917641	10.997788
H	3.426478	8.064110	14.085638
H	1.644200	8.576772	15.837268
H	3.043627	7.714709	16.501213
H	1.536331	6.847423	16.174563
H	2.744500	5.147897	14.746319
H	4.238195	5.979704	15.181063
H	3.863997	5.676234	13.480949
H	0.648355	5.600451	14.317148
H	-1.236930	5.459315	12.731522
H	-1.439502	7.134491	10.917901
H	0.938490	9.990051	10.507509
H	-2.025407	9.250422	10.356857
H	-1.281578	10.735763	9.753268
H	-1.294834	10.394296	11.494879
H	1.304193	8.149947	8.828467
H	0.229335	9.389008	8.191844
H	-0.427346	7.852942	8.785216
H	1.862241	11.099127	9.144046
H	1.824978	12.745021	10.937849
H	0.209302	12.675307	10.233363
H	1.286777	14.020417	9.821240
H	0.520473	13.232854	7.406176
H	-0.179486	11.737116	8.013751
H	1.112056	11.670537	6.803573
H	2.467711	13.913538	6.876283
H	4.720500	14.481651	6.038080
H	6.691818	13.342276	6.960961
H	6.547341	10.383382	8.832811
H	7.596268	12.763884	10.445743
H	8.191438	11.101775	10.544604
H	6.548327	11.476396	11.072375
H	7.783764	11.377665	6.915726
H	8.826929	10.904171	8.256909
H	8.451580	12.613807	8.004888
H	6.606681	9.092867	10.101010
H	5.786273	9.409489	12.421166
H	5.696923	7.460424	13.961085
H	6.373839	5.206393	13.151068
H	7.260277	4.917593	10.876664
H	4.675308	3.808044	3.318316
H	4.297736	5.532531	3.430113
H	5.968014	5.023529	3.160696
H	3.649181	3.374487	5.270232
H	2.562560	2.656807	8.061833
H	3.192205	1.771246	6.664628
H	3.969383	1.620978	8.258114
H	5.203604	7.737217	5.167302
H	5.017630	7.460444	2.662224
H	4.304170	8.972312	3.259623
H	5.886846	8.985314	2.456705
H	7.043735	10.102979	4.508778
H	5.385981	10.248997	5.109544
H	6.629915	9.511388	6.121562
H	8.217395	8.796485	3.217758
H	10.259815	7.431673	2.980945
H	10.375469	5.195409	4.029689
H	7.760580	3.884517	6.311183
H	8.569111	2.841983	3.560752
H	8.046943	1.823690	4.919564
H	6.920756	2.980749	4.199943
H	10.275469	4.320404	6.599944
H	9.795268	2.609418	6.578043
H	10.548399	3.330348	5.157434

H	7.551259	3.306255	8.404454
H	6.153922	1.702999	7.218617
H	7.805626	1.082510	7.248017
H	6.595529	0.407700	8.354164
H	8.526667	0.924620	10.066376
H	9.443549	1.871624	8.896880
H	8.927869	2.620235	10.418004
H	7.070779	1.560402	11.534686
H	5.245758	2.044145	13.118353
H	3.326713	3.436367	12.399624
H	2.960778	4.770624	8.926420
H	1.404125	4.024258	11.454573
H	0.759621	4.764947	9.995270
H	1.378014	3.097038	9.944256
H	4.165684	6.332284	10.512690
H	2.413641	6.718593	10.206416
H	2.925222	5.972566	11.717683
H	2.962951	5.923109	7.291484
H	1.570909	6.774824	5.491383
H	1.619048	9.186509	4.878696
H	3.162611	10.726328	6.104721
H	4.613943	9.874801	7.829872
H	9.277107	8.743195	10.378639
Cl	8.080435	8.068948	7.762471
H	8.644479	5.230293	7.963105
H	10.422774	4.073044	9.187625
H	11.633735	5.199204	11.056982
H	11.086169	7.581220	11.574351

180

[C-Cl] scf done: -4543.318946 / Sum of electronic and thermal Energies= -4541.666824 / Sum of electronic and thermal Enthalpies= -4541.665880 / Sum of electronic and thermal Free Energies= -4541.882467

C	1.351994	4.405250	-0.200600
C	-0.044473	4.358032	0.023488
C	-0.943177	4.922045	-0.911685
C	-0.421579	5.598684	-2.017937
C	0.949459	5.705716	-2.215454
C	1.820532	5.098791	-1.319684
N	-0.575939	3.696127	1.169095
C	-0.684198	4.381596	2.298823
C	-0.216140	5.826515	2.382489
C	-2.449738	4.801842	-0.729272
C	-3.228454	5.011066	-2.029394
C	2.342618	3.704141	0.715943
C	3.559878	3.168295	-0.044032
C	-1.321410	3.934623	3.478850
C	-2.204822	2.864556	3.726429
C	-3.034438	3.034943	4.984891
N	-2.360418	1.793490	2.955865
C	-3.454596	0.903452	3.159524
C	-3.483547	-0.099264	4.160539
C	-4.576430	-0.973333	4.204273
C	-5.619223	-0.890399	3.293405
C	-5.595161	0.104826	2.322026
C	-4.537008	1.010178	2.246080
C	-2.417248	-0.252671	5.234220
C	-2.994904	0.017938	6.633029
C	-4.612245	2.176873	1.278759
C	-5.318372	1.857311	-0.035992
C	-1.782874	-1.652452	5.245293
C	-5.274125	3.373492	1.975345
C	-3.001748	5.751760	0.343446
C	2.825494	4.571149	1.886441
Ca	-1.146617	1.406337	0.900201
C	-2.205505	0.086856	-1.026103
C	-3.181717	-0.857280	-1.422539
C	-4.040783	-0.670647	-2.508396

C	-3.989002	0.514407	-3.240378
C	-3.065895	1.491206	-2.879921
C	-2.192937	1.263313	-1.812505
Ca	-0.048636	-1.312308	-1.150022
N	-0.199640	-3.651875	-0.847034
C	-0.141890	-4.240893	0.444146
C	-1.262971	-4.238734	1.303077
C	-1.120265	-4.705146	2.613829
C	0.099459	-5.173853	3.080743
C	1.199680	-5.181304	2.229022
C	1.106224	-4.721493	0.913854
C	-2.594698	-3.659701	0.874210
C	-2.814560	-2.308132	1.557736
C	2.318305	-4.742998	-0.000935
C	3.646944	-4.763366	0.757780
C	-0.650516	-4.353763	-1.888436
C	-1.340338	-5.680437	-1.643594
C	-0.529873	-3.958829	-3.235577
C	0.305763	-2.985098	-3.839690
C	0.502552	-3.165458	-5.331337
N	0.890744	-1.990487	-3.190720
C	1.965030	-1.256905	-3.761132
C	1.787066	0.110627	-4.088974
C	2.889093	0.843264	-4.531603
C	4.143477	0.257893	-4.670754
C	4.306044	-1.084362	-4.359186
C	3.238569	-1.857968	-3.891793
C	0.416448	0.760683	-4.034781
C	0.458856	2.274765	-3.839162
C	3.499086	-3.312296	-3.542132
C	4.588674	-3.439649	-2.474139
C	-0.407923	0.396738	-5.275049
C	3.891248	-4.125858	-4.782199

C	-3.772162	-4.596979	1.155644
C	2.252948	-5.931728	-0.970446
C	4.179820	-0.207175	1.174064
C	4.275244	-0.468760	-0.198944
C	5.481831	-0.294995	-0.869423
C	6.614387	0.125247	-0.174041
C	6.533734	0.374333	1.196068
C	5.322692	0.215880	1.864235
C	2.869473	-0.289052	1.848538
C	2.413529	0.784484	2.623989
C	1.114296	0.801569	3.128693
C	0.268369	-0.286163	2.893722
C	0.737934	-1.402642	2.191701
C	2.024918	-1.393205	1.660717
H	-3.287675	4.086660	5.140993
H	-2.464429	2.700441	5.858983
H	-3.957761	2.453616	4.942001
H	-1.299323	4.668073	4.279725
H	0.489854	6.090746	1.596169
H	0.240395	6.025182	3.356595
H	-1.080303	6.493002	2.287869
H	-1.631290	0.486508	5.040013
H	-3.518298	0.974721	6.688896
H	-2.195597	0.016455	7.383647
H	-3.710631	-0.764683	6.910165
H	-2.520087	-2.408413	5.540563
H	-0.962887	-1.690617	5.971589
H	-1.379968	-1.952711	4.275863
H	-4.603468	-1.739533	4.975085
H	-6.449673	-1.589557	3.343973
H	-6.418564	0.189989	1.619471
H	-3.587508	2.491391	1.044105
H	-6.274234	3.098485	2.331131

H	-5.376416	4.214533	1.281806
H	-4.686852	3.711478	2.833646
H	-4.921280	0.949051	-0.495055
H	-5.176413	2.681384	-0.742462
H	-6.398945	1.732546	0.104766
H	-2.657076	3.777739	-0.390041
H	-2.747411	5.418325	1.351126
H	-4.094633	5.801322	0.279012
H	-2.611631	6.765528	0.195686
H	-3.250626	6.069145	-2.317502
H	-4.266292	4.687188	-1.897329
H	-2.797893	4.446621	-2.861292
H	-1.095617	6.050423	-2.738232
H	1.336760	6.240904	-3.078606
H	2.889596	5.150724	-1.499774
H	1.816444	2.845162	1.139739
H	3.204280	5.538016	1.532826
H	3.648170	4.065351	2.408145
H	2.037371	4.758700	2.617691
H	3.264106	2.614263	-0.937691
H	4.128451	2.486603	0.595759
H	4.240936	3.977173	-0.338174
H	3.062672	1.641612	2.772911
H	0.746813	1.668182	3.675107
H	-0.741261	-0.272688	3.286635
H	0.112535	-2.283471	2.080548
H	2.385009	-2.250554	1.100603
H	-0.470192	-3.110766	-5.834109
H	1.158769	-2.401949	-5.752190
H	0.921637	-4.152534	-5.549761
H	-1.006174	-4.629757	-3.944158
H	-2.356422	-5.516418	-1.266132
H	-1.409052	-6.272341	-2.558910
H	-0.810123	-6.261030	-0.883122
H	-0.126383	0.350764	-3.175238
H	-0.530530	-0.685359	-5.367530
H	-1.406041	0.843647	-5.209702
H	0.081149	0.765365	-6.184875
H	0.870337	2.787166	-4.716846
H	-0.555261	2.659110	-3.697548
H	1.046849	2.552974	-2.960362
H	2.765298	1.892640	-4.776144
H	4.986176	0.847384	-5.022388
H	5.283418	-1.546985	-4.475394
H	2.578908	-3.738179	-3.130103
H	4.859250	-3.793996	-5.176296
H	3.980023	-5.189692	-4.531397
H	3.156681	-4.022429	-5.585273
H	4.316867	-2.911217	-1.556877
H	4.761678	-4.493222	-2.228765
H	5.541625	-3.025160	-2.821797
H	2.276867	-3.832304	-0.614840
H	1.401351	-5.850512	-1.649830
H	3.161443	-5.979813	-1.581076
H	2.166650	-6.873783	-0.415732
H	3.804825	-5.724473	1.261286
H	4.479343	-4.622486	0.063900
H	3.704377	-3.974394	1.515613
H	2.152170	-5.542243	2.602381
H	0.197781	-5.525980	4.103989
H	-1.978633	-4.685446	3.280196
H	-2.548250	-3.498534	-0.207187
H	-3.937309	-4.722602	2.231718
H	-4.693737	-4.183098	0.730546
H	-3.607463	-5.590681	0.725416
H	-2.037127	-1.588412	1.276396

H	-3.776640	-1.860723	1.294171
H	-2.792572	-2.418296	2.643756
H	-3.284146	-1.788511	-0.871859
H	-4.756707	-1.445418	-2.776079
H	-4.658455	0.673622	-4.081949
H	-3.010662	2.416300	-3.445999
H	-1.440193	2.040147	-1.633100
H	5.258461	0.412018	2.931729
Cl	1.067096	0.955668	-0.568101
H	3.386046	-0.738569	-0.759340
H	5.519868	-0.466942	-1.940564
H	7.556148	0.264520	-0.698239
H	7.414511	0.696756	1.745105

158

[D] scf done: -4080.139486 / Sum of electronic and thermal Energies= -4078.683932 / Sum of electronic and thermal Enthalpies= -4078.682988 / Sum of electronic and thermal Free Energies= -4078.877642

C	0.964476	3.769444	-0.594897
C	-0.175532	4.144544	0.149418
C	-1.029229	5.167304	-0.333434
C	-0.722512	5.788153	-1.544850
C	0.403918	5.427934	-2.278562
C	1.237662	4.425985	-1.798734
N	-0.556913	3.412936	1.304710
C	-0.424922	3.987554	2.502231
C	0.417952	5.239411	2.617787
C	-2.306063	5.543080	0.398384
C	-3.523708	4.975630	-0.338900
C	1.877722	2.649862	-0.139224
C	1.973233	1.556219	-1.207339
C	-1.052958	3.553368	3.679764
C	-2.038037	2.557938	3.877742
C	-2.741584	2.610977	5.216870
N	-2.353665	1.638616	2.982527
C	-3.374686	0.683042	3.216694
C	-3.146015	-0.441778	4.043555
C	-4.170792	-1.379793	4.191867
C	-5.388309	-1.235838	3.534300
C	-5.585330	-0.150212	2.688826
C	-4.590239	0.812761	2.506367
C	-1.816703	-0.675219	4.735364
C	-1.972708	-0.769387	6.257951
C	-4.837887	1.992002	1.584002
C	-5.209014	1.544877	0.166265
C	-1.140307	-1.940045	4.195121
C	-5.900185	2.927836	2.170308
C	-2.449685	7.054808	0.598387
C	3.272405	3.159353	0.235607
Ca	-1.250577	1.203702	0.981001
C	-1.678419	0.442998	-1.403811
C	-2.402550	-0.499637	-2.167913
C	-3.310789	-0.145923	-3.165278
C	-3.554433	1.201214	-3.434120
C	-2.863319	2.173323	-2.716911
C	-1.940978	1.788290	-1.740652
Ca	0.222901	-1.188487	-1.103644
N	-0.383171	-3.443136	-0.998976
C	-0.651312	-3.973387	0.297249
C	-1.964763	-4.039046	0.807851
C	-2.175307	-4.624177	2.057560
C	-1.113565	-5.116409	2.806602
C	0.185287	-4.973495	2.332235
C	0.441075	-4.390164	1.089275
C	-3.116516	-3.393431	0.067347
C	-3.310713	-1.976082	0.616447
C	1.864649	-4.207910	0.596063
C	2.848747	-3.930580	1.733078

C	-0.512704	-4.257754	-2.044617
C	-1.138599	-5.622821	-1.841552
C	-0.069190	-3.977870	-3.352823
C	0.772051	-2.952080	-3.834932
C	1.420325	-3.219852	-5.177072
N	1.071763	-1.844382	-3.171722
C	2.079591	-0.976716	-3.669697
C	1.715995	0.221816	-4.329922
C	2.734839	1.075301	-4.759945
C	4.075247	0.786208	-4.520637
C	4.415615	-0.368543	-3.828109
C	3.434179	-1.263109	-3.394880
C	0.255222	0.585302	-4.536979
C	0.027748	2.085270	-4.723946
C	3.833800	-2.483144	-2.586309
C	4.312310	-2.059682	-1.192478
C	-0.389091	-0.191989	-5.690515
C	4.902450	-3.327972	-3.286610
C	-4.427793	-4.177180	0.140280
C	2.326437	-5.389732	-0.263558
H	-2.995562	3.644179	5.471897
H	-2.078445	2.234103	6.003895
H	-3.651854	2.008182	5.223536
H	-0.844227	4.157272	4.557346
H	-0.097556	6.098997	2.175902
H	1.358559	5.121119	2.070436
H	0.638732	5.474321	3.661534
H	-1.164696	0.174571	4.509793
H	-2.489645	0.102046	6.671227
H	-0.989833	-0.846626	6.737283
H	-2.547618	-1.659741	6.539625
H	-1.717774	-2.833653	4.453711
H	-0.136824	-2.051619	4.621299
H	-1.034902	-1.919496	3.107979
H	-4.004628	-2.245583	4.827998
H	-6.172694	-1.976450	3.666266
H	-6.529532	-0.046140	2.159911
H	-3.906180	2.568148	1.528331
H	-6.865000	2.415941	2.267753
H	-6.044615	3.801777	1.524750
H	-5.604799	3.279149	3.164779
H	-4.430676	0.917221	-0.281353
H	-5.347965	2.413241	-0.486469
H	-6.142753	0.970628	0.159111
H	-2.283782	5.077165	1.387577
H	-1.574033	7.479694	1.101054
H	-3.332612	7.273870	1.209940
H	-2.572145	7.581146	-0.355262
H	-3.610618	5.408398	-1.343190
H	-4.448281	5.197061	0.206992
H	-3.444861	3.890657	-0.450705
H	-1.381987	6.563633	-1.926610
H	0.625294	5.920860	-3.221332
H	2.116062	4.139107	-2.371874
H	1.447612	2.209786	0.766725
H	3.772231	3.613600	-0.628088
H	3.897074	2.333098	0.594515
H	3.216889	3.913581	1.027547
H	0.969070	1.281491	-1.560435
H	2.495102	0.677521	-0.810264
H	2.525047	1.885066	-2.092379
H	0.805436	-3.890547	-5.782947
H	1.608143	-2.299405	-5.734555
H	2.389930	-3.708154	-5.022155
H	-0.267895	-4.764882	-4.071966
H	-2.180825	-5.530001	-1.517092

H	-1.111301	-6.214396	-2.758861
H	-0.616119	-6.171924	-1.051169
H	-0.286249	0.293170	-3.629268
H	-0.405980	-1.265801	-5.492171
H	-1.425820	0.136476	-5.824234
H	0.151648	-0.018611	-6.629103
H	0.442891	2.446249	-5.673239
H	-1.045863	2.289729	-4.735044
H	0.467251	2.669460	-3.908902
H	2.477326	1.994666	-5.275497
H	4.849399	1.469055	-4.861127
H	5.460908	-0.580371	-3.617079
H	2.946372	-3.110433	-2.455860
H	5.856516	-2.793109	-3.361213
H	5.085339	-4.252122	-2.726183
H	4.594488	-3.599088	-4.301855
H	3.527230	-1.538678	-0.631183
H	4.617342	-2.932092	-0.605227
H	5.169895	-1.380271	-1.261528
H	1.864913	-3.327701	-0.059917
H	1.699678	-5.500931	-1.153090
H	3.360843	-5.242309	-0.597398
H	2.284065	-6.324357	0.308731
H	2.995994	-4.811510	2.368634
H	3.828849	-3.661415	1.326544
H	2.499368	-3.103910	2.360008
H	1.014330	-5.315391	2.943883
H	-1.293432	-5.578481	3.773756
H	-3.184130	-4.682944	2.456088
H	-2.835570	-3.315461	-0.988261
H	-4.840975	-4.183008	1.155173
H	-5.178062	-3.714504	-0.510994
H	-4.293415	-5.217717	-0.175997
H	-2.380588	-1.403342	0.519466
H	-4.091531	-1.435334	0.073060
H	-3.575256	-2.004076	1.676480
H	-2.263941	-1.571235	-1.996749
H	-3.832125	-0.917699	-3.727336
H	-4.268027	1.487210	-4.202809
H	-3.020754	3.228480	-2.925414
H	-1.389759	2.601891	-1.261295
Cl	0.747130	-0.501469	1.404219

170

[E-Cl] scf done: -4326.762147 / Sum of electronic and thermal Energies= -4325.206365 / Sum of electronic and thermal Enthalpies= -4325.205421 / Sum of electronic and thermal Free Energies= -4325.416577

C	0.465280	4.914309	-0.671731
C	-0.833892	4.401824	-0.452928
C	-1.767523	4.337813	-1.516038
C	-1.405706	4.861646	-2.758506
C	-0.150302	5.424608	-2.966409
C	0.774827	5.435672	-1.931837
N	-1.234498	3.904917	0.819887
C	-1.678232	4.771444	1.722178
C	-1.642766	6.269599	1.467118
C	-3.146411	3.734991	-1.307482
C	-3.710543	3.080682	-2.568576
C	1.527936	4.931001	0.413961
C	2.834907	4.283018	-0.064868
C	-2.278676	4.439215	2.953486
C	-2.811819	3.226251	3.416181
C	-3.768426	3.357590	4.585443
N	-2.602471	2.031783	2.866807
C	-3.388249	0.948657	3.345545
C	-2.840633	0.041557	4.281951
C	-3.643786	-1.009543	4.730847
C	-4.931199	-1.200354	4.237304

C	-5.436770	-0.332641	3.277458
C	-4.685858	0.755161	2.825036
C	-1.399979	0.185642	4.747615
C	-1.206395	1.308722	5.773976
C	-5.253121	1.673403	1.757161
C	-5.315387	0.953646	0.404324
C	-0.815743	-1.115125	5.298121
C	-6.632208	2.225123	2.132023
C	-4.136656	4.757986	-0.738761
C	1.829473	6.353174	0.904993
Ca	-1.234984	1.524857	1.022608
Cl	-1.818332	0.325547	-1.278630
Ca	0.475869	-1.043511	-1.074863
N	-0.200909	-3.289524	-1.332634
C	-0.200801	-4.100309	-0.172979
C	-1.128822	-3.870817	0.865350
C	-0.968211	-4.552734	2.076096
C	0.059645	-5.471025	2.252250
C	0.935553	-5.735975	1.200510
C	0.828210	-5.063152	-0.018083
C	-2.307691	-2.936073	0.674888
C	-2.258436	-1.749551	1.631477
C	1.752667	-5.373710	-1.183152
C	3.128127	-5.899661	-0.772699
C	-0.936004	-3.591260	-2.399051
C	-2.014009	-4.650637	-2.303173
C	-0.811369	-2.954219	-3.647476
C	0.186955	-2.069581	-4.108425
C	0.206221	-1.797937	-5.598087
N	1.085197	-1.479050	-3.327117
C	2.162441	-0.789562	-3.941446
C	2.035786	0.571969	-4.281341
C	3.124473	1.224698	-4.864877

C	4.318997	0.552695	-5.100301
C	4.451324	-0.777528	-4.710600
C	3.392350	-1.463071	-4.112945
C	0.777956	1.331689	-3.914572
C	1.006200	2.062968	-2.588386
C	3.550438	-2.880318	-3.597005
C	4.971828	-3.193502	-3.127904
C	0.304762	2.312693	-4.986985
C	3.077001	-3.928855	-4.609899
C	-3.640768	-3.675499	0.842581
C	1.095031	-6.368475	-2.150305
C	3.843649	-1.384026	0.478986
C	3.235123	-2.612261	0.708350
C	3.528552	-3.295399	1.885858
C	4.431159	-2.758781	2.801398
C	5.034624	-1.528449	2.545436
C	4.739011	-0.823724	1.380131
C	0.876555	0.089713	1.227254
C	1.476244	1.361629	1.377626
C	2.249316	1.738543	2.480563
C	2.490683	0.809734	3.488959
C	1.952813	-0.472503	3.378492
C	1.150895	-0.806949	2.286413
H	-4.795124	3.443047	4.210536
H	-3.551059	4.257711	5.166134
H	-3.742093	2.488854	5.246228
H	-2.538477	5.301445	3.559989
H	-1.077103	6.765591	2.263178
H	-2.659084	6.676000	1.499554
H	-1.202844	6.530002	0.505128
H	-0.800885	0.457136	3.867133
H	-1.438396	2.288160	5.351895
H	-0.162480	1.330970	6.108202

H	-1.842418	1.147120	6.652866
H	-1.295863	-1.408537	6.239313
H	0.248584	-0.975928	5.506353
H	-0.913146	-1.942448	4.586861
H	-3.250438	-1.711975	5.458587
H	-5.531522	-2.034298	4.591522
H	-6.433443	-0.497702	2.875680
H	-4.572636	2.524319	1.650225
H	-7.387744	1.431314	2.165249
H	-6.960802	2.961746	1.389730
H	-6.619256	2.711737	3.113000
H	-4.330703	0.605488	0.075560
H	-5.707342	1.622031	-0.371022
H	-5.973547	0.077869	0.458116
H	-3.047676	2.946033	-0.551930
H	-3.821353	5.115800	0.244077
H	-5.129093	4.304464	-0.628559
H	-4.228958	5.621895	-1.407769
H	-4.000236	3.826905	-3.318332
H	-4.607994	2.506225	-2.317071
H	-2.988843	2.390717	-3.014961
H	-2.116414	4.830278	-3.578283
H	0.109762	5.833168	-3.939061
H	1.763484	5.853060	-2.104384
H	1.140282	4.354958	1.262069
H	2.224208	6.967850	0.086973
H	2.583869	6.330410	1.700560
H	0.939378	6.849964	1.295376
H	2.678348	3.280103	-0.470139
H	3.549796	4.209261	0.762831
H	3.308165	4.884214	-0.849698
H	1.346851	2.111347	0.590285
H	2.664061	2.741699	2.545501
H	3.098052	1.077683	4.350077
H	2.156513	-1.211422	4.148641
H	0.726969	-1.812454	2.266347
H	-0.033793	-0.746256	-5.793902
H	1.203229	-1.973219	-6.013853
H	-0.514354	-2.423789	-6.129054
H	-1.518900	-3.283329	-4.400979
H	-2.965298	-4.168644	-2.045942
H	-2.152195	-5.169228	-3.255561
H	-1.795465	-5.383698	-1.523163
H	-0.027577	0.606473	-3.758620
H	0.178060	1.812670	-5.953791
H	-0.653875	2.751185	-4.692113
H	1.010028	3.140617	-5.123009
H	1.780966	2.827639	-2.698662
H	0.091112	2.545912	-2.239901
H	1.361899	1.382606	-1.801152
H	3.035743	2.273720	-5.135066
H	5.154017	1.069628	-5.566359
H	5.400784	-1.283703	-4.858545
H	2.886510	-2.953866	-2.727051
H	3.628376	-3.834367	-5.553472
H	3.245091	-4.939833	-4.219389
H	2.009074	-3.829366	-4.820353
H	5.342777	-2.429225	-2.437223
H	4.989112	-4.158208	-2.609601
H	5.673649	-3.264514	-3.967680
H	1.888774	-4.437843	-1.738759
H	0.183264	-5.960744	-2.590258
H	1.779880	-6.610541	-2.971263
H	0.840137	-7.300069	-1.630568
H	3.062463	-6.894099	-0.315093
H	3.762308	-5.997893	-1.660259

H	3.638096	-5.233954	-0.070421
H	1.716950	-6.479094	1.333549
H	0.169246	-5.995158	3.198220
H	-1.671002	-4.368354	2.885305
H	-2.268400	-2.541818	-0.345200
H	-3.771914	-4.021901	1.874335
H	-4.475085	-3.003333	0.611487
H	-3.704262	-4.548420	0.185325
H	-1.318632	-1.195558	1.524538
H	-3.101647	-1.077005	1.447282
H	-2.326903	-2.072026	2.672619
H	5.181199	0.145693	1.180289
Cl	3.457934	-0.510283	-0.996741
H	2.541122	-3.037802	-0.009557
H	3.028425	-4.238643	2.081206
H	4.658125	-3.294799	3.718484
H	5.729990	-1.100924	3.262166

170

[TS-EF-Cl]scf done: -4326.715627 / Sum of electronic and thermal Energies= -4325.161011 / Sum of electronic and thermal Enthalpies= -4325.160067 / Sum of electronic and thermal Free Energies= -4325.369543

C	5.653164	12.482747	8.715202
C	4.428997	11.806907	8.908857
C	3.548132	11.606186	7.818990
C	3.865529	12.178249	6.585536
C	5.042784	12.895279	6.401218
C	5.935144	13.021423	7.455918
N	4.028506	11.325467	10.195588
C	3.367105	12.198313	10.960459
C	3.304373	13.653962	10.543418
C	2.275859	10.790795	7.970122
C	1.972779	9.958072	6.722812
C	6.699988	12.606641	9.807120
C	7.979197	11.877078	9.376061
C	2.635335	11.883447	12.119109
C	2.140465	10.649132	12.586403
C	0.949923	10.782279	13.518000
N	2.577094	9.450441	12.214636
C	1.814262	8.316711	12.623980
C	2.211204	7.572095	13.759567
C	1.452360	6.459460	14.129340
C	0.338881	6.066760	13.393980
C	-0.027051	6.790704	12.267882
C	0.694082	7.918034	11.863948
C	3.433998	7.974632	14.564309
C	3.143735	9.101867	15.563930
C	0.257717	8.658576	10.614161
C	0.353532	7.752689	9.381162
C	4.079952	6.797032	15.297509
C	-1.164641	9.213330	10.754132
C	1.073333	11.661443	8.352435
C	7.043671	14.057087	10.167118
Ca	4.425385	9.081446	10.774677
Cl	4.070746	7.688302	8.595352
Ca	6.129939	6.052455	8.157625
N	4.943554	4.040792	7.910913
C	4.990019	3.146165	9.011848
C	4.184513	3.345122	10.153474
C	4.394342	2.532937	11.273459
C	5.355900	1.531007	11.269443
C	6.123398	1.319280	10.126391
C	5.961588	2.114341	8.991407
C	3.085453	4.389596	10.186712
C	3.323736	5.434538	11.274405
C	6.781077	1.872481	7.737367
C	8.181366	1.321062	8.006713
C	4.077374	3.873739	6.917690

C	2.942319	2.880581	7.058295
C	4.111508	4.582177	5.699870
C	5.116389	5.410834	5.159769
C	4.950507	5.807681	3.708249
N	6.173955	5.846379	5.835421
C	7.220526	6.510647	5.141499
C	7.169927	7.901975	4.923163
C	8.243023	8.522100	4.277651
C	9.349181	7.791979	3.861342
C	9.409610	6.425597	4.121251
C	8.365824	5.767089	4.773520
C	6.021090	8.737244	5.450879
C	6.472292	9.509078	6.695996
C	8.454274	4.292308	5.116904
C	9.888367	3.810029	5.334937
C	5.446121	9.694189	4.404337
C	7.744421	3.415261	4.079088
C	1.706385	3.748852	10.386394
C	6.026425	0.952318	6.768777
C	8.708098	5.825937	9.820808
C	8.312811	4.464317	9.969298
C	8.648520	3.772821	11.121536
C	9.501036	4.331077	12.079692
C	10.076016	5.577603	11.810027
C	9.749319	6.296799	10.672957
C	7.255847	7.018093	10.361167
C	7.502473	8.413580	10.347655
C	7.212505	9.229232	11.445990
C	6.609824	8.679743	12.591726
C	6.323958	7.304404	12.616351
C	6.646970	6.499325	11.525129
H	0.050565	10.985428	12.925852
H	1.088204	11.630945	14.194438
H	0.763921	9.882008	14.104391
H	2.192060	12.742916	12.609972
H	2.742486	14.249184	11.265677
H	2.824505	13.750367	9.563891
H	4.303388	14.080301	10.436179
H	4.163238	8.372246	13.847759
H	2.869647	10.029890	15.059199
H	4.031933	9.303715	16.175012
H	2.326032	8.820822	16.238005
H	3.472363	6.473088	16.150500
H	5.056608	7.091634	15.698260
H	4.219996	5.931101	14.640998
H	1.738776	5.880411	15.001518
H	-0.233729	5.193650	13.694605
H	-0.890828	6.477028	11.687312
H	0.936270	9.504822	10.467991
H	-1.899353	8.402850	10.828509
H	-1.426037	9.818800	9.878868
H	-1.271034	9.838731	11.645651
H	1.377664	7.412374	9.203794
H	0.017188	8.289623	8.487761
H	-0.284907	6.868441	9.493599
H	2.437612	10.089271	8.795264
H	1.205819	12.121755	9.335930
H	0.161386	11.053983	8.389678
H	0.918209	12.458323	7.614804
H	1.668112	10.583362	5.875187
H	1.150937	9.265728	6.926666
H	2.841567	9.363208	6.426724
H	3.183224	12.054717	5.750353
H	5.271609	13.330696	5.432422
H	6.871981	13.551302	7.303182
H	6.297541	12.120788	10.704112

H	7.441125	14.593454	9.297945
H	7.810123	14.081004	10.950742
H	6.176500	14.614373	10.532129
H	7.760213	10.860990	9.040377
H	8.708084	11.833101	10.194465
H	8.458272	12.396283	8.538322
H	7.986527	8.854538	9.478414
H	7.454286	10.288206	11.420743
H	6.408271	9.302373	13.460089
H	5.865834	6.863227	13.494309
H	6.477550	5.424636	11.590949
H	4.722466	6.877241	3.634129
H	5.877126	5.645752	3.149395
H	4.143574	5.248835	3.229235
H	3.294966	4.355040	5.022516
H	2.044954	3.404354	7.409447
H	2.699107	2.417737	6.098157
H	3.173968	2.098644	7.784931
H	5.217921	8.058165	5.756551
H	5.164173	9.162043	3.489069
H	4.560624	10.198003	4.802185
H	6.167569	10.472766	4.131125
H	7.251669	10.235453	6.440015
H	5.636550	10.044871	7.152581
H	6.899235	8.834278	7.448433
H	8.211749	9.595001	4.104544
H	10.172707	8.287372	3.353354
H	10.292557	5.867215	3.825612
H	7.914609	4.168234	6.063194
H	8.175410	3.567701	3.081920
H	7.853849	2.355102	4.337188
H	6.674998	3.635846	4.031637
H	10.418402	4.446830	6.050446
H	9.880736	2.788593	5.729619
H	10.460185	3.789966	4.399216
H	6.885216	2.841976	7.235709
H	5.069641	1.385814	6.468337
H	6.617546	0.783697	5.861333
H	5.829676	-0.020440	7.235945
H	8.148537	0.301538	8.409538
H	8.745448	1.276059	7.069182
H	8.742063	1.948658	8.706643
H	6.866205	0.527050	10.120706
H	5.503287	0.909599	12.149011
H	3.782177	2.685591	12.159280
H	3.083550	4.911061	9.224241
H	1.631734	3.277013	11.373275
H	0.924202	4.513952	10.328334
H	1.503454	2.981284	9.633438
H	4.285810	5.933360	11.134519
H	2.530633	6.185509	11.256919
H	3.327134	4.984640	12.273041
H	10.183235	7.272426	10.483228
Cl	8.901452	6.365840	8.021570
H	7.674567	3.986547	9.230513
H	8.244893	2.772454	11.253317
H	9.765454	3.778909	12.975684
H	10.809689	5.996299	12.495246

170

[F-Cl] scf done: -4326.890168 / Sum of electronic and thermal Energies= -4325.332041 / Sum of electronic and thermal Enthalpies= -4325.331097 / Sum of electronic and thermal Free Energies= -4325.539830

C	1.154365	4.507961	-1.294299
C	-0.186222	4.436984	-0.853001
C	-1.237621	4.822635	-1.723854
C	-0.923603	5.292194	-3.000907
C	0.396059	5.385948	-3.431431

C	1.418165	4.992724	-2.578949
N	-0.530166	3.915799	0.429845
C	-0.733550	4.751765	1.447922
C	-0.238399	6.185988	1.374209
C	-2.685655	4.762878	-1.271801
C	-3.652966	4.385875	-2.395274
C	2.322421	4.133997	-0.404685
C	3.337585	3.229464	-1.107632
C	-1.391264	4.435401	2.652622
C	-2.209691	3.338186	3.017194
C	-3.135253	3.623483	4.185064
N	-2.200794	2.154146	2.425292
C	-3.203132	1.186403	2.740090
C	-3.092813	0.328064	3.853264
C	-4.082859	-0.635803	4.067029
C	-5.156548	-0.775712	3.199664
C	-5.246933	0.058725	2.091053
C	-4.285084	1.037680	1.843021
C	-1.923468	0.383878	4.814674
C	-2.351848	0.768784	6.235222
C	-4.418928	1.953110	0.644263
C	-5.118514	1.295115	-0.544872
C	-1.186308	-0.960737	4.836600
C	-5.125339	3.255071	1.041171
C	-3.125343	6.090761	-0.641514
C	3.031015	5.391000	0.117072
Ca	-0.467792	1.588447	0.873289
Cl	-1.439332	-0.938703	0.900127
Ca	0.470051	-1.948039	-0.624621
N	-0.176861	-4.126730	-1.220918
C	0.123538	-5.061726	-0.196850
C	-0.755611	-5.227642	0.898225
C	-0.391153	-6.112819	1.917735

C	0.816044	-6.802861	1.880463
C	1.682285	-6.615917	0.807522
C	1.356307	-5.753779	-0.240665
C	-2.070126	-4.474504	0.979544
C	-2.325363	-3.895909	2.374600
C	2.329581	-5.542270	-1.386197
C	3.528530	-4.702128	-0.934912
C	-0.676339	-4.575735	-2.368105
C	-1.047182	-6.037862	-2.502843
C	-0.819075	-3.807486	-3.536320
C	-0.305710	-2.530815	-3.836135
C	-0.392015	-2.101966	-5.286270
N	0.257701	-1.719303	-2.950264
C	0.871388	-0.521601	-3.413770
C	0.109989	0.642060	-3.640100
C	0.766428	1.818844	-4.002561
C	2.144880	1.846819	-4.170991
C	2.891542	0.699077	-3.930341
C	2.277936	-0.486150	-3.523810
C	-1.389529	0.659414	-3.419635
C	-1.700087	1.232179	-2.032701
C	3.112795	-1.704800	-3.177497
C	4.381073	-1.343486	-2.400443
C	-2.141638	1.429338	-4.507279
C	3.433808	-2.539710	-4.421399
C	-3.249248	-5.353118	0.544006
C	2.804221	-6.861042	-2.003525
C	2.838190	-1.061910	2.714226
C	1.736785	-1.912046	2.511117
C	1.906402	-3.288029	2.375405
C	3.186952	-3.843005	2.432380
C	4.287151	-3.011914	2.624003
C	4.114133	-1.636270	2.767248

C	2.646874	0.394575	2.866424
C	3.621890	1.301989	2.432270
C	3.420729	2.674341	2.537518
C	2.229683	3.177224	3.059527
C	1.252228	2.288228	3.511697
C	1.464149	0.910690	3.421156
H	-3.614980	4.599399	4.065944
H	-2.554670	3.658404	5.114765
H	-3.905948	2.859280	4.293101
H	-1.451315	5.269697	3.346443
H	-0.963425	6.880588	1.807201
H	-0.018509	6.493896	0.350853
H	0.682785	6.275997	1.961957
H	-1.234836	1.152367	4.448206
H	-2.892832	1.718656	6.254276
H	-1.476636	0.857769	6.890189
H	-3.010893	0.004035	6.662716
H	-1.814460	-1.744508	5.274354
H	-0.269871	-0.902414	5.436448
H	-0.933132	-1.282312	3.823021
H	-4.002960	-1.294030	4.928468
H	-5.915369	-1.532529	3.380855
H	-6.081106	-0.058052	1.406238
H	-3.403557	2.223685	0.329019
H	-6.113558	3.038198	1.464015
H	-5.262363	3.906233	0.171124
H	-4.548297	3.808328	1.787306
H	-4.675120	0.320273	-0.775101
H	-5.035318	1.933012	-1.430983
H	-6.188421	1.146388	-0.357468
H	-2.746482	3.995437	-0.493105
H	-2.538663	6.330634	0.246874
H	-4.179072	6.043638	-0.342882
H	-3.010038	6.912727	-1.358580
H	-3.738115	5.181509	-3.144595
H	-4.656704	4.226772	-1.987157
H	-3.343850	3.471657	-2.908359
H	-1.724353	5.594927	-3.669078
H	0.623099	5.757663	-4.427333
H	2.449758	5.060084	-2.913054
H	1.922805	3.587883	0.455529
H	3.440653	5.971271	-0.718615
H	3.867481	5.121350	0.772750
H	2.357502	6.045989	0.673990
H	2.853562	2.385885	-1.599681
H	4.047167	2.827058	-0.375929
H	3.914791	3.784745	-1.857856
H	4.523031	0.930373	1.954614
H	4.185822	3.355382	2.178074
H	2.056633	4.247687	3.112408
H	0.328852	2.675408	3.933989
H	0.713890	0.228935	3.803443
H	-1.077879	-1.255930	-5.403849
H	0.583881	-1.761024	-5.646587
H	-0.739888	-2.918431	-5.922650
H	-1.274543	-4.327781	-4.371734
H	-1.417073	-6.457133	-1.564790
H	-1.800021	-6.178448	-3.282743
H	-0.162661	-6.620953	-2.787265
H	-1.751003	-0.373798	-3.425420
H	-1.892148	1.051689	-5.505153
H	-3.223914	1.330271	-4.363672
H	-1.901267	2.497793	-4.486596
H	-1.230442	2.215471	-1.912184
H	-2.776816	1.333372	-1.871623
H	-1.329842	0.531476	-1.276487

H	0.197880	2.733798	-4.136372
H	2.637751	2.771905	-4.455588
H	3.972591	0.735867	-4.029297
H	2.496152	-2.348905	-2.535231
H	4.002602	-1.947293	-5.148204
H	4.031332	-3.420897	-4.157000
H	2.516556	-2.886050	-4.907697
H	4.144118	-0.712802	-1.536949
H	4.880831	-2.250553	-2.043530
H	5.099765	-0.803729	-3.027821
H	1.810219	-4.977519	-2.166292
H	1.958644	-7.490888	-2.299007
H	3.413658	-6.666408	-2.893619
H	3.419479	-7.437981	-1.303039
H	4.103393	-5.220608	-0.159046
H	4.198836	-4.501171	-1.777848
H	3.220101	-3.740419	-0.509491
H	2.628976	-7.150639	0.783510
H	1.082334	-7.481384	2.686624
H	-1.062611	-6.256650	2.759712
H	-2.012709	-3.637509	0.276768
H	-3.326546	-6.243357	1.180525
H	-4.188374	-4.793073	0.625770
H	-3.145630	-5.685515	-0.493110
H	-1.449222	-3.361649	2.752940
H	-3.151305	-3.178874	2.342105
H	-2.583983	-4.679895	3.097471
H	4.978930	-1.005422	2.952360
Cl	1.640516	0.415549	-0.254005
H	0.737550	-1.490039	2.453771
H	1.044809	-3.935179	2.239946
H	3.310000	-4.915724	2.329881
H	5.287156	-3.434388	2.676708

12

[Bromobenzene] scf done: -244.983966 / Sum of electronic and thermal Energies= -244.887300 / Sum of electronic and thermal Enthalpies= -244.886356 / Sum of electronic and thermal Free Energies= -244.923932

C	-1.249978	-2.456884	6.354705
C	-2.291802	-1.615230	5.974278
C	-2.165564	-0.245531	6.200453
C	-1.016053	0.269621	6.796840
C	0.015616	-0.589924	7.170109
C	-0.093388	-1.962391	6.951619
Br	-1.411951	-4.332911	6.051189
H	0.705424	-2.637317	7.239675
H	0.914683	-0.195452	7.635543
H	-0.924436	1.337930	6.970151
H	-2.973793	0.418546	5.906622
H	-3.183497	-2.023047	5.510505

180

[B-Br] scf done: -4541.588327 / Sum of electronic and thermal Energies= -4539.938243 / Sum of electronic and thermal Enthalpies= -4539.937299 / Sum of electronic and thermal Free Energies= -4540.154574

C	1.072263	4.250533	-0.424318
C	-0.295084	4.309083	-0.063894
C	-1.225035	5.018046	-0.859768
C	-0.750196	5.762152	-1.943302
C	0.604869	5.794950	-2.246889
C	1.497375	5.028129	-1.505434
N	-0.780431	3.636183	1.088931
C	-0.887237	4.374155	2.197245
C	-0.269776	5.762172	2.235996
C	-2.718870	4.940604	-0.573844
C	-3.552249	4.998591	-1.857740
C	2.053909	3.332209	0.298723
C	3.328673	3.073478	-0.507960
C	-1.585639	4.004220	3.359081

C	-2.502692	2.951994	3.587596
C	-3.363612	3.129122	4.822549
N	-2.651086	1.905312	2.797635
C	-3.623783	0.910914	3.056315
C	-3.474433	-0.023688	4.105957
C	-4.446200	-1.017885	4.256296
C	-5.523254	-1.118722	3.385095
C	-5.643470	-0.214655	2.333799
C	-4.706654	0.802524	2.149086
C	-2.277264	-0.015566	5.039409
C	-2.700159	0.073694	6.510710
C	-4.864620	1.816292	1.031039
C	-5.580351	1.272738	-0.202064
C	-1.401070	-1.253323	4.815916
C	-5.539540	3.090234	1.552244
C	-3.223086	6.004969	0.410280
C	2.453654	3.805644	1.702216
Ca	-1.332532	1.328597	0.922714
C	-1.943211	0.226877	-1.238342
C	-2.473615	-0.948666	-1.806597
C	-3.365915	-0.958583	-2.879938
C	-3.807374	0.247369	-3.417851
C	-3.350940	1.443270	-2.867291
C	-2.438985	1.418372	-1.810556
Ca	0.206506	-1.145753	-0.803194
N	-0.331121	-3.490295	-0.749145
C	-0.220251	-4.142576	0.507909
C	-1.315309	-4.163031	1.397831
C	-1.130309	-4.703064	2.671802
C	0.101311	-5.219214	3.063072
C	1.175494	-5.189781	2.179418
C	1.039349	-4.644577	0.900560
C	-2.630423	-3.520008	1.001902

C	-2.629203	-2.048586	1.415656
C	2.214716	-4.570438	-0.054615
C	3.536679	-4.224902	0.631064
C	-0.856066	-4.152968	-1.771952
C	-1.440716	-5.533296	-1.552724
C	-0.927754	-3.646742	-3.086086
C	-0.183512	-2.615811	-3.687778
C	-0.406642	-2.412560	-5.171450
N	0.678119	-1.825433	-3.048590
C	1.670160	-1.164328	-3.823931
C	1.519726	0.178479	-4.224088
C	2.558241	0.796878	-4.927098
C	3.719674	0.108128	-5.250553
C	3.866165	-1.215726	-4.846429
C	2.866937	-1.866077	-4.121377
C	0.277416	0.971375	-3.879494
C	0.614200	2.030524	-2.829074
C	3.067377	-3.289945	-3.634008
C	4.513616	-3.572856	-3.220237
C	-0.366261	1.622014	-5.106716
C	2.608721	-4.324617	-4.669186
C	-3.863684	-4.222264	1.569928
C	2.336948	-5.862487	-0.870231
C	4.405962	-0.542732	0.406615
C	5.620909	-1.221812	0.356036
C	6.467211	-1.161083	1.461155
C	6.100615	-0.427319	2.589168
C	4.879500	0.241474	2.614925
C	4.015591	0.186592	1.521605
C	0.592609	-0.122675	1.581046
C	0.758427	0.835858	2.607796
C	1.409246	0.577928	3.817478
C	1.936205	-0.686646	4.061904

C	1.800699	-1.674422	3.089407
C	1.148337	-1.379311	1.893210
H	-3.732237	4.157769	4.879288
H	-2.772420	2.944991	5.726730
H	-4.215502	2.446769	4.829406
H	-1.554679	4.741658	4.155287
H	0.710139	5.782388	1.756649
H	-0.170594	6.112929	3.266218
H	-0.892463	6.479501	1.692279
H	-1.664725	0.859531	4.801432
H	-3.376475	0.914712	6.691609
H	-1.819971	0.194898	7.153028
H	-3.215360	-0.839893	6.830404
H	-1.973121	-2.175489	4.975651
H	-0.556406	-1.255392	5.512669
H	-0.989645	-1.280529	3.804215
H	-4.343182	-1.739795	5.062866
H	-6.262187	-1.904527	3.516640
H	-6.479464	-0.306132	1.647162
H	-3.859548	2.120811	0.708250
H	-6.541511	2.863512	1.935583
H	-5.638353	3.829875	0.749186
H	-4.956680	3.542508	2.359740
H	-5.105978	0.357041	-0.569451
H	-5.538364	2.011034	-1.008465
H	-6.638161	1.065593	-0.001367
H	-2.899496	3.969646	-0.097277
H	-2.880924	5.807654	1.428253
H	-4.319278	6.007899	0.429684
H	-2.888935	7.005237	0.108949
H	-3.614820	6.019581	-2.252942
H	-4.576293	4.665340	-1.655692
H	-3.129864	4.364380	-2.642760
H	-1.449194	6.322008	-2.557917
H	0.963567	6.389562	-3.082988
H	2.543718	5.021299	-1.791036
H	1.547926	2.362260	0.424493
H	2.912298	4.800879	1.657784
H	3.192059	3.116466	2.127759
H	1.604799	3.845327	2.386480
H	3.116665	2.715781	-1.519086
H	3.937270	2.316664	-0.005869
H	3.942818	3.979109	-0.582273
H	0.390370	1.861986	2.494693
H	1.497501	1.366228	4.561943
H	2.441368	-0.903297	4.999644
H	2.186156	-2.675533	3.259549
H	1.076423	-2.225075	1.204215
H	-0.964872	-3.239481	-5.616760
H	-0.985660	-1.490794	-5.311570
H	0.534518	-2.284790	-5.712112
H	-1.540883	-4.225457	-3.768450
H	-2.350224	-5.485943	-0.943067
H	-1.685176	-6.014358	-2.502142
H	-0.736593	-6.167722	-1.005929
H	-0.460115	0.282474	-3.455263
H	-0.562793	0.882484	-5.890421
H	-1.318147	2.083176	-4.823808
H	0.273559	2.403228	-5.533856
H	1.423806	2.680396	-3.174963
H	-0.243250	2.667842	-2.603090
H	0.954829	1.577200	-1.888032
H	2.447374	1.834772	-5.231405
H	4.512715	0.600967	-5.806978
H	4.783566	-1.745814	-5.085427
H	2.429197	-3.407880	-2.751610

H	3.157801	-4.199937	-5.610718
H	2.791916	-5.340501	-4.298728
H	1.540541	-4.236963	-4.880593
H	4.887548	-2.807392	-2.533121
H	4.579383	-4.545278	-2.720554
H	5.186993	-3.611359	-4.084604
H	1.991105	-3.765518	-0.764334
H	1.445876	-6.029461	-1.482210
H	3.200092	-5.817149	-1.543420
H	2.465716	-6.727893	-0.208748
H	3.893630	-5.038180	1.274329
H	4.308832	-4.043345	-0.123837
H	3.450685	-3.320392	1.241054
H	2.138118	-5.585790	2.491827
H	0.225235	-5.636048	4.059167
H	-1.959883	-4.714771	3.372824
H	-2.707177	-3.553906	-0.088733
H	-3.919356	-4.124249	2.659868
H	-4.772027	-3.764613	1.163038
H	-3.870194	-5.289740	1.321808
H	-1.706541	-1.548744	1.098772
H	-3.479949	-1.510579	0.988867
H	-2.678364	-1.961485	2.501044
H	-2.178691	-1.927386	-1.423399
H	-3.706717	-1.905550	-3.291865
H	-4.503682	0.258172	-4.252756
H	-3.701651	2.390246	-3.268593
H	-2.088780	2.392368	-1.453052
H	3.052356	0.680545	1.554102
Br	3.303180	-0.606254	-1.153448
H	5.898526	-1.786504	-0.527563
H	7.416534	-1.688916	1.434585
H	6.765884	-0.380667	3.446709
H	4.576373	0.804465	3.492217

180

[TS-BC-Br] scf done: -4541.532176 / Sum of electronic and thermal Energies= -4539.884306 / Sum of electronic and thermal Enthalpies= -4539.883362 / Sum of electronic and thermal Free Energies= -4540.099859

C	5.617242	12.188636	8.391338
C	4.335614	11.972422	8.955978
C	3.188508	12.551442	8.362617
C	3.352658	13.447600	7.303265
C	4.614427	13.744455	6.806930
C	5.725073	13.094491	7.330569
N	4.144381	11.151536	10.113463
C	4.108762	11.810959	11.277589
C	4.564132	13.258757	11.334051
C	1.792706	12.157639	8.817088
C	0.760832	12.226512	7.688638
C	6.845202	11.397061	8.829162
C	8.041532	11.555982	7.888859
C	3.587149	11.323748	12.491794
C	2.668822	10.277563	12.763569
C	1.808749	10.540138	13.983537
N	2.495680	9.194503	12.025111
C	1.454445	8.265573	12.321662
C	1.545203	7.331719	13.382995
C	0.555153	6.350749	13.499387
C	-0.499387	6.262761	12.600097
C	-0.595285	7.198151	11.577770
C	0.354251	8.211345	11.435336
C	2.662582	7.338602	14.413357
C	2.126902	7.654828	15.818144
C	0.173143	9.248789	10.345822
C	0.330560	8.642077	8.948787
C	3.406776	5.997986	14.465565
C	-1.172103	9.968575	10.485597

C	1.284412	12.962040	10.020636
C	7.325712	11.654704	10.262030
Ca	3.909273	8.783683	10.116685
C	3.922660	7.784956	7.793960
C	3.030518	6.947818	7.079603
C	2.204191	7.410369	6.054994
C	2.204818	8.763552	5.710062
C	3.058296	9.633924	6.379911
C	3.899115	9.133302	7.378147
Ca	5.967750	6.248213	7.971563
N	5.091769	4.035045	8.184517
C	5.098542	3.446584	9.477254
C	4.068273	3.725787	10.400222
C	4.151766	3.217104	11.697721
C	5.229800	2.433111	12.088235
C	6.240303	2.152089	11.172982
C	6.199224	2.648357	9.867529
C	2.920050	4.632878	10.020514
C	3.125078	6.006982	10.654515
C	7.311874	2.349661	8.881280
C	8.621973	1.945160	9.554350
C	4.353711	3.476363	7.225149
C	3.448404	2.308056	7.564913
C	4.329506	3.886941	5.880241
C	5.192456	4.748659	5.165585
C	4.999843	4.747382	3.662706
N	6.111797	5.523288	5.718905
C	7.152208	6.068049	4.916125
C	7.063668	7.351742	4.341037
C	8.156771	7.832530	3.611285
C	9.317783	7.086322	3.464463
C	9.407614	5.834858	4.066289
C	8.343586	5.312610	4.800680
C	5.833555	8.229851	4.482138
C	6.191779	9.590964	5.089185
C	8.449890	3.947765	5.454235
C	9.854165	3.634716	5.974853
C	5.130122	8.434644	3.134800
C	7.978635	2.841465	4.501489
C	1.543276	4.084650	10.397587
C	6.902611	1.284705	7.855637
C	8.892241	6.877443	9.373773
C	9.330852	5.562469	9.118647
C	10.365640	5.023421	9.874768
C	11.016396	5.773780	10.854613
C	10.636096	7.105687	11.036196
C	9.617773	7.674757	10.284452
C	6.990239	6.887784	10.279617
C	6.548971	8.169645	10.666917
C	6.065697	8.442388	11.955829
C	5.961471	7.398534	12.877239
C	6.360902	6.109482	12.510605
C	6.888363	5.869855	11.243022
H	1.385633	11.547597	13.924505
H	2.414745	10.493868	14.895208
H	0.991540	9.823689	14.070572
H	3.660764	12.037558	13.306889
H	5.437759	13.439938	10.707514
H	4.790782	13.554498	12.361242
H	3.769655	13.913719	10.959207
H	3.379169	8.115812	14.127598
H	1.547953	8.580576	15.845479
H	2.955504	7.746775	16.530348
H	1.474839	6.847676	16.171726
H	2.733548	5.186917	14.765092
H	4.215162	6.039231	15.205380

H	3.847920	5.732255	13.503529
H	0.618921	5.632383	14.312535
H	-1.245232	5.479363	12.702467
H	-1.430301	7.151304	10.883174
H	0.954803	10.001137	10.484626
H	-2.014303	9.281581	10.343499
H	-1.263281	10.761788	9.735608
H	-1.272181	10.421945	11.477506
H	1.304481	8.160325	8.804057
H	0.234446	9.405489	8.171162
H	-0.428994	7.873043	8.765323
H	1.865152	11.110148	9.129655
H	1.863247	12.757821	10.923665
H	0.240604	12.703952	10.234457
H	1.327752	14.037843	9.811097
H	0.530162	13.260781	7.407694
H	-0.178566	11.770064	8.017456
H	1.100097	11.694922	6.794596
H	2.480798	13.911916	6.852931
H	4.731350	14.451420	5.989895
H	6.697807	13.291440	6.895337
H	6.547876	10.339556	8.762725
H	7.631680	12.700558	10.382796
H	8.200305	11.028946	10.473871
H	6.566171	11.428868	11.010862
H	7.783886	11.339250	6.848515
H	8.830035	10.858242	8.184020
H	8.456841	12.569917	7.941009
H	6.687644	9.001881	9.974839
H	5.786110	9.456461	12.248951
H	5.595291	7.596336	13.878829
H	6.274593	5.291371	13.221144
H	7.257710	4.872450	11.008769

H	4.619862	3.786589	3.305869
H	4.262181	5.516585	3.403242
H	5.925197	4.986735	3.134760
H	3.619455	3.350918	5.259214
H	2.538140	2.659270	8.064716
H	3.150510	1.764900	6.665355
H	3.939363	1.614601	8.252920
H	5.128264	7.727797	5.153980
H	4.893794	7.481635	2.652872
H	4.196059	8.989786	3.277829
H	5.763152	9.006393	2.445183
H	6.961902	10.099025	4.497848
H	5.315774	10.243915	5.128966
H	6.573087	9.483042	6.108492
H	8.093496	8.814468	3.149183
H	10.154399	7.480879	2.893789
H	10.323838	5.259827	3.968418
H	7.759699	3.949637	6.306839
H	8.574470	2.849882	3.580510
H	8.086129	1.856040	4.969310
H	6.927761	2.968333	4.229251
H	10.252873	4.451448	6.584972
H	9.829814	2.725842	6.587268
H	10.559843	3.449297	5.156293
H	7.488640	3.275963	8.320805
H	6.098977	1.635275	7.204428
H	7.756264	1.028096	7.217690
H	6.571299	0.369410	8.360864
H	8.557472	0.941300	9.991468
H	9.428686	1.922643	8.813801
H	8.904769	2.647169	10.344261
H	7.080721	1.541007	11.486012
H	5.285196	2.040479	13.100224

H	3.363115	3.443124	12.410762
H	2.947065	4.766114	8.938023
H	1.421392	4.014110	11.483553
H	0.757135	4.754703	10.032796
H	1.380284	3.088748	9.972497
H	4.164713	6.330172	10.508118
H	2.409353	6.714367	10.217872
H	2.935845	5.974137	11.726973
H	2.990286	5.881464	7.297990
H	1.551972	6.716014	5.529336
H	1.551722	9.129975	4.921786
H	3.096802	10.689044	6.122493
H	4.589948	9.855253	7.818788
H	9.332334	8.709574	10.437276
Br	8.347497	7.905517	7.619105
H	8.861288	4.967731	8.341926
H	10.680311	4.004144	9.670138
H	11.825990	5.343115	11.435323
H	11.150736	7.725848	11.766666

180

[C-Br] scf done: -4541.694682 / Sum of electronic and thermal Energies= -4540.043728 / Sum of electronic and thermal Enthalpies= -4540.042784 / Sum of electronic and thermal Free Energies= -4540.258301

C	1.450447	4.446399	0.043397
C	0.047810	4.414610	0.223972
C	-0.815155	5.018391	-0.717223
C	-0.256024	5.714476	-1.792442
C	1.121351	5.803841	-1.948047
C	1.959431	5.162033	-1.042886
N	-0.535587	3.725006	1.325343
C	-0.723696	4.394420	2.454255
C	-0.216054	5.820716	2.613131
C	-2.325973	4.912810	-0.577358
C	-3.045413	4.944796	-1.927461
C	2.391790	3.691105	0.968797
C	3.679927	3.247149	0.269798
C	-1.463133	3.937506	3.564999
C	-2.386626	2.881978	3.712686
C	-3.325161	3.081706	4.890271
N	-2.503674	1.827657	2.913080
C	-3.572852	0.906521	3.109119
C	-3.589924	-0.053288	4.153607
C	-4.669191	-0.941635	4.231883
C	-5.707414	-0.915440	3.313686
C	-5.684899	0.025345	2.289280
C	-4.638635	0.940644	2.172083
C	-2.522338	-0.156823	5.234084
C	-3.097080	0.149948	6.626585
C	-4.698547	2.028550	1.114340
C	-5.534025	1.659378	-0.107480
C	-1.877475	-1.551708	5.289154
C	-5.191010	3.347186	1.723194
C	-2.914771	5.986989	0.346654
C	2.755014	4.453828	2.249824
Ca	-1.155058	1.468191	0.909657
C	-2.273173	0.141711	-0.973632
C	-3.057742	-0.937153	-1.436638
C	-3.926421	-0.857368	-2.528758
C	-4.089399	0.356008	-3.190471
C	-3.354739	1.460827	-2.767566
C	-2.453499	1.334035	-1.708387
Ca	-0.212046	-1.342765	-1.182533
N	-0.383040	-3.675438	-0.818310
C	-0.259221	-4.220098	0.488109
C	-1.354136	-4.238797	1.381242
C	-1.151406	-4.656501	2.700282
C	0.101083	-5.059900	3.141399

C	1.174202	-5.049788	2.256036
C	1.022287	-4.634415	0.931300
C	-2.719419	-3.722669	0.975744
C	-2.938187	-2.324475	1.557300
C	2.208715	-4.627394	-0.018541
C	3.560673	-4.619885	0.698634
C	-0.854402	-4.434677	-1.811056
C	-1.465988	-5.781703	-1.481829
C	-0.808061	-4.094937	-3.175597
C	-0.035100	-3.118090	-3.854489
C	0.119323	-3.365348	-5.341811
N	0.541773	-2.079757	-3.273601
C	1.547868	-1.327408	-3.939786
C	1.278108	0.001861	-4.345985
C	2.312755	0.753584	-4.904639
C	3.589543	0.225867	-5.066799
C	3.845371	-1.075204	-4.660052
C	2.845310	-1.866740	-4.087147
C	-0.115368	0.592769	-4.232361
C	-0.125438	2.117875	-4.143586
C	3.203754	-3.268533	-3.629848
C	4.318728	-3.231501	-2.580954
C	-1.007581	0.114769	-5.383922
C	3.622728	-4.157183	-4.807481
C	-3.862208	-4.654582	1.387192
C	2.149362	-5.813696	-0.991745
C	4.113006	-0.202510	1.584582
C	4.368694	-0.535975	0.248211
C	5.654630	-0.436396	-0.272317
C	6.705519	-0.008272	0.537954
C	6.461920	0.322275	1.870774
C	5.173645	0.229056	2.390476
C	2.731399	-0.256222	2.097739

C	2.205887	0.806237	2.844185
C	0.862166	0.824953	3.215397
C	0.036700	-0.249067	2.867733
C	0.565586	-1.350552	2.186219
C	1.898727	-1.342520	1.792287
H	-3.714789	4.104011	4.885453
H	-2.781617	2.948055	5.831825
H	-4.167293	2.389565	4.874151
H	-1.499985	4.655600	4.379841
H	0.540209	6.085599	1.875233
H	0.198088	5.964468	3.615752
H	-1.046097	6.526548	2.504021
H	-1.741371	0.580699	5.017260
H	-3.617777	1.108577	6.661935
H	-2.294783	0.165732	7.373756
H	-3.812348	-0.624898	6.926045
H	-2.607243	-2.301491	5.616834
H	-1.052405	-1.557581	6.010608
H	-1.478374	-1.883192	4.328370
H	-4.688902	-1.672058	5.036707
H	-6.530291	-1.620712	3.393611
H	-6.500147	0.054376	1.573868
H	-3.677889	2.214143	0.755501
H	-6.178632	3.210702	2.179368
H	-5.276298	4.115557	0.946901
H	-4.505142	3.718184	2.488219
H	-5.232701	0.694082	-0.523196
H	-5.399373	2.414796	-0.887239
H	-6.603948	1.623110	0.131025
H	-2.543641	3.944702	-0.107074
H	-2.653998	5.802631	1.390255
H	-4.008709	5.992859	0.276727
H	-2.551526	6.981590	0.062556

H	-3.083216	5.960160	-2.339663	H	4.379456	0.831282	-5.503564
H	-4.079314	4.602084	-1.810146	H	4.843234	-1.489109	-4.784148
H	-2.548120	4.306429	-2.664012	H	2.321038	-3.715409	-3.162204
H	-0.905960	6.191392	-2.519959	H	4.559942	-3.802285	-5.252598
H	1.541849	6.352582	-2.786811	H	3.781474	-5.189613	-4.473609
H	3.033279	5.202392	-1.194412	H	2.865920	-4.163301	-5.596814
H	1.855001	2.786521	1.269895	H	4.017632	-2.661262	-1.697817
H	3.117846	5.463308	2.021529	H	4.582142	-4.245425	-2.260739
H	3.559653	3.928198	2.779805	H	5.227375	-2.768577	-2.982095
H	1.908643	4.539464	2.933585	H	2.130526	-3.715879	-0.627950
H	3.473108	2.764525	-0.688807	H	1.288615	-5.745325	-1.660508
H	4.213458	2.526104	0.896933	H	3.050725	-5.843177	-1.614150
H	4.358838	4.092289	0.098439	H	2.088711	-6.759008	-0.439265
H	2.843025	1.651107	3.085103	H	3.754443	-5.579693	1.192141
H	0.447962	1.681702	3.744224	H	4.366446	-4.457343	-0.021592
H	-1.007073	-0.240805	3.161110	H	3.630305	-3.833599	1.457906
H	-0.051243	-2.223078	1.993483	H	2.151471	-5.360471	2.609843
H	2.305182	-2.190614	1.252365	H	0.245483	-5.374949	4.171210
H	-0.871105	-3.457943	-5.801825	H	-1.987949	-4.650992	3.393791
H	0.666081	-2.561542	-5.837494	H	-2.732352	-3.649080	-0.116050
H	0.644601	-4.309175	-5.520229	H	-3.978992	-4.693521	2.476037
H	-1.281868	-4.815684	-3.834997	H	-4.809650	-4.292787	0.972050
H	-2.428746	-5.657194	-0.974068	H	-3.696132	-5.677409	1.031513
H	-1.627499	-6.378488	-2.381950	H	-2.172663	-1.618064	1.216229
H	-0.820973	-6.341438	-0.797607	H	-3.906161	-1.906443	1.268786
H	-0.575228	0.222664	-3.306706	H	-2.902241	-2.352312	2.648754
H	-1.109542	-0.973285	-5.383923	H	-2.997923	-1.903976	-0.938306
H	-2.009743	0.546012	-5.289503	H	-4.479266	-1.737222	-2.850912
H	-0.584816	0.419991	-6.348840	H	-4.773797	0.440543	-4.030646
H	0.201543	2.581832	-5.081619	H	-3.469138	2.410894	-3.281407
H	-1.144763	2.465157	-3.956011	H	-1.834712	2.215545	-1.499361
H	0.511853	2.477696	-3.330443	H	4.986696	0.477302	3.432238
H	2.121315	1.775144	-5.214484	Br	1.159685	1.003631	-0.756805

H 3.539947 -0.807098 -0.397481

H 5.827535 -0.675984 -1.317498

H 7.709929 0.073806 0.131476

H 7.277842 0.652282 2.508266

158

[D-Br] scf done: -4078.518289 / Sum of electronic and thermal Energies= -4077.062965 / Sum of electronic and thermal Enthalpies= -4077.062021 / Sum of electronic and thermal Free Energies= -4077.258666

C 0.837717 3.733120 -0.694521

C -0.280778 4.134139 0.068854

C -1.136370 5.154930 -0.415699

C -0.850139 5.750698 -1.644550

C 0.257437 5.367696 -2.394982

C 1.091274 4.365870 -1.915396

N -0.641415 3.432412 1.249422

C -0.487327 4.041259 2.428359

C 0.371240 5.286044 2.494588

C -2.395886 5.554655 0.333503

C -3.630827 4.977130 -0.366251

C 1.748595 2.610740 -0.241751

C 1.817265 1.503790 -1.297967

C -1.103848 3.650087 3.626179

C -2.101060 2.675262 3.865745

C -2.821131 2.815793 5.190329

N -2.423887 1.720570 3.012284

C -3.449389 0.780817 3.288215

C -3.215662 -0.317034 4.149312

C -4.246313 -1.239834 4.346147

C -5.469720 -1.112353 3.696217

C -5.669568 -0.057290 2.813510

C -4.672185 0.894237 2.588734

C -1.878000 -0.536666 4.830087

C -1.997825 -0.478896 6.357908

C -4.925657 2.047653 1.636115

C -5.333452 1.565661 0.240319

C -1.261644 -1.871425 4.398364

C -5.963227 3.012753 2.219454

C -2.528919 7.071230 0.501746

C 3.153444 3.110130 0.106779

Ca -1.338721 1.214621 1.017422

C -1.815303 0.397426 -1.337143

C -2.559462 -0.565112 -2.055637

C -3.492538 -0.238226 -3.039195

C -3.741847 1.101003 -3.339215

C -3.032654 2.091694 -2.666577

C -2.086459 1.733242 -1.703204

Ca 0.086568 -1.236249 -1.084368

N -0.523676 -3.484755 -0.918268

C -0.734379 -3.990076 0.398517

C -2.020366 -4.018334 0.977497

C -2.175476 -4.558811 2.255251

C -1.084780 -5.044348 2.965165

C 0.189167 -4.945249 2.417442

C 0.390243 -4.408227 1.144007

C -3.200480 -3.376345 0.279424

C -3.359869 -1.947968 0.810632

C 1.789536 -4.267100 0.573012

C 2.847310 -4.049281 1.654612

C -0.722567 -4.308626 -1.945579

C -1.357711 -5.660299 -1.691266

C -0.342919 -4.050484 -3.277968

C 0.487648 -3.041425 -3.811900

C 1.075192 -3.339061 -5.175561

N 0.825354 -1.927621 -3.178876

C 1.822899 -1.076801 -3.724613

C 1.448482 0.114282 -4.392475

C 2.460289 0.952537 -4.866675

C	3.805203	0.656236	-4.664260
C	4.157601	-0.490242	-3.964343
C	3.183064	-1.369346	-3.486077
C	-0.014882	0.486061	-4.562708
C	-0.235543	1.985520	-4.761360
C	3.599329	-2.580021	-2.672185
C	4.155831	-2.139781	-1.313131
C	-0.694657	-0.299317	-5.690185
C	4.615015	-3.458644	-3.409082
C	-4.512281	-4.148777	0.427472
C	2.162287	-5.445700	-0.333720
H	-3.251521	3.819583	5.273787
H	-2.114048	2.697445	6.018677
H	-3.619827	2.081778	5.306598
H	-0.882247	4.281620	4.481070
H	-0.137699	6.137116	2.029531
H	1.304307	5.137180	1.941805
H	0.606099	5.551822	3.527782
H	-1.201203	0.261972	4.508872
H	-2.446241	0.458706	6.698978
H	-1.008858	-0.574716	6.820937
H	-2.620406	-1.300417	6.732332
H	-1.860088	-2.717076	4.755096
H	-0.250968	-1.978749	4.807716
H	-1.186813	-1.955114	3.312098
H	-4.079390	-2.082195	5.013027
H	-6.257116	-1.842394	3.864061
H	-6.618923	0.033330	2.291508
H	-3.988689	2.610116	1.543115
H	-6.930590	2.514545	2.355142
H	-6.113970	3.869054	1.552157
H	-5.639906	3.388945	3.195972
H	-4.573588	0.914076	-0.204493

H	-5.472977	2.417193	-0.434046
H	-6.275675	1.006267	0.268875
H	-2.356291	5.111615	1.332592
H	-1.641006	7.503835	0.975456
H	-3.397469	7.307929	1.127180
H	-2.670529	7.576306	-0.460735
H	-3.734798	5.386866	-1.378551
H	-4.543268	5.217105	0.192052
H	-3.560188	3.889340	-0.453216
H	-1.511064	6.524810	-2.026465
H	0.463457	5.842127	-3.350620
H	1.953996	4.059643	-2.502103
H	1.330507	2.183715	0.676546
H	3.645740	3.547429	-0.769945
H	3.773667	2.281825	0.468305
H	3.116671	3.875154	0.889326
H	0.805050	1.228572	-1.626336
H	2.345136	0.628447	-0.900459
H	2.352103	1.818097	-2.198568
H	1.286599	-2.429459	-5.741845
H	2.024118	-3.875079	-5.053545
H	0.407417	-3.980275	-5.757195
H	-0.584485	-4.844358	-3.976289
H	-2.377118	-5.545762	-1.307347
H	-1.394423	-6.262681	-2.601308
H	-0.798872	-6.209061	-0.925834
H	-0.533084	0.206878	-3.637593
H	-0.713318	-1.370636	-5.479208
H	-1.732283	0.034874	-5.800848
H	-0.177589	-0.140592	-6.644561
H	0.157412	2.332221	-5.725276
H	-1.307363	2.198762	-4.747183
H	0.229691	2.575599	-3.965041

H	2.194033	1.865730	-5.388599
H	4.573760	1.327265	-5.039102
H	5.207211	-0.708100	-3.782795
H	2.707777	-3.186966	-2.485538
H	5.571432	-2.940980	-3.547524
H	4.813849	-4.372877	-2.837929
H	4.247986	-3.747106	-4.399532
H	3.410592	-1.593280	-0.722148
H	4.473790	-3.007077	-0.725516
H	5.022078	-1.479600	-1.437941
H	1.784459	-3.372953	-0.064227
H	1.493203	-5.512412	-1.196298
H	3.185576	-5.330278	-0.711802
H	2.110226	-6.391000	0.219987
H	3.003310	-4.950520	2.258899
H	3.809822	-3.800366	1.196945
H	2.563303	-3.228263	2.321010
H	1.042441	-5.282584	2.996957
H	-1.220939	-5.466955	3.957068
H	-3.163027	-4.583642	2.707105
H	-2.968318	-3.319623	-0.789036
H	-4.875450	-4.136017	1.461297
H	-5.290176	-3.690514	-0.193846
H	-4.400412	-5.195039	0.121653
H	-2.423420	-1.391880	0.678937
H	-4.148994	-1.404825	0.282058
H	-3.593451	-1.955674	1.878201
H	-2.417265	-1.631822	-1.859131
H	-4.027835	-1.024887	-3.566272
H	-4.474024	1.366527	-4.097670
H	-3.194739	3.140787	-2.900371
H	-1.523629	2.558849	-1.259787
Br	0.785458	-0.570714	1.559419

170

[E-Br] scf done: -4323.517626 / Sum of electronic and thermal Energies= -4321.961551 / Sum of electronic and thermal Enthalpies= -4321.960607 / Sum of electronic and thermal Free Energies= -4322.169698

C	0.877340	4.660690	0.023581
C	-0.475007	4.250046	0.080297
C	-1.290187	4.302986	-1.078954
C	-0.729432	4.758170	-2.271866
C	0.608790	5.133490	-2.344651
C	1.398498	5.080995	-1.204909
N	-1.046814	3.716700	1.269014
C	-1.446501	4.537793	2.229582
C	-1.231417	6.035904	2.118232
C	-2.763074	3.932421	-1.005987
C	-3.365782	3.539172	-2.353389
C	1.761198	4.748165	1.257030
C	3.031764	3.902546	1.134040
C	-2.120685	4.151693	3.403996
C	-2.709809	2.930520	3.761934
C	-3.637183	2.994069	4.960151
N	-2.571529	1.782099	3.101851
C	-3.411246	0.709565	3.506705
C	-2.900330	-0.312832	4.338929
C	-3.757575	-1.355429	4.703322
C	-5.060385	-1.426464	4.217461
C	-5.531815	-0.440384	3.359052
C	-4.729187	0.646353	3.004368
C	-1.437741	-0.317612	4.759823
C	-1.112693	0.715599	5.844694
C	-5.264481	1.718983	2.069430
C	-5.265698	1.239457	0.612731
C	-0.951711	-1.697855	5.199975
C	-6.662545	2.198830	2.471895
C	-3.594902	5.051543	-0.366058

C	2.164098	6.203198	1.543505
Ca	-1.271563	1.332674	1.176711
Br	-1.723877	0.417171	-1.479823
Ca	0.548681	-1.169835	-1.018139
N	-0.269750	-3.399755	-0.979981
C	-0.411922	-4.016236	0.290737
C	-1.575985	-3.802846	1.058949
C	-1.647456	-4.343178	2.345490
C	-0.594690	-5.080330	2.871730
C	0.552641	-5.283884	2.109585
C	0.668486	-4.763237	0.818364
C	-2.691880	-2.904955	0.565060
C	-2.626481	-1.591985	1.341203
C	1.925253	-4.973039	-0.009906
C	3.123866	-5.431674	0.818671
C	-0.907600	-3.938968	-2.017048
C	-1.794198	-5.149363	-1.805389
C	-0.814183	-3.486448	-3.344315
C	0.102110	-2.600554	-3.942425
C	0.020260	-2.524823	-5.454126
N	0.991114	-1.869379	-3.281256
C	2.053508	-1.245037	-3.985827
C	1.914196	0.045698	-4.536916
C	3.044203	0.671730	-5.071272
C	4.281899	0.041594	-5.082560
C	4.400162	-1.251516	-4.579631
C	3.300327	-1.917356	-4.034620
C	0.570885	0.747647	-4.605048
C	0.544884	2.065643	-3.831047
C	3.417530	-3.349292	-3.532393
C	4.839920	-3.746414	-3.129814
C	0.167536	1.006075	-6.063931
C	2.895379	-4.352303	-4.570919
C	-4.087846	-3.515191	0.716134
C	1.697316	-5.951343	-1.170003
C	3.770242	-0.145251	-0.174600
C	3.928637	-1.448079	-0.637519
C	4.541622	-2.385581	0.192534
C	4.984643	-2.019180	1.460316
C	4.830637	-0.703670	1.894362
C	4.224255	0.249045	1.078550
C	0.792278	-0.200989	1.425431
C	1.209908	0.993118	2.057633
C	1.866099	1.047528	3.290809
C	2.203498	-0.139570	3.933245
C	1.861877	-1.353446	3.335822
C	1.149589	-1.372540	2.135903
H	-4.677334	3.072771	4.622050
H	-3.417974	3.868845	5.577157
H	-3.572730	2.094738	5.576751
H	-2.339485	4.972524	4.079272
H	-2.138898	6.570702	2.413446
H	-0.957941	6.341839	1.107514
H	-0.435350	6.348859	2.802561
H	-0.847950	-0.039199	3.875339
H	-1.281733	1.736448	5.496732
H	-0.056927	0.632227	6.127296
H	-1.720648	0.544862	6.741766
H	-1.434963	-2.020461	6.130536
H	0.124654	-1.660933	5.385686
H	-1.130579	-2.455404	4.430459
H	-3.393035	-2.147404	5.348842
H	-5.701786	-2.257320	4.499806
H	-6.542589	-0.510893	2.965636
H	-4.591748	2.580136	2.126899
H	-7.417953	1.417152	2.330320

H	-6.959770	3.056431	1.857431
H	-6.693510	2.504411	3.523367
H	-4.259984	0.999492	0.250102
H	-5.671979	2.013298	-0.049142
H	-5.876430	0.336076	0.496683
H	-2.854783	3.060206	-0.345168
H	-3.294150	5.239254	0.666484
H	-4.656727	4.777015	-0.360664
H	-3.486646	5.983524	-0.933456
H	-3.473143	4.407108	-3.015441
H	-4.366179	3.120193	-2.200674
H	-2.759616	2.782237	-2.858208
H	-1.343889	4.812729	-3.164428
H	1.030083	5.469963	-3.288008
H	2.438576	5.392782	-1.260080
H	1.184934	4.372104	2.109970
H	2.840741	6.574329	0.764873
H	2.692209	6.273154	2.502121
H	1.302632	6.873925	1.575608
H	2.795283	2.862183	0.913129
H	3.608685	3.941247	2.066266
H	3.677120	4.268774	0.327345
H	1.031367	1.963833	1.577978
H	2.121381	2.008353	3.732555
H	2.722937	-0.121967	4.888336
H	2.119494	-2.291709	3.822178
H	0.856577	-2.354069	1.760247
H	-0.718109	-1.763722	-5.733168
H	0.974789	-2.240052	-5.901412
H	-0.306469	-3.475175	-5.884070
H	-1.466966	-3.997451	-4.043646
H	-2.740673	-4.860350	-1.337687
H	-2.020311	-5.640972	-2.753943
H	-1.317787	-5.871624	-1.136195
H	-0.178092	0.088611	-4.154181
H	0.227298	0.097373	-6.670046
H	-0.858990	1.388126	-6.112150
H	0.822884	1.754682	-6.525416
H	1.325923	2.752895	-4.175790
H	-0.421417	2.561358	-3.961712
H	0.666881	1.921160	-2.754631
H	2.948363	1.671687	-5.487490
H	5.150830	0.547715	-5.494822
H	5.365373	-1.747735	-4.612790
H	2.762423	-3.439925	-2.655180
H	3.428429	-4.231657	-5.521529
H	3.051559	-5.378706	-4.218327
H	1.827398	-4.226570	-4.754158
H	5.318068	-3.004539	-2.482817
H	4.821377	-4.705381	-2.599069
H	5.480191	-3.880097	-4.009994
H	2.175681	-4.000330	-0.457268
H	0.971279	-5.565914	-1.888001
H	2.636378	-6.123678	-1.709636
H	1.341169	-6.918025	-0.794124
H	2.966717	-6.435994	1.229616
H	4.016830	-5.486396	0.186043
H	3.330780	-4.748265	1.649100
H	1.371796	-5.854175	2.534834
H	-0.660991	-5.489611	3.876329
H	-2.535741	-4.167102	2.946997
H	-2.523181	-2.680936	-0.491920
H	-4.358830	-3.631836	1.771370
H	-4.834679	-2.855380	0.260387
H	-4.161059	-4.498346	0.238946
H	-1.598106	-1.205723	1.326700

H	-3.319632	-0.858390	0.920654
H	-2.894302	-1.754599	2.384865
H	4.089263	1.264848	1.424139
Br	2.853272	1.090075	-1.305812
H	3.615363	-1.704847	-1.643422
H	4.676042	-3.399500	-0.165179
H	5.452747	-2.755723	2.106752
H	5.170942	-0.410368	2.882645

170

[TS-EF-Br] scf done: -4323.474024 / Sum of electronic and thermal Energies= -4321.919674 / Sum of electronic and thermal Enthalpies= -4321.918730 / Sum of electronic and thermal Free Energies= -4322.129385

C	6.249422	11.959224	9.668161
C	4.871244	11.650350	9.582382
C	4.215728	11.630910	8.322673
C	4.967028	11.872186	7.172682
C	6.332544	12.128150	7.244285
C	6.956508	12.181551	8.482675
N	4.113332	11.229951	10.709799
C	3.606855	12.097902	11.572996
C	3.969361	13.565224	11.494252
C	2.709457	11.421867	8.236084
C	2.219383	10.989258	6.856137
C	6.969973	12.152545	10.993040
C	8.214353	11.272284	11.153855
C	2.685111	11.767667	12.583367
C	1.978159	10.574536	12.804844
C	0.773904	10.710135	13.715949
N	2.238152	9.405696	12.222149
C	1.340142	8.329500	12.469332
C	1.659503	7.362518	13.452018
C	0.791454	6.284728	13.630919
C	-0.352091	6.139268	12.849998
C	-0.641644	7.080849	11.871275
C	0.187868	8.187525	11.668057
C	2.939922	7.485347	14.261519
C	2.808561	8.471214	15.430007
C	-0.148551	9.190170	10.578759
C	0.023085	8.566158	9.188965
C	3.463079	6.145541	14.782893
C	-1.562078	9.760995	10.735260
C	1.947107	12.676194	8.685115
C	7.389999	13.623560	11.153629
Ca	3.773371	8.925535	10.489400
Br	3.672344	7.811961	7.896191
Ca	6.003018	6.192161	7.813480
N	5.062249	4.044416	8.113779
C	5.142906	3.473254	9.410446
C	4.180941	3.773039	10.397395
C	4.334497	3.244603	11.681868
C	5.421462	2.443090	12.002475
C	6.380229	2.168123	11.030398
C	6.265517	2.670448	9.732384
C	3.028788	4.717354	10.121802
C	3.256182	6.022907	10.884002
C	7.328663	2.385784	8.685010
C	8.661120	1.938055	9.285346
C	4.266327	3.474775	7.209387
C	3.366692	2.330900	7.630090
C	4.199979	3.830280	5.850892
C	5.043054	4.657512	5.078651
C	4.793398	4.615066	3.585142
N	5.994237	5.430254	5.577118
C	6.943499	6.074864	4.742382
C	6.680609	7.333706	4.162012
C	7.718731	7.993389	3.497276
C	8.983988	7.431011	3.392748

C	9.225297	6.174475	3.940916
C	8.221990	5.477436	4.614953
C	5.301766	7.967015	4.195180
C	5.296305	9.330293	4.889117
C	8.470408	4.086141	5.172043
C	9.938616	3.813975	5.501598
C	4.737407	8.106918	2.774214
C	7.946848	3.007071	4.214688
C	1.662720	4.138865	10.500457
C	6.864303	1.361378	7.641891
C	8.445251	7.277674	8.840420
C	8.762852	6.048065	8.180124
C	9.648973	5.159866	8.785290
C	10.352785	5.505338	9.934764
C	10.250858	6.827504	10.398075
C	9.376644	7.729289	9.829079
C	6.855611	7.142118	10.033220
C	6.421763	8.348575	10.618268
C	6.120569	8.439837	11.984559
C	6.185160	7.295502	12.786204
C	6.566442	6.077335	12.218473
C	6.919546	6.010531	10.871991
H	-0.108105	10.948235	13.109109
H	0.912954	11.526525	14.429443
H	0.550179	9.790756	14.259849
H	2.360740	12.606873	13.189777
H	3.104268	14.191489	11.728670
H	4.344024	13.834466	10.504720
H	4.749774	13.796875	12.227688
H	3.696352	7.898794	13.582163
H	2.634335	9.490928	15.082110
H	3.729674	8.477197	16.025414
H	1.981452	8.183832	16.090099
H	2.801166	5.720727	15.546468
H	4.441496	6.286330	15.255014
H	3.581987	5.405575	13.984835
H	1.018619	5.533667	14.380837
H	-1.007664	5.285623	12.998924
H	-1.527911	6.958979	11.253679
H	0.555635	10.025073	10.663579
H	-2.325106	8.990689	10.574247
H	-1.734879	10.555702	10.000470
H	-1.719037	10.178456	11.735122
H	1.037762	8.188386	9.021124
H	-0.188226	9.300531	8.403544
H	-0.661199	7.720274	9.053251
H	2.427649	10.625472	8.942521
H	2.144828	12.923669	9.729396
H	0.867071	12.519997	8.577056
H	2.231362	13.535049	8.065961
H	2.329540	11.795555	6.121211
H	1.154109	10.740695	6.906326
H	2.755085	10.107207	6.498356
H	4.483723	11.851175	6.202792
H	6.904801	12.291581	6.335405
H	8.018965	12.403699	8.534046
H	6.270621	11.898887	11.797998
H	8.187414	13.871457	10.443376
H	7.776700	13.805244	12.163484
H	6.563018	14.313849	10.970753
H	7.968868	10.210221	11.158095
H	8.717714	11.503217	12.100336
H	8.933118	11.449001	10.345672
H	6.412131	9.251700	10.000496
H	5.850711	9.400748	12.427078
H	5.957145	7.360676	13.844772

H 6.597900 5.178005 12.827350

H 7.273933 5.061825 10.472013

H 3.980727 5.307473 3.335151

H 5.676081 4.918839 3.018269

H 4.486129 3.616490 3.262724

H 3.456924 3.287903 5.276024

H 2.549877 2.696593 8.260095

H 2.931462 1.828609 6.763630

H 3.920193 1.596952 8.223463

H 4.637388 7.307902 4.764525

H 4.769226 7.159044 2.229268

H 3.697392 8.453025 2.807857

H 5.314041 8.838830 2.196090

H 5.998074 10.025324 4.412817

H 4.295774 9.772690 4.834852

H 5.559573 9.250466 5.945685

H 7.527445 8.966484 3.051195

H 9.779720 7.964273 2.879396

H 10.212562 5.733309 3.844503

H 7.882485 3.996364 6.094669

H 8.424999 3.101803 3.232315

H 8.167288 2.007685 4.608056

H 6.865843 3.078618 4.078916

H 10.383512 4.622284 6.089945

H 10.025758 2.881838 6.071903

H 10.538190 3.689105 4.591829

H 7.501283 3.330059 8.151702

H 6.029533 1.736418 7.046963

H 7.684197 1.131653 6.951110

H 6.559541 0.426103 8.126966

H 8.589207 0.935280 9.723762

H 9.423584 1.888003 8.499902

H 9.013983 2.628751 10.058079

H 7.235171 1.553813 11.293319

H 5.528713 2.037260 13.005153

H 3.590434 3.470880 12.442025

H 3.023772 4.957715 9.054468

H 1.580352 3.983006 11.581427

H 0.865466 4.833587 10.215432

H 1.479497 3.178054 10.009110

H 4.240657 6.431935 10.623893

H 2.441729 6.719494 10.647196

H 3.241575 5.858352 11.961781

H 9.299378 8.744049 10.199985

Br 7.792891 8.679322 7.560561

H 8.420578 5.852993 7.165748

H 9.832598 4.206277 8.302561

H 11.048078 4.807989 10.390597

H 10.887823 7.165561 11.212400

170

[F-Br] scf done: -4323.635677 / Sum of electronic and thermal Energies= -4322.077413 / Sum of electronic and thermal Enthalpies= -4322.076468 / Sum of electronic and thermal Free Energies= -4322.284786

C 1.118276 4.754176 0.275752

C -0.223146 4.312977 0.275413

C -0.976280 4.311856 -0.924574

C -0.369094 4.750504 -2.100898

C 0.952391 5.183975 -2.109829

C 1.679286 5.189061 -0.927762

N -0.833630 3.736876 1.425467

C -1.287322 4.494984 2.411667

C -1.039629 5.990535 2.423288

C -2.435483 3.884672 -0.919376

C -2.950700 3.416023 -2.277015

C 1.980822 4.761214 1.524356

C 3.256666 3.927814 1.342214

C -2.065750 4.031498 3.490158

C	-2.816914	2.849162	3.638864
C	-3.943595	2.993867	4.650273
N	-2.665240	1.730896	2.934065
C	-3.660406	0.716277	3.083266
C	-3.499092	-0.317330	4.039564
C	-4.498088	-1.287031	4.152470
C	-5.620850	-1.273549	3.332899
C	-5.759067	-0.271372	2.383001
C	-4.799650	0.736623	2.245811
C	-2.286941	-0.382476	4.953055
C	-2.450586	0.487961	6.207480
C	-5.053280	1.859527	1.255850
C	-5.346459	1.342128	-0.154526
C	-1.947405	-1.808277	5.396874
C	-6.210154	2.750305	1.728751
C	-3.329166	4.995006	-0.353574
C	2.376595	6.187372	1.933529
Ca	-1.047305	1.416421	1.166745
Br	-1.975857	-0.128939	-1.077095
Ca	0.661714	-1.272996	-1.333510
N	0.101660	-3.542912	-1.090639
C	0.312658	-4.245739	0.120922
C	-0.374692	-3.882076	1.296265
C	-0.054461	-4.519081	2.499111
C	0.924445	-5.503657	2.549376
C	1.597997	-5.861776	1.384032
C	1.313287	-5.247485	0.163232
C	-1.431476	-2.800632	1.274216
C	-0.943034	-1.547279	2.008827
C	2.060134	-5.629080	-1.102198
C	3.522950	-6.003951	-0.853781
C	-0.779110	-4.002328	-1.982669
C	-1.809601	-5.021889	-1.546897
C	-0.849000	-3.581161	-3.322048
C	0.032021	-2.762851	-4.068165
C	-0.140706	-2.816727	-5.570260
N	0.951840	-1.978057	-3.526056
C	1.909840	-1.296454	-4.324110
C	1.602962	-0.064490	-4.938828
C	2.612433	0.602631	-5.640691
C	3.893015	0.077172	-5.738518
C	4.189485	-1.132746	-5.116407
C	3.219345	-1.830785	-4.396536
C	0.223158	0.561235	-4.859119
C	0.254936	1.888340	-4.094713
C	3.544783	-3.146124	-3.711710
C	5.038144	-3.345170	-3.447779
C	-0.379980	0.773365	-6.253716
C	3.002557	-4.344947	-4.501421
C	-2.776817	-3.274439	1.825584
C	1.349710	-6.763159	-1.852075
C	2.869141	-0.635968	2.103835
C	2.543986	-1.893945	1.577294
C	3.166441	-2.351758	0.412460
C	4.132950	-1.569580	-0.225048
C	4.496752	-0.341060	0.324304
C	3.868432	0.119003	1.476230
C	2.129052	-0.068290	3.251949
C	1.633150	1.245817	3.178907
C	0.928037	1.807039	4.242410
C	0.756036	1.078186	5.418871
C	1.250032	-0.223011	5.505112
C	1.908193	-0.803648	4.422039
H	-4.707138	3.663243	4.240512
H	-3.572405	3.463684	5.566572
H	-4.423287	2.049405	4.905103

H	-2.329362	4.807837	4.203048
H	-1.954030	6.525197	2.696180
H	-0.694443	6.354140	1.454301
H	-0.281419	6.238239	3.174470
H	-1.432115	0.016080	4.395253
H	-2.381758	1.551426	5.975736
H	-1.665554	0.252219	6.935100
H	-3.418135	0.299063	6.687359
H	-2.645739	-2.169931	6.160955
H	-0.945789	-1.827526	5.836935
H	-1.966793	-2.517686	4.565972
H	-4.391534	-2.078033	4.887968
H	-6.380245	-2.044515	3.432802
H	-6.638325	-0.257322	1.744504
H	-4.152581	2.481934	1.215991
H	-7.153034	2.190141	1.730959
H	-6.330564	3.608760	1.057640
H	-6.047315	3.127622	2.741299
H	-4.525176	0.734619	-0.542809
H	-5.505104	2.184603	-0.836730
H	-6.258246	0.733893	-0.174724
H	-2.543275	3.030766	-0.234430
H	-3.050578	5.249273	0.672051
H	-4.378809	4.678180	-0.350406
H	-3.245598	5.899400	-0.967687
H	-2.983139	4.235199	-3.004872
H	-3.969053	3.031170	-2.174419
H	-2.331250	2.609224	-2.676342
H	-0.931116	4.747925	-3.028493
H	1.412314	5.513038	-3.037740
H	2.712922	5.525785	-0.937992
H	1.395191	4.315114	2.336094
H	3.027151	6.635324	1.173159

H	2.931343	6.176192	2.879589
H	1.510842	6.843103	2.050937
H	3.036217	2.941896	0.927795
H	3.774763	3.807839	2.301740
H	3.949834	4.421853	0.651168
H	1.811576	1.825059	2.278824
H	0.510276	2.806739	4.151353
H	0.232713	1.521332	6.259391
H	1.125455	-0.789571	6.423986
H	2.290809	-1.818465	4.493391
H	-0.892410	-2.080631	-5.878413
H	0.789960	-2.575446	-6.088752
H	-0.487396	-3.802152	-5.891658
H	-1.633148	-4.049939	-3.907436
H	-2.611047	-4.504110	-1.005245
H	-2.256579	-5.537843	-2.399808
H	-1.385960	-5.761189	-0.862264
H	-0.432645	-0.122812	-4.308774
H	-0.367574	-0.145201	-6.848721
H	-1.417383	1.117724	-6.170111
H	0.177635	1.534983	-6.811530
H	0.923895	2.608564	-4.580318
H	-0.745803	2.329346	-4.066655
H	0.594411	1.767051	-3.062513
H	2.384986	1.553101	-6.116572
H	4.662842	0.608978	-6.291541
H	5.195553	-1.533816	-5.190284
H	3.022772	-3.138148	-2.745121
H	3.403072	-4.341616	-5.522198
H	3.300809	-5.285225	-4.023055
H	1.912178	-4.332992	-4.556828
H	5.500604	-2.463429	-2.990983
H	5.189679	-4.197462	-2.777364

H	5.583776	-3.559455	-4.374607
H	2.034916	-4.748574	-1.755129
H	0.348573	-6.466297	-2.172580
H	1.916393	-7.043421	-2.747759
H	1.257586	-7.650489	-1.214049
H	3.614858	-6.952483	-0.311643
H	4.041781	-6.131000	-1.810241
H	4.052812	-5.237132	-0.278088
H	2.367437	-6.626983	1.429514
H	1.164622	-5.991166	3.490523
H	-0.582116	-4.236309	3.407076
H	-1.607803	-2.517566	0.233530
H	-2.724388	-3.533038	2.887767
H	-3.529319	-2.487696	1.714026
H	-3.117381	-4.164396	1.286413
H	-0.098675	-1.075000	1.482018
H	-1.780293	-0.846396	2.116192
H	-0.574890	-1.770033	3.013000
H	4.135376	1.086537	1.889556
Br	1.363577	1.252547	-0.398653
H	1.793143	-2.513385	2.059235
H	2.898563	-3.326176	0.019163
H	4.608420	-1.928002	-1.131452
H	5.252266	0.271115	-0.159347

22

[Biphenyl] scf done: -463.166275 / Sum of electronic and thermal Energies= -462.974783 / Sum of electronic and thermal Enthalpies= -462.973839 / Sum of electronic and thermal Free Energies= -463.018285

C	2.971352	-0.111110	1.682235
C	2.908396	-1.414139	1.169356
C	3.500245	-1.729983	-0.050057
C	4.165870	-0.747954	-0.782250
C	4.235262	0.552210	-0.283717
C	3.644523	0.866646	0.936490
C	2.342106	0.224980	2.977019
C	2.391854	-0.670250	4.054633
C	1.798513	-0.353431	5.273055
C	1.144450	0.866581	5.438846
C	1.088374	1.766252	4.375402
C	1.680562	1.447881	3.156941
H	2.924883	-1.610402	3.940661
H	1.855647	-1.057655	6.098945
H	0.681796	1.114453	6.390279
H	0.573043	2.716086	4.491774
H	1.608680	2.141072	2.323077
H	3.726299	1.875053	1.333211
H	2.366381	-2.177222	1.721502
H	3.433031	-2.744411	-0.434407
H	4.627143	-0.994199	-1.734772
H	4.759652	1.322871	-0.842626

158

[9_opt] scf done: -4065.592927 / Sum of electronic and thermal Energies= -4064.134709 / Sum of electronic and thermal Enthalpies= -4064.133765 / Sum of electronic and thermal Free Energies= -4064.325187

Ca	8.890533	8.218537	8.149054
H	9.447610	9.547164	6.523405
N	9.904903	7.923724	10.239492
N	7.227718	9.040290	9.591267
C	10.519265	8.182922	12.593541
H	10.526943	7.118743	12.853374
H	10.217017	8.751748	13.475663
H	11.550670	8.446958	12.334957
C	9.592888	8.434315	11.424663
C	8.422255	9.165276	11.692057
H	8.325317	9.526585	12.709850
C	7.294266	9.372891	10.873660
C	6.094250	10.004599	11.548025
H	6.019357	11.066859	11.289724

H	6.167488	9.927201	12.635200
H	5.164480	9.531066	11.218751
C	10.949268	6.967224	10.146176
C	12.237024	7.359669	9.722378
C	13.199624	6.374249	9.489412
H	14.189872	6.664488	9.149020
C	12.911769	5.027951	9.693379
H	13.671860	4.273349	9.508103
C	11.648178	4.654688	10.138294
H	11.424072	3.602228	10.293477
C	10.651878	5.604244	10.368388
C	12.554101	8.823036	9.494045
H	11.830741	9.408979	10.072673
C	13.957919	9.213055	9.963547
H	14.138523	8.894459	10.996090
H	14.081241	10.300676	9.910776
H	14.734261	8.770131	9.329397
C	12.377909	9.188223	8.019264
H	13.011860	8.564155	7.382402
H	12.644703	10.231996	7.835007
H	11.340711	9.073256	7.680358
C	9.271363	5.162307	10.817481
H	8.673067	6.062060	10.994119
C	6.021082	9.226110	8.874500
C	5.248678	8.082781	8.559421
C	4.060679	8.249909	7.845212
H	3.448052	7.383083	7.615845
C	3.657626	9.507942	7.407880
H	2.731778	9.621019	6.850115
C	4.459269	10.614708	7.663364
H	4.158730	11.589688	7.288432
C	5.647468	10.499369	8.388671
C	5.704860	6.706423	9.011224
H	6.804410	6.703160	9.011236
C	5.249098	5.580376	8.083405
H	4.173037	5.390670	8.175122
H	5.770674	4.652861	8.339381
H	5.467849	5.808855	7.035079
C	5.291360	6.429784	10.461146
H	5.738206	7.158414	11.142986
H	5.619159	5.430956	10.771826
H	4.200918	6.480834	10.564917
C	6.535348	11.713655	8.591316
H	7.289156	11.454536	9.341316
C	5.760933	12.933279	9.101105
H	5.068357	13.316882	8.342734
H	6.454284	13.744934	9.349434
H	5.173854	12.697364	9.995406
C	7.283845	12.054696	7.295548
H	7.916410	11.224202	6.961401
H	7.930032	12.928531	7.444044
H	6.576638	12.291038	6.490427
C	9.492481	6.288040	6.540637
C	8.421349	5.471223	6.094432
H	7.500353	5.931638	5.738858
C	8.484701	4.076833	6.064139
H	7.629847	3.503713	5.712863
C	9.651994	3.425666	6.461568
H	9.711989	2.340026	6.431804
C	8.571977	4.348657	9.724938
H	8.500195	4.905719	8.786202
H	7.559688	4.071674	10.042598
H	9.120373	3.426799	9.503889
C	9.323151	4.371439	12.128998
H	9.870226	3.429053	12.006304
H	8.309138	4.125348	12.465646

H	9.816200	4.940585	12.924312
Ca	10.112804	8.210483	4.957205
N	9.257121	7.811034	2.823093
N	11.907099	8.932775	3.614506
C	8.764626	8.086494	0.440955
H	8.804607	7.036387	0.130910
H	9.081733	8.704989	-0.401797
H	7.716533	8.304122	0.672351
C	9.636396	8.317133	1.655348
C	10.831420	9.027549	1.447136
H	10.988013	9.377715	0.432695
C	11.916818	9.234679	2.322902
C	13.159791	9.835682	1.699538
H	13.213625	10.911073	1.902951
H	13.160093	9.699072	0.615643
H	14.066037	9.386223	2.115394
C	8.199614	6.866485	2.860924
C	6.931871	7.243303	3.349891
C	5.940875	6.267804	3.490195
H	4.963049	6.552621	3.871548
C	6.190686	4.940697	3.161013
H	5.412161	4.191472	3.279073
C	7.447943	4.576169	2.687444
H	7.647355	3.534305	2.450109
C	8.464482	5.517684	2.528389
C	6.661625	8.664737	3.798023
H	7.534673	9.268818	3.522158
C	5.441649	9.284512	3.112613
H	5.552054	9.273268	2.022863
H	5.310007	10.323794	3.435057
H	4.523113	8.741678	3.363341
C	6.504779	8.703498	5.320291
H	5.617022	8.152251	5.641951
H	6.420106	9.725651	5.696643
H	7.368291	8.232672	5.808428
C	9.844364	5.079163	2.073715
H	10.430245	5.977225	1.856478
C	13.066790	9.141078	4.400554
C	13.814537	8.009369	4.807179
C	14.942313	8.201940	5.607943
H	15.532151	7.344096	5.916217
C	15.316918	9.475639	6.026588
H	16.196665	9.607859	6.650721
C	14.545536	10.573726	5.664370
H	14.823292	11.562178	6.021081
C	13.411812	10.432057	4.860680
C	13.402256	6.620253	4.352050
H	12.303651	6.589294	4.331017
C	13.866336	5.505240	5.288414
H	14.947499	5.337518	5.211639
H	13.366774	4.568755	5.020871
H	13.627627	5.725698	6.333885
C	13.863734	6.346931	2.915294
H	13.400872	7.041931	2.210182
H	13.592363	5.329347	2.611935
H	14.953022	6.447428	2.838590
C	12.553898	11.642468	4.536714
H	11.861171	11.357503	3.738997
C	13.381463	12.832190	4.039375
H	14.011546	13.245316	4.835777
H	12.721215	13.636628	3.695768
H	14.039787	12.550164	3.210508
C	11.702804	12.048622	5.746818
H	11.048459	11.234134	6.077168
H	11.068686	12.908224	5.497313
H	12.340665	12.335177	6.592242

C 10.650587 5.571854 6.939151

H 11.514855 6.114048 7.325377

C 10.742424 4.178377 6.892935

H 11.657434 3.685611 7.211940

C 10.559910 4.329061 3.201605

H 10.616508 4.929516 4.114477

H 11.579201 4.059280 2.900971

H 10.025617 3.409043 3.461482

C 9.802218 4.230853 0.799194

H 9.298018 3.271945 0.967250

H 10.820176 4.010787 0.457038

H 9.274721 4.746001 -0.011235

158

[9'_opt] scf done: -4065.585692 / Sum of electronic and thermal Energies= -4064.127397 / Sum of electronic and thermal Enthalpies= -4064.126453 / Sum of electronic and thermal Free Energies= -4064.320099

Ca 3.912888 8.690503 9.861361

N 2.932489 9.409367 11.892942

N 4.497143 10.961685 9.734892

C 2.866116 10.673136 14.009386

H 2.700683 11.734873 14.211459

H 3.634304 10.321380 14.707460

H 1.946869 10.122332 14.218748

C 3.336070 10.464079 12.583735

C 4.189660 11.477973 12.092575

H 4.424811 12.244514 12.823502

C 4.624525 11.769758 10.786581

C 5.237849 13.140340 10.587520

H 6.229628 13.068313 10.128346

H 5.324046 13.681075 11.532177

H 4.622365 13.732325 9.900400

C 1.969742 8.514118 12.426462

C 2.327015 7.499169 13.340484

C 3.745150 7.331178 13.850800

H 4.360860 8.118437 13.404140

C 3.822580 7.461988 15.376946

H 3.373539 8.394254 15.732188

H 4.867267 7.432619 15.708119

H 3.296708 6.634711 15.868668

C 4.330905 5.985627 13.411761

H 3.780721 5.149828 13.860624

H 5.379379 5.907174 13.716669

H 4.298090 5.863370 12.327616

C 1.342400 6.599557 13.761470

H 1.610888 5.817715 14.467469

C 0.036819 6.683640 13.295336

H -0.715180 5.978083 13.638960

C -0.299586 7.669787 12.371713

H -1.315635 7.717520 11.992814

C 0.650652 8.583593 11.915365

C 0.293892 9.641971 10.886667

H 1.171388 9.767451 10.236139

C 0.048549 11.005622 11.543757

H -0.786535 10.945035 12.251825

H -0.198769 11.757978 10.785225

H 0.932970 11.352296 12.083736

C -0.889269 9.253595 10.001268

H -0.745869 8.268666 9.543105

H -1.015498 9.987998 9.199101

H -1.828817 9.231942 10.565724

C 4.788486 11.503281 8.449866

C 3.734008 12.040714 7.682389

C 2.302178 11.945224 8.171591

H 2.336002 11.676506 9.232486

C 1.532163 13.261524 8.054609

H 2.051709 14.070890 8.578944

H 0.532861 13.157450 8.493440

H	1.400412	13.567239	7.010109
C	1.586042	10.815098	7.420159
H	1.536668	11.035336	6.347011
H	0.562196	10.683889	7.787452
H	2.117259	9.859991	7.522476
C	4.023655	12.605706	6.438234
H	3.218476	13.029299	5.843406
C	5.324097	12.628750	5.946142
H	5.534440	13.078587	4.979145
C	6.348470	12.041263	6.682031
H	7.355532	12.019188	6.275674
C	6.100752	11.470634	7.931616
C	7.196046	10.745581	8.683986
H	6.905326	10.702363	9.738292
C	8.566929	11.416397	8.601754
H	8.977081	11.377091	7.586198
H	9.276518	10.899578	9.257794
H	8.518973	12.467246	8.908571
C	7.274510	9.309464	8.157243
H	6.278436	8.848728	8.190912
H	7.954948	8.698683	8.757806
H	7.602985	9.300395	7.116264
C	5.816234	7.126640	10.362281
C	6.351450	8.129089	11.200650
H	5.994080	9.165290	11.144541
C	7.357497	7.896052	12.138716
H	7.722763	8.710723	12.760721
C	7.898483	6.617345	12.270111
H	8.688843	6.425935	12.991715
C	7.404076	5.587435	11.475011
H	7.796585	4.578727	11.575971
C	6.381638	5.850638	10.560178
H	5.993742	4.976299	10.026343

Ca	5.348891	6.318192	7.961428
N	6.165621	5.884531	5.807704
N	4.640698	4.105214	7.616813
C	6.441677	4.959465	3.557669
H	6.165677	4.072992	2.982838
H	6.117793	5.847778	3.005890
H	7.534077	5.013872	3.621056
C	5.839427	4.933286	4.944498
C	4.968979	3.864043	5.234880
H	4.740566	3.210409	4.399968
C	4.460412	3.448079	6.478107
C	3.712009	2.132417	6.489091
H	2.905543	2.144011	7.227617
H	3.293929	1.913465	5.503215
H	4.381843	1.307664	6.758323
C	7.088077	6.895355	5.436759
C	6.653421	8.039263	4.726822
C	5.213597	8.189294	4.271925
H	4.714010	7.226931	4.422526
C	5.111189	8.545930	2.784629
H	5.668294	7.843205	2.154837
H	4.062778	8.534061	2.465360
H	5.502420	9.550539	2.585795
C	4.478384	9.230901	5.121289
H	4.949131	10.214088	5.036948
H	3.434960	9.322383	4.796305
H	4.465531	8.954546	6.179892
C	7.575218	9.056571	4.470938
H	7.246876	9.945780	3.939245
C	8.898614	8.958449	4.892080
H	9.600967	9.760123	4.678999
C	9.311848	7.835885	5.601214
H	10.342049	7.764948	5.942061

C	8.422331	6.798962	5.892689
C	8.895501	5.590266	6.680929
H	8.041925	4.910186	6.781990
C	9.997188	4.830390	5.935050
H	10.895560	5.447212	5.813668
H	10.282263	3.927289	6.487562
H	9.661737	4.528869	4.937015
C	9.358569	5.983583	8.088645
H	8.548943	6.426542	8.679851
H	9.721134	5.107109	8.637174
H	10.172632	6.716596	8.049428
C	4.352564	3.488862	8.857630
C	5.250557	2.531982	9.390676
C	6.451434	2.045042	8.597937
H	6.338768	2.384974	7.564830
C	6.553770	0.516734	8.581277
H	5.621080	0.056009	8.238300
H	7.361169	0.195870	7.912935
H	6.772833	0.115272	9.577643
C	7.747150	2.668250	9.128418
H	7.927289	2.374187	10.169586
H	8.607423	2.344356	8.530347
H	7.704200	3.760369	9.092137
C	5.054954	2.084819	10.698141
H	5.749747	1.363822	11.122162
C	3.998881	2.552555	11.474645
H	3.871437	2.199599	12.494312
C	3.113019	3.479854	10.939598
H	2.292610	3.852377	11.547493
C	3.273148	3.961684	9.637947
C	2.297409	4.981170	9.085558
H	2.736062	5.412340	8.177603
C	0.974536	4.328043	8.671695
H	0.492294	3.850530	9.532963
H	0.284922	5.077895	8.265808
H	1.134319	3.562822	7.905373
C	2.051456	6.124368	10.070194
H	3.000721	6.432285	10.526351
H	1.579930	6.971850	9.557878
H	1.396030	5.844328	10.899797
H	3.738027	7.745112	7.936791

168

[10_opt] scf done: -4296.584459 / Sum of electronic and thermal Energies= -4295.035912 / Sum of electronic and thermal Enthalpies= -4295.034968 / Sum of electronic and thermal Free Energies= -4295.235413

Ca	3.815227	8.766180	9.739653
N	2.860869	9.373666	11.828084
N	4.381181	11.069822	9.834140
C	2.659223	10.528727	14.000136
H	2.431825	11.579627	14.201481
H	3.391396	10.203320	14.748046
H	1.752858	9.935204	14.136040
C	3.233266	10.371148	12.606878
C	4.165626	11.368254	12.232865
H	4.441742	12.046452	13.034193
C	4.625662	11.739596	10.959959
C	5.426243	13.021655	10.884995
H	6.340044	12.869247	10.301488
H	5.694808	13.384341	11.879734
H	4.857559	13.805652	10.374219
C	1.883597	8.434426	12.237188
C	2.180643	7.408935	13.163513
C	3.546907	7.270076	13.807559
H	4.164389	8.111668	13.479944
C	3.465137	7.296842	15.338394
H	2.933387	8.180302	15.705256
H	4.471927	7.295191	15.772320

H	2.941119	6.413098	15.721363
C	4.243451	5.989012	13.337374
H	3.679663	5.098517	13.638418
H	5.246847	5.918975	13.769369
H	4.353589	5.963160	12.251390
C	1.191450	6.463193	13.450583
H	1.410331	5.672113	14.163603
C	-0.052122	6.500768	12.830178
H	-0.804171	5.752074	13.064785
C	-0.323044	7.495746	11.895603
H	-1.290962	7.516311	11.403577
C	0.629296	8.465577	11.582059
C	0.316014	9.576499	10.597445
H	1.271225	9.945775	10.200673
C	-0.322414	10.759939	11.334830
H	-1.270346	10.457594	11.795883
H	-0.522900	11.587097	10.644927
H	0.337562	11.127322	12.127193
C	-0.539976	9.126311	9.413967
H	-0.090026	8.274591	8.892917
H	-0.640469	9.942644	8.691228
H	-1.551890	8.842129	9.724841
C	4.648058	11.701755	8.589918
C	3.700109	12.600020	8.045982
C	2.476918	13.039192	8.832689
H	2.564535	12.636302	9.846357
C	2.385703	14.566080	8.937061
H	3.305815	14.999142	9.343568
H	1.555150	14.854638	9.591920
H	2.210251	15.025479	7.957202
C	1.190349	12.470560	8.226757
H	1.072764	12.784064	7.182448
H	0.312200	12.818571	8.783008

H	1.193906	11.377889	8.252706
C	3.895270	13.080718	6.749652
H	3.162165	13.759304	6.319546
C	5.004912	12.708115	5.997373
H	5.132161	13.084535	4.985933
C	5.949695	11.853209	6.551539
H	6.819047	11.558149	5.968869
C	5.792238	11.346839	7.843759
C	6.831897	10.405157	8.412564
H	6.520293	10.141172	9.426390
C	8.218339	11.046224	8.512426
H	8.605503	11.309741	7.521348
H	8.926230	10.347988	8.974376
H	8.193711	11.958557	9.117894
C	6.900216	9.121636	7.586023
H	5.879411	8.770517	7.378465
H	7.472917	8.352640	8.117527
H	7.372694	9.287538	6.615215
C	5.802094	7.312699	10.266930
C	6.127411	8.280958	11.242725
H	5.663584	9.273647	11.245364
C	7.040082	8.065287	12.275893
H	7.236776	8.855497	12.997282
C	7.688997	6.837550	12.382233
H	8.398455	6.655554	13.185642
C	7.400764	5.839674	11.455925
H	7.868849	4.863035	11.540318
C	6.480952	6.088129	10.435104
H	6.270984	5.225221	9.791732
Ca	5.317467	6.423192	7.909025
N	6.158162	5.831328	5.754747
N	4.786763	4.111698	7.829684
C	6.199016	4.696023	3.561507

H	6.395075	3.645315	3.329337
H	5.418222	5.042689	2.874922
H	7.101479	5.278593	3.366447
C	5.729157	4.840893	4.994937
C	4.828276	3.835847	5.421883
H	4.497812	3.166444	4.634041
C	4.464646	3.447880	6.719484
C	3.665209	2.169136	6.842430
H	2.713323	2.366194	7.348576
H	3.458620	1.726643	5.865544
H	4.194295	1.434016	7.456307
C	7.119945	6.756550	5.278287
C	6.774874	7.793655	4.383668
C	5.368747	7.959458	3.839831
H	4.759845	7.130994	4.213896
C	5.338825	7.931408	2.306910
H	5.824166	7.037885	1.902626
H	4.303258	7.955123	1.947856
H	5.852369	8.804515	1.886996
C	4.734354	9.255336	4.353456
H	5.279219	10.134407	3.990394
H	3.696073	9.332852	4.014907
H	4.726338	9.296056	5.443870
C	7.758746	8.721227	4.026834
H	7.502144	9.521519	3.337311
C	9.046868	8.648941	4.544191
H	9.795566	9.381736	4.254817
C	9.369058	7.638562	5.446274
H	10.371435	7.593117	5.860821
C	8.421240	6.690439	5.830987
C	8.775410	5.574288	6.796318
H	7.859635	5.303609	7.340975
C	9.193003	4.318352	6.021762

H	10.079023	4.524046	5.409350
H	9.431755	3.498348	6.707984
H	8.389881	3.983894	5.358536
C	9.832068	5.968024	7.827817
H	9.555907	6.887863	8.355120
H	9.945473	5.173044	8.571564
H	10.814611	6.122712	7.367012
C	4.612746	3.467038	9.084115
C	5.660576	2.670590	9.601204
C	6.888744	2.343714	8.768912
H	6.784070	2.857442	7.807993
C	6.988665	0.838658	8.492551
H	6.083362	0.457542	8.009654
H	7.839083	0.625026	7.834139
H	7.132454	0.273251	9.421131
C	8.177485	2.848916	9.423480
H	8.312412	2.426109	10.426147
H	9.050695	2.565078	8.824866
H	8.174081	3.938204	9.515209
C	5.546987	2.173860	10.901457
H	6.351064	1.566030	11.310242
C	4.427018	2.441408	11.682539
H	4.363327	2.057482	12.697172
C	3.388143	3.199287	11.154315
H	2.511009	3.408640	11.761473
C	3.458785	3.711823	9.857303
C	2.332405	4.565271	9.312986
H	2.534841	4.742024	8.252699
C	0.961671	3.894113	9.420412
H	0.669754	3.745694	10.466244
H	0.196009	4.522345	8.950650
H	0.955235	2.916444	8.926158
C	2.315161	5.918920	10.023514

H 3.340022 6.312823 10.081178

H 1.667646 6.628732 9.498220

H 1.960741 5.820926 11.051853

C 3.314572 7.874811 7.409514

C 2.898048 6.867369 6.512640

H 3.351743 5.870529 6.521168

C 1.903921 7.050269 5.550792

H 1.637210 6.232069 4.885198

C 1.258918 8.280980 5.445740

H 0.481465 8.434869 4.701551

C 1.636299 9.316332 6.295810

H 1.166998 10.292650 6.211168

C 2.641736 9.103082 7.241274

H 2.926279 9.990405 7.820013

168

[10'_opt] scf done: -4296.591576 / Sum of electronic and thermal Energies= -4295.042348 / Sum of electronic and thermal Enthalpies= -4295.041403 / Sum of electronic and thermal Free Energies= -4295.244602

C 10.698277 4.218361 6.985464

C 9.574175 3.501648 6.578627

C 8.437190 4.193850 6.165131

C 8.437396 5.590687 6.152501

C 9.547886 6.372329 6.565048

C 10.672471 5.615042 6.984796

Ca 10.276365 8.325436 5.053682

N 9.188704 7.982700 3.005268

C 8.138892 7.022750 3.022249

C 6.843631 7.370513 3.459157

C 5.869842 6.374690 3.564083

C 6.155353 5.055288 3.228709

C 7.432584 4.721623 2.791776

C 8.438642 5.684147 2.687753

C 6.539778 8.780343 3.916143

C 6.582546 8.818913 5.444204

C 9.830884 5.273896 2.246361

C 9.837539 4.743997 0.808657

C 9.468261 8.598532 1.857737

C 10.656913 9.297134 1.590380

C 11.868977 9.347981 2.317734

C 13.055051 9.869621 1.531585

C 8.467115 8.512526 0.725608

N 12.008508 8.954863 3.572172

C 13.259946 8.983481 4.234746

C 13.866395 7.742272 4.555366

C 15.053676 7.738492 5.288743

C 15.634177 8.926066 5.725008

C 15.021320 10.135280 5.427050

C 13.835085 10.192343 4.688885

C 13.257254 6.442231 4.061721

C 13.600660 6.222058 2.582677

C 13.204709 11.548022 4.425392

C 12.771033 12.222486 5.730482

C 5.206793 9.327345 3.404583

C 10.429452 4.248292 3.211691

C 13.649654 5.226727 4.900333

C 14.156553 12.470308 3.652190

Ca 8.791703 8.334447 8.050061

N 9.884355 8.037446 10.102084

C 10.946443 7.091168 10.097468

C 12.238445 7.450401 9.660197

C 13.225161 6.465969 9.569984

C 12.955366 5.146948 9.919639

C 11.680807 4.801751 10.355327

C 10.662150 5.752434 10.444719

C 12.525231 8.858563 9.187217

C 12.484664 8.878642 7.658690

C 9.273266 5.329652 10.884848

C	9.265261	4.829702	12.333230
C	9.595911	8.664445	11.241318
C	8.396659	9.348214	11.499376
C	7.183555	9.369071	10.772187
C	5.989585	9.881828	11.552243
C	10.598800	8.609384	12.373787
N	7.049622	8.955717	9.523654
C	5.798525	8.957806	8.860125
C	5.210758	7.704162	8.553482
C	4.024810	7.674518	7.818603
C	3.427895	8.848191	7.367085
C	4.022572	10.069727	7.651370
C	5.206493	10.152752	8.390740
C	5.838986	6.418856	9.061857
C	5.501039	6.210933	10.543933
C	5.816538	11.520503	8.639646
C	6.243002	12.186554	7.327862
C	13.849960	9.428923	9.694745
C	8.696794	4.275572	9.937006
C	5.462807	5.188071	8.238160
C	4.849458	12.437310	9.400432
C	9.522129	10.238821	6.539787
H	10.753024	7.576688	12.703750
H	10.273533	9.204757	13.229730
H	11.575997	8.975043	12.038174
H	8.331577	9.796065	12.486000
H	6.033670	10.973396	11.638061
H	5.996950	9.474675	12.567607
H	5.045879	9.617001	11.071024
H	14.216430	6.736238	9.215209
H	13.733709	4.391544	9.848472
H	11.467092	3.768606	10.618162
H	11.718987	9.501585	9.556024
H	13.911017	9.383829	10.787742
H	13.950945	10.475565	9.385937
H	14.708651	8.885745	9.284442
H	13.292169	8.275545	7.233757
H	12.563273	9.891946	7.262611
H	11.532800	8.450602	7.317775
H	8.627270	6.212102	10.834832
H	3.562489	6.720220	7.587487
H	2.507177	8.806776	6.791198
H	3.563397	10.985220	7.286893
H	6.932132	6.526195	9.008081
H	4.415986	4.902325	8.396442
H	6.084536	4.337954	8.532168
H	5.614575	5.358301	7.167508
H	5.879734	7.034495	11.155119
H	5.944156	5.281065	10.916753
H	4.414690	6.151812	10.680529
H	6.718553	11.387459	9.245354
H	3.983584	12.696119	8.779522
H	5.348597	13.373903	9.675525
H	4.471196	11.967389	10.312993
H	7.022364	11.613557	6.820530
H	6.647236	13.185980	7.519860
H	5.391346	12.291647	6.645122
H	7.535552	6.079600	5.784281
H	7.555419	3.651128	5.833314
H	9.584064	2.414239	6.583823
H	8.634712	4.653322	8.913052
H	7.693402	3.976482	10.260769
H	9.318547	3.373665	9.912383
H	9.891459	3.936867	12.449298
H	8.246309	4.567225	12.641928
H	9.640518	5.593148	13.022828

H	8.333066	7.473706	0.406113
H	8.781347	9.105155	-0.136329
H	7.482795	8.862422	1.057100
H	10.715385	9.732066	0.597626
H	12.998636	10.960180	1.440285
H	13.049872	9.457056	0.518456
H	14.002937	9.618266	2.011963
H	4.876408	6.636394	3.919235
H	5.387102	4.290794	3.311487
H	7.658567	3.688429	2.539567
H	7.337034	9.429213	3.538244
H	5.144688	9.269230	2.312267
H	5.092307	10.375898	3.701950
H	4.356221	8.777158	3.822416
H	5.781814	8.212768	5.877407
H	6.494311	9.836104	5.828146
H	7.539002	8.403873	5.788519
H	10.463727	6.166713	2.273917
H	15.530236	6.793814	5.530307
H	16.556262	8.905085	6.299816
H	15.467768	11.061487	5.780066
H	12.162671	6.534823	4.112531
H	14.700697	4.954178	4.746977
H	13.040714	4.364358	4.615345
H	13.493565	5.407288	5.968652
H	13.213028	7.034165	1.961841
H	13.170017	5.282670	2.219175
H	14.687965	6.175228	2.448814
H	12.303396	11.394735	3.823351
H	15.019642	12.749348	4.268259
H	13.642874	13.396082	3.367412
H	14.540167	11.995661	2.744317
H	12.001182	11.643659	6.245640
H	12.351803	13.213661	5.528196
H	13.622364	12.347605	6.410273
H	11.565321	6.123849	7.348027
H	11.589762	3.695041	7.322432
H	10.492682	4.647713	4.227335
H	11.435089	3.957548	2.887224
H	9.820959	3.338237	3.259114
H	9.227796	3.837234	0.715668
H	10.859016	4.494267	0.497776
H	9.442899	5.485568	0.106084
C	9.693262	11.014817	7.706345
C	9.678269	12.411255	7.714726
C	9.500142	13.106328	6.520725
C	9.332733	12.392805	5.336106
C	9.339022	10.996561	5.363083
H	9.860313	10.536793	8.680346
H	9.808907	12.953119	8.648941
H	9.491797	14.193224	6.513474
H	9.193715	12.920124	4.394806
H	9.179140	10.503402	4.395396

References

1. A. S. S. Wilson, M. S. Hill, M. F. Mahon, C. Dinoi, L. Maron, *Science*, 2017, **358**, 1168-1171.
2. F. F. Blicke and F. D. Smith, *J. Am. Chem. Soc.*, 1929, **51**, 3479-3483.
3. O. V. Dolomanov, L. J. Bourhis, T. J. Gildea, J. A. K. Howard, H. Puschmann, *J. Appl. Cryst.* **2009**, *42*, 339-341.
4. G. M. Sheldrick, *Acta Cryst.* **2015**, *A71*, 3-8.
5. G. M. Sheldrick, *Acta Cryst.* **201**, *C71*, 3-8.
6. Gaussian09, revision D.01. Frisch, M. J.; Trucks, G. W.; Schlegel, H. B.; Scuseria, G. E.; Robb, M. A.; Cheeseman, J. R.; Scalmani, G.; Barone, V.; Mennucci, B.; Petersson, G. A.; Nakatsuji, H.; Caricato, M.; Li, X.; Hratchian, H. P.; Izmaylov, A. F.; Bloino, J.; Zheng, G.; Sonnenberg, J. L.; Hada, M.; Ehara, M.; Toyota, K.; Fukuda, R.; Hasegawa, J.; Ishida, M.; Nakajima, T.; Honda, Y.; Kitao, O.; Nakai, H.; Vreven, T.; Montgomery, Jr., J. A.; Peralta, J. E.; Ogliaro, F.; Bearpark, M.; Heyd, J. J.; Brothers, E.; Kudin, K. N.; Staroverov, V. N.; Keith, T.; Kobayashi, R.; Normand, J.; Raghavachari, K.; Rendell, A.; Burant, J. C.; Iyengar, S. S.; Tomasi, J.; Cossi, M.; Rega, N.; Millam, J. M.; Klene, M.; Knox, J. E.; Cross, J. B.; Bakken, V.; Adamo, C.; Jaramillo, J.; Gomperts, R.; Stratmann, R. E.; Yazyev, O.; Austin, A. J.; Cammi, R.; Pomelli, C.; Ochterski, J. W.; Martin, R. L.; Morokuma, K.; Zakrzewski, V. G.; Voth, G. A.; Salvador, P.; Dannenberg, J. J.; Dapprich, S.; Daniels, A. D.; Farkas, O.; Foresman, J. B.; Ortiz, J. V.; Cioslowski, J.; Fox, D. J.; Gaussian, Inc., Wallingford CT, **2013**.
7. a) J. P. Perdew, J. A. Chevary, S. H. Vosko, K. A. Jackson, M. R. Pederson, D. J. Singh, C. Fiolhais, Phys. Rev. B 1992, 46, 6671; b) A. D. Becke, J. Chem. Phys. 1993, 98, 5648.d) Stephens, P.J.; Devlin, F.J.; Chabalowski, C.F.; Frisch, M.J. *J.Phys.Chem.* **1994**, *98*, 11623-11627.
8. A. Bergner, M. Dolg, W. Kuechle, H. Stoll, H. Preuss, *Mol. Phys.* **1993**, *80*, 1431.
9. L. Maron, C. Teichteil, *Chem. Phys.* **1998**, *237*, 105.
10. Grimme, S.; Ehrlich, S.; Goerigk, L. Effect of the Damping Function in Dispersion Corrected Density Functional Theory. *J. Comput. Chem.* **2011**, *32*, 1456–1465.
11. Reed, A. E.; L. A. Curtiss, L. A.; Weinhold, F. Intermolecular interactions from a natural bond orbital, donor-acceptor viewpoint. *Chem. Rev.* **1988**, *88*, 899–926.
12. Reed, A. E.; Weinhold, F. Natural bond orbital analysis of near-Hartree–Fock water dimer. *J. Chem. Phys.* **1983**, *78*, 4066–4073.
